# Supplementary material for: Assessing the capacity of symptom scores to predict COVID-19 positivity in Nigeria: a national derivation and validation cohort study
Source: BMJ Open. 2021 Sep 3;11(9):e049699. doi: 10.1136/bmjopen-2021-049699 (PMC8421116; doi:10.1136/bmjopen-2021-049699)
Supplement: Supplementary data [file bmjopen-2021-049699supp001.pdf]

Supplementary Materials

**Supplementary Table 1: NCDC COVID-19 case definitions, February 27-August 27, 2020**	
**Case category**	**Definition/criteria**
**Suspected COVID-19 case**	**1. Symptoms with international travel**<br>Anyone with acute respiratory symptoms (fever and either cough, difficulty breathing or shortness of breath) OR new respiratory symptoms (cough, difficulty breathing or shortness of breath) without fever and no other explanation, **AND** a history of travel to or residence in a country reporting cases within 14 days before symptom onset; Or new respiratory symptoms with contact to a confirmed case in the last 14 days before symptom onset;<br>OR<br>**2. Symptoms with contact to confirmed case**<br>Anyone with new respiratory symptoms (cough, difficulty breathing or shortness of breath, with or without fever), **AND** contact with a confirmed or probable COVID-19 case in the last 14 days prior to symptom onset;<br>OR<br>**3. Acute respiratory illness in an area of moderate or high COVID-19 prevalence with no other explanation**<br>Anyone with acute respiratory illness within the last 10 days (fever and either cough, difficulty breathing or shortness of breath), **AND** absence of an alternative diagnosis that explains the clinical presentation **AND** residing or working in the last 14 days in an area identified by NCDC as a moderate or high prevalence region.
**Probable case**	Any suspected case:<br>• For whom testing for COVID-19 is indeterminate test results;<br>• For whom testing was positive on a pan-coronavirus assay; OR<br>• Where samples were not collected prior to the demise of a suspect case
**Confirmed case**	Anyone with laboratory confirmation of SARS-CoV-2 infection with or without signs and symptoms.
**Contact**	Defined as anyone who experienced any one of the following exposures during the 2 days before and/or within 14 days after the onset of symptoms of a probable or confirmed case:<br>• Face-to-face contact with a probable or confirmed case within 1 metre and for more than 15 minutes;<br>• Direct physical contact with a probable or confirmed case;<br>• Direct care for a patient with probable or confirmed COVID-19 disease without using proper personal protective equipment; OR<br>• Other situations as indicated by local risk assessments.<br><br>For confirmed asymptomatic cases, the period of contact is measured as the 2 days before, through the 14 days after the date on which the sample was taken which led to confirmation.

Elimian KO, *et al. BMJ Open* 2022; 11:e049699. doi: 10.1136/bmjopen-2021-049699

Assessment of multicollinearity between clinical symptoms and COVID-19 positivity

The VIF and tolerance values (Supplementary Tables 3a, 3b and 3c for results) from the assessment of multicollinearity between the statistically significant symptoms in the unadjusted models and COVID-19 positivity in children, adults and elderly were less than 10 and greater than 0.1, implying it was appropriate to combine the independent symptoms into an index or clinical score.

Supplementary Table 2a: Findings from the assessment of multicollinearity between clinical signs and symptoms and COVID-19 diagnosis in children

Clinical sign/symptom	Derivation dataset n=7,911	
	**Variance of Inflation Factor (VIF)**	**Tolerance**
Cough	1.02	0.9800
Runny nose	1.03	0.9724
Fatigue	1.01	0.9864
Loss of taste	1.95	0.5132
Loss of smell	1.95	0.5139
Fever	1.01	0.9853
Mean VIF=1.33		

Supplementary Table 2b: Findings from the assessment of multicollinearity between clinical signs and symptoms and COVID-19 diagnosis in adults

Clinical sign/symptom	Derivation dataset n=13,167	
	**Variance of Inflation Factor (VIF)**	**Tolerance**
Cough	1.05	0.9569
Difficulty breathing	1.04	0.9613
Rapid breathing	1.01	0.9901
Runny nose	1.03	0.9734
Gastrointestinal tract symptom	1.02	0.9847
Chest pain	1.02	0.9770
Fatigue	1.02	0.9805
Headache	1.04	0.9609
Loss of taste	1.79	0.5574
Loss of smell	1.79	0.5581
Fever	1.03	0.9678
Mean VIF=1.17		

Supplementary Table 2c: Findings from the assessment of multicollinearity between clinical signs and symptoms and COVID-19 diagnosis in elderly

Clinical sign/symptom	Derivation dataset n=3,369	
	**Variance of Inflation Factor (VIF)**	**Tolerance**
Cough	1.01	0.9871
Difficulty breathing	1.02	0.9800
Loss of smell	1.01	0.9891
Mean VIF=1.01		

Elimian KO, *et al. BMJ Open* 2022; 11:e049699. doi: 10.1136/bmjopen-2021-049699

Supplementary Table 3: Assignment of clinical weights based on clinicians' judgement or experiences from managing COVID-19 cases in Nigeria

Symptom	Weighting based on clinicians' judgement						
	**Delta State**	**FCT**	**Kaduna State**	**Gombe State**	**Infectious disease consultant§**	**Infectious disease consultant¶**	**Average weight‡**
**Children**							
Cough	3	5	3	1	3	3	**3.0**
Runny nose	2	2	4	2	2	2	**2.3**
Fatigue	2	2	2	1	1	1	**1.5**
Loss of taste	1	3	2	1	1	1	**1.5**
Loss of smell	1	3	2	1	1	1	**1.5**
Fever	2	5	3	5	3	5	**3.8**
**Adults**							
Cough	5	5	4	5	3	3	**4.2**
Runny nose	3	1	2	3	2	3	**2.3**
Chest pain	3	1	2	1	3	2	**2.0**
Fatigue	5	1	3	4	2	4	**3.2**
Headache	3	2	4	1	2	1	**2.2**
Loss of taste	4	3	3	4	5	3	**3.7**
Loss of smell	5	3	3	4	5	3	**3.8**
Fever	3	5	4	4	3	5	**4.0**
**Elderly**							
Cough	4	5	4	5	3	4	**4.2**
Breathing difficulty	5	5	4	5	5	5	**4.8**
Loss of smell	5	3	3	5	5	3	**4.0**

FCT: Federal Capital Territory
‡: Sum of weights from various sources divided by 6 (i.e. number of weight sources)
§: Provides care to COVID-19 patients in a health-facility in the FCT
¶: Provides home-care to COVID-19 patients in the FCT

Table 4: Baseline sociodemographic and clinical characteristics of the study participants in relation to COVID-19 infection (derivation dataset)

Variable	Children		Adults		Elderly	
	PCR-confirmed cases [n=2,155 (%)]	PCR positive and negative cases [N=7,911 (%)]	PCR-confirmed cases [n=4,542 (%)]	PCR positive and negative cases [N=13,167 (%)]	PCR-confirmed cases [n=159 (%)]	PCR positive and negative cases [N=399 (%)]
**Socio-demographic features**						
**Sex**						
Female	902 (41.86)	3,065 (38.74)	1,534 (33.77)	4,814 (36.56)	49 (30.82)	148 (37.09)
Male	1,253 (58.14)	4,846 (61.26)†	3,008 (66.23)	8,353 (63.44)‡	110 (69.18)	251 (62.91)†
**Geopolitical zone§**						
South-west	632 (29.33)	1,761 (22.26)	1,285 (28.29)	3,111 (23.63)	47 (29.56)	97 (24.31)
South-south	712 (33.04)	2,823 (35.68)	1,617 (35.60)	5,441 (41.32)	51 (32.08)	164 (41.10)
South-east	46 (2.13)	129 (1.63)	139 (3.06)	294 (2.23)	11 (6.92)	16 (4.01)
North-central	402 (18.65)	1,557 (19.68)	749 (16.49)	2,045 (15.53)	13 (8.18)	32 (8.02)
North-west	315 (14.62)	1,498 (18.94)	609 (13.41)	1,978 (15.02)	20 (12.58)	63 (15.79)
North-east	48 (2.23)	143 (1.81)‡	143 (3.15)	298 (2.26)‡	17 (10.69)	27 (6.77)†
**Setting**						
Rural	111 (5.15)	429 (5.42)	233 (5.13)	755 (5.73)	20 (12.58)	41 (10.28)
Urban	1,015 (47.10)	3,467 (43.83)	2,218 (48.83)	6,485 (49.25)	92 (57.86)	209 (52.38)
Missing	1,029 (47.75)	4,015 (50.75)†	2,091 (46.04)	5,927 (45.01)†	47 (29.56)	149 (37.34)†
**Education**						
None	25 (1.16)	107 (1.35)	62 (1.37)	169 (1.28)	15 (9.43)	31 (7.77)
Nursery	16 (0.74)	70 (0.88)	2 (0.04)	4 (0.03)	0 (0.00)	0 (0.00)
Primary	67 (3.11)	250 (3.16)	41 (0.90)	151 (1.15)	2 (1.26)	5 (1.25)
Secondary	181 (8.40)	681 (8.61)	378 (8.32)	1,098 (8.34)	14 (8.81)	33 (8.27)
Tertiary	692 (32.11)	2,043 (25.82)	1,574 (34.65)	4,071 (30.92)	45 (28.30)	82 (20.55)
Other	98 (4.55)	540 (6.83)	134 (2.95)	336 (2.55)	13 (8.18)	25 (6.27)
Missing	1,076 (49.93)	4,220 (53.34)‡	2,351 (51.76)	7,338 (55.73)‡	70 (44.03)	223 (55.89)†
**Occupation**						
Student/pupil	297 (13.78)	1,401 (17.71)	212 (4.67)	775 (5.89)	1 (0.63)	2 (0.50)
Child/housewife	67 (3.11)	241 (3.05)	81 (1.78)	228 (1.73)	7 (4.40)	23 (5.76)
Business/trading	106 (4.92)	384 (4.85)	397 (8.74)	1,050 (7.97)	6 (3.77)	18 (4.51)
Transporter	6 (0.28)	35 (0.44)	24 (0.53)	87 (0.66)	0 (0.00)	1 (0.25)
Healthcare worker	269 (12.48)	950 (12.01)	479 (10.55)	1,473 (11.19)	4 (2.52)	13 (3.26)
Laboratorian	10 (0.46)	26 (0.33)	12 (0.26)	35 (0.27)	0 (0.00)	0 (0.00)
Farmer	18 (0.84)	92 (1.16)	84 (1.85)	283 (2.15)	16 (10.06)	27 (6.77)
Animal-related worker	3 (0.14)	11 (0.14)	15 (0.33)	40 (0.30)	1 (0.63)	4 (1.00)
Religious/traditional leader	2 (0.09)	11 (0.14)	30 (0.66)	69 (0.52)	2 (1.26)	4 (1.00)

Other	826 (38.33)	2,609 (32.98)	2,008 (44.21)	5,633 (42.78)	85 (53.46)	187 (46.87)
Missing	551 (25.57)	2,151 (27.19)‡	1,200 (26.42)	3,494 (26.54)‡	37 (23.27)	120 (30.08)NS
**Clinical signs and symptoms**						
**Clinical outcome**						
Recovered	1,396 (64.78)	2,221 (28.07)	2,844 (62.62)	3,677 (27.93)	70 (44.03)	89 (22.31)
Dead	15 (0.70)	24 (0.30)	229 (5.04)	252 (1.91)	50 (31.45)	50 (12.53)
No outcome yet	744 (34.52)	5,666 (71.62)‡	1,469 (32.34)	9,238 (70.16)‡	39 (24.53)	260 (65.16)‡
Obvious (visible to healthcare workers on sight)						
**Chills/sweat**						
No	2,119 (98.33)	7,791 (98.48)	4,471 (98.44)	12,978 (98.56)	158 (99.37)	393 (98.50)
Yes	36 (1.67)	120 (1.52)NS	71 (1.56)	189 (1.44)NS	1 (0.63)	6 (1.50)NS
**Cough**						
No	1,162 (53.92)	4,725 (59.73)	2,023 (44.54)	7,179 (54.52)	53 (33.33)	160 (40.10)
Yes	993 (46.08)	3,186 (40.27)‡	2,519 (55.46)	5,988 (45.48)‡	106 (66.67)	239 (59.90)†
**Breathing difficulty**						
No	1,912 (88.72)	7,031 (88.88)	3,778 (83.18)	11,181 (84.92)	102 (64.15)	283 (70.93)
Yes	243 (11.28)	880 (11.12)NS	764 (16.82)	1,986 (15.08)‡	57 (35.85)	116 (29.07)†
**Rapid breathing**						
No	2,123 (98.52)	7,802 (98.62)	4,470 (98.41)	13,008 (98.79)	155 (97.48)	392 (98.25)
Yes	32 (1.48)	109 (1.38)NS	72 (1.59)	159 (1.21)†	4 (2.52)	7 (1.75)NS
**Runny nose**						
No	1,450 (67.29)	5,817 (73.53)	3,386 (74.55)	10,218 (77.60)	130 (81.76)	330 (82.71)
Yes	705 (32.71)	2,094 (26.47)‡	1,156 (25.45)	2,949 (22.40)‡	29 (18.24)	69 (17.29)NS
Askable (can be found out by asking questions of patients/relatives)						
**Abdominal pain/diarrhoea**						
No	2,021 (93.78)	7,371 (93.17)	4,193 (92.32)	12,222 (92.82)	151 (94.97)	372 (93.23)
Yes	134 (6.22)	540 (6.83)NS	349 (7.68)	945 (7.18)NS	8 (5.03)	27 (6.77)NS
**GIT symptoms**						
No	1,942 (90.12)	7,110 (89.87)	4,048 (89.12)	11,859 (90.07)	145 (91.19)	362 (90.73)
Yes	213 (9.88)	801 (10.13)NS	494 (10.88)	1,308 (9.93)†	14 (8.81)	37 (9.27)NS
**Chest pain**						
No	2,089 (96.94)	7,711 (97.47)	4,348 (95.73)	12,759 (96.90)	153 (96.23)	387 (96.99)
Yes	66 (3.06)	200 (2.53)NS	194 (4.27)	408 (3.10)‡	6 (3.77)	12 (3.01)NS
**Fatigue**						
No	2,036 (94.48)	7,543 (95.35)	4,269 (93.99)	12,474 (94.74)	144 (90.57)	372 (93.23)
Yes	119 (5.52)	368 (4.65)†	273 (6.01)	693 (5.26)†	15 (9.43)	27 (6.77)NS
**Headache**						
No	1,829 (84.87)	6,796 (85.91)	3,880 (85.42)	11,592 (88.04)	149 (93.71)	367 (91.98)

Yes	326 (15.13)	1,115 (14.09)NS	662 (14.58)	1,575 (11.96)‡	10 (6.29)	32 (8.02)NS
**Musculoskeletal pain**						
No	2,097 (97.31)	7,710 (97.46)	4,440 (97.75)	12,878 (97.81)	152 (95.60)	386 (96.74)
Yes	58 (2.69)	201 (2.54)NS	102 (2.25)	289 (2.19)NS	7 (4.40)	13 (3.26)NS
**Sore throat**						
No	1,615 (74.94)	5,950 (75.21)	3,476 (76.53)	9,766 (74.17)	129 (81.13)	316 (79.20)
Yes	540 (25.06)	1,961 (24.79)NS	1,066 (23.47)	3,401 (25.83)‡	30 (18.87)	83 (20.80)NS
**Loss of taste**						
No	1,909 (88.58)	7,562 (95.59)	4,111 (90.51)	12,581 (95.55)	153 (96.23)	391 (97.99)
Yes	246 (11.42)	349 (4.41)‡	431 (9.49)	586 (4.45)‡	6 (3.77)	8 (2.01)†
**Loss of smell**						
No	1,851 (85.89)	7,489 (94.67)	4,067 (89.54)	12,545 (95.28)	152 (95.60)	390 (97.74)
Yes	304 (14.11)	422 (5.33)‡	475 (10.46)	622 (4.72)‡	7 (4.40)	9 (2.26)†
Measureable signs/symptoms						
**Fever**						
No	1,126 (52.25)	4,634 (58.58)	2,306 (50.77)	7,723 (58.65)	79 (49.69)	219 (54.89)
Yes	1,029 (47.75)	3,277 (41.41)‡	2,236 (49.23)	5,444 (41.35)‡	80 (50.31)	180 (45.11)NS

†=p-value<0.05; ‡=p<0.001; NS=p>0.05 or Not significant
GIT= gastrointestinal (nausea + vomiting)
Musculoskeletal pain= muscle /joint pain
§=State composition of geopolitical zones in Nigeria: South-West (Ekiti, Lagos, Ogun, Ondo, Osun and Oyo); South-South (Akwa-Ibom, Bayelsa, Cross-River, Rivers, Delta and Edo); South-East (Abia, Anambra, Ebonyi, Enugu and Imo); North-Central (Benue, Kogi, Kwara, Nasarawa, Niger, and Plateau States as well as the FCT); North-West (Jigawa, Kaduna, Kano, Katsina, Kebbi, Sokoto and Zamfara); and North-East (Adamawa, Bauchi, Borno, Gombe, Taraba and Yobe).

Elimian KO, *et al. BMJ Open* 2022; 11:e049699. doi: 10.1136/bmjopen-2021-049699

Table 5: Baseline sociodemographic and clinical characteristics of the study participants in relation to COVID-19 infection (validation dataset)

Variable	Children		Adults		Elderly	
	PCR-confirmed cases [n=2,260 (%)]	PCR positive and negative cases [N=8,077 (%)]	PCR-confirmed cases [n=4,612 (%)]	PCR positive and negative cases [N=13,274 (%)]	PCR-confirmed cases [n= 158 (%)]	PCR positive and negative cases [N=393 (%)]
**Socio-demographic features**						
**Sex**						
Female	871 (38.54)	2,986 (36.97)	1,546 (33.52)	4,808 (36.22)	61 (38.61)	151 (38.42)
Male	1,389 (61.46)	5,091 (63.03)NS	3,066 (66.48)	8,466 (63.78)‡	97 (61.39)	242 (61.58)NS
**Geopolitical zone§**						
South-west	670 (29.65)	1,778 (22.01)	1,307 (28.34)	3,134 (23.61)	39 (24.68)	88 (22.39)
South-south	723 (31.99)	2,866 (35.48)	1,611 (34.93)	5,470 (41.21)	57 (36.08)	176 (44.78)
South-east	44 (1.95)	149 (1.84)	134 (2.91)	315 (2.37)	14 (8.86)	21 (5.34)
North-central	414 (18.32)	1,499 (18.56)	777 (16.85)	2,016 (15.19)	17 (10.76)	44 (11.20)
North-west	343 (15.18)	1,620 (20.06)	649 (14.07)	2,027 (15.27)	25 (15.82)	53 (13.49)
North-east	66 (2.92)	165 (2.04)‡	134 (2.91)	312 (2.35)‡	6 (3.80)	11 (2.80)†
**Setting**						
Rural	119 (5.27)	458 (5.67)	278 (6.03)	807 (6.08)	15 (9.49)	33 (8.40)
Urban	1,033 (45.71)	3,476 (43.04)	2,269 (49.20)	6,603 (49.74)	91 (57.59)	214 (54.45)
Missing	1,108 (49.03)	4,143 (51.29)†	2,065 (44.77)	5,864 (44.18)NS	52 (32.91)	146 (37.15)NS
**Education**						
None	33 (1.46)	110 (1.36)	66 (1.43)	199 (1.50)	10 (6.33)	17 (4.33)
Nursery	14 (0.62)	73 (0.90)	1 (0.02)	3 (0.02)	0 (0.00)	0 (0.00)
Primary	63 (2.79)	270 (3.34)	61 (1.32)	171 (1.29)	6 (3.80)	13 (3.31)
Secondary	204 (9.03)	698 (8.64)	414 (8.98)	1,164 (8.77)	9 (5.70)	25 (6.36)
Tertiary	718 (31.77)	2,023 (25.05)	1,567 (33.98)	4,074 (30.69)	42 (26.58)	86 (21.88)
Other	81 (3.58)	525 (6.50)	127 (2.75)	352 (2.65)	7 (4.43)	20 (5.09)
Missing	1,147 (50.75)	4,378 (54.20)‡	2,376 (51.52)	7,311 (55.08)‡	84 (53.16)	232 (59.03)NS
**Occupation**						
Student/pupil	302 (13.36)	1,408 (17.43)	222 (4.81)	741 (5.58)	0 (0.00)	0 (0.00)
Child/housewife	66 (2.92)	248 (3.07)	97 (2.10)	272 (2.05)	6 (3.80)	19 (4.83)
Business/trading	111 (4.91)	381 (4.72)	360 (7.81)	993 (7.48)	13 (8.23)	29 (7.38)
Transporter	5 (0.22)	45 (0.56)	21 (0.46)	87 (0.66)	0 (0.00)	2 (0.51)
Healthcare worker	332 (14.69)	1,003 (12.42)	494 (10.71)	1,504 (11.33)	3 (1.90)	7 (1.78)
Laboratorian	5 (0.22)	14 (0.17)	14 (0.30)	28 (0.21)	0 (0.00)	0 (0.00)
Farmer	18 (0.80)	100 (1.24)	89 (1.93)	306 (2.31)	8 (5.06)	20 (5.09)

Animal-related worker	5 (0.22)	17 (0.21)	10 (0.22)	40 (0.30)	0 (0.00)	5 (1.27)
Religious/traditional leader	3 (0.13)	13 (0.16)	23 (0.50)	60 (0.45)	4 (2.53)	5 (1.27)
Other	807 (35.71)	2,517 (31.16)	2,064 (44.75)	5,775 (43.51)	79 (50.00)	188 (47.84)
Missing	606 (26.81)	2,331 (28.86)‡	1,218 (26.41)	3,468 (26.13)†	45 (28.48)	118 (30.03)NS
**Clinical signs and symptoms**						
**Clinical outcome**						
Recovered	1,451 (64.20)	2,222 (27.51)	2,850 (61.80)	3,636 (27.39)	80 (50.63)	95 (24.17)
Dead	21 (0.93)	27 (0.33)	256 (5.55)	297 (2.24)	33 (20.89)	34 (8.65)
No outcome yet	788 (34.87)	5,828 (72.16)‡	1,506 (32.65)	9,341 (70.37)‡	45 (28.48)	264 (67.18)‡
Obvious (visible to healthcare workers on sight)						
**Chills/sweat**						
No	2,236 (98.94)	7,973 (98.71)	4,538 (98.40	13,051 (98.32)	157 (99.37)	390 (99.24)
Yes	24 (1.06)	104 (1.29)NS	74 (1.60)	223 (1.68)NS	1 (0.63)	3 (0.76)NS
**Cough**						
No	1,183 (52.35)	4,668 (57.79)	2,107 (45.69)	7,212 (54.33)	61 (38.61)	161 (40.97)
Yes	1,077 (47.65)	3,409 (42.21)‡	2,505 (54.31)	6,062 (45.67)‡	97 (61.39)	232 (59.03)NS
**Breathing difficulty**						
No	2,024 (89.56)	7,252 (89.79)	3,771 (81.76)	11,244 (84.71)	98 (62.03)	258 (65.65)
Yes	236 (10.44)	825 (10.21)NS	841 (18.24)	2,030 (15.29)‡	60 (37.97)	135 (34.35)NS
**Rapid breathing**						
No	2,222 (98.32)	7,981 (98.81)	4,532 (98.27)	13,104 (98.72)	152 (96.20)	379 (96.44)
Yes	38 (1.68)	96 (1.19)†	80 (1.73)	170 (1.28)†	6 (3.80)	14 (3.56)NS
**Runny nose**						
No	1,607 (71.11)	6,018 (74.51)	3,406 (73.85)	10,278 (77.43)	129 (81.65)	328 (83.46)
Yes	653 (28.89)	2,059 (25.49)‡	1,206 (26.15)	2,996 (22.57)‡	29 (18.35)	65 (16.54)NS
Askable (can be found out by asking questions of patients/relatives)						
**Abdominal pain/diarrhoea**						
No	2,121 (93.85)	7,568 (93.70)	4,271 (92.61)	12,358 (93.10)	146 (92.41)	356 (90.59)
Yes	139 (6.15)	509 (6.30)NS	341 (7.39)	916 (6.90)NS	12 (7.59)	37 (9.41)NS
**GIT symptoms**						
No	2,030 (89.82)	7,265 (89.95)	4,052 (87.86)	11,907 (89.70)	137 (86.71)	343 (87.28)
Yes	230 (10.18)	812 (10.05)NS	560 (12.14)	1,367 (10.30)‡	21 (13.29)	50 (12.72)NS
**Chest pain**						
No	2,197 (97.21)	7,866 (97.39)	4,434 (96.14)	12,862 (96.90)	150 (94.94)	379 (96.44)
Yes	63 (2.79)	211 (2.61)NS	178 (3.86)	412 (3.10)‡	8 (5.06)	14 (3.56)NS
**Fatigue**						
No	2,107 (93.23)	7,686 (95.16)	4,314 (93.54)	12,540 (94.47)	148 (93.67)	378 (96.18)
Yes	153 (6.77)	391 (4.84)‡	298 (6.46)	734 (5.53)†	10 (6.33)	15 (3.82)†

**Headache**						
No	1,924 (85.13)	6,916 (85.63)	3,969 (86.06)	11,691 (88.07)	138 (87.34)	371 (94.40)
Yes	336 (14.87)	1,161 (14.37)NS	643 (13.94)	1,583 (11.93)‡	20 (12.66)	22 (5.60)‡
**Musculoskeletal pain**						
No	2,202 (97.43)	7,885 (97.62)	4,461 (96.73)	12,905 (97.22)	154 (97.47)	385 (97.96)
Yes	58 (2.57)	192 (2.38)NS	151 (3.27)	369 (2.78)†	4 (2.53)	8 (2.04)NS
**Sore throat**						
No	1,691 (74.82)	6,018 (74.51)	3,492 (75.72)	9,817 (73.96)	130 (82.28)	333 (84.73)
Yes	569 (25.18)	2,059 (25.49)NS	1,120 (24.28)	3,457 (26.04)‡	28 (17.72)	60 (15.27)NS
**Loss of taste**						
No	2,016 (89.20)	7,721 (95.59)	4,190 (90.85)	12,687 (95.58)	150 (94.94)	379 (96.44)
Yes	244 (10.80)	356 (4.41)‡	422 (9.15)	587 (4.42)‡	8 (5.06)	14 (3.56)NS
**Loss of smell**						
No	1,964 (86.90)	7,671 (94.97)	4,119 (89.31)	12,653 (95.32)	155 (98.10)	388 (98.73)
Yes	296 (13.10)	406 (5.03)‡	493 (10.69)	621 (4.68)‡	3 (1.90)	5 (1.27)NS
Measureable signs/symptoms						
**Fever**						
No	1,224 (54.16)	4,796 (59.38)	2,249 (48.76)	7,711 (58.09)	84 (53.16)	234 (59.54)
Yes	1,036 (45.84)	3,281 (40.62)‡	2,363 (51.24)	5,563 (41.91)‡	74 (46.84)	159 (40.46)†

†=p-value<0.05; ‡=p<0.001; NS=p>0.05 or Not significant
GIT= gastrointestinal (nausea + vomiting)
Musculoskeletal pain= muscle /joint pain
§=State composition of geopolitical zones in Nigeria: South-West (Ekiti, Lagos, Ogun, Ondo, Osun and Oyo); South-South (Akwa-Ibom, Bayelsa, Cross-River, Rivers, Delta and Edo); South-East (Abia, Anambra, Ebonyi, Enugu and Imo); North-Central (Benue, Kogi, Kwara, Nasarawa, Niger, and Plateau States as well as the FCT); North-West (Jigawa, Kaduna, Kano, Katsina, Kebbi, Sokoto and Zamfara); and North-East (Adamawa, Bauchi, Borno, Gombe, Taraba and Yobe).

Elimian KO, *et al. BMJ Open* 2022; 11:e049699. doi: 10.1136/bmjopen-2021-049699

Table 6: Predictive value of individual clinical symptoms for predicting COVID-19 positivity in children (n=8,077)

Variable	ROC area (95% CI)	Sensitivity (%) (95%CI)	Specificity (%) (95%CI)	PPV (%) (95% CI)	NPV (%) (95% CI)
**Cough**	0.54 (0.53-0.55)	**47.7 (45.6-49.7)**	59.9 (58.6-61.2)	31.6 (30.0-33.2)	**74.7 (73.4-75.9)**
**Runny nose**	0.52 (0.51-0.53)	28.9 (27.0-30.8)	75.8 (74.7-76.9)	31.7 (29.7-33.8)	73.3 (72.2-74.4)
**Fatigue**	0.51 (0.51-0.52)	6.8 (5.8-7.9)	95.9 (95.4-96.4)	39.1 (34.3-44.2)	72.6 (71.6-73.6)
**Loss of taste**	0.54 (0.54-0.55)	10.8 (9.5-12.1)	**98.1 (97.7-98.4)**	68.5 (63.4-73.3)	73.9 (72.9-74.9)
**Loss of smell**	**0.56 (0.55-0.56)**	13.1 (11.7-14.6)	**98.1 (97.7-98.4)**	**72.9 (68.3-77.2)**	74.4 (73.4-75.4)
**Fever**	0.54 (0.52-0.55)	45.8 (43.8-47.9)	61.4 (60.1-62.7)	31.6 (30.0-33.2)	74.5 (73.2-75.7)

The best predictive performance measures (AUROC, sensitivity, specificity, PPV and NPV values) for each outcome are highlighted in bold

Table 7: Predictive value of individual clinical symptoms for predicting COVID-19 positivity in adults (n=13,274)

Variable	ROC area (95% CI)	Sensitivity (%) (95%CI)	Specificity (%) (95%CI)	PPV (%) (95% CI)	NPV (%) (95% CI)
Cough	**0.57 (0.56-0.58)**	**54.3 (52.9-55.8)**	58.9 (57.9-60.0)	41.3 (40.1-42.6)	**70.8 (69.7-71.8)**
Runny nose	0.53 (0.52-0.54)	26.1 (24.9-27.4)	79.3 (78.5-80.2)	40.3 (38.5-42.0)	66.9 (65.9-67.8)
Chest pain	0.51 (0.50-0.51)	3.9 (3.3-4.5)	97.3 (96.9-97.6)	43.2 (38.4-48.1)	65.5 (64.7-66.3)
Fatigue	0.51 (0.50-0.51)	6.5 (5.8-7.2)	95.0 (94.5-95.4)	40.6 (37.0-44.3)	65.6 (64.8-66.4)
Headache	0.52 (0.51-0.52)	13.9 (13.0-15.0)	89.1 (88.5-89.8)	40.6 (38.2-43.1)	66.1 (65.2-66.9)
Loss of taste	0.54 (0.53-0.54)	9.2 (8.3-10.0)	98.1 (97.8-98.4)	71.9 (68.1-75.5)	67.0 (66.1-67.8)
Loss of smell	0.55 (0.54-0.55)	10.7 (9.8-11.6)	**98.5 (98.2-98.8)**	**79.4 (76.0-82.5)**	67.4 (66.6-68.3)
Fever	**0.57 (0.56-0.58)**	51.2 (49.8-52.7)	63.1 (62.0-64.1)	42.5 (41.2-43.8)	**70.8 (69.8-71.8)**

Table 8: Predictive value of individual clinical symptoms for predicting COVID-19 positivity in the elderly (n=393)

Variable	ROC area (95% CI)	Sensitivity (%) (95%CI)	Specificity (%) (95%CI)	PPV (%) (95% CI)	NPV (%) (95% CI)
Cough	0.52 (0.47-0.57)	**61.4 (53.3-69.0)**	42.6 (36.1-49.1)	41.8 (35.4-48.4)	**62.1 (54.1-69.6)**
Breathing difficulty	**0.53 (0.48-0.58)**	38.0 (30.4-46.0)	68.1 (61.7-74.0)	44.4 (35.9-53.2)	62.0 (55.8-68.0)
Loss of smell	0.51 (0.49-0.52)	1.9 (0.4-5.4)	**99.1 (97.0-99.9)**	**60.0 (14.7-94.7)**	60.1 (55.0-65.0)

Table 9: Predictive performance of un-weighted clinical score thresholds for predicting COVID-19 positivity in children, adults and elderly

Outcome	Score	ROC area (95% CI)	Sensitivity (%) (95% CI)	Specificity (%) (95% CI)	PPV (%) (95% CI)	NPV (%) (95% CI)
**Children (<17 years) n=8,077**						
**Number of symptoms**	≥1	0.55 (0.54-0.56)	**86.1 (84.6-87.5)**	23.6 (22.5-24.7)	30.4 (29.3-31.6)	**81.3 (79.4-83.2)**
	≥2	**0.58 (0.57-0.59)**	43.3 (41.2-45.3)	72.9 (71.7-74.0)	38.3 (36.4-40.2)	76.8 (75.6-77.9)
	≥3	0.55 (0.54-0.56)	16.6 (15.1-18.2)	93.4 (92.7-94.0)	49.4 (45.8-53.0)	74.2 (73.2-75.2)
	≥4	0.52 (0.52-0.53)	5.1 (4.2-6.1)	**99.5 (99.3-99.7)**	**79.3 (71.8-85.6)**	73.0 (72.0-73.9)
**Adults (17-64 years) n=13,274**						
**Number of symptoms**	≥1	0.56 (0.55-0.56)	**89.7 (88.8-90.6)**	21.7 (20.9-22.6)	37.9 (37.0-38.8)	**79.9 (78.2-81.5)**
	≥2	**0.61 (0.60-0.61)**	51.0 (49.5-52.4)	70.3 (69.3-71.2)	47.7 (46.3-49.1)	72.9 (72.0-73.9)
	≥3	0.56 (0.55-0.57)	22.3 (21.1-23.5)	90.0 (89.4-90.6)	54.3 (52.1-56.6)	68.5 (67.7-69.4)
	≥4	0.53 (0.53-0.54)	8.3 (7.5-9.1)	**98.0 (97.7-98.3)**	**68.8 (64.7-72.6)**	66.7 (65.9-67.6)
**Elderly (>65 years) n=393**						
**Number of symptoms**	≥1	0.52 (0.48-0.57)	**75.3 (67.5-81.8)**	29.4 (23.6-35.6)	41.8 (36.0-47.7)	**63.9 (54.1-72.9)**
	≥2	**0.53 (0.49-0.57)**	25.9 (19.3-33.5)	**80.4 (74.8-85.3)**	**47.1 (36.3-58.1)**	61.8 (56.1-67.2)

The best predictive performance measures (AUROC, sensitivity, specificity, PPV and NPV values) for each age group are highlighted in bold

Table 10: Predictive performance of statistically-derived weighted clinical score thresholds for predicting COVID-19 in children, adults and elderly

Outcome	Score	ROC area (95% CI)	Sensitivity (%) (95% CI)	Specificity (%) (95% CI)	PPV (%) (95% CI)	NPV (%) (95% CI)
**Children (<17 years) n=8,077**						
**Number of symptoms**	≥1	0.55 (0.54-0.56)	**86.1 (84.6-87.5)**	23.6 (22.5-24.7)	30.4 (29.3-31.6)	**81.3 (79.4-83.2)**
	≥2	0.55 (0.54-0.56)	**86.1 (84.6-87.5)**	23.6 (22.5-24.7)	30.4 (29.3-31.6)	**81.3 (79.4-83.2)**
	≥3	0.55 (0.54-0.57)	70.7 (68.8-72.6)	40.3 (39.5-41.5)	31.5 (30.2-32.8)	78.0 (76.4-79.4)
	≥4	**0.59 (0.58-0.60)**	48.1 (46.0-50.2)	**69.4 (68.2-70.6)**	**37.9 (36.1-39.7)**	77.5 (76.3-78.6)
**Adults (17-64 years) n=13,274**						
**Number of symptoms**	≥1	0.56 (0.55-0.56)	**89.7 (88.8-90.6)**	21.7 (20.9-22.6)	37.9 (37.0-38.8)	**79.9 (78.2-81.5)**
	≥2	0.58 (0.57-0.58)	85.6 (84.6-86.6)	30.0 (29.0-30.9)	39.4 (38.5-40.4)	79.6 (78.2-81.0)
	≥3	0.58 (0.57-0.59)	82.9 (81.8-84.0)	33.4 (32.4-34.4)	39.8 (38.9-40.8)	78.6 (77.2-79.9)
	≥4	**0.59 (0.58-0.59)**	80.8 (79.6-81.9)	**36.4 (35.4-37.5)**	**40.4 (39.4-41.4)**	78.1 (76.8-79.3)
**Elderly (>65 years) n=393**						
**Number of symptoms**	≥1	0.52 (0.48-0.57)	75.3 (67.8-81.8)	29.4 (23.6-35.6)	41.8 (36.0-47.7)	63.9 (54.1-72.9)
	≥2	0.52 (0.48-0.57)	75.3 (67.8-81.8)	29.4 (23.6-35.6)	41.8 (36.0-47.7)	63.9 (54.1-72.9)

The best predictive performance measures (AUROC, sensitivity, specificity, PPV and NPV values) for each age group are highlighted in bold

Table 11: Predictive performance of clinically-derived weighted clinical score thresholds for predicting COVID-19 in children, adults and elderly

Outcome	Score	ROC area (95% CI)	Sensitivity (%) (95% CI)	Specificity (%) (95% CI)	PPV (%) (95% CI)	NPV (%) (95% CI)
**Children (<17 years) n=8,077**						
**Number of symptoms**	≥1	0.55 (0.54-0.56)	**86.1 (84.6-87.5)**	23.6 (22.5-24.7)	30.4 (29.3-31.6)	**81.3 (79.4-83.2)**
	≥2	0.54 (0.53-0.55)	81.2 (79.6-82.8)	27.1 (25.9-28.2)	30.2 (29.0-31.4)	78.8 (76.9-80.6)
	≥3	0.56 (0.55-0.57)	74.2 (72.4-76.0)	37.7 (36.5-39.0)	31.7 (30.4-32.9)	79.0 (77.5-80.5)
	≥4	**0.57 (0.56-0.58)**	40.7 (38.6-42.7)	**73.6 (72.4-74.7)**	**37.4 (35.5-39.4)**	76.1 (75.0-77.2)
**Adults (17-64 years) n=13,274**						
**Number of symptoms**	≥1	0.56 (0.55-0.56)	**89.7 (88.8-90.6)**	21.7 (20.9-22.6)	37.9 (37.0-38.8)	**79.9 (78.2-81.5)**
	≥2	0.56 (0.55-0.56)	**89.7 (88.8-90.6)**	21.7 (20.9-22.6)	37.9 (37.0-38.8)	**79.9 (78.2-81.5)**
	≥3	**0.58 (0.58-0.59)**	82.5 (81.3-83.5)	34.1 (33.1-35.1)	40.0 (39.0-41.0)	78.5 (77.1-79.8)
	≥4	**0.58 (0.58-0.59)**	79.0 (77.8-80.2)	**37.9 (36.9-39.0**	**40.4 (39.4-41.4)**	77.2 (75.9-78.5)
**Elderly (>65 years) n=393**						
**Number of symptoms**	≥1	0.52 (0.48-0.57)	75.3 (67.8-81.8)	29.4 (23.6-35.6)	41.8 (36.0-47.7)	63.9 (54.1-72.9)
	≥2	0.52 (0.48-0.57)	75.3 (67.8-81.8)	29.4 (23.6-35.6)	41.8 (36.0-47.7)	63.9 (54.1-72.9)

The best predictive performance measures (AUROC, sensitivity, specificity, PPV and NPV values) for each age group are highlighted in bold

Unweighted score **Statistically weighted score** **Clinically weighted score**

Childre
n

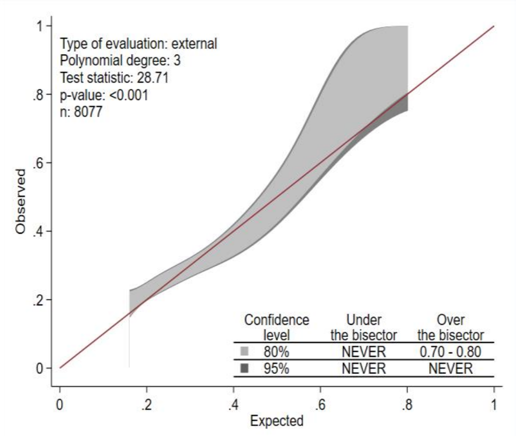

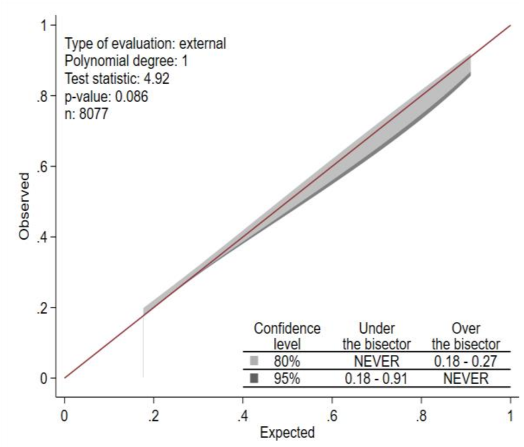

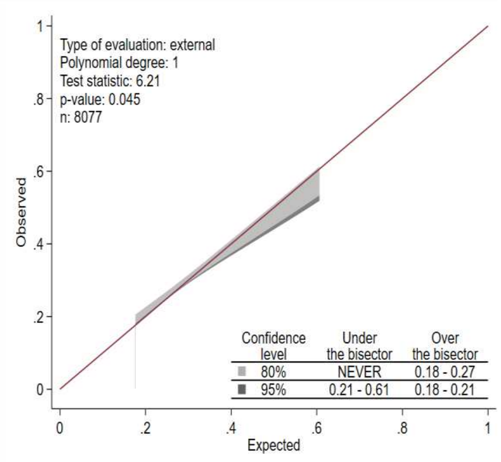

Adults

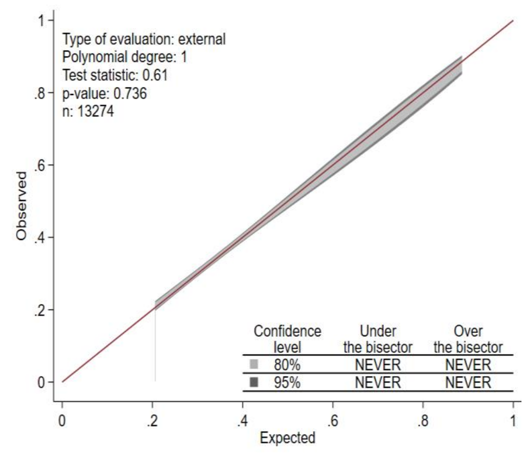

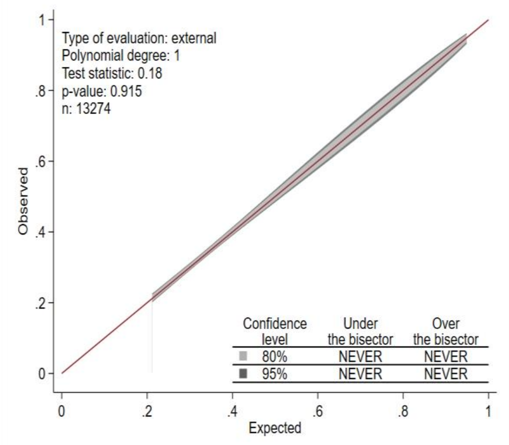

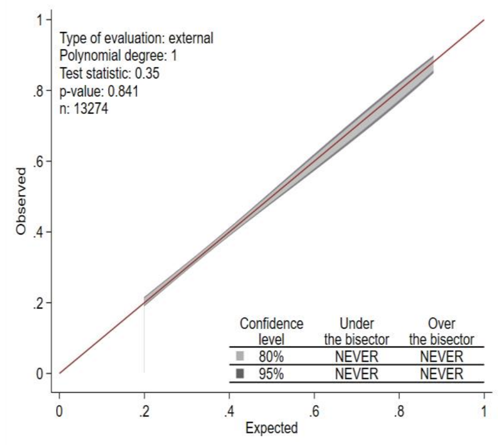

Elimian KO, *et al. BMJ Open* 2022; 11:e049699. doi: 10.1136/bmjopen-2021-049699

Elderly

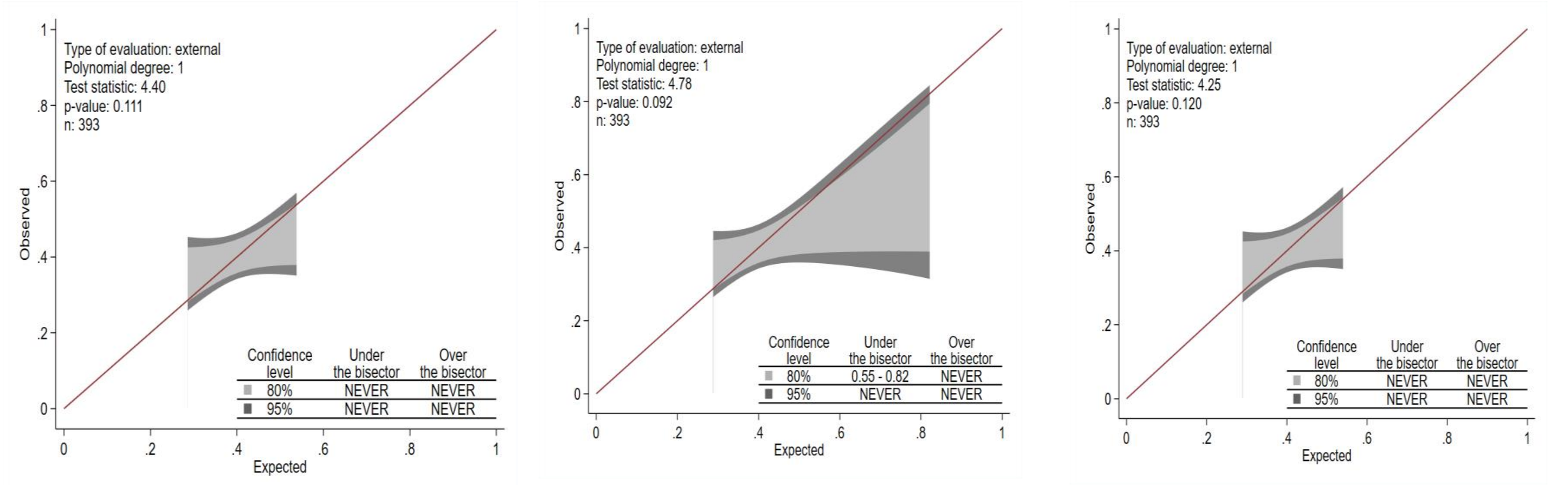

Supplementary Figure: Calibration belts and tests for children (upper panel), adults (middle panel), and elderly (lower panel)

Elimian KO, *et al. BMJ Open* 2022; 11:e049699. doi: 10.1136/bmjopen-2021-049699
