# Supplementary material for: Assessing the capacity of symptom scores to predict COVID-19 positivity in Nigeria: a national derivation and validation cohort study
Source: BMJ Open. 2021 Sep 3;11(9):e049699. doi: 10.1136/bmjopen-2021-049699 (PMC8421116; doi:10.1136/bmjopen-2021-049699)
Supplement: Author's manuscript [file bmjopen-2021-049699.draft_revisions.pdf]

BMJ Open is committed to open peer review. As part of this commitment we make the peer review history of every article we publish publicly available.

When an article is published we post the peer reviewers' comments and the authors' responses online. We also post the versions of the paper that were used during peer review. These are the versions that the peer review comments apply to.

The versions of the paper that follow are the versions that were submitted during the peer review process. They are not the versions of record or the final published versions. They should not be cited or distributed as the published version of this manuscript.

BMJ Open is an open access journal and the full, final, typeset and author-corrected version of record of the manuscript is available on our site with no access controls, subscription charges or pay-per-view fees (http://bmjopen.bmj.com).

If you have any questions on BMJ Open's open peer review process please email
info.bmjopen@bmj.com

BMJ Open

Symptom scores have poor predictive capacity for COVID-19 positivity: national derivation and validation cohort study in Nigeria

Journal:	*BMJ Open*
Manuscript ID	bmjopen-2021-049699
Article Type:	Original research
Date Submitted by the Author:	02-Feb-2021

Complete List of Authors:	Elimian, Kelly; Nigeria Centre for Disease Control; Karolinska Institutet, Department of Global Public Health<br>Aderinola, Olaolu; Nigeria Centre for Disease Control<br>Gibson, Jack; University of Nottingham, Department of Epidemiology and Public Health<br>Myles, Puja; University of Nottingham Faculty of Medicine and Health Sciences, Epidemiology & Public Health<br>Ochu, Chinwe; Nigeria Centre for Disease Control<br>King, Carina; Karolinska Institute, Department of Global Public Health<br>Okwor, Tochi; Nigeria Centre for Disease Control, Prevention Programmes and Knowledge Management<br>Gaudenzi, Giulia; Karolinska Institutet, Department of Global Public Health<br>Olayinka, Adebola; World Health Organization<br>Zaiyad, Habib; University of Abuja Teaching Hospital<br>Ohonsi, Cornelius; Nigeria Centre for Disease Control<br>Ebhodaghe, Blessing; Nigeria Centre for Disease Control<br>Ipadeola oipadeola, Oladipupo ; University of Ilorin<br>Dan-Nwafor, Chioma; Nigeria Centre for Disease Control<br>Nwachukwu , William ; Nigeria Centre for Disease Control<br>Abdus-salam, Ismail; Lagos State Ministry of Health<br>Akande, Oluwatosin; University of Ilorin Teaching Hospital, Department of Epidemiology and Community Health<br>Falodun, Olanrewaju; National Hospital, Department of Internal Medicine<br>Arinze, Chinedu; Nigeria Centre for Disease Control<br>Ezeokafor, Chidiebere; National Agency for the Control of AIDS<br>Jafiya, Abubakar; Nigeria Centre for Disease Control<br>Ojimba, Anastacia; Federal Medical Centre Asaba<br>Aremu, John; Federal Teaching Hospital Gombe<br>Joseph, Emmanuel; Kaduna State Infectious Disease Control Center Community Medicine<br>Bowale, Abimbola; Mainland Hospital Yaba<br>Mutiu, Babatunde; Lagos State Government Ministry of Health<br>Saka, Babatunde; Lagos State Government Ministry of Health<br>Jinadu, Arisekola; Nigeria Centre for Disease Control<br>Hamza, Khadeejah; Ahmadu Bello University, Department of Community Medicine<br>Ibeh, Christian; Nnamdi Azikiwe University Teaching Hospital, Department of Community Medicine

Bello, Shaibu; Usmanu Danfodiyo University, Department of Medical Education
Asuzu, Michael; University College Hospital, Department of Community Medicine
Mba, Nwando; Nigeria Centre for Disease Control
Oladejo, John; Nigeria Centre for Disease Control
Ilori, Elsie; Nigeria Centre for Disease Control
Alfvén, Tobias ; Karolinska Institutet, Department of Global Public Health
Igumbor, Ehimario; Nigeria Centre for Disease Control; University of the Western Cape, School of Public Health
Ihekweazu, Chikwe; Nigeria Centre for Disease Control

Keywords:	COVID-19, Public health < INFECTIOUS DISEASES, Epidemiology < TROPICAL MEDICINE

SCHOLARONE™
Manuscripts

I, the Submitting Author has the right to grant and does grant on behalf of all authors of the Work (as defined in the below author licence), an exclusive licence and/or a non-exclusive licence for contributions from authors who are: i) UK Crown employees; ii) where BMJ has agreed a CC-BY licence shall apply, and/or iii) in accordance with the terms applicable for US Federal Government officers or employees acting as part of their official duties; on a worldwide, perpetual, irrevocable, royalty-free basis to BMJ Publishing Group Ltd ("BMJ") its licensees and where the relevant Journal is co-owned by BMJ to the co-owners of the Journal, to publish the Work in this journal and any other BMJ products and to exploit all rights, as set out in our licence.

The Submitting Author accepts and understands that any supply made under these terms is made by BMJ to the Submitting Author unless you are acting as an employee on behalf of your employer or a postgraduate student of an affiliated institution which is paying any applicable article publishing charge ("APC") for Open Access articles. Where the Submitting Author wishes to make the Work available on an Open Access basis (and intends to pay the relevant APC), the terms of reuse of such Open Access shall be governed by a Creative Commons licence – details of these licences and which Creative Commons licence will apply to this Work are set out in our licence referred to above.

Other than as permitted in any relevant BMJ Author's Self Archiving Policies, I confirm this Work has not been accepted for publication elsewhere, is not being considered for publication elsewhere and does not duplicate material already published. I confirm all authors consent to publication of this Work and authorise the granting of this licence.

For peer review only

Symptom scores have poor predictive capacity for COVID-19 positivity: national derivation and validation cohort study in Nigeria

Kelly Elimian[1,2,3,4], Olaolu Aderinola[1], Jack Gibson[5], Puja Myles[5], Chinwe Ochu[1‡], Carina King[2], Tochi Okwor[1], Giulia Gaudenzi[2], Adebola Olayinka[6], Habib Zaiyad[7], Cornelius Ohonsi[1], Blessing Ebhodaghe[1], Oladipupo Ipadeola[8], Chioma Dan-Nwafor[1], William Nwachukwu[1], Ismail Abdus-Salam[9], Oluwatosin Akande[10], Olanrewaju Falodun[11], Chinedu Arinze[1], Chidiebere Ezeokafor[12], Jafiya Abubakar[1], Anastacia Ojimba[13], John Aremu[14], Emmanuel Joseph[15], Abimbola Bowale[16], Bamidele Mutiu[9], Babatunde Akeem Saka[9], Arisekola Jinadu[1], Khadeejah Hamza[17], Christian Ibeh[18], Shaibu Oricha Bello[19], Michael Asuzu[20], Nwando Mba[1], John Oladejo[1], Elsie Ilori[1], Tobias Alfven[2], Ehimario Igumbor[1], Chikwe Ihekweazu[1]

[1]Nigeria Centre for Disease Control, Abuja, Nigeria
[2]Department of Global Public Health, Karolinska Institutet, Stockholm, Sweden
[3]Department of Microbiology, University of Benin, Edo State, Nigeria
[4]Nigeria COVID-19 Research Consortium, Abuja, Nigeria
[5]Department of Epidemiology and Public Health, University of Nottingham, UK
[6]World Health Organization, Abuja, Nigeria
[7]University of Abuja Teaching Hospital, Nigeria
[8]University of Ilorin, Kwara State, Nigeria
[9]Ministry of Health, Lagos State, Nigeria
[10]University of Ilorin Teaching Hospital, Kwara State, Nigeria
[11]Department of Internal Medicine, National Hospital, Abuja, Nigeria
[12]National Agency for the Control of AIDS, Abuja, Nigeria
[13]Federal Medical Centre, Asaba, Delta State, Nigeria
[14]Federal Teaching Hospital Gombe, Gombe State, Nigeria
[15]Kaduna State Infectious Disease Control Center, Community Medicine, Kaduna State, Nigeria
[16]Mainland Hospital Yaba, Lagos State, Nigeria
[17]Department of Community Medicine, Ahmadu Bello University, Kaduna State, Nigeria
[18]Department of Community Medicine, Nnamdi Azikiwe University Teaching Hospital, Nigeria
[19]College of Health Sciences, Usmanu Danfodiyo University, Sokoto State, Nigeria
[20]Department of Community Medicine, University College Hospital, Oyo State, Nigeria.

‡Corresponding author and email address
Dr Kelly Elimian
kellyelimian@gmail.com

For peer review only - http://bmjopen.bmj.com/site/about/guidelines.xhtml

Abstract

Objectives Suboptimal molecular testing capacity for COVID-19 diagnosis underlines the importance of alternative diagnostic approaches, such as symptom-based predictive scores. Therefore, this study aimed to develop and validate a symptom prediction tool for COVID-19 test positivity in Nigeria.

Design Predictive modelling study.

Participants A cohort of 43,221 individuals within the national COVID-19 surveillance dataset collected from February 27 to August 27, 2020.

Methods The dataset was randomly split into two equal halves: derivation and validation datasets. Using derivation dataset (n=21,477), backward multivariable logistic regression approach was used to identify symptoms positively associated with COVID-19 positivity (by RT-PCR) in children (≤17 years), adults (18-64 years) and elderly (≥65 years) patients separately. Weighted statistical and clinical scores were developed using beta regression coefficients and clinicians' judgements, respectively. Using the validation dataset (n=21,744), the predictive capacity of individual symptoms, un-weighted score and the two weighted scores was assessed using Area Under the Receiver Operating Characteristic curve (AUROC) values.

Results

Overall, 27.6% of children (4,415/15,988), 34.6% of adults (9,154/26,441), and 40.0% of elderly (317/792) that had been tested were positive for COVID-19. Best individual symptom predictor of COVID-19 positivity was loss of smell in children (AUROC 0.56, 95% CI: 0.55-0.56), either fever or cough in adults (AUROC 0.57, 95% CI: 0.56-0.58), and difficulty in breathing in the elderly (AUROC 0.53, 0.48-0.58) patients. In children, adults, and the elderly patients all scoring approaches showed similar predictive performance.

Conclusion

The predictive capacity of various symptom scores for COVID-19 was poor overall, emphasising the need to continue the capacity strengthening molecular testing for COVID-19 in Nigeria.

Keywords

Diagnosis; Symptoms; COVID-19; Predictive; Nigeria

For peer review only - http://bmjopen.bmj.com/site/about/guidelines.xhtml

Strengths and limitations of this study

- This study appears to be the first to have developed and validated symptom prediction scores for COVID-19 positivity using nationally-representative data from Nigeria.
- The study adopted a participatory approach in the derivation of clinically weighted score by engaging clinicians managing COVID-19 patients, which can enhance the relevance of the findings in the medical community.
- Splitting the dataset for both derivation and validation of symptom scores in the present study might have underestimated prediction performance due to loss of power
- The study lacked detailed clinical laboratory data (e.g. albumin or albumin/globin, direct bilirubin values) that have been found to be significant variables in COVID-19 diagnostic models.

Introduction

The index case of COVID-19 in Nigeria was recorded on February 27, 2020, in Ogun State; one of the contacts of the index case was later diagnosed with the disease in the same state. As of epidemiological week 44 (October 26–November 1), Nigeria has recorded 62,964 confirmed cases, 1,146 deaths and 58,790 recoveries, resulting in a case fatality rate of 1.8%.[1] The rapid increase in COVID-19 cases in Nigeria caused a strain on the healthcare systems, including in the laboratory sector, particularly in the area of molecular testing using real-time polymerase chain reaction (RT-PCR). Deficiencies in molecular laboratory capacity hindered the rapid identification of persons with COVID-19 infection and resultant initiation of appropriate treatment and public health measures.[2] As of April 9, 2020, about 5,000 tests for Severe Acute Respiratory Syndrome Coronavirus 2 (SARS-CoV-2) had been performed across Nigeria by eight laboratories across six States.[3] Concerted efforts to expand molecular testing capacity across the country have since been made, with 673,183 tests for SARS-CoV-2 and expansion of the NCDC COVID-19 laboratory networks to 69 functioning laboratories as of November 6, 2020.[4] With a population of over 200 million and community transmission of COVID-19, this testing rate for Nigeria is still well below the Africa CDC-set target of 1% for population. However, laboratory capacity to test for SARS-CoV-2 remains a challenge in many sub-Saharan African countries, and it is worth noting that this was apparent especially in the early phase of COVID-19 pandemic.[5]

For peer review only - http://bmjopen.bmj.com/site/about/guidelines.xhtml

Although PCR testing is considered the gold-standard for COVID-19 diagnosis, limited access and centralised laboratory systems often contribute to delay.[6] This has contributed to making diagnostic results unavailable for urgent decision-making by frontline healthcare workers, with consequent effect on healthcare delivery and increased risk of nosocomial infection.[7] Between February 27 and June 6, 2020, for example, the median turn-around time for laboratory diagnosis of COVID-19 in Nigeria was 2 (IQR: 1-4) days, and 9% of the 12,289 confirmed COVID-19 cases during the same period was recorded among healthcare workers.[8] Thus, to mitigate the impact of COVID-19 on the healthcare system, while also providing the best possible care for patients, rapid and accurate diagnosis are crucial.[9] The Nigeria Centre for Disease Control (NCDC) and its partners are currently coordinating a national study to validate rapid diagnostic test kits to determine their accuracy and clinical utility in Nigeria.

One approach to supplement testing is the use of diagnostic prediction models that utilise clinical variables (including standard blood counts) to estimate the likelihood of individuals being COVID-19 positive (or experiencing a poor clinical outcome from infection).[9] A systematic review of developed and/or validated prediction models found the most frequently reported predictors of COVID-19 diagnosis to be flu-like symptoms (e.g. chills, fatigue) and neutrophil count, with c-statistic estimates ≥0.90 (an excellent discriminative performance).[9] However, to date, these prediction models have been judged to be at a high risk of bias, largely because they were fitted on data that were not representative of the target population, and have suffered from poor reporting on intended use of the models or their calibration performance. While the reported performances of models to date were encouraging albeit optimistic, their adoption in clinical practice have not been recommended.[9] A key limitation has been the lack of models or included data from sub-Saharan Africa. The majority of these models use data from China and European countries, thus limiting their generalisability to settings such as sub-Sahara Africa with different COVID-19 epidemiology, health care systems, living conditions, and health-seeking behaviours.

The majority of confirmed COVID-19 cases in Nigeria are asymptomatic at the point of diagnosis [8]; for those confirmed cases who present with symptoms, the most common symptoms have been non-specific (e.g. fever, cough and difficulty in breathing).[8,10,11] Using data from February 27 to June 6, 2020, presenting with cough, fever, loss of smell and loss of taste was found to be positively and independently associated with COVID-19 positivity in Nigeria[12]. Given the dearth of evidence regarding the predictive capacity of clinical symptoms with regards to COVID-19 positivity in Nigeria and indeed in sub-Saharan African context,

For peer review only - http://bmjopen.bmj.com/site/about/guidelines.xhtml

this study aims to develop and validate the predictive capacity of clinical signs and symptoms with regards testing positive for COVID-19 and to investigate whether there were any gains in the predictive capacity of statistically- and clinically-derived weighted combined symptom scores as compared to an un-weighted combined score in Nigeria.

Methods

Study design and data source

This is a predictive modelling study using a retrospective cohort of persons enrolled in the Surveillance, Outbreak Response Management and Analysis System (SORMAS) database from February 27 to August 27, 2020. A detailed description of SORMAS in the COVID-19 context in Nigeria is available elsewhere.[8] Briefly, SORMAS is an open-source real-time electronic health surveillance and laboratory database, which has been in use in Nigeria since 2017. SORMAS is hosted and coordinated at the NCDC's headquarter in Abuja.

Study participants

The study population comprised children (≤17 years), adults (18-64 years) and the elderly (≥65 years) who were tested for SARS-CoV-2 infection. Eligibility for RT-PCR test during the study period was based on the NCDC COVID-19 standard case definition for suspected cases (Supplementary Table 1).[13] However, provisions were made to test persons concerned about COVID-19 infection upon presentation to designated testing centres regardless of whether they met the NCDC case definitions or not.

Data collection and management

Sociodemographic (e.g. age, sex, occupation, education and geopolitical zone of residence) and clinical presentation (signs and symptoms in the 14 days prior to testing) were collected from all persons tested. The collection and transportation of respiratory samples (oropharyngeal and nasopharyngeal swabs) for laboratory analysis were facilitated by trained healthcare workers according to NCDC guidelines.[14] Testing for SARS-CoV-2 by RT-PCR was performed according with the WHO guidelines.[15] All the collected data across the country, including laboratory diagnostic outcomes, were submitted by trained healthcare workers to NCDC via the SORMAS application installed on tablets or laptops. Patients who tested positive for COVID-19 were managed either in a government health facility or at home, depending on the illness severity or availability of bed space at health facilities, while observing the NCDC case management guidelines.[16]

For peer review only - http://bmjopen.bmj.com/site/about/guidelines.xhtml

Given that this was an analysis of a secondary dataset, there was no formal sample size calculation for this study; however, the study met the standard sample size requirement of 10 outcome events per degree of freedom in prediction models (e.g., 10 binary variables in the model require 100 COVID-19 positive cases). We used the split-sample method to randomly split the dataset into two, assigning half of the records to either the derivation or external validation datasets. However, we made four major assumptions to define the study eligibility criteria. First, we assumed that a missing variable was indicative of absence (i.e., record not present), supported by findings ($p<0.001$) from a chi-square test of association between two *ad hoc* variables 'any-missing' and 'any-absent'. Second, we decided that eligible study participants needed to have complete records for both age and sex since these are crucial demographic variables. Third, given the high proportion of asymptomatic COVID-19 cases in Nigeria,[8] the study participants needed to have had at least one symptom positively recorded (i.e. symptomatic). Lastly, analyses were performed separately for children, adults and the elderly, given the evidence supporting the age-dependence of COVID-19 symptom presentation, with children more likely to be asymptomatic than adults.[17,18] Figure 1 shows a flowchart showing the processes for selecting the study population as stated in the eligibility criteria (definitions of suspected and probable cases are available in the NCDC case definition [13]). All symptoms were coded as '1' if present and '0' if not. Sex was classified as a binary variable, with '1' and '0' indicative of male and female gender, respectively.

Figure 1 about here

Outcome and predictor variables

The outcome was COVID-19 positivity, defined as either presence or absence of SARS-CoV-2 by RT-PCR confirmation (yes/no). Clinical prediction variables were informed by evidence from our previous study using the same dataset, though it covered a shorter period (February 27 to June 5, 2020).[12] The clinical variables were collected using a combination of self-reports by COVID-19 suspected cases and/or their caretakers as well as by objective assessment by a healthcare worker. The three categories of clinical signs and symptoms (each coded yes/no) with their respective examples include: 1. "obvious or as observed by a healthcare worker" (chills/sweat, cough, breathing difficulty, rapid breathing and runny nose); 2. "elicitable or information volunteered or given upon inquiry by patients/relatives" (abdominal pain/diarrhoea, gastrointestinal tract symptoms, chest pain, fatigue/weakness, headache,

For peer review only - http://bmjopen.bmj.com/site/about/guidelines.xhtml

musculoskeletal pain, sore throat, loss of taste, and loss of smell); 3. and "measurable as assessed by a healthcare worker" (fever, defined as an axillary temperature ≥37.5°C).

Statistical analyses

The data were set up and analysed using Stata 13.0 (Stata Corporation, College Station, TX, USA).

Model development

Using the derivation dataset, associations between individual symptoms and COVID-19 positivity was investigated, in turn, using univariable logistic regression. Analyses were stratified by children, adults, and the elderly. The results are presented as unadjusted odds ratios (ORs) and 95% confidence intervals (95% CI). To determine which of the symptoms were independently associated with COVID-19 positivity, we first conducted a test for multicollinearity by the various age groups (children, adults, and the elderly) using the variance inflation factor (VIF) and tolerance values of each statistically significant symptom identified from the univariable logistic regression model (see Supplementary Table 2a, 2b and 2c for results). This was to ensure that symptoms were independent of each other or not collinear and therefore suitable for combining into a single score. Both VIF and tolerance values measure how much the regression coefficient for a symptom is determined by the other symptoms in the model; low VIF and high tolerance values suggest the absence of multicollinearity and vice-versa.[19] Thereafter we used the backward multivariable logistic regression approach to select all the statistically significant symptoms for the model. As before, analyses were conducted for children, adults, and the elderly separately.

We then created three different symptom scores. First, an un-weighted combined score was calculated for each person (separately for children, adults, and the elderly) by allocating '1' point for each symptom before summation. The un-weighted combined scores were included in the model as a continuous variable. The combined scores were further recoded as binary categorical variables to represent clinical scores≥1 (yes/no), ≥2 (yes/no), ≥3 (yes/no), and ≥4 (yes/no). Additionally, weighted combined scores were developed using two approaches. First, we used the beta regression coefficients obtained from the multivariable logistic regression model that was run previously. Each symptom was given the value of '1' if present and '0' if otherwise. Each symptom was then multiplied by its weight, obtained by multiplying the respective beta regression coefficients (or log of the ORs) by 10. Essentially, the beta regression coefficients measure the relative prognostic strength of each symptom when they

For peer review only - http://bmjopen.bmj.com/site/about/guidelines.xhtml

are included simultaneously in a multivariable regression model so that the bigger the value of a symptom, the more its weight. Second, we engaged six clinicians (including infectious disease consultants) who have been managing COVID-19 patients in treatment centres and at home in the FCT, Gombe, Delta, and Kaduna. Here, the clinicians were provided with a list of symptoms identified to be statistically significant from the univariable logistic models, with the freedom to add any missing sign or symptom in the model as needed. Each clinician was then asked to independently assign a weight of 1-5 for each symptom based on experiences from managing COVID-19 patients (see Supplementary Table 3 for detail of weighted scores assigned by each clinician for the identified symptoms). Similar to the statistical weighting approach, each symptom was multiplied by the average of the combined weights assigned by all the clinicians. Predictive capacity of the various score thresholds was also compared to see which combination of symptoms was more predictive of COVID-19 positivity.

Model validation

The different models were then applied to the validation dataset. Separately for children, adults and the elderly, predictive capacity was assessed in terms of sensitivity, specificity, Area under the Receiver Operating Characteristic (ROC) curve (AUROC) value, positive predictive value (PPV) and negative predictive value (NPV);[20] and ROC curves were plotted for the comparison of clinical prediction scores.

Hosmer-Lemeshow or the goodness-of-fit test

The Hosmer-Lemeshow or the goodness-of-fit test was used to test the calibration i.e. the proximity of observed and expected values, with a significant value ($p<0.05$), indicating that the model was not well calibrated.

Where applicable, this study is reported according to the TRIPOD (Transparent Reporting of a multivariable prediction model for Individual Prognosis Or Diagnosis) statement.[21]

Ethics and ethical considerations

The Nigeria National Health Research Ethics Committee approved the use of the SORMAS database for this study (reference number: NHREC/01/01/2007-22/06/2020).

Patient and public involvement

Being an analysis of de-identified secondary dataset, it was not possible to involve patients or the public in the design, or conduct, or reporting, or dissemination plans of this study. However, clinicians who have been managing COVID-19 patients both in health facilities and at home

For peer review only - http://bmjopen.bmj.com/site/about/guidelines.xhtml

in Nigeria were actively engaged both in planning the study and in developing the clinical scores.

Results

Description of study population

The baseline sociodemographic and clinical characteristics of the study participants in the combined datasets are presented in Table 1. Overall, 15,988 children met the study eligibility criteria, 4,415 (27.6%) of whom tested positive for COVID-19. Of the 15,988 children, 62.2% were male. A total of 26,441 adults met the study eligibility criteria, 9,154 (34.6%) of whom tested positive for COVID-19. Sixty-four percent (16,819/26,441) of adults were male, and 11.3% (2,977/26,441) were healthcare workers. Seven hundred and ninety-two elderly patients met the study eligibility criteria, 317 (40.0%) of whom tested positive for COVID-19 and 62.3% were males. Similar characteristics were observed in the derivation (Supplementary Table 4) and validation (Supplementary Table 5) cohorts for all age-groups.

For peer review only

Table 1: Baseline sociodemographic and clinical characteristics of all the study participants in relation to COVID-19 infection (combined dataset)

Variable	Children (≤17 years)		Adults (18-64 years)		Elderly (≥65 years)	
	PCR-confirmed cases [n=4,415 (%)]	PCR positive and negative cases [N=15,988 (%)]	PCR-confirmed cases [n=9,154 (%)]	PCR positive and negative cases [N=26,441 (%)]	PCR-confirmed cases [n=317 (%)]	PCR positive and negative cases [N=792 (%)]
**Socio-demographic features**						
**Sex**						
Female	1,773 (40.16)	6,051 (37.85)	3,080 (33.65)	9,622 (36.39)	110 (34.70)	299 (37.75)
Male	2,642 (59.84)	9,937 (62.15)‡	6,074 (66.35)	16,819 (63.61)‡	207 (65.30)	493 (62.25)NS
**Geopolitical zone§**						
South-west	1,302 (29.49)	3,539 (22.14)	2,592 (28.32)	6,245 (23.62)	86 (27.13)	185 (23.36)
South-south	1,435 (32.50)	5,689 (35.58)	3,228 (35.26)	10,911 (41.27)	108 (34.07)	340 (42.93)
South-east	90 (2.04)	278 (1.74)	273 (2.98)	609 (2.30)	25 (7.89)	37 (4.67)
North-central	816 (18.48)	3,056 (19.11)	1,526 (16.67)	4,061 (15.36)	30 (9.46)	76 (9.60)
North-west	658 (14.90)	3,118 (19.50)	1,258 (13.72)	4,005 (15.15)	45 (14.20)	116 (14.65)
North-east	114 (2.58)	308 (1.93)‡	277 (3.03)	610 (2.31)‡	23 (7.26)	38 (4.80)‡
**Setting**						
Rural	230 (5.21)	887 (5.55)	511 (5.58)	1,562 (5.91)	35 (11.04)	74 (9.34)
Urban	2,048 (46.39)	6,943 (43.43)	4,487 (49.02)	13,088 (49.50)	183 (57.73)	423 (53.41)
Missing	2,137 (48.40)	8,158 (51.03)‡	4,156 (45.40)	11,791 (44.59)NS	99 (31.23)	295 (37.25)†
**Education**						
None	58 (1.31)	217 (1.36)	128 (1.40)	368 (1.39)	25 (7.89)	48 (6.06)
Nursery	30 (0.68)	143 (0.89)	3 (0.03)	7 (0.03)	0 (0.00)	0 (0.00)
Primary	130 (2.94)	520 (3.25)	102 (1.11)	322 (1.22)	8 (2.52)	18 (2.27)
Secondary	385 (8.72)	1,379 (8.63)	792 (8.65)	2,262 (8.55)	23 (7.26)	58 (7.32)
Tertiary	1,410 (31.94)	4,066 (25.43)	3,141 (34.31)	8,145 (30.80)	87 (27.44)	168 (21.21)
Other	179 (4.05)	1,065 (6.66)	261 (2.85)	688 (2.60)	20 (6.31)	45 (5.68)
Missing	2,223 (50.35)	8,598 (53.78)‡	4,727 (51.64)	14,649 (55.40)‡	154 (48.58)	455 (57.45)†
**Occupation**						
Student/pupil	599 (13.57)	2,809 (17.57)	434 (4.74)	1,516 (5.73)	1 (0.32)	2 (0.25)
Child/housewife	133 (3.01)	489 (3.06)	178 (1.94)	500 (1.89)	13 (4.10)	42 (5.30)
Business/trading	217 (4.92)	765 (4.78)	757 (8.27)	2,043 (7.73)	19 (5.99)	47 (5.93)
Transporter	11 (0.25)	80 (0.50)	45 (0.49)	174 (0.66)	0 (0.00)	3 (0.38)
Healthcare worker	601 (13.61)	1,953 (12.22)	973 (10.63)	2,977 (11.26)	7 (2.21)	20 (2.53)
Laboratorian	15 (0.34)	40 (0.25)	26 (0.28)	63 (0.24)	0 (0.00)	0 (0.00)
Farmer	36 (0.82)	192 (1.20)	173 (1.89)	589 (2.23)	24 (7.57)	47 (5.93)
Animal-related worker	8 (0.18)	28 (0.18)	25 (0.27)	80 (0.30)	1 (0.32)	9 (1.14)
Religious/traditional leader	5 (0.11)	24 (0.15)	53 (0.58)	129 (0.49)	6 (1.89)	9 (1.14)

Other	1,633 (36.99)	5,126 (32.06)	4,072 (44.48)	11,408 (43.15)	164 (51.74)	375 (47.35)
Missing	1,157 (26.21)	4,482 (28.03)‡	2,418 (26.41)	6,962 (26.33)‡	82 (25.87)	238 (30.05)NS
**Clinical signs and symptoms**						
**Clinical outcome**						
Recovered	2,847 (64.48)	4,443 (27.79)	5,694 (62.20)	7,313 (27.66)	150 (47.32)	184 (23.23)
Dead	36 (0.82)	51 (0.32)	485 (5.30)	549 (2.08)	83 (26.18)	84 (10.61)
No outcome yet	1,532 (34.70)	11,494 (71.89)‡	2,975 (32.50)	18,579 (70.27)‡	84 (26.50)	524 (66.16)‡
Obvious (visible to healthcare workers on sight)						
**Chills/sweat**						
No	4,355 (98.64)	15,764 (98.60)	9,009 (98.42)	26,029 (98.44)	315 (99.37)	783 (98.86)
Yes	60 (1.36)	224 (1.40)NS	145 (1.58)	412 (1.56)NS	2 (0.63)	9 (1.14)NS
**Cough**						
No	2,345 (53.11)	9,393 (58.75)	4,130 (45.12)	14,391 (54.43)	114 (35.96)	321 (40.53)
Yes	2,070 (46.89)	6,595 (41.25)‡	5,024 (54.88)	12,050 (45.57)‡	203 (64.04)	471 (59.47)†
**Breathing difficulty**						
No	3,936 (89.15)	14,283 (89.34)	7,549 (82.47)	22,425 (84.81)	200 (63.09)	541 (68.31)
Yes	479 (10.85)	1,705 (10.66)NS	1,605 (17.53)	4,016 (15.19)‡	117 (36.91)	251 (31.69)†
**Rapid breathing**						
No	4,345 (98.41)	15,783 (98.72)	9,002 (98.34)	26,112 (98.76)	307 (96.85)	771 (97.35)
Yes	70 (1.59)	205 (1.28)†	152 (1.66)	329 (1.24)‡	10 (3.15)	21 (2.65)NS
**Runny nose**						
No	3,057 (69.24)	11,835 (74.02)	6,792 (74.20)	20,496 (77.52)	259 (81.70)	658 (83.08)
Yes	1,358 (30.76)	4,153 (25.98)‡	2,362 (25.80)	5,945 (22.48)‡	58 (18.30)	134 (16.92)NS
Elicitable (can be found out by asking questions of patients/relatives)						
**Abdominal pain/diarrhoea**						
No	4,142 (93.82)	14,939 (93.44)	8,464 (92.46)	24,580 (92.96)	297 (93.69)	728 (91.92)
Yes	273 (6.18)	1,049 (6.56)NS	690 (7.54)	1,861 (7.04)NS	20 (6.31)	64 (8.08)NS
**GIT symptoms**						
No	3,972 (89.97)	14,375 (89.91)	8,100 (88.49)	23,766 (89.88)	282 (88.96)	705 (89.02)
Yes	443 (10.03)	1,613 (10.09)NS	1,054 (11.51)	2,675 (10.12)‡	35 (11.04)	87 (10.98)NS
**Chest pain**						
No	4,286 (97.08)	15,577 (97.43)	8,782 (95.94)	25,621 (96.90)	303 (95.58)	766 (96.72)
Yes	129 (2.92)	411 (2.57)NS	372 (4.06)	820 (3.10)‡	14 (4.42)	26 (3.28)NS
**Fatigue**						
No	4,143 (93.84)	15,229 (95.25)	8,583 (93.76)	25,014 (94.60)	292 (92.11)	750 (94.70)
Yes	272 (6.16)	759 (4.75)‡	571 (6.24)	1,427 (5.40)‡	25 (7.89)	42 (5.30)†
**Headache**						
No	3,753 (85.01)	13,712 (85.76)	7,849 (85.74)	23,283 (88.06)	287 (90.54)	738 (93.18)

For peer review only - http://bmjopen.bmj.com/site/about/guidelines.xhtml

Yes	662 (14.99)	2,276 (14.24)NS	1,305 (14.26)	3,158 (11.94)‡	30 (9.46)	54 (6.82)†
**Musculoskeletal pain**						
No	4,299 (97.37)	15,595 (97.54)	8,901 (97.24)	25,783 (97.51)	306 (96.53)	771 (97.35)
Yes	116 (2.63)	393 (2.46)NS	253 (2.76)	658 (2.49)†	11 (3.47)	21 (2.65)NS
**Sore throat**						
No	3,306 (74.88)	11,968 (74.86)	6,968 (76.12)	19,583 (74.06)	259 (81.70)	649 (81.94)
Yes	1,109 (25.12)	4,020 (25.14)NS	2,186 (23.88)	6,858 (25.94)‡	58 (18.30)	143 (18.06)NS
**Loss of taste**						
No	3,925 (88.90)	15,283 (95.59)	8,301 (90.68)	25,268 (95.56)	303 (95.58)	770 (97.22)
Yes	490 (11.10)	705 (4.41)‡	853 (9.32)	1,173 (4.44)‡	14 (4.42)	22 (2.78)†
**Loss of smell**						
No	3,815 (86.41)	15,160 (94.82)	8,186 (89.43)	25,198 (95.30)	307 (96.85)	778 (98.23)
Yes	600 (13.59)	828 (5.18)‡	968 (10.57)	1,243 (4.70)‡	10 (3.15)	14 (1.77)†
Measureable						
**Fever**						
No	2,350 (53.23)	9,430 (58.98)	4,555 (49.76)	15,434 (58.37)	163 (51.42)	453 (57.20)
Yes	2,065 (46.77)	6,558 (41.02)‡	4,599 (50.24)	11,007 (41.63)‡	154 (48.58)	339 (42.80)†

†=p-value<0.05; ‡=p<0.001; NS=p>0.05 or Not significant
GIT= gastrointestinal (nausea + vomiting)
Musculoskeletal pain= muscle /joint pain
§=State composition of geopolitical zones in Nigeria: South-West (Ekiti, Lagos, Ogun, Ondo, Osun and Oyo); South-South (Akwa-Ibom, Bayelsa, Cross-River, Rivers, Delta and Edo); South-East (Abia, Anambra, Ebonyi, Enugu and Imo); North-Central (Benue, Kogi, Kwara, Nasarawa, Niger, and Plateau States as well as the FCT); North-West (Jigawa, Kaduna, Kano, Katsina, Kebbi, Sokoto and Zamfara); and North-East (Adamawa, Bauchi, Borno, Gombe, Taraba and Yobe).

For peer review only - http://bmjopen.bmj.com/site/about/guidelines.xhtml

Performance of individual symptoms for predicting COVID-19 positivity

In the unadjusted model, presentation with cough, runny nose, fatigue, loss of taste, loss of smell, and fever was significantly associated with higher odds of COVID-19 positivity in children (Table 2). In the adjusted model, however, presentation with the following symptoms remained significantly ($p<0.001$) associated with higher odds of COVID-19 positivity: cough (adjusted OR 1.32, 95% CI: 1.19-1.47), runny nose (aOR 1.48, 95% CI: 1.32-1.66), fatigue (aOR 1.53, 95% CI: 1.21-1.93), loss of taste (aOR 2.26, 95% CI: 1.63-3.12), loss of smell (aOR 4.87, 95% CI: 3.66-6.49), or fever (aOR 1.47, 95% CI: 1.33-1.63). Regarding predictive performance, loss of smell recorded the highest AUROC value (0.56, 95% CI: 0.55-0.56). Specifically, cough recorded the highest sensitivity (47.7%, 95% CI: 45.6-49.7%) and NPV (74.7%, 95% CI: 73.4-75.9%), while loss of smell recorded the highest specificity (98.1%, 95% CI: 97.7-98.4%) and PPV (72.9%, 95% CI: 68.3-77.2%) (see details in Supplementary Table 6).

For peer review only

Table 2: Associations and predictive performance of individual clinical characteristics in relation to COVID-19 positivity in children

Variable	Derivation dataset (n=7,911)				
	**Unadjusted OR (95% CI)**	**Wald's P-value**	**Adjusted OR (95% CI)‡**	**Wald's P-value**	**AUROC (95% CI)§**
**Clinical signs and symptoms**					
Obvious (visible to healthcare workers on sight)					
**Chills/sweat**					
No	1.00	0.494			
Yes	1.15 (0.77-1.70)				
**Cough**					
No	1.00	<0.001	1.00	<0.001	0.54
Yes	1.39 (1.26-1.53)		**1.32 (1.19-1.47)**		(0.53-0.55)
**Breathing difficulty**					
No	1.00	0.792			
Yes	1.02 (0.87-1.19)				
**Rapid breathing**					
No	1.00	0.617			
Yes	1.11 (0.73-1.68)				
**Runny nose**					
No	1.00	<0.001	1.00	<0.001	0.52
Yes	1.53 (1.37-1.70)		**1.48 (1.32-1.66)**		(0.51-0.53)
Elicitable (can be found out from patients' responses or by asking questions of relatives)					
**Abdominal pain/diarrhoea**					
No	1.00	0.190			
Yes	0.87 (0.71-1.07)				
**GIT symptoms**					
No	1.00	0.664			
Yes	0.96 (0.82-1.14)				
**Chest pain**					
No	1.00	0.065			
Yes	1.33 (0.98-1.79)				
**Fatigue/weakness**					
No	1.00	0.025	1.00	<0.001	0.51
Yes	1.29 (1.03-1.62)		**1.53 (1.21-1.93)**		(0.51-0.52)
**Headache**					
No	1.00	0.106			
Yes	1.12 (0.98-1.29)				
**Musculoskeletal pain**					
No	1.00	0.602			
Yes	1.09 (0.80-1.48)				
**Sore throat**					
No	1.00	0.734			
Yes	1.02 (0.91-1.14)				
**Loss of taste**					
No	1.00	<0.001	1.00	<0.001	0.54
Yes	7.07 (5.59-8.95)		**2.26 (1.63-3.12)**		(0.54-0.55)
**Loss of smell**					
No	1.00	<0.001	1.00	<0.001	**0.56**
Yes	7.85 (6.30-9.77)		**4.87 (3.66-6.49)**		**(0.55-0.56)**
Measureable					
**Fever**					
No	1.00	<0.001	1.00	<0.001	0.54
Yes	1.43 (1.29-1.58)		**1.47 (1.33-1.63)**		(0.52-0.55)

OR: Odds ratio; CI: Confidence Interval; significant results in the adjusted model are in bold fonts.
‡: The fully-adjusted model includes all statistically significant variables from the unadjusted model.
§: Area under receiver operating characteristic curve; figures are for variable that were statistically significant in the adjusted model only; the best predictive AUROC value is highlighted in bold.

311

The presentation of symptoms significantly associated with increased COVID-19 positivity was higher in adults than in children (Table 3). In the adjusted model, however, it was only the presentation with cough (aOR 1.77, 95% CI: 1.64-1.91), runny nose (aOR 1.18, 95% CI: 1.08-1.30), chest pain (aOR 1.50, 95% CI: 1.21-1.85), fatigue (aOR 1.39, 95% CI: 1.17-1.64), headache (aOR 1.24, 95% CI: 1.11-1.39), loss of taste (aOR 2.33, 95% CI: 1.83-2.96), loss of smell (aOR 4.18, 95% CI: 3.30-5.28), or fever (aOR 1.58, 95% CI: 1.47-1.71) that remained independently associated with COVID-19 positivity. Regarding predictive performance, presentation with either fever or cough appeared to be the best predictor of COVID-19 positivity (AUROC 0.57, 95% CI: 0.56-0.58). Additionally, presenting with cough or fever recorded the highest sensitivity at 54.3% (52.9-55.8%) and NPV at 70.8% (69.7-71.8%); however, presenting with loss of smell recorded the highest specificity (98.5%; 95% CI: 98.2-98.8%) and PPV (79.4%, 95% CI: 76.0-82.5%) (see details in Supplementary Table 7).

For peer review only

Table 3: Associations and predictive performance of individual clinical characteristics in relation to COVID-19 positivity in adults

Variable	Derivation dataset (n=13,167)				
	**Unadjusted OR (95% CI)**	**Wald's P-value**	**Adjusted OR (95% CI)‡**	**Wald's P-value**	**AUROC (95% CI)§**
**Clinical signs and symptoms**					
Obvious (visible to healthcare workers on sight)					
**Chills/sweat**					
No	1.00	0.371			
Yes	1.14 (0.85-1.54)				
**Cough**					
No	1.00	<0.001	1.00	<0.001	**0.57**
Yes	1.85 (1.72-1.99)		**1.77 (1.64-1.91)**		**(0.56-0.58)**
**Breathing difficulty**					
No	1.00	<0.001	1.00	0.071	
Yes	1.23 (1.11-1.35)		1.10 (0.99-1.22)		
**Rapid breathing**					
No	1.00	0.004	1.00	0.203	
Yes	1.58 (1.15-2.16)		1.24 (0.89-1.73)		
**Runny nose**					
No	1.00	<0.001	1.00	<0.001	0.53
Yes	1.30 (1.20-1.42)		**1.18 (1.08-1.30)**		(0.52-0.54)
Elicitable (can be found out from patients' responses or by asking questions of relatives)					
**Abdominal pain/diarrhoea**					
No	1.00	0.102			
Yes	1.12 (0.98-1.29)				
**GIT symptoms**					
No	1.00	0.009	1.00	0.092	
Yes	1.17 (1.04-1.32)		1.11 (0.98-1.26)		
**Chest pain**					
No	1.00	<0.001	1.00	<0.001	0.51
Yes	1.75 (1.44-2.14)		**1.50 (1.21-1.85)**		(0.50-0.51)
**Fatigue/weakness**					
No	1.00	0.005	1.00	<0.001	0.51
Yes	1.25 (1.07-1.46)		**1.39 (1.17-1.64)**		(0.50-0.51)
**Headache**					
No	1.00	<0.001	1.00	<0.001	0.52
Yes	1.44 (1.29-1.60)		**1.24 (1.11-1.39)**		(0.51-0.52)
**Musculoskeletal pain**					
No	1.00	0.773			
Yes	1.04 (0.81-1.32)				
**Sore throat**					
No	1.00	<0.001			
Yes	0.83 (0.76-0.90)				
**Loss of taste**					
No	1.00	<0.001	1.00	<0.001	0.54
Yes	5.73 (4.75-6.91)		**2.33 (1.83-2.96)**		(0.53-0.54)
**Loss of smell**					
No	1.00	<0.001	1.00	<0.001	0.55
Yes	6.74 (5.58-8.14)		**4.18 (3.30-5.28)**		(0.54-0.55)
Measureable					
**Fever**					
No	1.00	<0.001	1.00	<0.001	**0.57**
Yes	1.64 (1.52-1.76)		**1.58 (1.47-1.71)**		**(0.56-0.58)**

OR: Odds ratio; CI: Confidence Interval; significant results in the adjusted model are in bold fonts.
‡: The fully-adjusted model includes all statistically significant variables from the unadjusted model.
§: Figures are for variable that were statistically significant in the adjusted model only; and the best predictive AUROC value is highlighted in bold.

Only three symptoms in the elderly patients were significantly associated with COVID-19 positivity both in the unadjusted and adjusted models, with cough (aOR 1.59, 95% CI: 1.04-2.43; p=0.033), difficulty in breathing (aOR 1.74, 95% CI: 1.12-2.72; p=0.015) or loss of smell (aOR 7.15, 95% CI: 1.44-35.44; p=0.016) being independently associated with COVID-19 positivity in the latter model (Table 4). Regarding predictive performance, presenting with difficulty in breathing appeared to be the best predictor of COVID-19 positivity (AUROC 0.53, 0.48-0.58). However, presenting with cough recorded the highest sensitivity (61.4%, 95% CI: 53.3-69.0%) and NPV (62.1%, 95% CI: 54.1-69.6%), while loss of smell recorded the highest specificity (99.1%, 95% CI: 97.0-99.9%) and PPV (60.0%, 95% CI: 14.7-94.7%) (see details in Supplementary Table 8).

Table 4: Associations and predictive performance of individual clinical characteristics in relation to COVID-19 positivity in elderly patients

Variable	Derivation dataset (n=399)				
	Unadjusted OR (95% CI)	Wald's P-value	Adjusted OR (95% CI)‡	Wald's P-value	AUROC (95% CI)§
**Clinical signs and symptoms**					
Obvious (visible to healthcare workers on sight)					
**Chills/sweat**					
No	1.00	0.270			
Yes	0.30 (0.03-2.57)				
**Cough**					
No	1.00	0.025	1.00	0.033	0.52
Yes	1.61 (1.06-2.44)		1.59 (1.04-2.43)		(0.47-0.57)
**Breathing difficulty**					
No	1.00	0.016	1.00	0.015	**0.53**
Yes	1.71 (1.11-2.66)		1.74 (1.12-2.72)		**(0.48-0.58)**
**Rapid breathing**					
No	1.00	0.355			
Yes	2.04 (0.45-9.23)				
**Runny nose**					
No	1.00	0.684			
Yes	1.12 (0.66-1.88)				
Elicitable (can be found out from patients' responses or by asking questions of relatives)					
**Abdominal**					
**pain/diarrhoea**	1.00	0.265			
No	0.62 (0.26-1.44)				
Yes					
**GIT symptoms**					
No	1.00	0.793			
Yes	0.91 (0.45-1.83)				
**Chest pain**					
No	1.00	0.469			
Yes	1.53 (0.48-4.83)				
**Fatigue/weakness**					
No	1.00	0.089			
Yes	2.00 (0.90-4.35)				
**Headache**					
No	1.00	0.303			
Yes	0.67 (0.31-1.44)				

For peer review only - http://bmjopen.bmj.com/site/about/guidelines.xhtml

**Musculoskeletal pain**					
No	1.00	0.301			
Yes	1.80 (0.59-5.45)				
**Sore throat**					
No	1.00	0.439			
Yes	0.82 (0.50-1.35)				
**Loss of taste**					
No	1.00	0.061			
Yes	4.67 (0.93-23.42)				
**Loss of smell**					
No	1.00	0.035	1.00	0.016	0.51
Yes	5.48 (1.12-26.72)		7.15 (1.44-35.44)		(0.49-0.52)
Measureable					
**Fever**					
No	1.00	0.090			
Yes	1.42 (0.95-2.12)				

OR: Odds ratio; CI: Confidence Interval; significant results in the adjusted model are in bold fonts.
‡: The fully-adjusted model includes all statistically significant variables from the unadjusted model.
§: Figures are for variable that were statistically significant in the adjusted model only; and the best predictive AUROC value is highlighted in bold.

Performance of un-weighted, statistically- and clinically-weighted scores for predicting COVID-19 positivity

In adults, the statistically-derived weighted combined score appeared to be slightly better in predicting COVID-19 positivity (AUROC: 0.65), when compared with both the clinically-derived weighted combined and un-weighted scores with AUROC 0.63 each (Table 5). A similar pattern was recorded in children and elderly populations. For individual symptom thresholds, presenting with ≥2 symptoms on the un-weighted score appeared to be a better predictor of COVID-19 positivity (AUROC 0.61, 95% CI: 0.60-0.61) than the other symptom thresholds in adults. For the statistically weighted score, presenting with ≥4 symptoms appeared to be a better predictor of COVID-19 positivity (AUROC 0.59, 95% CI: 0.58-0.59) than the other symptom thresholds in adults. For the clinically-derived weighted score, presenting with either ≥3 or ≥4 symptoms was a better predictor of COVID-19 positivity (AUROC 0.58, 95% CI: 0.58-0.59) in adults. The detailed results including sensitivity, specificity, PPV and NPV values for each age-group are available in Supplementary Tables 9, 10 and 11. Figure 2 shows the ROC curves comparing the predictive performance of unweighted, statistically- and clinically-derived weighted combined scores in children (2a), adult (2b) and the elderly (2c) populations.

For peer review only - http://bmjopen.bmj.com/site/about/guidelines.xhtml

Table 5: Predictive performance of un-weighted, statistically- and clinically-weighted score thresholds for predicting COVID-19 positivity in children, adults and elderly

Outcome	Score	Un-weighted score	Statically-weighted score	Clinically-weighted score
		AUROC value (95% CI)		
**Children (<17 years) n=8,077**				
**Combined score**		0.6064	0.6177	0.5915
**Symptom threshold**	≥1	0.55 (0.54-0.56)	0.55 (0.54-0.56)	0.55 (0.54-0.56)
	≥2	**0.58 (0.57-0.59)**	0.55 (0.54-0.56)	0.54 (0.53-0.55)
	≥3	0.55 (0.54-0.56)	0.55 (0.54-0.57)	0.56 (0.55-0.57)
	≥4	0.52 (0.52-0.53)	**0.59 (0.58-0.60)**	**0.57 (0.56-0.58)**
**Adults (17-64 years) n=13,274**				
**Combined score**		0.6333	0.6475	0.6389
**Symptom threshold**	≥1	0.56 (0.55-0.56)	0.56 (0.55-0.56)	0.56 (0.55-0.56)
	≥2	**0.61 (0.60-0.61)**	0.58 (0.57-0.58)	0.56 (0.55-0.56)
	≥3	0.56 (0.55-0.57)	0.58 (0.57-0.59)	**0.58 (0.58-0.59)**
	≥4	0.53 (0.53-0.54)	**0.59 (0.58-0.59)**	**0.58 (0.58-0.59)**
**Elderly (≥65 years) n=393**				
**Combined score**		0.5413	0.5453	0.5426
**Symptom threshold**	≥1	0.52 (0.48-0.57)	0.52 (0.48-0.57)	0.52 (0.48-0.57)
	≥2	**0.53 (0.49-0.57)**	0.52 (0.48-0.57)	0.52 (0.48-0.57)

The best predictive AUROC values for each age group are highlighted in bold

Hosmer-Lemeshow goodness-of-fit test

The Hosmer-Lemeshow goodness-of-fit tests suggest that only the un-weighted combined score in children and elderly as well as the statistically-derived weighted combined score in adults and the clinically-derived weighted combined score in the elderly appeared to have passed the goodness of fit test with $p>0.05$ (see Supplementary Table 12).

For peer review only - http://bmjopen.bmj.com/site/about/guidelines.xhtml

Discussion

Principal findings

In this study we developed and validated symptom's prediction scores for COVID-19 positivity, independently in children, adults, and the elderly patients in the Nigerian context. The best individual symptom predictors of COVID-19 positivity in children, adult, and the elderly patients were loss of smell (AUROC 0.56, 95% CI: 0.55-0.56), either fever or cough (AUROC 0.57, 95% CI: 0.56-0.58), and difficulty in breathing (AUROC 0.53, 0.48-0.58), respectively. In adults, all the symptom scores showed similar performance, with the statistically weighted score (AUROC: 0.65) slightly showing better performance than the un-weighted (0.63) and clinically-derived weighted (0.64) scores. Similar results were found in children and the elderly patients. Overall, none of the symptom scores had good enough discrimination to use in practice.

Strengths and limitations of this study

To the best of our knowledge, this is the first study to have developed and validated symptom prediction scores with a view to aiding prompt recognition of COVID-19 by frontline healthcare workers in the Nigerian context and possibly in sub-Sahara Africa at large. We also adopted a transparent methodology and adhered to the TRIPOD reporting statement thereby minimising the vagueness often associated with the reporting of studies on the predictive performance of diagnostic models for COVID-19.[9] The methodology taken to the derivation of weighted scores is also a strength of this study. In accordance with the preferred approach for building prediction models,[9] the participatory approach to the derivation of clinically weighted score can enhance the relevance of our findings in the medical community.[22] However, there is need for caution considering the poor discriminatory capacity of the clinical score with a view to preventing the perpetuation of poor clinical practice. The use of beta regression coefficients over odds ratios in deriving the statistical weighted scores has the advantage of being less prone to bias by small to moderate sample size.[23] A common limitation of many COVID-19 diagnostic models is bias due to over-fitting of the models on data that are not representative of the target population.[9] By using SORMAS database (hosts data from all over the country) for both derivation and validation in the present study, our findings are relatively generalisable to the COVID-19 situation in Nigeria and less prone to overestimation of COVID-19 risk among those tested. Nonetheless, this study has some limitations that warrant discussion.

For peer review only - http://bmjopen.bmj.com/site/about/guidelines.xhtml

Being a secondary data analysis based on practical recording of routine clinical assessments, the fundamental assumption is that the data recorded on clinical symptoms are reasonably complete; for instance, we assumed that where a symptom was not recorded as being absent rather than missing. Without any objective means of verifying this assumption, any bias caused by misclassification of the individual symptoms could potentially minimise differences in comparisons, in which case observed differences are likely to be in the direction of the null hypothesis. The approach of splitting the dataset for both derivation and validation of symptom scores may have lowered the precision of estimated effect (wider 95% CIs) [24] and potentially underestimated prediction performance due to loss of power;[25] however, the small number of records in the elderly patients could be due to their difficulty in reporting symptoms.[26] Lastly, the study lacked detailed clinical laboratory data, such as record for albumin or albumin/globin, direct bilirubin values, and red blood cell distribution width, that have been found to be significant variables in COVID-19 diagnostic models [9]. However, the time and technical requirements for testing these laboratory data could limit their clinical utility.

Interpretation & implications of findings

Based on the systematic and critical review of diagnostic models or scores by Wynant *et al*., it seems the performance of a diagnostic model is influenced by its composition, with higher number of clinical and laboratory parameters in a model indicating better predictive performance.[9] For instance, studies in China,[27,28] Brazil,[29] Italy,[30], the Netherlands[31] and France[32] with several clinical and laboratory parameters recorded excellent discriminatory performance, although with substantial evidence of bias[9] and limited utility to frontline healthcare workers. Conversely, the prediction model (containing fewer number of symptoms as well as heart rate, systolic and diastolic blood pressure) developed by Sun *et al*.[33] in Singapore was poor overall (C statistic: 0.65, 0.57 to 0.73). In contrast, evidence suggests that the discriminatory accuracy of a prediction model, including its sensitivity, can be enhanced by limiting the variables to a combination of loss of smell or taste and fever.[34] In the absence of comparison study from another sub-Saharan African country, it is difficult to fully explain the variation in findings here and elsewhere. A follow-up study using both clinical and laboratory parameters in the Nigerian context or at large in sub-Saharan Africa with similar healthcare system and demographic structure, is therefore recommended.

Prediction performance of the un-weighted score with regards COVID-19 positivity appeared to be better in adults than in children and the elderly patients. Despite the poor predictive capacity, this finding has an important implication on the NCDC's current definition of

For peer review only - http://bmjopen.bmj.com/site/about/guidelines.xhtml

COVID-19 suspected cases which emphasises acute respiratory symptoms and either travel history within 14 days prior to symptom onset or self-reported contact with a confirmed case.[13] With our findings indicating age-dependency of symptom scores, it may be worthwhile to review the current case definitions in the Nigerian context. For example, loss of smell and either fever or cough were better in predicting COVID-19 positivity in children and adults, respectively, whereas breathing difficulty seemed more predictive in the elderly patients. Loss of smell recorded the highest specificity with regards to COVID-19 positivity for the three age groups: 98.1% in children, 98.5% in adults and 99.1% in the elderly. However, unlike the present study which explored the predictive capacity of loss of smell and taste separately, a combination of both symptoms have been shown to be more predictive.[35] Thus, the potential use of combined loss of smell and taste to differentiate COVID-19 from other endemic febrile and respiratory illnesses—e.g., malaria, pneumonia—with overlapping symptoms in Nigeria needs be explored further. Presentation with cough or fever was found to have less predictive value in this study, in line with findings from a meta-analysis.[36] Moreover, these endemic diseases in Nigeria are less common in many developed settings, such as Europe, hence the poor predictive performance of our clinical scores is not unexpected.

Clinical validity is an important criterion for assessing a clinical prediction tool.[37] Clinical validity—characterised by sensitivity, specificity and AUROC values—refers to the ability of a prediction tool to distinguish between who has an outcome (in this case SARS-CoV-2 infection) and who does not.[38] The clinical validity of all our prediction scores was generally poor. For the un-weighted and weighted (statistical and clinical) predictive scores in children and adults, presenting with fewer number of symptoms appeared to be more sensitive compared with many symptoms; it was however the other way round with respect to specificity, with ≥4 symptoms recording higher specificity values than fewer symptom thresholds. The poor sensitivity associated with many symptoms could be attributable to a high proportion of false negatives, indicating that some symptoms have limited validity with respect to COVID-19 in children and adults. This finding also underlines the importance of incorporating malaria test as part of the initial evaluation of COVID-19 suspected patients in Nigeria. However, similarity in the predictive performance of various symptom thresholds on the two weighted scores in the elderly suggests that weighting has less predictive value for this population. The high specificity of more symptoms could be indicative of low proportion of false positives, underlining the need to accurately assess symptoms. In practice, there is a trade-off between sensitivity and specificity such that when the consequences of having a false positive test is

For peer review only - http://bmjopen.bmj.com/site/about/guidelines.xhtml

very serious, specificity is prioritised over sensitivity and vice-versa.[39] This is the case for the various symptom thresholds on the un-weighted scale where specificity is higher than sensitivity. A higher specificity over sensitivity for the majority of clinical scores in the current study is of practical relevance when the political implication of refusing to test someone with suspected COVID-19 is considered. However, higher sensitivity might be preferred over specificity in the early phases of a pandemic before surge capacity is reached.

Positive predictive values across various prediction thresholds, especially for the two weighted scales, were generally low despite increasing proportionately with the thresholds. This could be attributable, in part, to the general mildness of the pandemic with resultant low incidence of mortality in Nigeria. For instance, of the 12,289 confirmed COVID-19 cases in Nigeria between February 27 and June 6, 2020, 66% were asymptomatic at diagnosis and the overall cumulative incidence and case fatality were 5·6 per 100,000 population and 2·8%, respectively [8]—these figures were substantially lower than those from European countries during the same period.[40] Therefore, the developed predictive tools in the current study could perform differently during a more severe COVID-19 outbreak in Nigeria.

Conclusion

This study has investigated the possibility of using symptoms to predict COVID-19 positivity in Nigeria, and found the capacity of various symptom scores to be poor overall. This emphasises the need to continue the capacity strengthening of molecular laboratory to determine COVID-19 positivity in Nigeria.

Acknowledgments

We acknowledge the contribution of all the COVID-19 treatment centres and laboratories within the NCDC Laboratory Networks of both epidemiological and laboratory data to SORMAS on a regular basis. We express our profound gratitude to the NCDC SORMAS team for collating and maintaining a quality dataset utilised for this study. Last but not least, we thank Professor Jo Leonard-Bee of the University of Nottingham for her input on statistical analyses.

For peer review only - http://bmjopen.bmj.com/site/about/guidelines.xhtml

References

1. Nigeria Centre for Disease Control. *COVID-19 Situation Report: Weekly Epidemiological Report 3 (Epi Week 44)*. Abuja, https://ncdc.gov.ng/diseases/sitreps/?cat=14&name=An update of COVID-19 outbreak in Nigeria (2020).

2. Onyeaghala AA, and Olajide I. Managing COVID-19 outbreak in Nigeria: Matters arising. *Clinical Chemistry and Laboratory Medicine* 2020; 58: 1645–1650.

3. Oladimeji O, Atiba BP, and Adeyinka DA. Leveraging polymerase chain reaction technique (Genexpert) to upscaling testing capacity for SARS-CoV-2 (COVID-19) in Nigeria: A game changer. *Pan African Medical Journal* 2020; 35: 1–2.

4. Nigeria Centre for Disease Control. NCDC Coronavirus COVID-19 Microsite: Government Laboratories, https://covid19.ncdc.gov.ng/laboratory/ (2020, accessed 6 November 2020).

5. Nkengasong J. Let Africa into the market for COVID-19 diagnostics. *Nature* 2020; 580: 565.

6. Stites EC, and Wilen CB. The Interpretation of SARS-CoV-2 Diagnostic Tests. *Med*. Epub ahead of print 21 August 2020. DOI: 10.1016/j.medj.2020.08.001.

7. Smith DS, Richey EA, and Brunetto WL. A Symptom-Based Rule for Diagnosis of COVID-19. *SN Compr Clin Med* 2020; 1–8.

8. Elimian KO, Ochu CL, Ilori E, et al. Descriptive epidemiology of coronavirus disease 2019 in Nigeria, 27 February-6 June, 2020. *Epidemiol Infect* 2020; 1–42.

9. Wynants L, Van Calster B, Collins GS, et al. Prediction models for diagnosis and prognosis of covid-19: Systematic review and critical appraisal. *BMJ* 2020; 369: 18.

10. Abayomi A, Odukoya O, Osibogun A, et al. Presenting Symptoms and Predictors of Poor Outcomes Among 2,184 Patients with COVID-19 in Lagos State, Nigeria. *Int J Infect Dis*; 0. Epub ahead of print October 2020. DOI: 10.1016/j.ijid.2020.10.024.

11. Ibrahim OR, Suleiman BM, Abdullahi SB, et al. Epidemiology of COVID-19 and Predictors of Outcome in Nigeria: A Single-Center Study. *Am J Trop Med Hyg*. Epub ahead of print 26 October 2020. DOI: 10.4269/ajtmh.20-0759.

12. Elimian KO, Ochu CL, Ebhodaghe B, et al. Patient characteristics associated with COVID-19 positivity and fatality in Nigeria: retrospective cohort study. *BMJ Open* 2020; 10: 44079.

13. Nigeria Centre for Disease Control. *Case Definitions for Coronavirus Disease* . Abuja, https://covid19.ncdc.gov.ng/media/files/Case_Definition.pdf (2020, accessed 22 July 2020).

14. Nigeria Centre for Disease Control. *National interim guidelines for clinical management of COVID-19, version 2, May 2020*. Abuja, https://ncdc.gov.ng/themes/common/docs/protocols/177_1584210847.pdf (14 March 2020, accessed 26 May 2020).

15. World Health Organization. Laboratory testing for coronavirus disease (COVID-19) in suspected human cases: Interim guidance, 19 March 2020. *WHO*,

For peer review only - http://bmjopen.bmj.com/site/about/guidelines.xhtml

https://www.who.int/publications-detail/laboratory-testing-for-2019-novel-coronavirus-in-suspected-human-cases-20200117 (2020, accessed 1 June 2020).

16. Nigeria Centre for Disease Control. *National interim guidelines for clinical management of COVID-19: version 1*. Abuja, https://ncdc.gov.ng/themes/common/docs/protocols/177_1584210847.pdf (14 March 2020, accessed 27 July 2020).

17. Kronbichler A, Kresse D, Yoon S, et al. Asymptomatic patients as a source of COVID-19 infections: A systematic review and meta-analysis. *Int J Infect Dis*. Epub ahead of print 17 June 2020. DOI: 10.1016/j.ijid.2020.06.052.

18. Miri SM, Noorbakhsh F, Mohebbi SR, and Ghaemi A. Higher prevalence of asymptomatic or mild COVID-19 in children, claims and clues. *J Med Virol* 2020; jmv.26069.

19. Glantz SA, Slinker BK, and Neilands TB. *Primer of Applied Regression and Analysis of Variance* . 3rd ed. McGraw-Hill Medical, 2016.

20. Zweig M, and Campbell G. Receiver-operating characteristic (ROC) plots: A fundamental evaluation tool in clinical medicine. *Clinical Chemistry* 1993; 39: 561–577.

21. Moons KGM, Altman DG, Reitsma JB, et al. Transparent reporting of a multivariable prediction model for individual prognosis or diagnosis (TRIPOD): Explanation and elaboration. *Ann Intern Med* 2015; 162: W1–W73.

22. Salloum RG, Theis RP, Pbert L, et al. Stakeholder Engagement in Developing an Electronic Clinical Support Tool for Tobacco Prevention in Adolescent Primary Care. *Children* 2018; 5: 170.

23. Nemes S, Jonasson JM, Genell A, and Steineck G. Bias in odds ratios by logistic regression modelling and sample size. *BMC Med Res Methodol* 2009; 9: 56.

24. Katz MH. *Multivariable Analysis: A Practical Guide for Clinicians and Public Health Researchers*. 3rd Edition. Cambridge University Press, 2011.

25. Steyerberg EW, Harrell FE, Borsboom GJJM, et al. Internal validation of predictive models: Efficiency of some procedures for logistic regression analysis. *J Clin Epidemiol* 2001; 54: 774–781.

26. Adams DR, Wroblewski KE, Kern DW, et al. Factors Associated with Inaccurate Self-Reporting of Olfactory Dysfunction in Older US Adults. *Chem Senses* 2017; 42: 223–231.

27. Wang Z, Weng J, Li Z, et al. Development and Validation of a Diagnostic Nomogram to Predict COVID-19 Pneumonia. *medRxiv* 2020; 2020.04.03.20052068.

28. Chen X, Tang Y, Mo Y, et al. A diagnostic model for coronavirus disease 2019 (COVID-19) based on radiological semantic and clinical features: a multi-center study. *Eur Radiol* 2020; 30: 4893–4902.

29. Batista AF de M, Miraglia JL, Donato THR, and Chiavegatto Filho ADP. COVID-19 diagnosis prediction in emergency care patients: a machine learning approach. *medRxiv* 2020; 2020.04.04.20052092.

For peer review only

For peer review only - http://bmjopen.bmj.com/site/about/guidelines.xhtml

30. Brinati D, Campagner A, Ferrari D, et al. Detection of COVID-19 Infection from Routine Blood Exams with Machine Learning: a Feasibility Study. *medRxiv* 2020; 2020.04.22.20075143.

31. Kurstjens S, Van Der Horst A, Herpers R, et al. Rapid identification of SARS-CoV-2-infected patients at the emergency department using routine testing. *Clin Chem Lab Med* 2020; 58: 1587–1593.

32. Tordjman M, Mekki A, Mali R, et al. Pre-test probability for SARS-Cov-2-related Infection Score: the PARIS score. *medRxiv* 2020; 2020.04.28.20081687.

33. Sun Y, Koh V, Marimuthu K, et al. Epidemiological and Clinical Predictors of COVID-19. *Clin Infect Dis* 2020; 71: 786–792.

34. Roland LT, Gurrola JG, Loftus PA, Cheung SW, and Chang JL. Smell and taste symptom-based predictive model for COVID-19 diagnosis. *Int Forum Allergy Rhinol*. Epub ahead of print 2020. DOI: 10.1002/alr.22602.

35. Menni C, Valdes AM, Freidin MB, et al. Real-time tracking of self-reported symptoms to predict potential COVID-19. *Nat Med* 2020; 26: 1037–1040.

36. Borsetto D, Hopkins C, Philips V, et al. Self-reported alteration of sense of smell or taste in patients with COVID-19: a systematic review and meta-analysis on 3563 patients. *Rhinology*. Epub ahead of print 6 July 2020. DOI: 10.4193/Rhin20.185.

37. Myles PR, Nguyen-Van-Tam JS, Lim WS, et al. Comparison of CATs, CURB-65 and PMEWS as Triage Tools in Pandemic Influenza Admissions to UK Hospitals: Case Control Analysis Using Retrospective Data. *PLoS One* 2012; 7: e34428.

38. Simon R. Clinical trial designs for evaluating the medical utility of prognostic and predictive biomarkers in oncology. *Personalized Medicine* 2010; 7: 33–47.

39. Akobeng AK. Understanding diagnostic tests 3: Receiver operating characteristic curves. *Acta Paediatrica, International Journal of Paediatrics* 2007; 96: 644–647.

40. Kinross P, Suetens C, Gomes Dias J, et al. Rapidly increasing cumulative incidence of coronavirus disease (COVID-19) in the European Union/European Economic Area and the United Kingdom, 1 January to 15 March 2020. *Eurosurveillance* 2020; 25: 2000285.

For peer review only

Competing interests

None declared.

Funding

This study received no specific grant from any funding agency in the public, commercial or not-for-profit sectors.

Data sharing statement

The dataset utilised for this study can be made available upon reasonable request to the Head of NCDC Research at chinwe.ochu@ncdc.gov.ng

Authors' contribution

KE contributed to the conceptualization, literature search, data management, data analysis, writing (original draft, review and editing) and formatting of document. OA contributed to the conceptualization, writing (review and editing), formatting of manuscript and funding acquisition. JB contributed to the conceptualization, data management, data analysis, writing (original draft, review and editing) and formatting of document. PM contributed to the writing (original draft, review and editing), literature search and formatting of manuscript. CO contributed to the writing (review and editing) and formatting of manuscript. CK contributed to the writing (review and editing) and formatting of manuscript. TO contributed to the writing (review and editing) and formatting of manuscript. GG contributed to the writing (review and editing) and formatting of manuscript. AO contributed to the writing (review and editing) and formatting of manuscript. HZ contributed to the writing (review and editing) and formatting of manuscript. CO contributed to the writing (review and editing) and formatting of manuscript. BE contributed to the writing (review and editing) and formatting of manuscript. OI contributed to the writing (review and editing) and formatting of manuscript. CDN contributed to the writing (review and editing) and formatting of manuscript. WN contributed to the writing (review and editing) and formatting of manuscript. IAS contributed to data acquisition and curation, and writing (review) of manuscript. OA contributed to the writing (review and editing) and formatting of manuscript. OF contributed to the writing (review and editing) and formatting of manuscript. CA contributed to data acquisition and curation, and writing (review) of manuscript. CE contributed to the writing (review and editing) and formatting of manuscript. JA contributed to the writing (review and editing) and formatting of manuscript. AO contributed to the writing (review and editing) and formatting of manuscript. JA contributed to

For peer review only - http://bmjopen.bmj.com/site/about/guidelines.xhtml

the writing (review and editing) and formatting of manuscript. EJ contributed to the writing (review and editing) and formatting of manuscript. AB contributed to the writing (review and editing) and formatting of manuscript. BM contributed to the writing (review and editing) and formatting of manuscript. BAS contributed to the writing (review and editing) and formatting of manuscript. AJ contributed to the writing (review and editing) and formatting of manuscript. KH contributed to the writing (review and editing) and formatting of manuscript. CI contributed to the writing (review and editing) and formatting of manuscript. SOB contributed to the writing (review and editing) and formatting of manuscript. MA contributed to the writing (review and editing) and formatting of manuscript. NM contributed to supervision, writing (review), funding acquisition, project administration, and resources. JO contributed to supervision, writing (review), funding acquisition, project administration, and resources. EI contributed to supervision, writing (review), funding acquisition, project administration, and resources. TA contributed to the writing (review and editing) and formatting of manuscript. EI contributed to the writing (original draft, review and editing), literature search and formatting of manuscript. CI contributed to conceptualization, supervision, writing (review and editing), funding acquisition, project administration, and resources. All authors invest significant contributions and approved the final draft.

Legends

Figure 1 presents a flow chart showing the processes for selecting records utilised for this study.

Figure 2 presents a comparison of unweighted, statistically weighted and clinically weighted combined scores for predicting COVID-19 positivity. Specifically, Figure 2a focuses on children, 2b on adults, and 2c on the elderly populations.

For peer review only - http://bmjopen.bmj.com/site/about/guidelines.xhtml

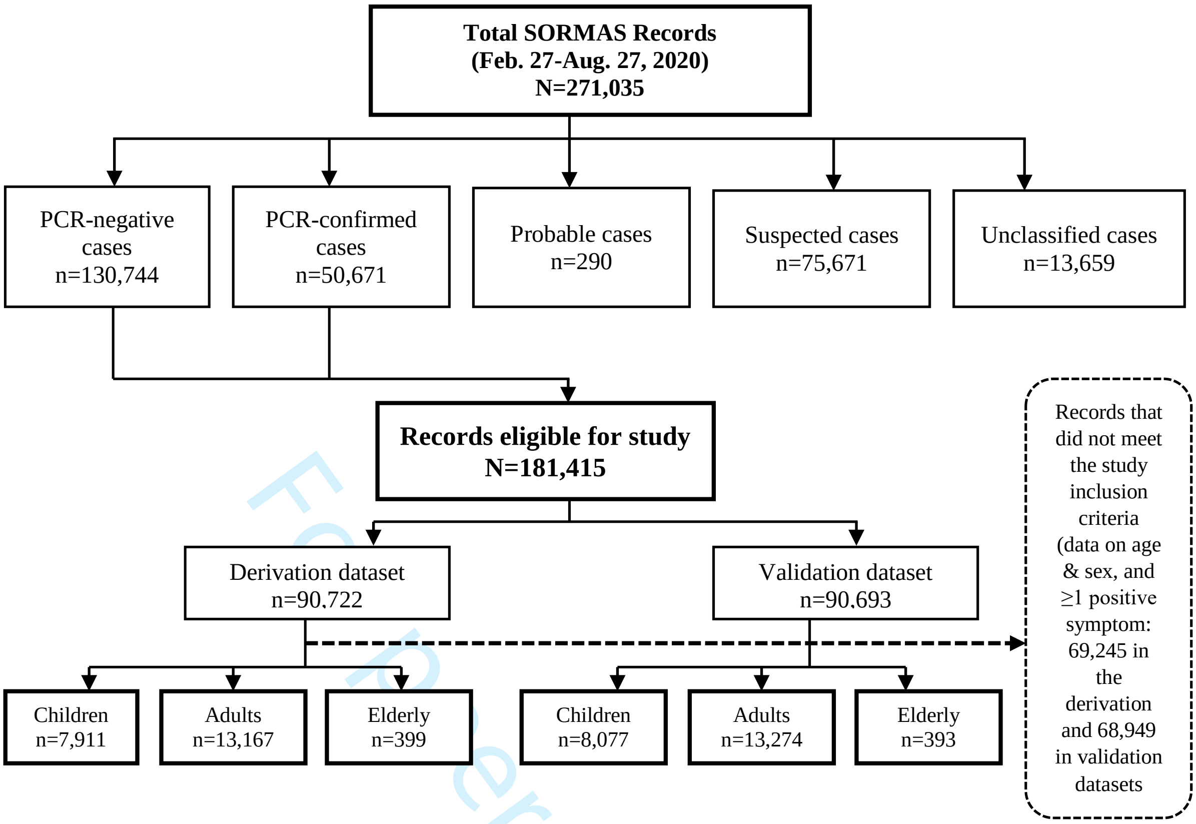

Figure 1: Flow charts showing the processes for selecting records for this study

For peer review only

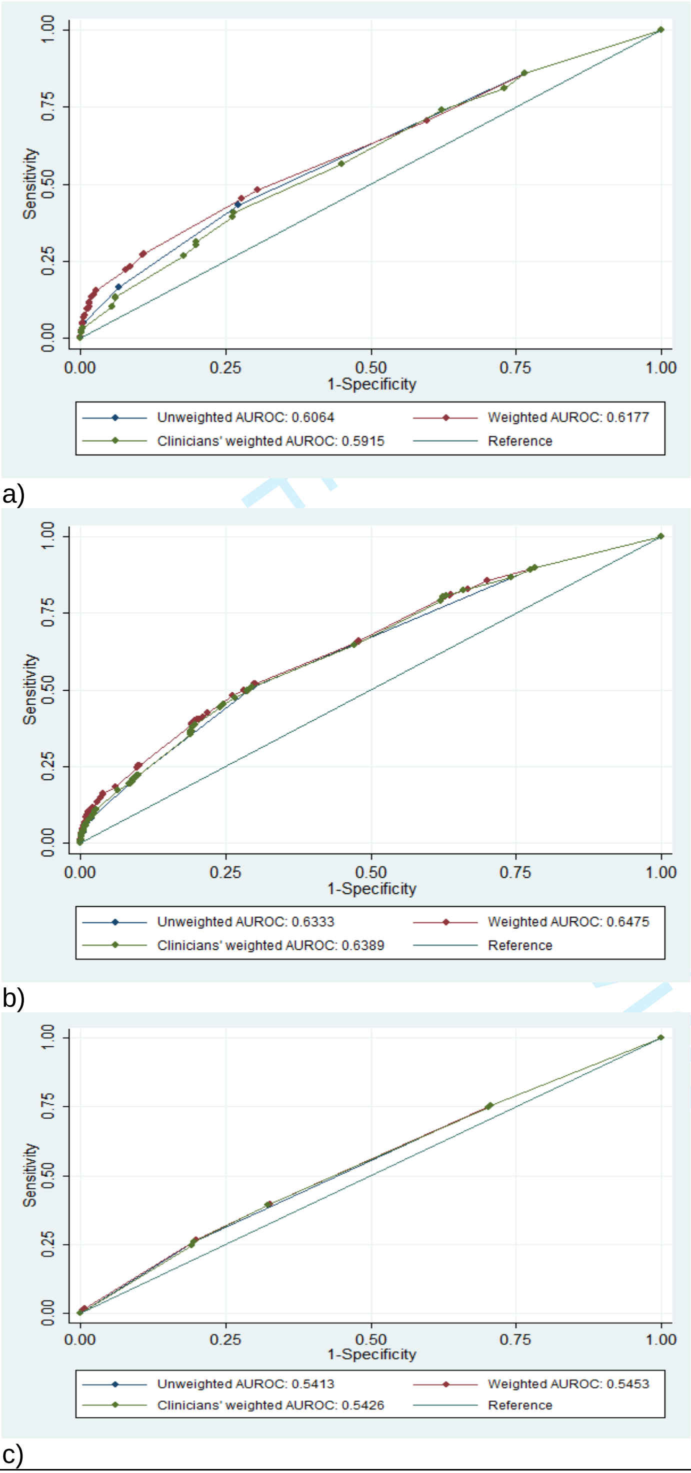

Figure 2: Comparison of unweighted, statistically weighted and clinically weighted combined scores for predicting COVID-19 positivity in children (a), adults (b) and the elderly (c)

Supplementary Files

Supplementary Table 1: NCDC COVID-19 case definitions, February 27-August 27, 2020	
**Case category**	**Definition/criteria**
**Suspected COVID-19 case**	**1. Symptoms with international travel**<br>Anyone with acute respiratory symptoms (fever and either cough, difficulty breathing or shortness of breath) OR new respiratory symptoms (cough, difficulty breathing or shortness of breath) without fever and no other explanation, **AND** a history of travel to or residence in a country reporting cases within 14 days before symptom onset; Or new respiratory symptoms with contact to a confirmed case in the last 14 days before symptom onset;<br>OR<br>**2. Symptoms with contact to confirmed case**<br>Anyone with new respiratory symptoms (cough, difficulty breathing or shortness of breath, with or without fever), **AND** contact with a confirmed or probable COVID-19 case in the last 14 days prior to symptom onset;<br>OR<br>**3. Acute respiratory illness in an area of moderate or high COVID-19 prevalence with no other explanation**<br>Anyone with acute respiratory illness within the last 10 days (fever and either cough, difficulty breathing or shortness of breath), **AND** absence of an alternative diagnosis that explains the clinical presentation **AND** residing or working in the last 14 days in an area identified by NCDC as a moderate or high prevalence region.
**Probable case**	Any suspected case:<br>• For whom testing for COVID-19 is indeterminate test results;<br>• For whom testing was positive on a pan-coronavirus assay; OR<br>• Where samples were not collected prior to the demise of a suspect case
**Confirmed case**	Anyone with laboratory confirmation of SARS-CoV-2 infection with or without signs and symptoms.
**Contact**	Defined as anyone who experienced any one of the following exposures during the 2 days before and/or within 14 days after the onset of symptoms of a probable or confirmed case:<br>• Face-to-face contact with a probable or confirmed case within 1 metre and for more than 15 minutes;<br>• Direct physical contact with a probable or confirmed case;<br>• Direct care for a patient with probable or confirmed COVID-19 disease without using proper personal protective equipment; OR<br>• Other situations as indicated by local risk assessments.<br><br>For confirmed asymptomatic cases, the period of contact is measured as the 2 days before, through the 14 days after the date on which the sample was taken which led to confirmation.

For peer review only

For peer review only - http://bmjopen.bmj.com/site/about/guidelines.xhtml

Assessment of multicollinearity between clinical symptoms and COVID-19 positivity

The VIF and tolerance values (Supplementary Tables 3a, 3b and 3c for results) from the assessment of multicollinearity between the statistically significant symptoms in the unadjusted models and COVID-19 positivity in children, adults and elderly were less than 10 and greater than 0.1, implying it was appropriate to combine the independent symptoms into an index or clinical score.

Supplementary Table 2a: Findings from the assessment of multicollinearity between clinical signs and symptoms and COVID-19 diagnosis in children

Clinical sign/symptom	Derivation dataset n=7,911	
	Variance of Inflation Factor (VIF)	Tolerance
Cough	1.02	0.9800
Runny nose	1.03	0.9724
Fatigue	1.01	0.9864
Loss of taste	1.95	0.5132
Loss of smell	1.95	0.5139
Fever	1.01	0.9853

Mean VIF=1.33

Supplementary Table 2b: Findings from the assessment of multicollinearity between clinical signs and symptoms and COVID-19 diagnosis in adults

Clinical sign/symptom	Derivation dataset n=13,167	
	Variance of Inflation Factor (VIF)	Tolerance
Cough	1.05	0.9569
Difficulty breathing	1.04	0.9613
Rapid breathing	1.01	0.9901
Runny nose	1.03	0.9734
Gastrointestinal tract symptom	1.02	0.9847
Chest pain	1.02	0.9770
Fatigue	1.02	0.9805
Headache	1.04	0.9609
Loss of taste	1.79	0.5574
Loss of smell	1.79	0.5581
Fever	1.03	0.9678

Mean VIF=1.17

Supplementary Table 2c: Findings from the assessment of multicollinearity between clinical signs and symptoms and COVID-19 diagnosis in elderly

Clinical sign/symptom	Derivation dataset n=3,369	
	Variance of Inflation Factor (VIF)	Tolerance
Cough	1.01	0.9871
Difficulty breathing	1.02	0.9800
Loss of smell	1.01	0.9891

Mean VIF=1.01

For peer review only - http://bmjopen.bmj.com/site/about/guidelines.xhtml

Supplementary Table 3: Assignment of clinical weights based on clinicians' judgement or experiences from managing COVID-19 cases in Nigeria

Symptom	Weighting based on clinicians' judgement						
	**Delta State**	**FCT**	**Kaduna State**	**Gombe State**	**Infectious disease consultant§**	**Infectious disease consultant¶**	**Average weight‡**
**Children**							
Cough	3	5	3	1	3	3	**3.0**
Runny nose	2	2	4	2	2	2	**2.3**
Fatigue	2	2	2	1	1	1	**1.5**
Loss of taste	1	3	2	1	1	1	**1.5**
Loss of smell	1	3	2	1	1	1	**1.5**
Fever	2	5	3	5	3	5	**3.8**
**Adults**							
Cough	5	5	4	5	3	3	**4.2**
Runny nose	3	1	2	3	2	3	**2.3**
Chest pain	3	1	2	1	3	2	**2.0**
Fatigue	5	1	3	4	2	4	**3.2**
Headache	3	2	4	1	2	1	**2.2**
Loss of taste	4	3	3	4	5	3	**3.7**
Loss of smell	5	3	3	4	5	3	**3.8**
Fever	3	5	4	4	3	5	**4.0**
**Elderly**							
Cough	4	5	4	5	3	4	**4.2**
Breathing difficulty	5	5	4	5	5	5	**4.8**
Loss of smell	5	3	3	5	5	3	**4.0**

FCT: Federal Capital Territory
‡: Sum of weights from various sources divided by 6 (i.e. number of weight sources)
§: Provides care to COVID-19 patients in a health-facility in the FCT
¶: Provides home-care to COVID-19 patients in the FCT

For peer review only

For peer review only - http://bmjopen.bmj.com/site/about/guidelines.xhtml

Table 4: Baseline sociodemographic and clinical characteristics of the study participants in relation to COVID-19 infection (derivation dataset)

Variable	Children		Adults		Elderly	
	PCR-confirmed cases [n=2,155 (%)]	PCR positive and negative cases [N=7,911 (%)]	PCR-confirmed cases [n=4,542 (%)]	PCR positive and negative cases [N=13,167 (%)]	PCR-confirmed cases [n=159 (%)]	PCR positive and negative cases [N=399 (%)]
**Socio-demographic features**						
**Sex**						
Female	902 (41.86)	3,065 (38.74)	1,534 (33.77)	4,814 (36.56)	49 (30.82)	148 (37.09)
Male	1,253 (58.14)	4,846 (61.26)†	3,008 (66.23)	8,353 (63.44)‡	110 (69.18)	251 (62.91)†
**Geopolitical zone§**						
South-west	632 (29.33)	1,761 (22.26)	1,285 (28.29)	3,111 (23.63)	47 (29.56)	97 (24.31)
South-south	712 (33.04)	2,823 (35.68)	1,617 (35.60)	5,441 (41.32)	51 (32.08)	164 (41.10)
South-east	46 (2.13)	129 (1.63)	139 (3.06)	294 (2.23)	11 (6.92)	16 (4.01)
North-central	402 (18.65)	1,557 (19.68)	749 (16.49)	2,045 (15.53)	13 (8.18)	32 (8.02)
North-west	315 (14.62)	1,498 (18.94)	609 (13.41)	1,978 (15.02)	20 (12.58)	63 (15.79)
North-east	48 (2.23)	143 (1.81)‡	143 (3.15)	298 (2.26)‡	17 (10.69)	27 (6.77)†
**Setting**						
Rural	111 (5.15)	429 (5.42)	233 (5.13)	755 (5.73)	20 (12.58)	41 (10.28)
Urban	1,015 (47.10)	3,467 (43.83)	2,218 (48.83)	6,485 (49.25)	92 (57.86)	209 (52.38)
Missing	1,029 (47.75)	4,015 (50.75)†	2,091 (46.04)	5,927 (45.01)†	47 (29.56)	149 (37.34)†
**Education**						
None	25 (1.16)	107 (1.35)	62 (1.37)	169 (1.28)	15 (9.43)	31 (7.77)
Nursery	16 (0.74)	70 (0.88)	2 (0.04)	4 (0.03)	0 (0.00)	0 (0.00)
Primary	67 (3.11)	250 (3.16)	41 (0.90)	151 (1.15)	2 (1.26)	5 (1.25)
Secondary	181 (8.40)	681 (8.61)	378 (8.32)	1,098 (8.34)	14 (8.81)	33 (8.27)
Tertiary	692 (32.11)	2,043 (25.82)	1,574 (34.65)	4,071 (30.92)	45 (28.30)	82 (20.55)
Other	98 (4.55)	540 (6.83)	134 (2.95)	336 (2.55)	13 (8.18)	25 (6.27)
Missing	1,076 (49.93)	4,220 (53.34)‡	2,351 (51.76)	7,338 (55.73)‡	70 (44.03)	223 (55.89)†
**Occupation**						
Student/pupil	297 (13.78)	1,401 (17.71)	212 (4.67)	775 (5.89)	1 (0.63)	2 (0.50)
Child/housewife	67 (3.11)	241 (3.05)	81 (1.78)	228 (1.73)	7 (4.40)	23 (5.76)
Business/trading	106 (4.92)	384 (4.85)	397 (8.74)	1,050 (7.97)	6 (3.77)	18 (4.51)
Transporter	6 (0.28)	35 (0.44)	24 (0.53)	87 (0.66)	0 (0.00)	1 (0.25)
Healthcare worker	269 (12.48)	950 (12.01)	479 (10.55)	1,473 (11.19)	4 (2.52)	13 (3.26)
Laboratorian	10 (0.46)	26 (0.33)	12 (0.26)	35 (0.27)	0 (0.00)	0 (0.00)
Farmer	18 (0.84)	92 (1.16)	84 (1.85)	283 (2.15)	16 (10.06)	27 (6.77)
Animal-related worker	3 (0.14)	11 (0.14)	15 (0.33)	40 (0.30)	1 (0.63)	4 (1.00)
Religious/traditional leader	2 (0.09)	11 (0.14)	30 (0.66)	69 (0.52)	2 (1.26)	4 (1.00)

Other	826 (38.33)	2,609 (32.98)	2,008 (44.21)	5,633 (42.78)	85 (53.46)	187 (46.87)
Missing	551 (25.57)	2,151 (27.19)‡	1,200 (26.42)	3,494 (26.54)‡	37 (23.27)	120 (30.08)NS
**Clinical signs and symptoms**						
**Clinical outcome**						
Recovered	1,396 (64.78)	2,221 (28.07)	2,844 (62.62)	3,677 (27.93)	70 (44.03)	89 (22.31)
Dead	15 (0.70)	24 (0.30)	229 (5.04)	252 (1.91)	50 (31.45)	50 (12.53)
No outcome yet	744 (34.52)	5,666 (71.62)‡	1,469 (32.34)	9,238 (70.16)‡	39 (24.53)	260 (65.16)‡
Obvious (visible to healthcare workers on sight)						
**Chills/sweat**						
No	2,119 (98.33)	7,791 (98.48)	4,471 (98.44)	12,978 (98.56)	158 (99.37)	393 (98.50)
Yes	36 (1.67)	120 (1.52)NS	71 (1.56)	189 (1.44)NS	1 (0.63)	6 (1.50)NS
**Cough**						
No	1,162 (53.92)	4,725 (59.73)	2,023 (44.54)	7,179 (54.52)	53 (33.33)	160 (40.10)
Yes	993 (46.08)	3,186 (40.27)‡	2,519 (55.46)	5,988 (45.48)‡	106 (66.67)	239 (59.90)††
**Breathing difficulty**						
No	1,912 (88.72)	7,031 (88.88)	3,778 (83.18)	11,181 (84.92)	102 (64.15)	283 (70.93)
Yes	243 (11.28)	880 (11.12)NS	764 (16.82)	1,986 (15.08)‡	57 (35.85)	116 (29.07)††
**Rapid breathing**						
No	2,123 (98.52)	7,802 (98.62)	4,470 (98.41)	13,008 (98.79)	155 (97.48)	392 (98.25)
Yes	32 (1.48)	109 (1.38)NS	72 (1.59)	159 (1.21)††	4 (2.52)	7 (1.75)NS
**Runny nose**						
No	1,450 (67.29)	5,817 (73.53)	3,386 (74.55)	10,218 (77.60)	130 (81.76)	330 (82.71)
Yes	705 (32.71)	2,094 (26.47)‡	1,156 (25.45)	2,949 (22.40)‡	29 (18.24)	69 (17.29)NS
Askable (can be found out by asking questions of patients/relatives)						
**Abdominal pain/diarrhoea**						
No	2,021 (93.78)	7,371 (93.17)	4,193 (92.32)	12,222 (92.82)	151 (94.97)	372 (93.23)
Yes	134 (6.22)	540 (6.83)NS	349 (7.68)	945 (7.18)NS	8 (5.03)	27 (6.77)NS
**GIT symptoms**						
No	1,942 (90.12)	7,110 (89.87)	4,048 (89.12)	11,859 (90.07)	145 (91.19)	362 (90.73)
Yes	213 (9.88)	801 (10.13)NS	494 (10.88)	1,308 (9.93)††	14 (8.81)	37 (9.27)NS
**Chest pain**						
No	2,089 (96.94)	7,711 (97.47)	4,348 (95.73)	12,759 (96.90)	153 (96.23)	387 (96.99)
Yes	66 (3.06)	200 (2.53)NS	194 (4.27)	408 (3.10)‡	6 (3.77)	12 (3.01)NS
**Fatigue**						
No	2,036 (94.48)	7,543 (95.35)	4,269 (93.99)	12,474 (94.74)	144 (90.57)	372 (93.23)
Yes	119 (5.52)	368 (4.65)††	273 (6.01)	693 (5.26)††	15 (9.43)	27 (6.77)NS
**Headache**						
No	1,829 (84.87)	6,796 (85.91)	3,880 (85.42)	11,592 (88.04)	149 (93.71)	367 (91.98)

For peer review only - http://bmjopen.bmj.com/site/about/guidelines.xhtml

Yes	326 (15.13)	1,115 (14.09)NS	662 (14.58)	1,575 (11.96)‡	10 (6.29)	32 (8.02)NS
**Musculoskeletal pain**						
No	2,097 (97.31)	7,710 (97.46)	4,440 (97.75)	12,878 (97.81)	152 (95.60)	386 (96.74)
Yes	58 (2.69)	201 (2.54)NS	102 (2.25)	289 (2.19)NS	7 (4.40)	13 (3.26)NS
**Sore throat**						
No	1,615 (74.94)	5,950 (75.21)	3,476 (76.53)	9,766 (74.17)	129 (81.13)	316 (79.20)
Yes	540 (25.06)	1,961 (24.79)NS	1,066 (23.47)	3,401 (25.83)‡	30 (18.87)	83 (20.80)NS
**Loss of taste**						
No	1,909 (88.58)	7,562 (95.59)	4,111 (90.51)	12,581 (95.55)	153 (96.23)	391 (97.99)
Yes	246 (11.42)	349 (4.41)‡	431 (9.49)	586 (4.45)‡	6 (3.77)	8 (2.01)†
**Loss of smell**						
No	1,851 (85.89)	7,489 (94.67)	4,067 (89.54)	12,545 (95.28)	152 (95.60)	390 (97.74)
Yes	304 (14.11)	422 (5.33)‡	475 (10.46)	622 (4.72)‡	7 (4.40)	9 (2.26)†
Measureable signs/symptoms						
**Fever**						
No	1,126 (52.25)	4,634 (58.58)	2,306 (50.77)	7,723 (58.65)	79 (49.69)	219 (54.89)
Yes	1,029 (47.75)	3,277 (41.41)‡	2,236 (49.23)	5,444 (41.35)‡	80 (50.31)	180 (45.11)NS

†=p-value<0.05; ‡=p<0.001; NS=p>0.05 or Not significant
GIT= gastrointestinal (nausea + vomiting)
Musculoskeletal pain= muscle /joint pain
§=State composition of geopolitical zones in Nigeria: South-West (Ekiti, Lagos, Ogun, Ondo, Osun and Oyo); South-South (Akwa-Ibom, Bayelsa, Cross-River, Rivers, Delta and Edo); South-East (Abia, Anambra, Ebonyi, Enugu and Imo); North-Central (Benue, Kogi, Kwara, Nasarawa, Niger, and Plateau States as well as the FCT); North-West (Jigawa, Kaduna, Kano, Katsina, Kebbi, Sokoto and Zamfara); and North-East (Adamawa, Bauchi, Borno, Gombe, Taraba and Yobe).

For peer review only - http://bmjopen.bmj.com/site/about/guidelines.xhtml

Table 5: Baseline sociodemographic and clinical characteristics of the study participants in relation to COVID-19 infection (validation dataset)

Variable	Children		Adults		Elderly	
	PCR-confirmed cases [n=2,260 (%)]	PCR positive and negative cases [N=8,077 (%)]	PCR-confirmed cases [n=4,612 (%)]	PCR positive and negative cases [N=13,274 (%)]	PCR-confirmed cases [n= 158 (%)]	PCR positive and negative cases [N=393 (%)]
**Socio-demographic features**						
**Sex**						
Female	871 (38.54)	2,986 (36.97)	1,546 (33.52)	4,808 (36.22)	61 (38.61)	151 (38.42)
Male	1,389 (61.46)	5,091 (63.03)NS	3,066 (66.48)	8,466 (63.78)‡	97 (61.39)	242 (61.58)NS
**Geopolitical zone§**						
South-west	670 (29.65)	1,778 (22.01)	1,307 (28.34)	3,134 (23.61)	39 (24.68)	88 (22.39)
South-south	723 (31.99)	2,866 (35.48)	1,611 (34.93)	5,470 (41.21)	57 (36.08)	176 (44.78)
South-east	44 (1.95)	149 (1.84)	134 (2.91)	315 (2.37)	14 (8.86)	21 (5.34)
North-central	414 (18.32)	1,499 (18.56)	777 (16.85)	2,016 (15.19)	17 (10.76)	44 (11.20)
North-west	343 (15.18)	1,620 (20.06)	649 (14.07)	2,027 (15.27)	25 (15.82)	53 (13.49)
North-east	66 (2.92)	165 (2.04)‡	134 (2.91)	312 (2.35)‡	6 (3.80)	11 (2.80)†
**Setting**						
Rural	119 (5.27)	458 (5.67)	278 (6.03)	807 (6.08)	15 (9.49)	33 (8.40)
Urban	1,033 (45.71)	3,476 (43.04)	2,269 (49.20)	6,603 (49.74)	91 (57.59)	214 (54.45)
Missing	1,108 (49.03)	4,143 (51.29)†	2,065 (44.77)	5,864 (44.18)NS	52 (32.91)	146 (37.15)NS
**Education**						
None	33 (1.46)	110 (1.36)	66 (1.43)	199 (1.50)	10 (6.33)	17 (4.33)
Nursery	14 (0.62)	73 (0.90)	1 (0.02)	3 (0.02)	0 (0.00)	0 (0.00)
Primary	63 (2.79)	270 (3.34)	61 (1.32)	171 (1.29)	6 (3.80)	13 (3.31)
Secondary	204 (9.03)	698 (8.64)	414 (8.98)	1,164 (8.77)	9 (5.70)	25 (6.36)
Tertiary	718 (31.77)	2,023 (25.05)	1,567 (33.98)	4,074 (30.69)	42 (26.58)	86 (21.88)
Other	81 (3.58)	525 (6.50)	127 (2.75)	352 (2.65)	7 (4.43)	20 (5.09)
Missing	1,147 (50.75)	4,378 (54.20)‡	2,376 (51.52)	7,311 (55.08)‡	84 (53.16)	232 (59.03)NS
**Occupation**						
Student/pupil	302 (13.36)	1,408 (17.43)	222 (4.81)	741 (5.58)	0 (0.00)	0 (0.00)
Child/housewife	66 (2.92)	248 (3.07)	97 (2.10)	272 (2.05)	6 (3.80)	19 (4.83)
Business/trading	111 (4.91)	381 (4.72)	360 (7.81)	993 (7.48)	13 (8.23)	29 (7.38)
Transporter	5 (0.22)	45 (0.56)	21 (0.46)	87 (0.66)	0 (0.00)	2 (0.51)
Healthcare worker	332 (14.69)	1,003 (12.42)	494 (10.71)	1,504 (11.33)	3 (1.90)	7 (1.78)
Laboratorian	5 (0.22)	14 (0.17)	14 (0.30)	28 (0.21)	0 (0.00)	0 (0.00)
Farmer	18 (0.80)	100 (1.24)	89 (1.93)	306 (2.31)	8 (5.06)	20 (5.09)

Animal-related worker	5 (0.22)	17 (0.21)	10 (0.22)	40 (0.30)	0 (0.00)	5 (1.27)
Religious/traditional leader	3 (0.13)	13 (0.16)	23 (0.50)	60 (0.45)	4 (2.53)	5 (1.27)
Other	807 (35.71)	2,517 (31.16)	2,064 (44.75)	5,775 (43.51)	79 (50.00)	188 (47.84)
Missing	606 (26.81)	2,331 (28.86)‡	1,218 (26.41)	3,468 (26.13)†	45 (28.48)	118 (30.03)NS
**Clinical signs and symptoms**						
**Clinical outcome**						
Recovered	1,451 (64.20)	2,222 (27.51)	2,850 (61.80)	3,636 (27.39)	80 (50.63)	95 (24.17)
Dead	21 (0.93)	27 (0.33)	256 (5.55)	297 (2.24)	33 (20.89)	34 (8.65)
No outcome yet	788 (34.87)	5,828 (72.16)‡	1,506 (32.65)	9,341 (70.37)‡	45 (28.48)	264 (67.18)‡
Obvious (visible to healthcare workers on sight)						
**Chills/sweat**						
No	2,236 (98.94)	7,973 (98.71)	4,538 (98.40	13,051 (98.32)	157 (99.37)	390 (99.24)
Yes	24 (1.06)	104 (1.29)NS	74 (1.60)	223 (1.68)NS	1 (0.63)	3 (0.76)NS
**Cough**						
No	1,183 (52.35)	4,668 (57.79)	2,107 (45.69)	7,212 (54.33)	61 (38.61)	161 (40.97)
Yes	1,077 (47.65)	3,409 (42.21)‡	2,505 (54.31)	6,062 (45.67)‡	97 (61.39)	232 (59.03)NS
**Breathing difficulty**						
No	2,024 (89.56)	7,252 (89.79)	3,771 (81.76)	11,244 (84.71)	98 (62.03)	258 (65.65)
Yes	236 (10.44)	825 (10.21)NS	841 (18.24)	2,030 (15.29)‡	60 (37.97)	135 (34.35)NS
**Rapid breathing**						
No	2,222 (98.32)	7,981 (98.81)	4,532 (98.27)	13,104 (98.72)	152 (96.20)	379 (96.44)
Yes	38 (1.68)	96 (1.19)†	80 (1.73)	170 (1.28)†	6 (3.80)	14 (3.56)NS
**Runny nose**						
No	1,607 (71.11)	6,018 (74.51)	3,406 (73.85)	10,278 (77.43)	129 (81.65)	328 (83.46)
Yes	653 (28.89)	2,059 (25.49)‡	1,206 (26.15)	2,996 (22.57)‡	29 (18.35)	65 (16.54)NS
Askable (can be found out by asking questions of patients/relatives)						
**Abdominal pain/diarrhoea**						
No	2,121 (93.85)	7,568 (93.70)	4,271 (92.61)	12,358 (93.10)	146 (92.41)	356 (90.59)
Yes	139 (6.15)	509 (6.30)NS	341 (7.39)	916 (6.90)NS	12 (7.59)	37 (9.41)NS
**GIT symptoms**						
No	2,030 (89.82)	7,265 (89.95)	4,052 (87.86)	11,907 (89.70)	137 (86.71)	343 (87.28)
Yes	230 (10.18)	812 (10.05)NS	560 (12.14)	1,367 (10.30)‡	21 (13.29)	50 (12.72)NS
**Chest pain**						
No	2,197 (97.21)	7,866 (97.39)	4,434 (96.14)	12,862 (96.90)	150 (94.94)	379 (96.44)
Yes	63 (2.79)	211 (2.61)NS	178 (3.86)	412 (3.10)‡	8 (5.06)	14 (3.56)NS
**Fatigue**						
No	2,107 (93.23)	7,686 (95.16)	4,314 (93.54)	12,540 (94.47)	148 (93.67)	378 (96.18)
Yes	153 (6.77)	391 (4.84)‡	298 (6.46)	734 (5.53)†	10 (6.33)	15 (3.82)†

**Headache**						
No	1,924 (85.13)	6,916 (85.63)	3,969 (86.06)	11,691 (88.07)	138 (87.34)	371 (94.40)
Yes	336 (14.87)	1,161 (14.37)NS	643 (13.94)	1,583 (11.93)‡	20 (12.66)	22 (5.60)‡
**Musculoskeletal pain**						
No	2,202 (97.43)	7,885 (97.62)	4,461 (96.73)	12,905 (97.22)	154 (97.47)	385 (97.96)
Yes	58 (2.57)	192 (2.38)NS	151 (3.27)	369 (2.78)†	4 (2.53)	8 (2.04)NS
**Sore throat**						
No	1,691 (74.82)	6,018 (74.51)	3,492 (75.72)	9,817 (73.96)	130 (82.28)	333 (84.73)
Yes	569 (25.18)	2,059 (25.49)NS	1,120 (24.28)	3,457 (26.04)‡	28 (17.72)	60 (15.27)NS
**Loss of taste**						
No	2,016 (89.20)	7,721 (95.59)	4,190 (90.85)	12,687 (95.58)	150 (94.94)	379 (96.44)
Yes	244 (10.80)	356 (4.41)‡	422 (9.15)	587 (4.42)‡	8 (5.06)	14 (3.56)NS
**Loss of smell**						
No	1,964 (86.90)	7,671 (94.97)	4,119 (89.31)	12,653 (95.32)	155 (98.10)	388 (98.73)
Yes	296 (13.10)	406 (5.03)‡	493 (10.69)	621 (4.68)‡	3 (1.90)	5 (1.27)NS
Measureable signs/symptoms						
**Fever**						
No	1,224 (54.16)	4,796 (59.38)	2,249 (48.76)	7,711 (58.09)	84 (53.16)	234 (59.54)
Yes	1,036 (45.84)	3,281 (40.62)‡	2,363 (51.24)	5,563 (41.91)‡	74 (46.84)	159 (40.46)†

†=p-value<0.05; ‡=p<0.001; NS=p>0.05 or Not significant
GIT= gastrointestinal (nausea + vomiting)
Musculoskeletal pain= muscle /joint pain
§=State composition of geopolitical zones in Nigeria: South-West (Ekiti, Lagos, Ogun, Ondo, Osun and Oyo); South-South (Akwa-Ibom, Bayelsa, Cross-River, Rivers, Delta and Edo); South-East (Abia, Anambra, Ebonyi, Enugu and Imo); North-Central (Benue, Kogi, Kwara, Nasarawa, Niger, and Plateau States as well as the FCT); North-West (Jigawa, Kaduna, Kano, Katsina, Kebbi, Sokoto and Zamfara); and North-East (Adamawa, Bauchi, Borno, Gombe, Taraba and Yobe).

For peer review only - http://bmjopen.bmj.com/site/about/guidelines.xhtml

Table 6: Predictive value of individual clinical symptoms for predicting COVID-19 positivity in children (n=8,077)

Variable	ROC area (95% CI)	Sensitivity (%) (95%CI)	Specificity (%) (95%CI)	PPV (%) (95% CI)	NPV (%) (95% CI)
**Cough**	0.54 (0.53-0.55)	**47.7 (45.6-49.7)**	59.9 (58.6-61.2)	31.6 (30.0-33.2)	**74.7 (73.4-75.9)**
**Runny nose**	0.52 (0.51-0.53)	28.9 (27.0-30.8)	75.8 (74.7-76.9)	31.7 (29.7-33.8)	73.3 (72.2-74.4)
**Fatigue**	0.51 (0.51-0.52)	6.8 (5.8-7.9)	95.9 (95.4-96.4)	39.1 (34.3-44.2)	72.6 (71.6-73.6)
**Loss of taste**	0.54 (0.54-0.55)	10.8 (9.5-12.1)	**98.1 (97.7-98.4)**	68.5 (63.4-73.3)	73.9 (72.9-74.9)
**Loss of smell**	**0.56 (0.55-0.56)**	13.1 (11.7-14.6)	**98.1 (97.7-98.4)**	**72.9 (68.3-77.2)**	74.4 (73.4-75.4)
**Fever**	0.54 (0.52-0.55)	45.8 (43.8-47.9)	61.4 (60.1-62.7)	31.6 (30.0-33.2)	74.5 (73.2-75.7)

The best predictive performance measures (AUROC, sensitivity, specificity, PPV and NPV values) for each outcome are highlighted in bold

Table 7: Predictive value of individual clinical symptoms for predicting COVID-19 positivity in adults (n=13,274)

Variable	ROC area (95% CI)	Sensitivity (%) (95%CI)	Specificity (%) (95%CI)	PPV (%) (95% CI)	NPV (%) (95% CI)
Cough	**0.57 (0.56-0.58)**	**54.3 (52.9-55.8)**	58.9 (57.9-60.0)	41.3 (40.1-42.6)	**70.8 (69.7-71.8)**
Runny nose	0.53 (0.52-0.54)	26.1 (24.9-27.4)	79.3 (78.5-80.2)	40.3 (38.5-42.0)	66.9 (65.9-67.8)
Chest pain	0.51 (0.50-0.51)	3.9 (3.3-4.5)	97.3 (96.9-97.6)	43.2 (38.4-48.1)	65.5 (64.7-66.3)
Fatigue	0.51 (0.50-0.51)	6.5 (5.8-7.2)	95.0 (94.5-95.4)	40.6 (37.0-44.3)	65.6 (64.8-66.4)
Headache	0.52 (0.51-0.52)	13.9 (13.0-15.0)	89.1 (88.5-89.8)	40.6 (38.2-43.1)	66.1 (65.2-66.9)
Loss of taste	0.54 (0.53-0.54)	9.2 (8.3-10.0)	98.1 (97.8-98.4)	71.9 (68.1-75.5)	67.0 (66.1-67.8)
Loss of smell	0.55 (0.54-0.55)	10.7 (9.8-11.6)	**98.5 (98.2-98.8)**	**79.4 (76.0-82.5)**	67.4 (66.6-68.3)
Fever	**0.57 (0.56-0.58)**	51.2 (49.8-52.7)	63.1 (62.0-64.1)	42.5 (41.2-43.8)	**70.8 (69.8-71.8)**

For peer review only - http://bmjopen.bmj.com/site/about/guidelines.xhtml

Table 8: Predictive value of individual clinical symptoms for predicting COVID-19 positivity in the elderly (n=393)

Variable	ROC area (95% CI)	Sensitivity (%) (95%CI)	Specificity (%) (95%CI)	PPV (%) (95% CI)	NPV (%) (95% CI)
Cough	0.52 (0.47-0.57)	**61.4 (53.3-69.0)**	42.6 (36.1-49.1)	41.8 (35.4-48.4)	**62.1 (54.1-69.6)**
Breathing difficulty	**0.53 (0.48-0.58)**	38.0 (30.4-46.0)	68.1 (61.7-74.0)	44.4 (35.9-53.2)	62.0 (55.8-68.0)
Loss of smell	0.51 (0.49-0.52)	1.9 (0.4-5.4)	**99.1 (97.0-99.9)**	**60.0 (14.7-94.7)**	60.1 (55.0-65.0)

Table 9: Predictive performance of un-weighted clinical score thresholds for predicting COVID-19 positivity in children, adults and elderly

Outcome	Score	ROC area (95% CI)	Sensitivity (%) (95% CI)	Specificity (%) (95% CI)	PPV (%) (95% CI)	NPV (%) (95% CI)
**Children (<17 years) n=8,077**						
Number of symptoms	≥1	0.55 (0.54-0.56)	**86.1 (84.6-87.5)**	23.6 (22.5-24.7)	30.4 (29.3-31.6)	**81.3 (79.4-83.2)**
	≥2	**0.58 (0.57-0.59)**	43.3 (41.2-45.3)	72.9 (71.7-74.0)	38.3 (36.4-40.2)	76.8 (75.6-77.9)
	≥3	0.55 (0.54-0.56)	16.6 (15.1-18.2)	93.4 (92.7-94.0)	49.4 (45.8-53.0)	74.2 (73.2-75.2)
	≥4	0.52 (0.52-0.53)	5.1 (4.2-6.1)	**99.5 (99.3-99.7)**	**79.3 (71.8-85.6)**	73.0 (72.0-73.9)
**Adults (17-64 years) n=13,274**						
Number of symptoms	≥1	0.56 (0.55-0.56)	**89.7 (88.8-90.6)**	21.7 (20.9-22.6)	37.9 (37.0-38.8)	**79.9 (78.2-81.5)**
	≥2	**0.61 (0.60-0.61)**	51.0 (49.5-52.4)	70.3 (69.3-71.2)	47.7 (46.3-49.1)	72.9 (72.0-73.9)
	≥3	0.56 (0.55-0.57)	22.3 (21.1-23.5)	90.0 (89.4-90.6)	54.3 (52.1-56.6)	68.5 (67.7-69.4)
	≥4	0.53 (0.53-0.54)	8.3 (7.5-9.1)	**98.0 (97.7-98.3)**	**68.8 (64.7-72.6)**	66.7 (65.9-67.6)
**Elderly (>65 years) n=393**						
Number of symptoms	≥1	0.52 (0.48-0.57)	**75.3 (67.5-81.8)**	29.4 (23.6-35.6)	41.8 (36.0-47.7)	**63.9 (54.1-72.9)**
	≥2	**0.53 (0.49-0.57)**	25.9 (19.3-33.5)	**80.4 (74.8-85.3)**	**47.1 (36.3-58.1)**	61.8 (56.1-67.2)

The best predictive performance measures (AUROC, sensitivity, specificity, PPV and NPV values) for each age group are highlighted in bold

For peer review only - http://bmjopen.bmj.com/site/about/guidelines.xhtml

Table 10: Predictive performance of statistically-derived weighted clinical score thresholds for predicting COVID-19 in children, adults and elderly						
**Outcome**	**Score**	**ROC area (95% CI)**	**Sensitivity (%) (95% CI)**	**Specificity (%) (95% CI)**	**PPV (%) (95% CI)**	**NPV (%) (95% CI)**
**Children (<17 years) n=8,077**						
**Number of symptoms**	≥1	0.55 (0.54-0.56)	**86.1 (84.6-87.5)**	23.6 (22.5-24.7)	30.4 (29.3-31.6)	**81.3 (79.4-83.2)**
	≥2	0.55 (0.54-0.56)	**86.1 (84.6-87.5)**	23.6 (22.5-24.7)	30.4 (29.3-31.6)	**81.3 (79.4-83.2)**
	≥3	0.55 (0.54-0.57)	70.7 (68.8-72.6)	40.3 (39.5-41.5)	31.5 (30.2-32.8)	78.0 (76.4-79.4)
	≥4	**0.59 (0.58-0.60)**	48.1 (46.0-50.2)	**69.4 (68.2-70.6)**	**37.9 (36.1-39.7)**	77.5 (76.3-78.6)
**Adults (17-64 years) n=13,274**						
**Number of symptoms**	≥1	0.56 (0.55-0.56)	**89.7 (88.8-90.6)**	21.7 (20.9-22.6)	37.9 (37.0-38.8)	**79.9 (78.2-81.5)**
	≥2	0.58 (0.57-0.58)	85.6 (84.6-86.6)	30.0 (29.0-30.9)	39.4 (38.5-40.4)	79.6 (78.2-81.0)
	≥3	0.58 (0.57-0.59)	82.9 (81.8-84.0)	33.4 (32.4-34.4)	39.8 (38.9-40.8)	78.6 (77.2-79.9)
	≥4	**0.59 (0.58-0.59)**	80.8 (79.6-81.9)	**36.4 (35.4-37.5)**	**40.4 (39.4-41.4)**	78.1 (76.8-79.3)
**Elderly (>65 years) n=393**						
**Number of symptoms**	≥1	0.52 (0.48-0.57)	75.3 (67.8-81.8)	29.4 (23.6-35.6)	41.8 (36.0-47.7)	63.9 (54.1-72.9)
	≥2	0.52 (0.48-0.57)	75.3 (67.8-81.8)	29.4 (23.6-35.6)	41.8 (36.0-47.7)	63.9 (54.1-72.9)

The best predictive performance measures (AUROC, sensitivity, specificity, PPV and NPV values) for each age group are highlighted in bold

only

For peer review only - http://bmjopen.bmj.com/site/about/guidelines.xhtml

Table 11: Predictive performance of clinically-derived weighted clinical score thresholds for predicting COVID-19 in children, adults and elderly

Outcome	Score	ROC area (95% CI)	Sensitivity (%) (95% CI)	Specificity (%) (95% CI)	PPV (%) (95% CI)	NPV (%) (95% CI)
**Children (<17 years) n=8,077**						
Number of symptoms	≥1	0.55 (0.54-0.56)	**86.1 (84.6-87.5)**	23.6 (22.5-24.7)	30.4 (29.3-31.6)	**81.3 (79.4-83.2)**
	≥2	0.54 (0.53-0.55)	81.2 (79.6-82.8)	27.1 (25.9-28.2)	30.2 (29.0-31.4)	78.8 (76.9-80.6)
	≥3	0.56 (0.55-0.57)	74.2 (72.4-76.0)	37.7 (36.5-39.0)	31.7 (30.4-32.9)	79.0 (77.5-80.5)
	≥4	**0.57 (0.56-0.58)**	40.7 (38.6-42.7)	**73.6 (72.4-74.7)**	**37.4 (35.5-39.4)**	76.1 (75.0-77.2)
**Adults (17-64 years) n=13,274**						
Number of symptoms	≥1	0.56 (0.55-0.56)	**89.7 (88.8-90.6)**	21.7 (20.9-22.6)	37.9 (37.0-38.8)	**79.9 (78.2-81.5)**
	≥2	0.56 (0.55-0.56)	**89.7 (88.8-90.6)**	21.7 (20.9-22.6)	37.9 (37.0-38.8)	**79.9 (78.2-81.5)**
	≥3	**0.58 (0.58-0.59)**	82.5 (81.3-83.5)	34.1 (33.1-35.1)	40.0 (39.0-41.0)	78.5 (77.1-79.8)
	≥4	**0.58 (0.58-0.59)**	79.0 (77.8-80.2)	**37.9 (36.9-39.0**	**40.4 (39.4-41.4)**	77.2 (75.9-78.5)
**Elderly (>65 years) n=393**						
Number of symptoms	≥1	0.52 (0.48-0.57)	75.3 (67.8-81.8)	29.4 (23.6-35.6)	41.8 (36.0-47.7)	63.9 (54.1-72.9)
	≥2	0.52 (0.48-0.57)	75.3 (67.8-81.8)	29.4 (23.6-35.6)	41.8 (36.0-47.7)	63.9 (54.1-72.9)

The best predictive performance measures (AUROC, sensitivity, specificity, PPV and NPV values) for each age group are highlighted in bold

Supplementary Table 12: Results from the Hosmer-Lemeshow goodness-of-fit test

Age group	Unweighted combined scores	Weighted combined scores (beta-regression coefficients)	Weighted combined scores (clinicians' weights)
	P-values	P-values	P-values
**Children**	**0.2160**	0.0066	<0.0001
**Adults**	0.0001	<0.0001	<0.0001
**Elderly**	**0.6703**	**0.9842**	**0.9054**

For peer review only - http://bmjopen.bmj.com/site/about/guidelines.xhtml

TRIPOD Checklist: Prediction Model Development and Validation

Section/Topic	Item		Checklist Item	Page
**Title and abstract**				
Title	1	D;V	Identify the study as developing and/or validating a multivariable prediction model, the target population, and the outcome to be predicted.	Page 1
Abstract	2	D;V	Provide a summary of objectives, study design, setting, participants, sample size, predictors, outcome, statistical analysis, results, and conclusions.	Page 2; lines 43-69
**Introduction**				
Background and objectives	3a	D;V	Explain the medical context (including whether diagnostic or prognostic) and rationale for developing or validating the multivariable prediction model, including references to existing models.	Page 4; lines 105-130
	3b	D;V	Specify the objectives, including whether the study describes the development or validation of the model or both.	Page 5; lines 138-141
**Methods**				
Source of data	4a	D;V	Describe the study design or source of data (e.g., randomized trial, cohort, or registry data), separately for the development and validation data sets, if applicable.	Page 5; lines 143-149
	4b	D;V	Specify the key study dates, including start of accrual; end of accrual; and, if applicable, end of follow-up.	Page 5; lines 145-146
Participants	5a	D;V	Specify key elements of the study setting (e.g., primary care, secondary care, general population) including number and location of centres.	Page 5; lines 146-147
	5b	D;V	Describe eligibility criteria for participants.	Page 5; lines 152-154
	5c	D;V	Give details of treatments received, if relevant.	Not relevant
Outcome	6a	D;V	Clearly define the outcome that is predicted by the prediction model, including how and when assessed.	Page 6; lines 188-192
	6b	D;V	Report any actions to blind assessment of the outcome to be predicted.	Not relevant, the study being an analysis of a secondary data
Predictors	7a	D;V	Clearly define all predictors used in developing or validating the multivariable prediction model, including how and when they were measured.	Page 6; lines 192-200
	7b	D;V	Report any actions to blind assessment of predictors for the outcome and other predictors.	Not relevant, the study being an analysis of a secondary data
Sample size	8	D;V	Explain how the study size was arrived at.	Page 6; lines 169-172
Missing data	9	D;V	Describe how missing data were handled (e.g., complete-case analysis, single imputation, multiple imputation) with details of any imputation method.	Not relevant given the assumptions made prior to analyses—see page 6, lines 174-187
Statistical analysis methods	10a	D	Describe how predictors were handled in the analyses.	Page 6; lines 175-187
	10b	D	Specify type of model, all model-building procedures (including any predictor selection), and method for internal validation.	Pages 7-8; lines 206-256
	10c	V	For validation, describe how the predictions were calculated.	Page 8; lines 245-250
	10d	D;V	Specify all measures used to assess model performance and, if relevant, to compare multiple models.	Page 8; lines 245-250
	10e	V	Describe any model updating (e.g., recalibration) arising from the validation, if done.	Not done
Risk groups	11	D;V	Provide details on how risk groups were created, if done.	Not done
Development vs. validation	12	V	For validation, identify any differences from the development data in setting, eligibility criteria, outcome, and predictors.	Not relevant
**Results**				
Participants	13a	D;V	Describe the flow of participants through the study, including the number of participants with and without the outcome and, if applicable, a summary of the follow-up time. A diagram may be helpful.	Page 6; line 189
	13b	D;V	Describe the characteristics of the participants (basic demographics, clinical features, available predictors), including the number of participants with missing data for predictors and outcome.	Page 9; lines 267-276
	13c	V	For validation, show a comparison with the development data of the distribution of important variables (demographics, predictors and outcome).	Page 9; lines 275-276
Model development	14a	D	Specify the number of participants and outcome events in each analysis.	Page 9
	14b	D	If done, report the unadjusted association between each candidate predictor and outcome.	Page 13; lines 277-283
Model specification	15a	D	Present the full prediction model to allow predictions for individuals (i.e., all regression coefficients, and model intercept or baseline survival at a given time point).	Not done
	15b	D	Explain how to the use the prediction model.	Page 8; lines 246-250

For peer review only - http://bmjopen.bmj.com/site/about/guidelines.xhtml

TRIPOD Checklist: Prediction Model Development and Validation

Model performance	16	D;V	Report performance measures (with CIs) for the prediction model.	Pages 13-18; lines 284-288; lines 316-321; lines 345-350
Model-updating	17	V	If done, report the results from any model updating (i.e., model specification, model performance).	Not relevant
**Discussion**				
Limitations	18	D;V	Discuss any limitations of the study (such as nonrepresentative sample, few events per predictor, missing data).	Page 21; lines 413-426
Interpretation	19a	V	For validation, discuss the results with reference to performance in the development data, and any other validation data.	Page 21-23; lines 443-461 & 485-493
	19b	D;V	Give an overall interpretation of the results, considering objectives, limitations, results from similar studies, and other relevant evidence.	Page 21-23; lines 427-493
Implications	20	D;V	Discuss the potential clinical use of the model and implications for future research.	Page 21-23; lines 427-493
**Other information**				
Supplementary information	21	D;V	Provide information about the availability of supplementary resources, such as study protocol, Web calculator, and data sets.	Page 27; lines 634-636
Funding	22	D;V	Give the source of funding and the role of the funders for the present study.	Page 27; lines 631-633

*Items relevant only to the development of a prediction model are denoted by D, items relating solely to a validation of a prediction model are denoted by V, and items relating to both are denoted D;V. We recommend using the TRIPOD Checklist in conjunction with the TRIPOD Explanation and Elaboration document.

For peer review only

BMJ Open

Symptom scores have poor predictive capacity for COVID-19 positivity: national derivation and validation cohort study in Nigeria

Journal:	*BMJ Open*
Manuscript ID	bmjopen-2021-049699.R1
Article Type:	Original research
Date Submitted by the Author:	19-Jun-2021
Complete List of Authors:	Elimian, Kelly; Nigeria Centre for Disease Control; Karolinska Institutet, Department of Global Public Health<br>Aderinola, Olaolu; Nigeria Centre for Disease Control<br>Gibson, Jack; University of Nottingham, Department of Epidemiology and Public Health<br>Myles, Puja; University of Nottingham Faculty of Medicine and Health Sciences, Epidemiology & Public Health<br>Ochu, Chinwe; Nigeria Centre for Disease Control<br>King, Carina; Karolinska Institute, Department of Global Public Health<br>Okwor, Tochi; Nigeria Centre for Disease Control, Prevention Programmes and Knowledge Management<br>Gaudenzi, Giulia; Karolinska Institutet, Department of Global Public Health<br>Olayinka, Adebola; World Health Organization<br>Zaiyad, Habib; University of Abuja Teaching Hospital<br>Ohonsi, Cornelius; Nigeria Centre for Disease Control<br>Ebhodaghe, Blessing; Nigeria Centre for Disease Control<br>Ipadeola, Oladipupo ; University of Ilorin, Kwara State, Nigeria<br>Dan-Nwafor, Chioma; Nigeria Centre for Disease Control<br>Nwachukwu , William ; Nigeria Centre for Disease Control<br>Abdus-salam, Ismail; Lagos State Ministry of Health<br>Akande, Oluwatosin; University of Ilorin Teaching Hospital, Department of Epidemiology and Community Health<br>Falodun, Olanrewaju; National Hospital, Department of Internal Medicine<br>Arinze, Chinedu; Nigeria Centre for Disease Control<br>Ezeokafor, Chidiebere; National Agency for the Control of AIDS<br>Jafiya, Abubakar; Nigeria Centre for Disease Control<br>Ojimba, Anastacia; Federal Medical Centre Asaba<br>Aremu, John; Federal Teaching Hospital Gombe<br>Joseph, Emmanuel; Kaduna State Infectious Disease Control Center Community Medicine<br>Bowale, Abimbola; Mainland Hospital Yaba<br>Mutiu, Babatunde; Lagos State Government Ministry of Health<br>Saka, Babatunde; Lagos State Government Ministry of Health<br>Jinadu, Arisekola; Nigeria Centre for Disease Control<br>Hamza, Khadeejah; Ahmadu Bello University, Department of Community Medicine<br>Ibeh, Christian; Nnamdi Azikiwe University Teaching Hospital, Department of Community Medicine

	Bello, Shaibu; Usmanu Danfodiyo University, Department of Medical Education<br>Asuzu, Michael; University College Hospital, Department of Community Medicine<br>Mba, Nwando; Nigeria Centre for Disease Control<br>Oladejo, John; Nigeria Centre for Disease Control<br>Ilori, Elsie; Nigeria Centre for Disease Control<br>Alfvén, Tobias ; Karolinska Institutet, Department of Global Public Health<br>Igumbor, Ehimario; Nigeria Centre for Disease Control; University of the Western Cape, School of Public Health<br>Ihekweazu, Chikwe; Nigeria Centre for Disease Control
**Primary Subject Heading**:	Epidemiology
Secondary Subject Heading:	Global health, Infectious diseases, Public health
Keywords:	COVID-19, Public health < INFECTIOUS DISEASES, Epidemiology < TROPICAL MEDICINE

SCHOLARONE™
Manuscripts

I, the Submitting Author has the right to grant and does grant on behalf of all authors of the Work (as defined in the below author licence), an exclusive licence and/or a non-exclusive licence for contributions from authors who are: i) UK Crown employees; ii) where BMJ has agreed a CC-BY licence shall apply, and/or iii) in accordance with the terms applicable for US Federal Government officers or employees acting as part of their official duties; on a worldwide, perpetual, irrevocable, royalty-free basis to BMJ Publishing Group Ltd ("BMJ") its licensees and where the relevant Journal is co-owned by BMJ to the co-owners of the Journal, to publish the Work in this journal and any other BMJ products and to exploit all rights, as set out in our licence.

The Submitting Author accepts and understands that any supply made under these terms is made by BMJ to the Submitting Author unless you are acting as an employee on behalf of your employer or a postgraduate student of an affiliated institution which is paying any applicable article publishing charge ("APC") for Open Access articles. Where the Submitting Author wishes to make the Work available on an Open Access basis (and intends to pay the relevant APC), the terms of reuse of such Open Access shall be governed by a Creative Commons licence – details of these licences and which Creative Commons licence will apply to this Work are set out in our licence referred to above.

Other than as permitted in any relevant BMJ Author's Self Archiving Policies, I confirm this Work has not been accepted for publication elsewhere, is not being considered for publication elsewhere and does not duplicate material already published. I confirm all authors consent to publication of this Work and authorise the granting of this licence.

For peer review only

Symptom scores have poor predictive capacity for COVID-19 positivity: national derivation and validation cohort study in Nigeria

Kelly Elimian[1,2,3,4], Olaolu Aderinola[1], Jack Gibson[5], Puja Myles[5], Chinwe Ochu[1‡], Carina King[2], Tochi Okwor[1], Giulia Gaudenzi[2], Adebola Olayinka[6], Habib Zaiyad[7], Cornelius Ohonsi[1], Blessing Ebhodaghe[1], Oladipupo Ipadeola[8], Chioma Dan-Nwafor[1], William Nwachukwu[1], Ismail Abdus-Salam[9], Oluwatosin Akande[10], Olanrewaju Falodun[11], Chinedu Arinze[1], Chidiebere Ezeokafor[12], Jafiya Abubakar[1], Anastacia Ojimba[13], John Aremu[14], Emmanuel Joseph[15], Abimbola Bowale[16], Bamidele Mutiu[9], Babatunde Akeem Saka[9], Arisekola Jinadu[1], Khadeejah Hamza[17], Christian Ibeh[18], Shaibu Oricha Bello[19], Michael Asuzu[20], Nwando Mba[1], John Oladejo[1], Elsie Ilori[1], Tobias Alfven[2], Ehimario Igumbor[1], Chikwe Ihekweazu[1]

[1]Nigeria Centre for Disease Control, Abuja, Nigeria
[2]Department of Global Public Health, Karolinska Institutet, Stockholm, Sweden
[3]Department of Microbiology, University of Benin, Edo State, Nigeria
[4]Nigeria COVID-19 Research Consortium, Abuja, Nigeria
[5]Department of Epidemiology and Public Health, University of Nottingham, UK
[6]World Health Organization, Abuja, Nigeria
[7]University of Abuja Teaching Hospital, Nigeria
[8]University of Ilorin, Kwara State, Nigeria
[9]Ministry of Health, Lagos State, Nigeria
[10]University of Ilorin Teaching Hospital, Kwara State, Nigeria
[11]Department of Internal Medicine, National Hospital, Abuja, Nigeria
[12]National Agency for the Control of AIDS, Abuja, Nigeria
[13]Federal Medical Centre, Asaba, Delta State, Nigeria
[14]Federal Teaching Hospital Gombe, Gombe State, Nigeria
[15]Kaduna State Infectious Disease Control Center, Community Medicine, Kaduna State, Nigeria
[16]Mainland Hospital Yaba, Lagos State, Nigeria
[17]Department of Community Medicine, Ahmadu Bello University, Kaduna State, Nigeria
[18]Department of Community Medicine, Nnamdi Azikiwe University Teaching Hospital, Nigeria
[19]College of Health Sciences, Usmanu Danfodiyo University, Sokoto State, Nigeria
[20]Department of Community Medicine, University College Hospital, Oyo State, Nigeria.

‡Corresponding author and email address
Dr Kelly Elimian
kellyelimian@gmail.com

For peer review only - http://bmjopen.bmj.com/site/about/guidelines.xhtml

Abstract

Objectives: This study aimed to develop and validate a symptom prediction tool for COVID-19 test positivity in Nigeria.

Design: Predictive modelling study.

Setting: All Nigeria States and the Federal Capital Territory.

Participants: A cohort of 43,221 individuals within the national COVID-19 surveillance dataset from February 27 to August 27, 2020.

Methods: Complete dataset was randomly split into two equal halves: derivation and validation datasets. Using the derivation dataset (n=21,477), backward multivariable logistic regression approach was used to identify symptoms positively associated with COVID-19 positivity (by RT-PCR) in children (≤17 years), adults (18-64 years) and elderly (≥65 years) patients separately. Weighted statistical and clinical scores were developed using beta regression coefficients and clinicians' judgements, respectively. Using the validation dataset (n=21,744), the predictive capacity of individual symptoms, un-weighted score and the two weighted scores was assessed using Area Under the Receiver Operating Characteristic curve (AUROC) values.

Results: Overall, 27.6% of children (4,415/15,988), 34.6% of adults (9,154/26,441), and 40.0% of elderly (317/792) that had been tested were positive for COVID-19. Best individual symptom predictor of COVID-19 positivity was loss of smell in children (AUROC 0.56, 95% CI: 0.55-0.56), either fever or cough in adults (AUROC 0.57, 95% CI: 0.56-0.58), and difficulty in breathing in the elderly (AUROC 0.53, 0.48-0.58) patients. In children, adults, and the elderly patients all scoring approaches showed similar predictive performance.

Conclusions: The predictive capacity of various symptom scores for COVID-19 positivity was poor overall; however, the findings have the potential to serve as an advocacy tool for more investments in resources for capacity strengthening of molecular testing for COVID-19 in Nigeria.

Keywords

Diagnosis; Symptoms; COVID-19; Predictive; Nigeria

For peer review only - http://bmjopen.bmj.com/site/about/guidelines.xhtml

Strengths and limitations of this study

- This study provides the early evidence on the development and validation of symptom prediction scores for COVID-19 positivity in a sub-Saharan African country like Nigeria.
- The study adopted a participatory approach in the derivation of clinically weighted score by engaging clinicians managing COVID-19 patients, a practice that can enhance the clinical relevance of the findings.
- The study has provided evidential-basis for advocacy for more investments in capacity strengthening of molecular testing for COVID-19 in Nigeria and similar settings.
- Splitting the dataset for both derivation and validation of symptom scores in the present study might have underestimated the predictive performance of our model due to loss of power.
- The study lacked sufficient clinical laboratory data (e.g. albumin or albumin/globin, direct bilirubin values) that have been found to be significant variables in COVID-19 diagnostic models.

Introduction

The index case of COVID-19 in Nigeria was recorded on February 27, 2020, in Ogun State; one of the contacts of the index case was later diagnosed with the disease in the same state. As of epidemiological week 44 (October 26–November 1), Nigeria had recorded 62,964 confirmed cases, of whom 1,146 died and 58,790 recovered, resulting in a case fatality rate of 1.8%.[1] The rapid increase in COVID-19 cases in Nigeria caused a strain on the healthcare systems, particularly in the area of molecular testing using real-time polymerase chain reaction (RT-PCR). Deficiencies in molecular laboratory capacity can hinder the rapid identification of persons with COVID-19 infection and initiation of appropriate treatment and public health measures.[2] For example, as of April 9, 2020, only 5,000 tests for Severe Acute Respiratory Syndrome Coronavirus 2 (SARS-CoV-2) were performed across Nigeria by eight laboratories across six States.[3] Concerted efforts to expand molecular testing capacity across the country have since been made, with 673,183 tests for SARS-CoV-2 conducted and the Nigeria Centre for Disease Control (NCDC) COVID-19 laboratory networks expanded to 69 functioning laboratories as of November 6, 2020.[4] However, with a population of over 200 million and community transmission of COVID-19, this testing rate for Nigeria is still well below the Africa CDC-set target of 1% for population. Generally, laboratory capacity to test for SARS-

For peer review only - http://bmjopen.bmj.com/site/about/guidelines.xhtml

CoV-2 remains a challenge in many sub-Saharan African countries, especially in the early phase of COVID-19 pandemic.[5]

Although PCR testing is considered the gold-standard for COVID-19 diagnosis, limited access and centralised laboratory systems often contribute to delay.[6] This has contributed to making diagnostic results unavailable for urgent decision-making by frontline healthcare workers, with consequent effect on healthcare delivery and increased risk of nosocomial infection.[7] Between February 27 and June 6, 2020, for example, the median turn-around time for laboratory diagnosis of COVID-19 in Nigeria was 2 (IQR: 1-4) days; 9% of the 12,289 confirmed COVID-19 cases during this period was recorded among healthcare workers.[8] Thus, to mitigate the impact of COVID-19 on the healthcare system, while also improving community surveillance and care for patients, rapid and accurate diagnosis are crucial.[9] NCDC and its technical partners are currently conducting a study to validate rapid diagnostic test kits to determine their accuracy and clinical utility in Nigeria.

One approach to supplement laboratory testing is however the use of diagnostic prediction models that utilise clinical variables (including standard blood counts when available) to estimate the likelihood of individuals being COVID-19 positive or experiencing poor clinical outcome.[9] A systematic review of developed and/or validated prediction models found the most frequently reported predictors of COVID-19 diagnosis to be flu-like symptoms (e.g. chills, fatigue) and neutrophil count, with c-statistic estimates ≥0.90 (an excellent discriminative performance).[9] However, to date, these prediction models have been judged to be at a high risk of bias, largely because they were fitted on data that were not representative of the target population, and have suffered from poor reporting on intended use of the models or their calibration performance. While the reported performances of models to date are encouraging, their adoption in clinical practice have not been recommended.[9] In addition, there has been a death of evidence from sub-Saharan Africa as the majority of available models use data from China and European countries, thus limiting their generalisability to settings such as Nigeria with different COVID-19 epidemiology, health care systems, socioeconomic conditions, and health-seeking behaviours.

The majority of confirmed COVID-19 cases in Nigeria are asymptomatic at the point of diagnosis[8]; for the confirmed cases who present with symptoms the most common symptoms tend to be non-specific (e.g. fever, cough and difficulty in breathing).[8,10,11] Using data from February 27 to June 6, 2020, presentation with cough, fever, loss of smell and loss of taste was

For peer review only - http://bmjopen.bmj.com/site/about/guidelines.xhtml

found to be positively and independently associated with COVID-19 positivity in Nigeria[12]. But the dearth of evidence on the predictive capacity of clinical symptoms to predict COVID-19 in Nigeria (and indeed in sub-Saharan Africa) is the rationale for this study. Thus, we aimed to develop and validate the predictive capacity of clinical signs and symptoms with regards to testing positive for COVID-19 and to investigate whether there are any gains in the predictive capacity of statistically- and clinically-derived weighted combined symptom scores, as compared to an un-weighted combined score in Nigeria.

Methods

Study design and data source

This is a predictive modelling study using a retrospective cohort of persons enrolled in the Surveillance, Outbreak Response Management and Analysis System (SORMAS) database from February 27 to August 27, 2020. A detailed description of SORMAS in the COVID-19 context in Nigeria is available elsewhere.[8] Briefly, SORMAS is an open-source real-time electronic health surveillance and laboratory database, which has been in use in Nigeria since 2017. SORMAS is hosted and coordinated at the NCDC's headquarter in Abuja.

Study participants

The study population comprised children (≤17 years), adults (18-64 years) and the elderly (≥65 years) who were tested for SARS-CoV-2 infection. Eligibility for RT-PCR test during the study period was based on the NCDC COVID-19 standard case definition for suspected cases (Supplementary Table 1).[13] However, provisions were made to test persons concerned about COVID-19 infection upon presentation to designated testing centres regardless of whether they met the NCDC case definitions or not.

Data collection and management

Sociodemographic (e.g. age, sex, occupation, education and geopolitical zone of residence) and clinical presentation (signs and symptoms in the 14 days prior to testing) were collected from all persons tested. The collection and transportation of respiratory samples (oropharyngeal and nasopharyngeal swabs) for laboratory analysis were facilitated by trained healthcare workers according to NCDC guidelines.[14] Testing for SARS-CoV-2 by RT-PCR was performed according with the WHO guidelines.[15] All the collected data across the country, including laboratory diagnostic outcomes, were submitted by trained healthcare workers to NCDC via the SORMAS application installed on tablets or laptops. Patients who tested positive for

For peer review only - http://bmjopen.bmj.com/site/about/guidelines.xhtml

COVID-19 were managed either in a government health facility or at home, depending on the illness severity or availability of bed space at health facilities, while observing the NCDC case management guidelines.[16]

Since this was an analysis of a secondary dataset, there was no formal sample size calculation for this study; however, the study met the standard sample size requirement of 10 outcome events per degree of freedom in prediction models (e.g., 10 binary variables in the model require 100 COVID-19 positive cases). We used the train-test split-sample method to randomly split the complete dataset into two (i.e. training and testing datasets), assigning half of the records to either the training (derivation) or testing (validation) datasets based on a random number generated within the statistical software. However, we made four major assumptions to define the study eligibility criteria. First, we assumed that a missing variable was indicative of absence (i.e., record not present), supported by findings ($p<0.001$) from a chi-square test of association between two *ad hoc* variables 'any-missing' and 'any-absent'. Second, we decided that eligible study participants needed to have complete records for both age and sex since these are crucial demographic variables. Third, given the high proportion of asymptomatic COVID-19 cases in Nigeria,[8] the study participants needed to have had at least one symptom positively recorded (i.e. symptomatic). Lastly, analyses were performed separately for children, adults and the elderly, given the evidence supporting the age-dependence of COVID-19 symptom presentation, with children more likely to be asymptomatic than adults.[17,18] Figure 1 shows a flowchart showing the processes for selecting the study population as stated in the eligibility criteria (definitions of suspected and probable cases are available in the NCDC case definition [13]). All symptoms were coded as '1' if present and '0' if not. Sex was classified as a binary variable, with '1' and '0' indicative of male and female gender, respectively.

Figure 1 about here

Outcome and predictor variables

The outcome was COVID-19 positivity, defined as either presence or absence of SARS-CoV-2 by RT-PCR confirmation (yes/no). Clinical prediction variables were informed by evidence from our previous study using the same dataset, though it covered a shorter period (February 27 to June 5, 2020).[12] The clinical variables were collected using a combination of self-reports by COVID-19 suspected cases and/or their caretakers as well as by objective assessment by a healthcare worker. The three categories of clinical signs and symptoms (each coded yes/no) with their respective examples include: 1. "obvious or as observed by a healthcare worker"

For peer review only - http://bmjopen.bmj.com/site/about/guidelines.xhtml

(chills/sweat, cough, breathing difficulty, rapid breathing and runny nose); 2. "elicitable or information volunteered or given upon inquiry by patients/relatives" (abdominal pain/diarrhoea, gastrointestinal tract symptoms, chest pain, fatigue/weakness, headache, musculoskeletal pain, sore throat, loss of taste, and loss of smell); 3. and "measurable as assessed by a healthcare worker" (fever, defined as an axillary temperature ≥37.5°C).

Statistical analyses

The data were set up and analysed using Stata 13.0 (Stata Corporation, College Station, TX, USA).

Model development

Using the derivation dataset, associations between individual symptoms and COVID-19 positivity was investigated, in turn, using univariable logistic regression. Analyses were stratified by children, adults, and the elderly. The results are presented as unadjusted odds ratios (ORs) and 95% confidence intervals (95% CI). To determine which of the symptoms were independently associated with COVID-19 positivity, we first conducted a test for multicollinearity by the various age groups (children, adults, and the elderly) using the variance inflation factor (VIF) and tolerance values of each statistically significant symptom identified from the univariable logistic regression model (see Supplementary Table 2a, 2b and 2c for results). This was to ensure that symptoms were independent of each other or not collinear and therefore suitable for combining into a single score. Both VIF and tolerance values measure how much the regression coefficient for a symptom is determined by the other symptoms in the model; low VIF and high tolerance values suggest the absence of multicollinearity and vice-versa.[19] Thereafter we used the backward multivariable logistic regression approach to select all the statistically significant symptoms for the model. As before, analyses were conducted for children, adults, and the elderly separately.

We then created three different symptom scores. First, an un-weighted combined score was calculated for each person (separately for children, adults, and the elderly) by allocating '1' point for each symptom before summation. The un-weighted combined scores were included in the model as a continuous variable. The combined scores were further recoded as binary categorical variables to represent clinical scores≥1 (yes/no), ≥2 (yes/no), ≥3 (yes/no), and ≥4 (yes/no). Additionally, weighted combined scores were developed using two approaches. First, we used the beta regression coefficients obtained from the multivariable logistic regression model that was run previously. Each symptom was given the value of '1' if present and '0' if

For peer review only - http://bmjopen.bmj.com/site/about/guidelines.xhtml

otherwise. Each symptom was then multiplied by its weight, obtained by multiplying the respective beta regression coefficients (or log of the ORs) by 10. Essentially, the beta regression coefficients measure the relative prognostic strength of each symptom when they are included simultaneously in a multivariable regression model so that the bigger the value of a symptom, the more its weight. Second, we engaged six clinicians (including infectious disease consultants) who have been managing COVID-19 patients in treatment centres and at home in the FCT, Gombe, Delta, and Kaduna. Here, the clinicians were provided with a list of symptoms identified to be statistically significant from the univariable logistic models, with the freedom to add any missing sign or symptom in the model as needed. Each clinician was then asked to independently assign a weight of 1-5 for each symptom based on experiences from managing COVID-19 patients (see Supplementary Table 3 for detail of weighted scores assigned by each clinician for the identified symptoms). Similar to the statistical weighting approach, each symptom was multiplied by the average of the combined weights assigned by all the clinicians. Predictive capacity of the various score thresholds was also compared to see which combination of symptoms was more predictive of COVID-19 positivity.

Model validation

The different models were then applied to the validation dataset. Separately for children, adults and the elderly, predictive capacity was assessed in terms of sensitivity, specificity, Area under the Receiver Operating Characteristic (ROC) curve (AUROC) value, positive predictive value (PPV) and negative predictive value (NPV);[20] and ROC curves were plotted for the comparison of clinical prediction scores.

Where applicable, this study is reported according to the TRIPOD (Transparent Reporting of a multivariable prediction model for Individual Prognosis Or Diagnosis) statement.[21]

External calibration belt and test for model goodness-of-fit

Using the validation dataset, external calibration test and belt were used to evaluate the goodness-of-fit of our models, an approach that examines the relationship between estimated probabilities and observed outcome rates[22]. Tests and belts often return concordant outputs: non-significant tests are often associated with the belt encompassing the 45-degree lines (good fit) and significant tests with the belt deviating from the bisector (poor fit).

Ethics and ethical considerations

The Nigeria National Health Research Ethics Committee approved the use of the SORMAS database for this study (reference number: NHREC/01/01/2007-22/06/2020).

For peer review only - http://bmjopen.bmj.com/site/about/guidelines.xhtml

Patient and public involvement

Being an analysis of de-identified secondary dataset, it was not possible to involve patients or the public in the design, or conduct, or reporting, or dissemination plans of this study. However, clinicians who have been managing COVID-19 patients both in health facilities and at home in Nigeria were actively engaged both in planning the study and in developing the clinical scores.

Results

Description of study population

The baseline sociodemographic and clinical characteristics of the study participants in the combined datasets are presented in Table 1. Overall, 15,988 children met the study eligibility criteria, 4,415 (27.6%) of whom tested positive for COVID-19 and 62.2% were male. A total of 26,441 adults met the study eligibility criteria, 9,154 (34.6%) of whom tested positive for COVID-19, 64.0% (16,819/26,441) were male, and 11.3% (2,977/26,441) were healthcare workers. Seven hundred and ninety-two elderly patients met the study eligibility criteria, 317 (40.0%) of whom tested positive for COVID-19 and 62.3% were males. Similar characteristics were observed in the derivation (Supplementary Table 4) and validation (Supplementary Table 5) cohorts for all age-groups.

For peer review only - http://bmjopen.bmj.com/site/about/guidelines.xhtml

Table 1: Baseline sociodemographic and clinical characteristics of all the study participants in relation to COVID-19 infection (combined dataset)

Variable	Children (≤17 years)		Adults (18-64 years)		Elderly (≥65 years)	
	PCR-confirmed cases [n=4,415 (%)]	PCR positive and negative cases [N=15,988 (%)]	PCR-confirmed cases [n=9,154 (%)]	PCR positive and negative cases [N=26,441 (%)]	PCR-confirmed cases [n=317 (%)]	PCR positive and negative cases [N=792 (%)]
**Socio-demographic features**						
**Sex**						
Female	1,773 (40.16)	6,051 (37.85)	3,080 (33.65)	9,622 (36.39)	110 (34.70)	299 (37.75)
Male	2,642 (59.84)	9,937 (62.15)‡	6,074 (66.35)	16,819 (63.61)‡	207 (65.30)	493 (62.25)NS
**Geopolitical zone§**						
South-west	1,302 (29.49)	3,539 (22.14)	2,592 (28.32)	6,245 (23.62)	86 (27.13)	185 (23.36)
South-south	1,435 (32.50)	5,689 (35.58)	3,228 (35.26)	10,911 (41.27)	108 (34.07)	340 (42.93)
South-east	90 (2.04)	278 (1.74)	273 (2.98)	609 (2.30)	25 (7.89)	37 (4.67)
North-central	816 (18.48)	3,056 (19.11)	1,526 (16.67)	4,061 (15.36)	30 (9.46)	76 (9.60)
North-west	658 (14.90)	3,118 (19.50)	1,258 (13.72)	4,005 (15.15)	45 (14.20)	116 (14.65)
North-east	114 (2.58)	308 (1.93)‡	277 (3.03)	610 (2.31)‡	23 (7.26)	38 (4.80)‡
**Setting**						
Rural	230 (5.21)	887 (5.55)	511 (5.58)	1,562 (5.91)	35 (11.04)	74 (9.34)
Urban	2,048 (46.39)	6,943 (43.43)	4,487 (49.02)	13,088 (49.50)	183 (57.73)	423 (53.41)
Missing	2,137 (48.40)	8,158 (51.03)‡	4,156 (45.40)	11,791 (44.59)NS	99 (31.23)	295 (37.25)†
**Education**						
None	58 (1.31)	217 (1.36)	128 (1.40)	368 (1.39)	25 (7.89)	48 (6.06)
Nursery	30 (0.68)	143 (0.89)	3 (0.03)	7 (0.03)	0 (0.00)	0 (0.00)
Primary	130 (2.94)	520 (3.25)	102 (1.11)	322 (1.22)	8 (2.52)	18 (2.27)
Secondary	385 (8.72)	1,379 (8.63)	792 (8.65)	2,262 (8.55)	23 (7.26)	58 (7.32)
Tertiary	1,410 (31.94)	4,066 (25.43)	3,141 (34.31)	8,145 (30.80)	87 (27.44)	168 (21.21)
Other	179 (4.05)	1,065 (6.66)	261 (2.85)	688 (2.60)	20 (6.31)	45 (5.68)
Missing	2,223 (50.35)	8,598 (53.78)‡	4,727 (51.64)	14,649 (55.40)‡	154 (48.58)	455 (57.45)†
**Occupation**						
Student/pupil	599 (13.57)	2,809 (17.57)	434 (4.74)	1,516 (5.73)	1 (0.32)	2 (0.25)
Child/housewife	133 (3.01)	489 (3.06)	178 (1.94)	500 (1.89)	13 (4.10)	42 (5.30)
Business/trading	217 (4.92)	765 (4.78)	757 (8.27)	2,043 (7.73)	19 (5.99)	47 (5.93)
Transporter	11 (0.25)	80 (0.50)	45 (0.49)	174 (0.66)	0 (0.00)	3 (0.38)
Healthcare worker	601 (13.61)	1,953 (12.22)	973 (10.63)	2,977 (11.26)	7 (2.21)	20 (2.53)
Laboratorian	15 (0.34)	40 (0.25)	26 (0.28)	63 (0.24)	0 (0.00)	0 (0.00)
Farmer	36 (0.82)	192 (1.20)	173 (1.89)	589 (2.23)	24 (7.57)	47 (5.93)
Animal-related worker	8 (0.18)	28 (0.18)	25 (0.27)	80 (0.30)	1 (0.32)	9 (1.14)
Religious/traditional leader	5 (0.11)	24 (0.15)	53 (0.58)	129 (0.49)	6 (1.89)	9 (1.14)

For peer review only - http://bmjopen.bmj.com/site/about/guidelines.xhtml

Other	1,633 (36.99)	5,126 (32.06)	4,072 (44.48)	11,408 (43.15)	164 (51.74)	375 (47.35)
Missing	1,157 (26.21)	4,482 (28.03)‡	2,418 (26.41)	6,962 (26.33)‡	82 (25.87)	238 (30.05)NS
**Clinical signs and symptoms**						
**Clinical outcome**						
Recovered	2,847 (64.48)	4,443 (27.79)	5,694 (62.20)	7,313 (27.66)	150 (47.32)	184 (23.23)
Dead	36 (0.82)	51 (0.32)	485 (5.30)	549 (2.08)	83 (26.18)	84 (10.61)
No outcome yet	1,532 (34.70)	11,494 (71.89)‡	2,975 (32.50)	18,579 (70.27)‡	84 (26.50)	524 (66.16)‡
Obvious (visible to healthcare workers on sight)						
**Chills/sweat**						
No	4,355 (98.64)	15,764 (98.60)	9,009 (98.42)	26,029 (98.44)	315 (99.37)	783 (98.86)
Yes	60 (1.36)	224 (1.40)NS	145 (1.58)	412 (1.56)NS	2 (0.63)	9 (1.14)NS
**Cough**						
No	2,345 (53.11)	9,393 (58.75)	4,130 (45.12)	14,391 (54.43)	114 (35.96)	321 (40.53)
Yes	2,070 (46.89)	6,595 (41.25)‡	5,024 (54.88)	12,050 (45.57)‡	203 (64.04)	471 (59.47)†
**Breathing difficulty**						
No	3,936 (89.15)	14,283 (89.34)	7,549 (82.47)	22,425 (84.81)	200 (63.09)	541 (68.31)
Yes	479 (10.85)	1,705 (10.66)NS	1,605 (17.53)	4,016 (15.19)‡	117 (36.91)	251 (31.69)†
**Rapid breathing**						
No	4,345 (98.41)	15,783 (98.72)	9,002 (98.34)	26,112 (98.76)	307 (96.85)	771 (97.35)
Yes	70 (1.59)	205 (1.28)†	152 (1.66)	329 (1.24)‡	10 (3.15)	21 (2.65)NS
**Runny nose**						
No	3,057 (69.24)	11,835 (74.02)	6,792 (74.20)	20,496 (77.52)	259 (81.70)	658 (83.08)
Yes	1,358 (30.76)	4,153 (25.98)‡	2,362 (25.80)	5,945 (22.48)‡	58 (18.30)	134 (16.92)NS
Elicitable (can be found out by asking questions of patients/relatives)						
**Abdominal pain/diarrhoea**						
No	4,142 (93.82)	14,939 (93.44)	8,464 (92.46)	24,580 (92.96)	297 (93.69)	728 (91.92)
Yes	273 (6.18)	1,049 (6.56)NS	690 (7.54)	1,861 (7.04)NS	20 (6.31)	64 (8.08)NS
**GIT symptoms**						
No	3,972 (89.97)	14,375 (89.91)	8,100 (88.49)	23,766 (89.88)	282 (88.96)	705 (89.02)
Yes	443 (10.03)	1,613 (10.09)NS	1,054 (11.51)	2,675 (10.12)‡	35 (11.04)	87 (10.98)NS
**Chest pain**						
No	4,286 (97.08)	15,577 (97.43)	8,782 (95.94)	25,621 (96.90)	303 (95.58)	766 (96.72)
Yes	129 (2.92)	411 (2.57)NS	372 (4.06)	820 (3.10)‡	14 (4.42)	26 (3.28)NS
**Fatigue**						
No	4,143 (93.84)	15,229 (95.25)	8,583 (93.76)	25,014 (94.60)	292 (92.11)	750 (94.70)
Yes	272 (6.16)	759 (4.75)‡	571 (6.24)	1,427 (5.40)‡	25 (7.89)	42 (5.30)†
**Headache**						
No	3,753 (85.01)	13,712 (85.76)	7,849 (85.74)	23,283 (88.06)	287 (90.54)	738 (93.18)

Yes	662 (14.99)	2,276 (14.24)NS	1,305 (14.26)	3,158 (11.94)‡	30 (9.46)	54 (6.82)†̄
**Musculoskeletal pain**						
No	4,299 (97.37)	15,595 (97.54)	8,901 (97.24)	25,783 (97.51)	306 (96.53)	771 (97.35)
Yes	116 (2.63)	393 (2.46)NS	253 (2.76)	658 (2.49)†̄	11 (3.47)	21 (2.65)NS
**Sore throat**						
No	3,306 (74.88)	11,968 (74.86)	6,968 (76.12)	19,583 (74.06)	259 (81.70)	649 (81.94)
Yes	1,109 (25.12)	4,020 (25.14)NS	2,186 (23.88)	6,858 (25.94)‡	58 (18.30)	143 (18.06)NS
**Loss of taste**						
No	3,925 (88.90)	15,283 (95.59)	8,301 (90.68)	25,268 (95.56)	303 (95.58)	770 (97.22)
Yes	490 (11.10)	705 (4.41)‡	853 (9.32)	1,173 (4.44)‡	14 (4.42)	22 (2.78)†̄
**Loss of smell**						
No	3,815 (86.41)	15,160 (94.82)	8,186 (89.43)	25,198 (95.30)	307 (96.85)	778 (98.23)
Yes	600 (13.59)	828 (5.18)‡	968 (10.57)	1,243 (4.70)‡	10 (3.15)	14 (1.77)†̄
Measureable						
**Fever**						
No	2,350 (53.23)	9,430 (58.98)	4,555 (49.76)	15,434 (58.37)	163 (51.42)	453 (57.20)
Yes	2,065 (46.77)	6,558 (41.02)‡	4,599 (50.24)	11,007 (41.63)‡	154 (48.58)	339 (42.80)†̄

†̄=p-value<0.05; ‡=p<0.001; NS=p>0.05 or Not significant

GIT= gastrointestinal (nausea + vomiting)

Musculoskeletal pain= muscle /joint pain

§=State composition of geopolitical zones in Nigeria: South-West (Ekiti, Lagos, Ogun, Ondo, Osun and Oyo); South-South (Akwa-Ibom, Bayelsa, Cross-River, Rivers, Delta and Edo); South-East (Abia, Anambra, Ebonyi, Enugu and Imo); North-Central (Benue, Kogi, Kwara, Nasarawa, Niger, and Plateau States as well as the FCT); North-West (Jigawa, Kaduna, Kano, Katsina, Kebbi, Sokoto and Zamfara); and North-East (Adamawa, Bauchi, Borno, Gombe, Taraba and Yobe).

For peer review only - http://bmjopen.bmj.com/site/about/guidelines.xhtml

Performance of individual symptoms for predicting COVID-19 positivity

In the unadjusted model, presentation with cough, runny nose, fatigue, loss of taste, loss of smell, and fever was significantly associated with higher odds of COVID-19 positivity in children (left panel of Table 2). In the adjusted model, however, presentation with the following symptoms remained significantly ($p<0.001$) associated with higher odds of COVID-19 positivity: cough (adjusted OR 1.32, 95% CI: 1.19-1.47), runny nose (aOR 1.48, 95% CI: 1.32-1.66), fatigue (aOR 1.53, 95% CI: 1.21-1.93), loss of taste (aOR 2.26, 95% CI: 1.63-3.12), loss of smell (aOR 4.87, 95% CI: 3.66-6.49), or fever (aOR 1.47, 95% CI: 1.33-1.63). Regarding predictive performance, loss of smell recorded the highest AUROC value (0.56, 95% CI: 0.55-0.56). Specifically, cough recorded the highest sensitivity (47.7%, 95% CI: 45.6-49.7%) and NPV (74.7%, 95% CI: 73.4-75.9%), while loss of smell recorded the highest specificity (98.1%, 95% CI: 97.7-98.4%) and PPV (72.9%, 95% CI: 68.3-77.2%) (see details in Supplementary Table 6).

The presentation of symptoms significantly associated with increased COVID-19 positivity was higher in adults than in children (middle panel of Table 2). In the adjusted model, however, it was only the presentation with cough (aOR 1.77, 95% CI: 1.64-1.91), runny nose (aOR 1.18, 95% CI: 1.08-1.30), chest pain (aOR 1.50, 95% CI: 1.21-1.85), fatigue (aOR 1.39, 95% CI: 1.17-1.64), headache (aOR 1.24, 95% CI: 1.11-1.39), loss of taste (aOR 2.33, 95% CI: 1.83-2.96), loss of smell (aOR 4.18, 95% CI: 3.30-5.28), or fever (aOR 1.58, 95% CI: 1.47-1.71) that remained independently associated with COVID-19 positivity. Regarding predictive performance, presentation with either fever or cough appeared to be the best predictor of COVID-19 positivity (AUROC 0.57, 95% CI: 0.56-0.58). Additionally, presenting with cough or fever recorded the highest sensitivity at 54.3% (52.9-55.8%) and NPV at 70.8% (69.7-71.8%); however, presenting with loss of smell recorded the highest specificity (98.5%; 95% CI: 98.2-98.8%) and PPV (79.4%, 95% CI: 76.0-82.5%) (see details in Supplementary Table 7).

For peer review only - http://bmjopen.bmj.com/site/about/guidelines.xhtml

Table 2: Associations and predictive performance of individual clinical characteristics of the study participants in relation to COVID-19 positivity (based on derivation datasets)

Variable	Children [n=7,911]			Adults [n=13,167]			Elderly [n=399]		
	Unadjusted OR (95% CI)	Adjusted OR (95% CI)*	AUROC (95% CI)§	Unadjusted OR (95% CI)	Adjusted OR (95% CI)‡	AUROC (95% CI)§	Unadjusted OR (95% CI)	Adjusted OR (95% CI)‡	AUROC (95% CI)§
Obvious (visible to healthcare workers on sight)									
**Chills/sweat**<br>No<br>Yes	1.00<br>1.15 (0.77-1.70)NS			1.00<br>1.14 (0.85-1.54)NS			1.00<br>0.30 (0.03-2.57)NS		
**Cough**<br>No<br>Yes	1.00<br>1.39 (1.26-1.53)‡	1.00<br>**1.32 (1.19-1.47)**‡	0.54<br>(0.53-0.55)	1.00<br>1.85 (1.72-1.99)‡	1.00<br>**1.77 (1.64-1.91)**‡	**0.57**<br>**(0.56-0.58)**	1.00<br>1.61 (1.06-2.44)†	1.00<br>1.59 (1.04-2.43)†	0.52<br>(0.47-0.57)
**Breathing difficulty**<br>No<br>Yes	1.00<br>1.02 (0.87-1.19)NS			1.00<br>1.23 (1.11-1.35)‡	1.00<br>1.10 (0.99-1.22)NS		1.00<br>1.71 (1.11-2.66)†	1.00<br>1.74 (1.12-2.72)†	**0.53**<br>**(0.48-0.58)**
**Rapid breathing**<br>No<br>Yes	1.00<br>1.11 (0.73-1.68)NS			1.00<br>1.58 (1.15-2.16)†	1.00<br>1.24 (0.89-1.73)NS		1.00<br>2.04 (0.45-9.23)NS		
**Runny nose**<br>No<br>Yes	1.00<br>1.53 (1.37-1.70)‡	1.00<br>**1.48 (1.32-1.66)**‡	0.52<br>(0.51-0.53)	1.00<br>1.30 (1.20-1.42)‡	1.00<br>**1.18 (1.08-1.30)**‡	0.53<br>(0.52-0.54)	1.00<br>1.12 (0.66-1.88)NS		
Elicitable (can be found out from patients' responses or by asking questions of relatives)									
**Abdominal pain/diarrhoea**<br>No<br>Yes	1.00<br>0.87 (0.71-1.07)NS			1.00<br>1.12 (0.98-1.29)NS			1.00<br>0.62 (0.26-1.44)NS		
**GIT symptoms**<br>No<br>Yes	1.00<br>0.96 (0.82-1.14)NS			1.00<br>1.17 (1.04-1.32)†	1.00<br>1.11 (0.98-1.26)NS		1.00<br>0.91 (0.45-1.83)NS		
**Chest pain**<br>No<br>Yes	1.00<br>1.33 (0.98-1.79)NS			1.00<br>1.75 (1.44-2.14)‡	1.00<br>**1.50 (1.21-1.85)**‡	0.51<br>(0.50-0.51)	1.00<br>1.53 (0.48-4.83)NS		
**Fatigue/weakness**<br>No<br>Yes	1.00<br>1.29 (1.03-1.62)†	1.00<br>**1.53 (1.21-1.93)**‡	0.51<br>(0.51-0.52)	1.00<br>1.25 (1.07-1.46)†	1.00<br>**1.39 (1.17-1.64)**‡	0.51<br>(0.50-0.51)	1.00<br>2.00 (0.90-4.35)NS		
**Headache**<br>No<br>Yes	1.00<br>1.12 (0.98-1.29)NS			1.00<br>1.44 (1.29-1.60)‡	1.00<br>**1.24 (1.11-1.39)**‡	0.52<br>(0.51-0.52)	1.00<br>0.67 (0.31-1.44)NS		
**Musculoskeletal pain**<br>No<br>Yes	1.00<br>1.09 (0.80-1.48)NS			1.00<br>1.04 (0.81-1.32)NS			1.00<br>1.80 (0.59-5.45)NS		

For peer review only - http://bmjopen.bmj.com/site/about/guidelines.xhtml

**Sore throat**<br>No<br>Yes	1.00<br>1.02 (0.91-1.14)NS			1.00<br>0.83 (0.76-0.90)‡			1.00<br>0.82 (0.50-1.35)NS		
**Loss of taste**<br>No<br>Yes	1.00<br>7.07 (5.59-8.95)	1.00<br>**2.26 (1.63-3.12)**‡	0.54<br>(0.54-0.55)	1.00<br>5.73 (4.75-6.91)‡	1.00<br>**2.33 (1.83-2.96)**‡	0.54<br>(0.53-0.54)	1.00<br>4.67 (0.93-23.42)NS		
**Loss of smell**<br>No<br>Yes	1.00<br>7.85 (6.30-9.77)‡	1.00<br>**4.87 (3.66-6.49)**‡	**0.56**<br>**(0.55-0.56)**	1.00<br>6.74 (5.58-8.14)‡	1.00<br>**4.18 (3.30-5.28)**‡	0.55<br>(0.54-0.55)	1.00<br>5.48 (1.12-26.72)†	1.00<br>7.15 (1.44-35.44)†	0.51<br>(0.49-0.52)
Measureable									
**Fever**<br>No<br>Yes	1.00<br>1.43 (1.29-1.58)‡	1.00<br>**1.47 (1.33-1.63)**‡	0.54<br>(0.52-0.55)	1.00<br>1.64 (1.52-1.76)‡	1.00<br>**1.58 (1.47-1.71)**‡	**0.57**<br>**(0.56-0.58)**	1.00<br>1.42 (0.95-2.12)NS		

OR: Odds ratio; CI: Confidence Interval; significant results in the adjusted model are in bold fonts.
*: The fully-adjusted model includes all statistically significant variables from the unadjusted model.
§: Area under receiver operating characteristic curve; figures are for variable that were statistically significant in the adjusted model only; the best predictive AUROC value is highlighted in bold.
Wald's p-values: ‡<0.001; †<0.05; NS=Not significant (p>0.05)

er review only

For peer review only - http://bmjopen.bmj.com/site/about/guidelines.xhtml

Only three symptoms in the elderly patients were significantly associated with COVID-19 positivity both in the unadjusted and adjusted models, with cough (aOR 1.59, 95% CI: 1.04-2.43; p=0.033), difficulty in breathing (aOR 1.74, 95% CI: 1.12-2.72; p=0.015) or loss of smell (aOR 7.15, 95% CI: 1.44-35.44; p=0.016) being independently associated with COVID-19 positivity in the latter model (right panel of Table 2). Regarding predictive performance, presenting with difficulty in breathing appeared to be the best predictor of COVID-19 positivity (AUROC 0.53, 0.48-0.58). However, presenting with cough recorded the highest sensitivity (61.4%, 95% CI: 53.3-69.0%) and NPV (62.1%, 95% CI: 54.1-69.6%), while loss of smell recorded the highest specificity (99.1%, 95% CI: 97.0-99.9%) and PPV (60.0%, 95% CI: 14.7-94.7%) (see details in Supplementary Table 8).

Performance of un-weighted, statistically- and clinically-weighted scores for predicting COVID-19 positivity

In adults, the statistically-derived weighted combined score appeared to be slightly better in predicting COVID-19 positivity (AUROC: 0.65), when compared with both the clinically-derived weighted combined and un-weighted scores with AUROC 0.63 each (Table 3). A similar pattern was recorded in children and elderly populations. For individual symptom thresholds, presenting with ≥2 symptoms on the un-weighted score appeared to be a better predictor of COVID-19 positivity (AUROC 0.61, 95% CI: 0.60-0.61) than the other symptom thresholds in adults. For the statistically weighted score, presenting with ≥4 symptoms appeared to be a better predictor of COVID-19 positivity (AUROC 0.59, 95% CI: 0.58-0.59) than the other symptom thresholds in adults. For the clinically-derived weighted score, presenting with either ≥3 or ≥4 symptoms was a better predictor of COVID-19 positivity (AUROC 0.58, 95% CI: 0.58-0.59) in adults. The detailed results including sensitivity, specificity, PPV and NPV values for each age-group are available in Supplementary Tables 9, 10 and 11. Figure 2 shows the ROC curves comparing the predictive performance of unweighted, statistically- and clinically-derived weighted combined scores in children (2a), adult (2b) and the elderly (2c) populations.

For peer review only - http://bmjopen.bmj.com/site/about/guidelines.xhtml

Table 3: Predictive performance of un-weighted, statistically- and clinically-weighted score thresholds for predicting COVID-19 positivity in children, adults and elderly

Outcome	Score	Un-weighted score	Statically-weighted score	Clinically-weighted score
		AUROC value (95% CI)		
**Children (<17 years) n=8,077**				
**Combined score**		0.6064	0.6177	0.5915
**Symptom threshold**	≥1	0.55 (0.54-0.56)	0.55 (0.54-0.56)	0.55 (0.54-0.56)
	≥2	**0.58 (0.57-0.59)**	0.55 (0.54-0.56)	0.54 (0.53-0.55)
	≥3	0.55 (0.54-0.56)	0.55 (0.54-0.57)	0.56 (0.55-0.57)
	≥4	0.52 (0.52-0.53)	**0.59 (0.58-0.60)**	**0.57 (0.56-0.58)**
**Adults (17-64 years) n=13,274**				
**Combined score**		0.6333	0.6475	0.6389
**Symptom threshold**	≥1	0.56 (0.55-0.56)	0.56 (0.55-0.56)	0.56 (0.55-0.56)
	≥2	**0.61 (0.60-0.61)**	0.58 (0.57-0.58)	0.56 (0.55-0.56)
	≥3	0.56 (0.55-0.57)	0.58 (0.57-0.59)	**0.58 (0.58-0.59)**
	≥4	0.53 (0.53-0.54)	**0.59 (0.58-0.59)**	**0.58 (0.58-0.59)**
**Elderly (≥65 years) n=393**				
**Combined score**		0.5413	0.5453	0.5426
**Symptom threshold**	≥1	0.52 (0.48-0.57)	0.52 (0.48-0.57)	0.52 (0.48-0.57)
	≥2	**0.53 (0.49-0.57)**	0.52 (0.48-0.57)	0.52 (0.48-0.57)

The best predictive AUROC values for each age group are highlighted in bold

Figure 2 about here

External calibration belt and test for model goodness-of-fit

The calibration belt in the produced plots and tests for children (p=0.086), adults (p=0.915) and elderly (p=0.091) is presented in Figure 3 and suggest that the hypothesis of good calibration is not rejected. The calibration belt and tests for the unweighted, statistically- and clinically-weighted scores in children, adults, and elderly are presented in a Supplementary Figure. With the exception of unweighted and clinically weighted scores in children, all other scores showed good calibration.

For peer review only - http://bmjopen.bmj.com/site/about/guidelines.xhtml

Figure 3 about here

Discussion

Principal findings

In this study we developed and validated symptom's prediction scores for COVID-19 positivity, independently in children, adults, and the elderly patients in the Nigerian context. The best individual symptom predictors of COVID-19 positivity in children, adult, and the elderly patients were loss of smell (AUROC 0.56, 95% CI: 0.55-0.56), either fever or cough (AUROC 0.57, 95% CI: 0.56-0.58), and difficulty in breathing (AUROC 0.53, 0.48-0.58), respectively. In adults, all the symptom scores showed similar performance, with the statistically weighted score (AUROC: 0.65) slightly showing better performance than the un-weighted (AUROC: 0.63) and clinically-derived weighted (AUROC: 0.64) scores. Similar results were found in children and the elderly patients. Overall, none of the symptom scores had good enough discrimination to use in practice.

Strengths and limitations of this study

To the best of our knowledge, this is the first study to have developed and validated symptom prediction scores with a view to aiding prompt recognition of COVID-19 by frontline healthcare workers in the Nigerian context and possibly in sub-Sahara Africa at large. Despite the limited accuracy of the developed prediction tool, the findings are very important for a country with limited capacity for molecular diagnosis of COVID-19, as it provides the evidential basis for advocacy for more investments in molecular diagnostics by policymakers in Nigeria. We also adopted a transparent methodology and adhered to the TRIPOD reporting statement hence minimising the vagueness often associated with the reporting of studies on the predictive performance of diagnostic models for COVID-19.[9] The methodology taken to the derivation of weighted scores is also a strength of this study. In accordance with the preferred approach for building prediction models,[9] the participatory approach taken to deriving the clinically weighted score can enhance the study relevance in the medical community.[23] The use of beta regression coefficients as opposed to odds ratios in deriving the statistical weighted scores has the advantage of being less prone to bias by small to moderate sample size.[24] A common limitation of many COVID-19 diagnostic models is bias due to over-fitting of the models on data that are not representative of the target population.[9] By using SORMAS database (hosts data from all over the country) for both derivation and validation in the present

For peer review only - http://bmjopen.bmj.com/site/about/guidelines.xhtml

study, our findings are considerably generalisable to the COVID-19 situation in Nigeria and less prone to overestimation of COVID-19 risk among individuals tested.

This study however has some limitations that warrant discussion. First, being an analysis of secondary data based on practical recording of routine clinical assessments, the fundamental assumption is that the data recorded on clinical symptoms are reasonably complete; for instance, we assumed that where a symptom was not recorded as being absent rather than missing. Without any objective means of verifying this assumption, any bias caused by misclassification of the individual symptoms could potentially minimise differences in comparisons, in which case observed differences are likely to be in the direction of the null hypothesis. Second, the approach of splitting the dataset for both derivation and validation of symptom scores may have lowered the precision of estimated effect (wider 95% CIs) [25] and potentially underestimated prediction performance due to loss of power;[26] Moreover, evidence supporting the 10 event per outcome rule of thumb has been found by Smeden *et al*.[27] to be weak. Third, the study lacked detailed clinical laboratory data, such as record for albumin or albumin/globin, direct bilirubin values, and red blood cell distribution width, which have been found to be significant variables in COVID-19 diagnostic models.[9] Practically, however, the time and technical requirements for testing these laboratory data could limit their clinical utility.

Interpretation & implications of findings

Based on the systematic and critical review of diagnostic scores by Wynant *et al*., the performance of a diagnostic model is influenced by its composition, with higher number of clinical and laboratory parameters in a model indicating better predictive performance.[9] For instance, studies in China,[28,29] Brazil,[30] Italy,[31], the Netherlands[32] and France[33] with several clinical and laboratory parameters recorded excellent discriminatory performance, albeit with substantial evidence of bias[9] and limited clinical utility. Conversely, a prediction model (containing fewer number of symptoms, heart rate, systolic and diastolic blood pressure) developed by Sun *et al*.[34] in Singapore had a poor discriminatory capacity (C statistic: 0.65; 0.57-0.73). There is evidence to however suggest that the discriminatory accuracy of a prediction model, particularly its sensitivity, can be enhanced by including certain variables including a combination of loss of smell or taste and fever.[35] In the absence of comparison study from a sub-Saharan African country, it is difficult to fully explain the variation in the findings from the present study and elsewhere. Thus a follow-up study using both clinical and

For peer review only - http://bmjopen.bmj.com/site/about/guidelines.xhtml

laboratory parameters in a Nigerian setting or in sub-Saharan Africa (with similar healthcare system and demographic structure) is recommended.

Prediction performance of the un-weighted score with regards to COVID-19 positivity was better in adults than in children and the elderly patients in our study, though the predictive capacity of all the scores was poor overall. This finding has an important implication on NCDC's current definition of COVID-19 suspected cases, which emphasises acute respiratory symptoms and either travel history within 14 days prior to symptom onset or self-reported contact with a confirmed case.[13] Given our findings are indicative of age-dependency of symptom, it may be useful to review the current case definitions of COVID-19 in Nigeria. For example, we found loss of smell and either fever or cough to be better in predicting COVID-19 positivity in children and adults, respectively, while breathing difficulty was more predictive of the disease in the elderly patients.

Loss of smell recorded the highest specificity with regards to COVID-19 positivity for the three age groups: 98.1% in children, 98.5% in adults and 99.1% in the elderly. However, unlike the present study which explored the predictive capacity of loss of smell and taste separately, a combination of both symptoms has been shown to be more predictive.[36] Thus, the potential use of both loss of smell and taste to differentiate COVID-19 from endemic febrile and respiratory illnesses in Nigeria, such as malaria and pneumonia, with overlapping symptoms warrant further study. Additionally, possibility of using both loss of smell and taste as early indicators of emerging COVID-19 wave or a surge in Nigeria would be useful in improving COVID-19 response, such as allocation of already limited testing resources, risk communication, and aid decision making concerning lockdowns and quarantines.[37] The poor predictive capacity of cough or fever alone in the present study is congruent with that in a meta-analysis.[38]

Clinical validity (characterised by sensitivity, specificity and AUROC values) is an important criterion for assessing a clinical prediction tool[39] as it is —the ability of the prediction tool to distinguish between who has an outcome (in this case SARS-CoV-2 infection) and who does not.[40] The clinical validity of all our prediction scores was generally poor but appeared to be dependent on the number of symptoms. For instance, in our study, the un-weighted and weighted (both statistical and clinical) predictive scores presenting with fewer number of symptoms were more sensitive compared with many symptoms in children and adults; it was however the opposite relative to specificity given ≥4 symptoms recorded higher specificity values than lower symptom thresholds. The poor sensitivity of many symptoms could potentially be attributable to a high proportion of false negatives, suggesting that some

For peer review only - http://bmjopen.bmj.com/site/about/guidelines.xhtml

symptoms have limited validity for COVID-19 in children and adults. However, similarity in the predictive performance of various symptom thresholds on the two weighted scores in elderly suggests that weighting has less predictive value for this group of population. The high specificity of more symptoms could be indicative of low proportion of false positives, underlining the need to accurately assess symptoms. In practice, there is a trade-off between sensitivity and specificity such that when the consequences of having a false positive test is very serious, specificity is prioritised over sensitivity and vice-versa.[41] This is the case for the various symptom thresholds on the un-weighted scale where specificity is higher than sensitivity. A higher specificity over sensitivity is of practical relevance when the political implication of refusing to test someone with suspected COVID-19 is considered, though higher sensitivity over specificity might be given preference in the early phases of a pandemic before surge capacity is reached.

Given the rapid increase in community transmission of COVID-19 cases and deleterious impacts of instituting another lockdown (partial or complete), large-scale surveillance for capturing the epidemiological trend of COVID-19 in Nigeria is crucial. However, Nigeria has limited SARS-CoV-2 testing capacity with an average turnaround of two days, making syndromic surveillance (symptomatic monitoring) a viable complementary surveillance system. As such, our findings would be relevant in informing the design of such a surveillance system, which has been demonstrated, in Japan[42,43] and in the US,[44] to be useful in improving the understanding of COVID-19 epidemiology (often in real-time), assessing the effectiveness of public health interventions, and enhancing preparedness for the emergence of COVID-19 wave or a surge. For instance, an evaluation of a syndromic surveillance system in the US found new taste/smell loss to be highly correlated with a range of COVID-19 outcomes, highlighting their usefulness in supporting the surveillance system as an early warning system for COVID-19 prevention and control. However, the feasibility (e.g. considering selection bias and recall bias) and acceptability of a syndromic surveillance system first need to be ascertained given the large proportion of asymptomatic COVID-19 cases at diagnosis in Nigeria.[8]Positive predictive values across the various prediction thresholds, especially for the weighted scales, were generally low despite increasing proportionately with the thresholds. This could be attributable, in part, to the general mildness of the pandemic with resultant low incidence of mortality in Nigeria. For instance, 66% of the 12,289 confirmed COVID-19 cases in Nigeria between February 27 and June 6, 2020 were asymptomatic at diagnosis, with an overall cumulative incidence and case fatality rate of 5·6 per 100,000 population and 2·8%,

For peer review only - http://bmjopen.bmj.com/site/about/guidelines.xhtml

respectively[8]—these figures were substantially lower than those from European countries during the same period.[45] As such our predictive tools could perform differently during a more severe COVID-19 outbreak in Nigeria.

Conclusion

This study has investigated the possibility of using symptoms to predict COVID-19 positivity in Nigeria, and found the predictive capacity of various symptom scores to be poor overall. However, the findings have the potential to serve as an advocacy tool for more investments in resources for capacity strengthening of molecular testing for COVID-19 in Nigeria, which is crucial for improving both clinical case management and surveillance.

Acknowledgments

We acknowledge the contribution of all the COVID-19 treatment centres and laboratories within the NCDC Laboratory Networks of both epidemiological and laboratory data to SORMAS on a regular basis. We express our profound gratitude to the NCDC SORMAS team for collating and maintaining a quality dataset utilised for this study. Last but not least, we thank Professor Jo Leonard-Bee of the University of Nottingham for her input on statistical analyses.

References

1. Nigeria Centre for Disease Control. *COVID-19 Situation Report: Weekly Epidemiological Report 3 (Epi Week 44)*. Abuja, https://ncdc.gov.ng/diseases/sitreps/?cat=14&name=An update of COVID-19 outbreak in Nigeria (2020).

2. Onyeaghala AA, and Olajide I. Managing COVID-19 outbreak in Nigeria: Matters arising. *Clinical Chemistry and Laboratory Medicine* 2020; 58: 1645–1650.

3. Oladimeji O, Atiba BP, and Adeyinka DA. Leveraging polymerase chain reaction technique (Genexpert) to upscaling testing capacity for SARS-CoV-2 (COVID-19) in Nigeria: A game changer. *Pan African Medical Journal* 2020; 35: 1–2.

4. Nigeria Centre for Disease Control. NCDC Coronavirus COVID-19 Microsite: Government Laboratories, https://covid19.ncdc.gov.ng/laboratory/ (2020, accessed 6 November 2020).

5. Nkengasong J. Let Africa into the market for COVID-19 diagnostics. *Nature* 2020; 580: 565.

6. Stites EC, and Wilen CB. The Interpretation of SARS-CoV-2 Diagnostic Tests. *Med*. Epub ahead of print 21 August 2020. DOI: 10.1016/j.medj.2020.08.001.

For peer review only - http://bmjopen.bmj.com/site/about/guidelines.xhtml

7. Smith DS, Richey EA, and Brunetto WL. A Symptom-Based Rule for Diagnosis of COVID-19. *SN Compr Clin Med* 2020; 1–8.

8. Elimian KO, Ochu CL, Ilori E, et al. Descriptive epidemiology of coronavirus disease 2019 in Nigeria, 27 February-6 June, 2020. *Epidemiol Infect*; 148. Epub ahead of print 2020. DOI: 10.1017/S095026882000206X.

9. Wynants L, Van Calster B, Collins GS, et al. Prediction models for diagnosis and prognosis of covid-19: Systematic review and critical appraisal. *BMJ* 2020; 369: 18.

10. Abayomi A, Odukoya O, Osibogun A, et al. Presenting Symptoms and Predictors of Poor Outcomes Among 2,184 Patients with COVID-19 in Lagos State, Nigeria. *Int J Infect Dis*; 0. Epub ahead of print October 2020. DOI: 10.1016/j.ijid.2020.10.024.

11. Ibrahim OR, Suleiman BM, Abdullahi SB, et al. Epidemiology of COVID-19 and Predictors of Outcome in Nigeria: A Single-Center Study. *Am J Trop Med Hyg*. Epub ahead of print 26 October 2020. DOI: 10.4269/ajtmh.20-0759.

12. Elimian KO, Ochu CL, Ebhodaghe B, et al. Patient characteristics associated with COVID-19 positivity and fatality in Nigeria: retrospective cohort study. *BMJ Open* 2020; 10: 44079.

13. Nigeria Centre for Disease Control. *Case Definitions for Coronavirus Disease* . Abuja, https://covid19.ncdc.gov.ng/media/files/Case_Definition.pdf (2020, accessed 22 July 2020).

14. Nigeria Centre for Disease Control. *National interim guidelines for clinical management of COVID-19, version 2, May 2020*. Abuja, https://ncdc.gov.ng/themes/common/docs/protocols/177_1584210847.pdf (14 March 2020, accessed 26 May 2020).

15. World Health Organization. Laboratory testing for coronavirus disease (COVID-19) in suspected human cases: Interim guidance, 19 March 2020. *WHO*, https://www.who.int/publications-detail/laboratory-testing-for-2019-novel-coronavirus-in-suspected-human-cases-20200117 (2020, accessed 1 June 2020).

16. Nigeria Centre for Disease Control. *National interim guidelines for clinical management of COVID-19: version 1*. Abuja, https://ncdc.gov.ng/themes/common/docs/protocols/177_1584210847.pdf (14 March 2020, accessed 27 July 2020).

17. Kronbichler A, Kresse D, Yoon S, et al. Asymptomatic patients as a source of COVID-19 infections: A systematic review and meta-analysis. *Int J Infect Dis*. Epub ahead of print 17 June 2020. DOI: 10.1016/j.ijid.2020.06.052.

18. Miri SM, Noorbakhsh F, Mohebbi SR, and Ghaemi A. Higher prevalence of asymptomatic or mild COVID-19 in children, claims and clues. *J Med Virol* 2020; jmv.26069.

19. Glantz SA, Slinker BK, and Neilands TB. *Primer of Applied Regression and Analysis of Variance* . 3rd ed. McGraw-Hill Medical, 2016.

20. Zweig M, and Campbell G. Receiver-operating characteristic (ROC) plots: A fundamental evaluation tool in clinical medicine. *Clinical Chemistry* 1993; 39: 561–577.

For peer review only - http://bmjopen.bmj.com/site/about/guidelines.xhtml

567 21. Moons KGM, Altman DG, Reitsma JB, et al. Transparent reporting of a multivariable
568 prediction model for individual prognosis or diagnosis (TRIPOD): Explanation and
569 elaboration. *Ann Intern Med* 2015; 162: W1–W73.

570 22. Nattino G, Lemeshow S, Phillips G, Finazzi S, and Bertolini G. Assessing the
571 calibration of dichotomous outcome models with the calibration belt. *Stata J* 2017; 17:
572 1003–1014.

573 23. Salloum RG, Theis RP, Pbert L, et al. Stakeholder Engagement in Developing an
574 Electronic Clinical Support Tool for Tobacco Prevention in Adolescent Primary Care.
575 *Children* 2018; 5: 170.

576 24. Nemes S, Jonasson JM, Genell A, and Steineck G. Bias in odds ratios by logistic
577 regression modelling and sample size. *BMC Med Res Methodol* 2009; 9: 56.

578 25. Katz MH. *Multivariable Analysis: A Practical Guide for Clinicians and Public Health*
579 *Researchers*. 3rd Edition. Cambridge University Press, 2011.

580 26. Steyerberg EW, Harrell FE, Borsboom GJJM, et al. Internal validation of predictive
581 models: Efficiency of some procedures for logistic regression analysis. *J Clin*
582 *Epidemiol* 2001; 54: 774–781.

583 27. Van Smeden M, De Groot JAH, Moons KGM, et al. No rationale for 1 variable per 10
584 events criterion for binary logistic regression analysis. *BMC Med Res Methodol* 2016;
585 16: 1–12.

586 28. Wang Z, Weng J, Li Z, et al. Development and Validation of a Diagnostic Nomogram
587 to Predict COVID-19 Pneumonia. *medRxiv* 2020; 2020.04.03.20052068.

588 29. Chen X, Tang Y, Mo Y, et al. A diagnostic model for coronavirus disease 2019
589 (COVID-19) based on radiological semantic and clinical features: a multi-center study.
590 *Eur Radiol* 2020; 30: 4893–4902.

591 30. Batista AF de M, Miraglia JL, Donato THR, and Chiavegatto Filho ADP. COVID-19
592 diagnosis prediction in emergency care patients: a machine learning approach.
593 *medRxiv* 2020; 2020.04.04.20052092.

594 31. Brinati D, Campagner A, Ferrari D, et al. Detection of COVID-19 Infection from
595 Routine Blood Exams with Machine Learning: a Feasibility Study. *medRxiv* 2020;
596 2020.04.22.20075143.

597 32. Kurstjens S, Van Der Horst A, Herpers R, et al. Rapid identification of SARS-CoV-2-
598 infected patients at the emergency department using routine testing. *Clin Chem Lab*
599 *Med* 2020; 58: 1587–1593.

600 33. Tordjman M, Mekki A, Mali R, et al. Pre-test probability for SARS-Cov-2-related
601 Infection Score: the PARIS score. *medRxiv* 2020; 2020.04.28.20081687.

602 34. Sun Y, Koh V, Marimuthu K, et al. Epidemiological and Clinical Predictors of
603 COVID-19. *Clin Infect Dis* 2020; 71: 786–792.

604 35. Roland LT, Gurrola JG, Loftus PA, Cheung SW, and Chang JL. Smell and taste
605 symptom-based predictive model for COVID-19 diagnosis. *Int Forum Allergy Rhinol*.
606 Epub ahead of print 2020. DOI: 10.1002/alr.22602.

607 36. Menni C, Valdes AM, Freidin MB, et al. Real-time tracking of self-reported symptoms

For peer review only

For peer review only - http://bmjopen.bmj.com/site/about/guidelines.xhtml

to predict potential COVID-19. *Nat Med* 2020; 26: 1037–1040.

37. Desjardins MR. Syndromic surveillance of COVID-19 using crowdsourced data. Epub ahead of print 2020. DOI: 10.1016/j.lanwpc.2020.10.

38. Borsetto D, Hopkins C, Philips V, et al. Self-reported alteration of sense of smell or taste in patients with COVID-19: a systematic review and meta-analysis on 3563 patients. *Rhinology*. Epub ahead of print 6 July 2020. DOI: 10.4193/Rhin20.185.

39. Myles PR, Nguyen-Van-Tam JS, Lim WS, et al. Comparison of CATs, CURB-65 and PMEWS as Triage Tools in Pandemic Influenza Admissions to UK Hospitals: Case Control Analysis Using Retrospective Data. *PLoS One* 2012; 7: e34428.

40. Simon R. Clinical trial designs for evaluating the medical utility of prognostic and predictive biomarkers in oncology. *Personalized Medicine* 2010; 7: 33–47.

41. Akobeng AK. Understanding diagnostic tests 3: Receiver operating characteristic curves. *Acta Paediatrica, International Journal of Paediatrics* 2007; 96: 644–647.

42. Yoneoka D, Tanoue Y, Kawashima T, et al. Large-scale epidemiological monitoring of the COVID-19 epidemic in Tokyo. *Lancet Reg Heal - West Pacific*; 3. Epub ahead of print 1 October 2020. DOI: 10.1016/j.lanwpc.2020.100016.

43. Nomura S, Yoneoka D, Shi S, et al. An assessment of self-reported COVID-19 related symptoms of 227,898 users of a social networking service in Japan: Has the regional risk changed after the declaration of the state of emergency? *Lancet Reg Heal - West Pacific* 2020; 1: 100011.

44. Güemes A, Ray S, Aboumerhi K, et al. A syndromic surveillance tool to detect anomalous clusters of COVID-19 symptoms in the United States. *Sci Rep*; 11. Epub ahead of print 2021. DOI: 10.1038/S41598-021-84145-5.

45. Kinross P, Suetens C, Gomes Dias J, et al. Rapidly increasing cumulative incidence of coronavirus disease (COVID-19) in the European Union/European Economic Area and the United Kingdom, 1 January to 15 March 2020. *Eurosurveillance* 2020; 25: 2000285.

Competing interests

None declared.

Funding

This study received no specific grant from any funding agency in the public, commercial or not-for-profit sectors.

Data sharing statement

The dataset utilised for this study can be made available upon reasonable request to the Head of NCDC Research at chinwe.ochu@ncdc.gov.ng

For peer review only

Authors' contribution

KE contributed to the conceptualization, literature search, data management, data analysis, writing (original draft, review and editing) and formatting of document. OA contributed to the conceptualization, writing (review and editing), formatting of manuscript and funding acquisition. JG contributed to the conceptualization, data management, data analysis, writing (original draft, review and editing) and formatting of document. PM contributed to the writing (original draft, review and editing), literature search and formatting of manuscript. CO contributed to the writing (review and editing) and formatting of manuscript. CK contributed to the writing (review and editing) and formatting of manuscript. TO contributed to the writing (review and editing) and formatting of manuscript. GG contributed to the writing (review and editing) and formatting of manuscript. AO contributed to the writing (review and editing) and formatting of manuscript. HZ contributed to the writing (review and editing) and formatting of manuscript. CO contributed to the writing (review and editing) and formatting of manuscript. BE contributed to the writing (review and editing) and formatting of manuscript. OI contributed to the writing (review and editing) and formatting of manuscript. CDN contributed to the writing (review and editing) and formatting of manuscript. WN contributed to the writing (review and editing) and formatting of manuscript. IAS contributed to data acquisition and curation, and writing (review) of manuscript. OA contributed to the writing (review and editing) and formatting of manuscript. OF contributed to the writing (review and editing) and formatting of manuscript. CA contributed to data acquisition and curation, and writing (review) of manuscript. CE contributed to the writing (review and editing) and formatting of manuscript. JA contributed to the writing (review and editing) and formatting of manuscript. AO contributed to the writing (review and editing) and formatting of manuscript. JA contributed to the writing (review and editing) and formatting of manuscript. EJ contributed to the writing (review and editing) and formatting of manuscript. AB contributed to the writing (review and editing) and formatting of manuscript. BM contributed to the writing (review and editing) and formatting of manuscript. BAS contributed to the writing (review and editing) and formatting of manuscript. AJ contributed to the writing (review and editing) and formatting of manuscript. KH contributed to the writing (review and editing) and formatting of manuscript. CI contributed to the writing (review and editing) and formatting of manuscript. SOB contributed to the writing (review and editing) and formatting of manuscript. MA contributed to the writing (review and editing) and formatting of manuscript. NM contributed to supervision, writing (review), funding acquisition, project administration, and resources. JO contributed to

For peer review only - http://bmjopen.bmj.com/site/about/guidelines.xhtml

supervision, writing (review), funding acquisition, project administration, and resources. EI contributed to supervision, writing (review), funding acquisition, project administration, and resources. TA contributed to the writing (review and editing) and formatting of manuscript. EI contributed to the writing (original draft, review and editing), literature search and formatting of manuscript. CI contributed to conceptualization, supervision, writing (review and editing), funding acquisition, project administration, and resources. All authors invest significant contributions and approved the final draft.

Legends

Figure 1 presents a flow chart showing the processes for selecting records utilised for this study.

Figure 2 presents a comparison of unweighted, statistically weighted and clinically weighted combined scores for predicting COVID-19 positivity. Specifically, Figure 2a focuses on children, 2b on adults, and 2c on the elderly populations.

Figure 3 presents the calibration belt and tests for children, adults and elderly.

For peer review only - http://bmjopen.bmj.com/site/about/guidelines.xhtml

For peer review only

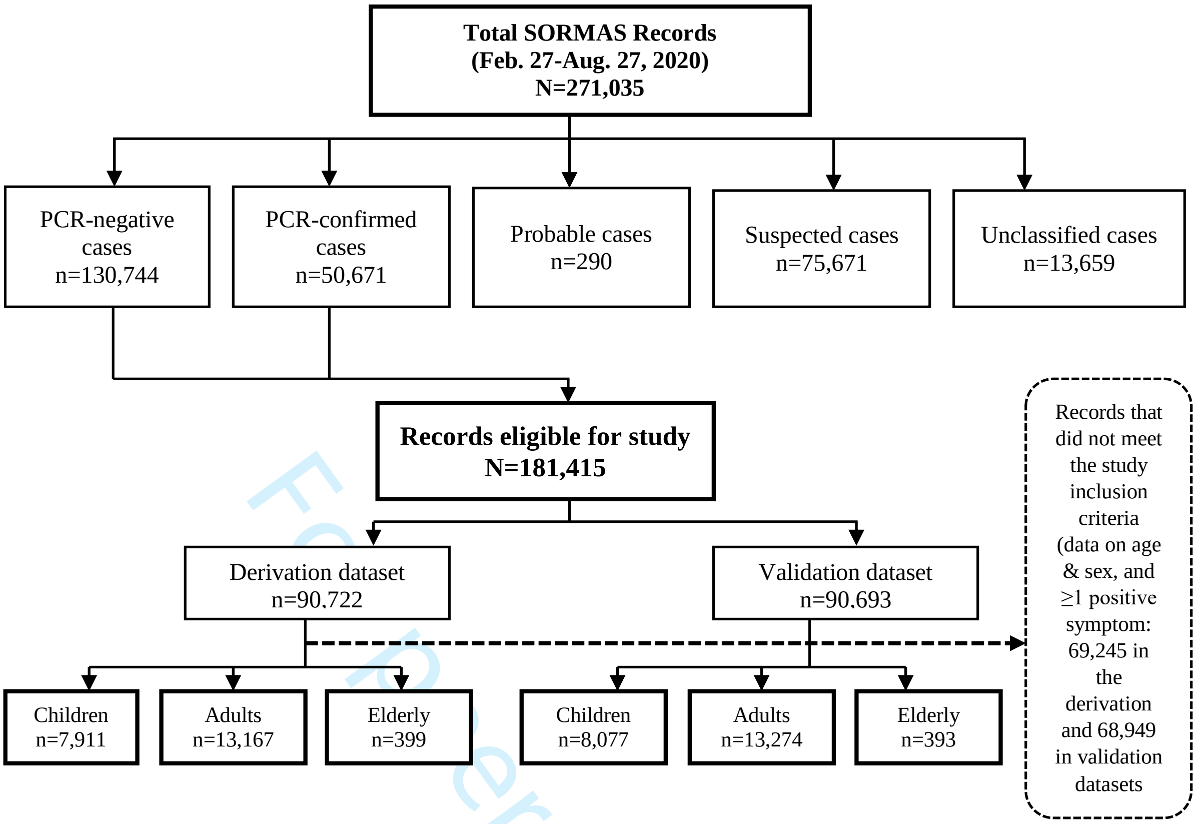

Figure 1: Flow charts showing the processes for selecting records for this study

For peer review only

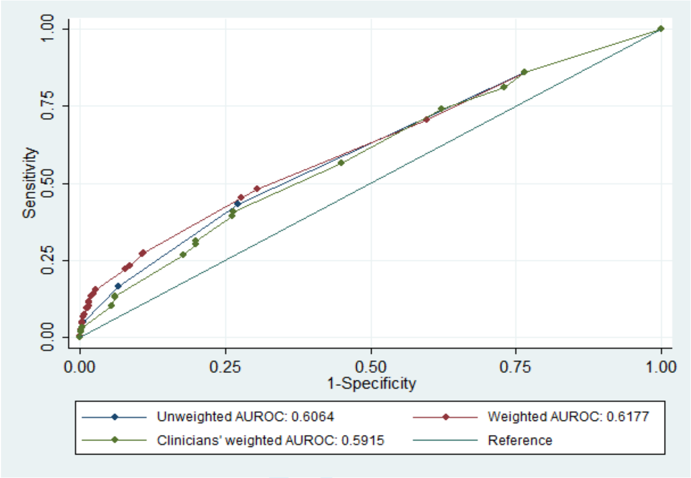

a)

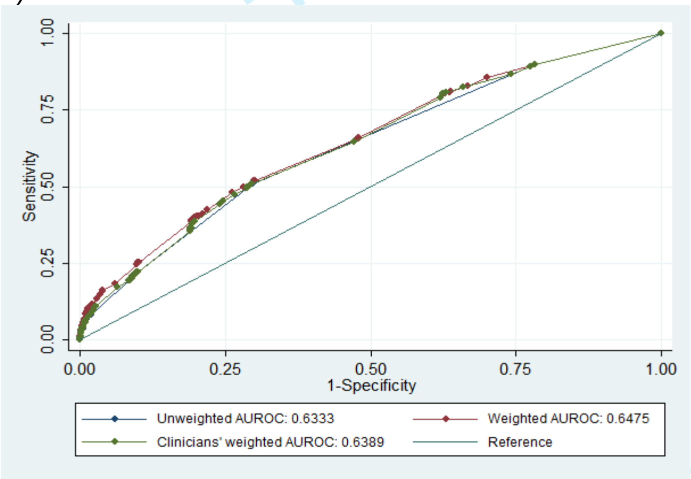

b)

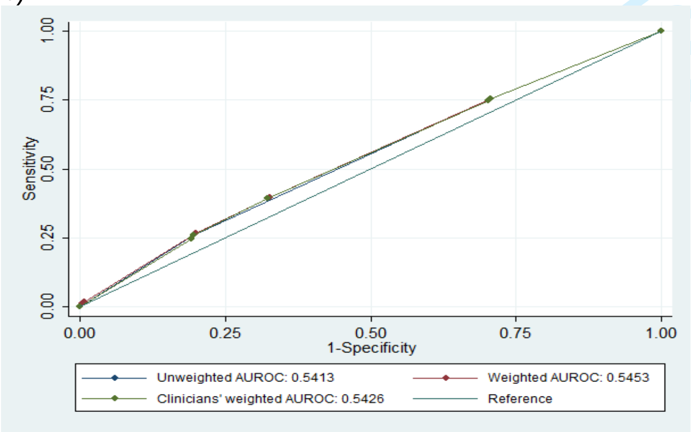

c)

Figure 2: Comparison of unweighted, statistically weighted and clinically weighted combined scores for predicting COVID-19 positivity in children (a), adults (b) and the elderly (c)

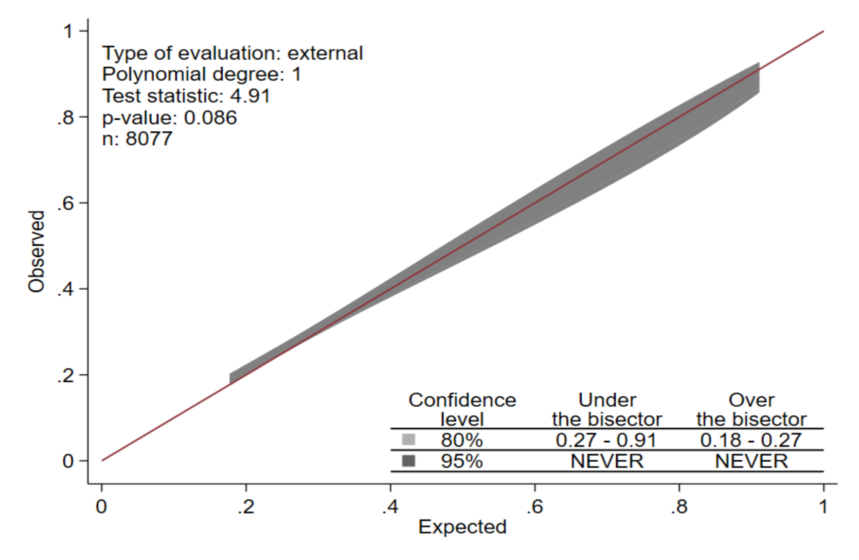

a) Children

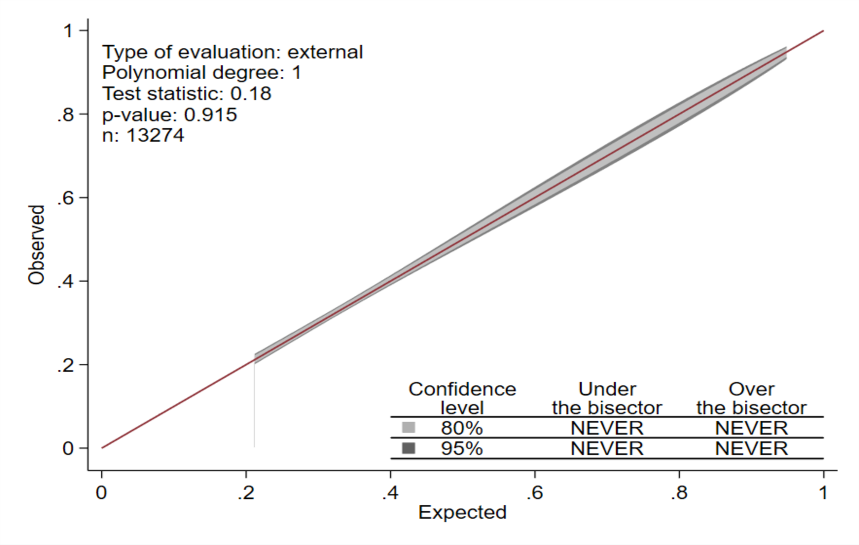

b) Adults

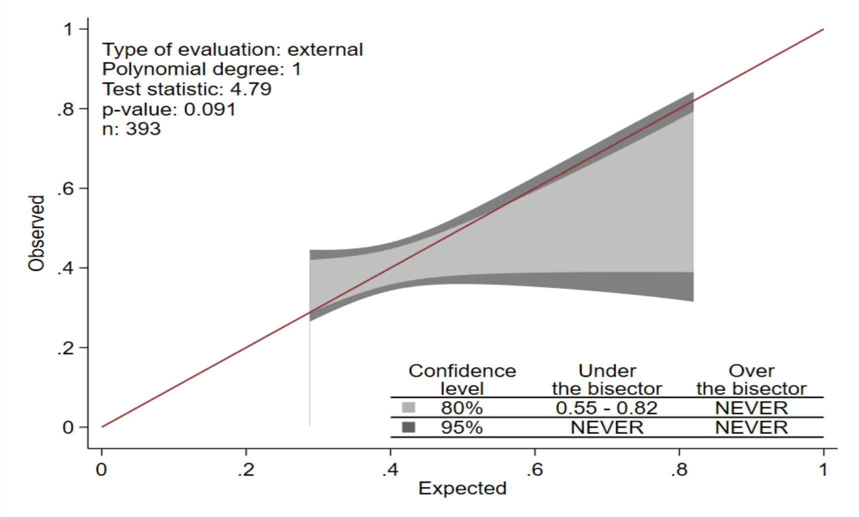

c) Elderly

Figure 3: Calibration belts and tests for children (upper panel), adults (middle panel), and elderly (lower panel)

Supplementary Materials

Supplementary Table 1: NCDC COVID-19 case definitions, February 27-August 27, 2020	
**Case category**	**Definition/criteria**
**Suspected COVID-19 case**	**1. Symptoms with international travel**<br>Anyone with acute respiratory symptoms (fever and either cough, difficulty breathing or shortness of breath) OR new respiratory symptoms (cough, difficulty breathing or shortness of breath) without fever and no other explanation, **AND** a history of travel to or residence in a country reporting cases within 14 days before symptom onset; Or new respiratory symptoms with contact to a confirmed case in the last 14 days before symptom onset;<br>OR<br>**2. Symptoms with contact to confirmed case**<br>Anyone with new respiratory symptoms (cough, difficulty breathing or shortness of breath, with or without fever), **AND** contact with a confirmed or probable COVID-19 case in the last 14 days prior to symptom onset;<br>OR<br>**3. Acute respiratory illness in an area of moderate or high COVID-19 prevalence with no other explanation**<br>Anyone with acute respiratory illness within the last 10 days (fever and either cough, difficulty breathing or shortness of breath), **AND** absence of an alternative diagnosis that explains the clinical presentation **AND** residing or working in the last 14 days in an area identified by NCDC as a moderate or high prevalence region.
**Probable case**	Any suspected case:<br>• For whom testing for COVID-19 is indeterminate test results;<br>• For whom testing was positive on a pan-coronavirus assay; OR<br>• Where samples were not collected prior to the demise of a suspect case
**Confirmed case**	Anyone with laboratory confirmation of SARS-CoV-2 infection with or without signs and symptoms.
**Contact**	Defined as anyone who experienced any one of the following exposures during the 2 days before and/or within 14 days after the onset of symptoms of a probable or confirmed case:<br>• Face-to-face contact with a probable or confirmed case within 1 metre and for more than 15 minutes;<br>• Direct physical contact with a probable or confirmed case;<br>• Direct care for a patient with probable or confirmed COVID-19 disease without using proper personal protective equipment; OR<br>• Other situations as indicated by local risk assessments.<br><br>For confirmed asymptomatic cases, the period of contact is measured as the 2 days before, through the 14 days after the date on which the sample was taken which led to confirmation.

For peer review only

For peer review only - http://bmjopen.bmj.com/site/about/guidelines.xhtml

Assessment of multicollinearity between clinical symptoms and COVID-19 positivity

The VIF and tolerance values (Supplementary Tables 3a, 3b and 3c for results) from the assessment of multicollinearity between the statistically significant symptoms in the unadjusted models and COVID-19 positivity in children, adults and elderly were less than 10 and greater than 0.1, implying it was appropriate to combine the independent symptoms into an index or clinical score.

Supplementary Table 2a: Findings from the assessment of multicollinearity between clinical signs and symptoms and COVID-19 diagnosis in children

Clinical sign/symptom	Derivation dataset n=7,911	
	Variance of Inflation Factor (VIF)	Tolerance
Cough	1.02	0.9800
Runny nose	1.03	0.9724
Fatigue	1.01	0.9864
Loss of taste	1.95	0.5132
Loss of smell	1.95	0.5139
Fever	1.01	0.9853

Mean VIF=1.33

Supplementary Table 2b: Findings from the assessment of multicollinearity between clinical signs and symptoms and COVID-19 diagnosis in adults

Clinical sign/symptom	Derivation dataset n=13,167	
	Variance of Inflation Factor (VIF)	Tolerance
Cough	1.05	0.9569
Difficulty breathing	1.04	0.9613
Rapid breathing	1.01	0.9901
Runny nose	1.03	0.9734
Gastrointestinal tract symptom	1.02	0.9847
Chest pain	1.02	0.9770
Fatigue	1.02	0.9805
Headache	1.04	0.9609
Loss of taste	1.79	0.5574
Loss of smell	1.79	0.5581
Fever	1.03	0.9678

Mean VIF=1.17

Supplementary Table 2c: Findings from the assessment of multicollinearity between clinical signs and symptoms and COVID-19 diagnosis in elderly

Clinical sign/symptom	Derivation dataset n=3,369	
	Variance of Inflation Factor (VIF)	Tolerance
Cough	1.01	0.9871
Difficulty breathing	1.02	0.9800
Loss of smell	1.01	0.9891

Mean VIF=1.01

For peer review only - http://bmjopen.bmj.com/site/about/guidelines.xhtml

Supplementary Table 3: Assignment of clinical weights based on clinicians' judgement or experiences from managing COVID-19 cases in Nigeria

Symptom	Weighting based on clinicians' judgement						
	**Delta State**	**FCT**	**Kaduna State**	**Gombe State**	**Infectious disease consultant§**	**Infectious disease consultant¶**	**Average weight‡**
**Children**							
Cough	3	5	3	1	3	3	**3.0**
Runny nose	2	2	4	2	2	2	**2.3**
Fatigue	2	2	2	1	1	1	**1.5**
Loss of taste	1	3	2	1	1	1	**1.5**
Loss of smell	1	3	2	1	1	1	**1.5**
Fever	2	5	3	5	3	5	**3.8**
**Adults**							
Cough	5	5	4	5	3	3	**4.2**
Runny nose	3	1	2	3	2	3	**2.3**
Chest pain	3	1	2	1	3	2	**2.0**
Fatigue	5	1	3	4	2	4	**3.2**
Headache	3	2	4	1	2	1	**2.2**
Loss of taste	4	3	3	4	5	3	**3.7**
Loss of smell	5	3	3	4	5	3	**3.8**
Fever	3	5	4	4	3	5	**4.0**
**Elderly**							
Cough	4	5	4	5	3	4	**4.2**
Breathing difficulty	5	5	4	5	5	5	**4.8**
Loss of smell	5	3	3	5	5	3	**4.0**

FCT: Federal Capital Territory
‡: Sum of weights from various sources divided by 6 (i.e. number of weight sources)
§: Provides care to COVID-19 patients in a health-facility in the FCT
¶: Provides home-care to COVID-19 patients in the FCT

review only

Table 4: Baseline sociodemographic and clinical characteristics of the study participants in relation to COVID-19 infection (derivation dataset)

Variable	Children		Adults		Elderly	
	PCR-confirmed cases [n=2,155 (%)]	PCR positive and negative cases [N=7,911 (%)]	PCR-confirmed cases [n=4,542 (%)]	PCR positive and negative cases [N=13,167 (%)]	PCR-confirmed cases [n=159 (%)]	PCR positive and negative cases [N=399 (%)]
**Socio-demographic features**						
**Sex**						
Female	902 (41.86)	3,065 (38.74)	1,534 (33.77)	4,814 (36.56)	49 (30.82)	148 (37.09)
Male	1,253 (58.14)	4,846 (61.26)†	3,008 (66.23)	8,353 (63.44)‡	110 (69.18)	251 (62.91)†
**Geopolitical zone§**						
South-west	632 (29.33)	1,761 (22.26)	1,285 (28.29)	3,111 (23.63)	47 (29.56)	97 (24.31)
South-south	712 (33.04)	2,823 (35.68)	1,617 (35.60)	5,441 (41.32)	51 (32.08)	164 (41.10)
South-east	46 (2.13)	129 (1.63)	139 (3.06)	294 (2.23)	11 (6.92)	16 (4.01)
North-central	402 (18.65)	1,557 (19.68)	749 (16.49)	2,045 (15.53)	13 (8.18)	32 (8.02)
North-west	315 (14.62)	1,498 (18.94)	609 (13.41)	1,978 (15.02)	20 (12.58)	63 (15.79)
North-east	48 (2.23)	143 (1.81)‡	143 (3.15)	298 (2.26)‡	17 (10.69)	27 (6.77)†
**Setting**						
Rural	111 (5.15)	429 (5.42)	233 (5.13)	755 (5.73)	20 (12.58)	41 (10.28)
Urban	1,015 (47.10)	3,467 (43.83)	2,218 (48.83)	6,485 (49.25)	92 (57.86)	209 (52.38)
Missing	1,029 (47.75)	4,015 (50.75)†	2,091 (46.04)	5,927 (45.01)†	47 (29.56)	149 (37.34)†
**Education**						
None	25 (1.16)	107 (1.35)	62 (1.37)	169 (1.28)	15 (9.43)	31 (7.77)
Nursery	16 (0.74)	70 (0.88)	2 (0.04)	4 (0.03)	0 (0.00)	0 (0.00)
Primary	67 (3.11)	250 (3.16)	41 (0.90)	151 (1.15)	2 (1.26)	5 (1.25)
Secondary	181 (8.40)	681 (8.61)	378 (8.32)	1,098 (8.34)	14 (8.81)	33 (8.27)
Tertiary	692 (32.11)	2,043 (25.82)	1,574 (34.65)	4,071 (30.92)	45 (28.30)	82 (20.55)
Other	98 (4.55)	540 (6.83)	134 (2.95)	336 (2.55)	13 (8.18)	25 (6.27)
Missing	1,076 (49.93)	4,220 (53.34)‡	2,351 (51.76)	7,338 (55.73)‡	70 (44.03)	223 (55.89)†
**Occupation**						
Student/pupil	297 (13.78)	1,401 (17.71)	212 (4.67)	775 (5.89)	1 (0.63)	2 (0.50)
Child/housewife	67 (3.11)	241 (3.05)	81 (1.78)	228 (1.73)	7 (4.40)	23 (5.76)
Business/trading	106 (4.92)	384 (4.85)	397 (8.74)	1,050 (7.97)	6 (3.77)	18 (4.51)
Transporter	6 (0.28)	35 (0.44)	24 (0.53)	87 (0.66)	0 (0.00)	1 (0.25)
Healthcare worker	269 (12.48)	950 (12.01)	479 (10.55)	1,473 (11.19)	4 (2.52)	13 (3.26)
Laboratorian	10 (0.46)	26 (0.33)	12 (0.26)	35 (0.27)	0 (0.00)	0 (0.00)
Farmer	18 (0.84)	92 (1.16)	84 (1.85)	283 (2.15)	16 (10.06)	27 (6.77)
Animal-related worker	3 (0.14)	11 (0.14)	15 (0.33)	40 (0.30)	1 (0.63)	4 (1.00)
Religious/traditional leader	2 (0.09)	11 (0.14)	30 (0.66)	69 (0.52)	2 (1.26)	4 (1.00)

For peer review only - http://bmjopen.bmj.com/site/about/guidelines.xhtml

Other	826 (38.33)	2,609 (32.98)	2,008 (44.21)	5,633 (42.78)	85 (53.46)	187 (46.87)
Missing	551 (25.57)	2,151 (27.19)‡	1,200 (26.42)	3,494 (26.54)‡	37 (23.27)	120 (30.08)NS
**Clinical signs and symptoms**						
**Clinical outcome**						
Recovered	1,396 (64.78)	2,221 (28.07)	2,844 (62.62)	3,677 (27.93)	70 (44.03)	89 (22.31)
Dead	15 (0.70)	24 (0.30)	229 (5.04)	252 (1.91)	50 (31.45)	50 (12.53)
No outcome yet	744 (34.52)	5,666 (71.62)‡	1,469 (32.34)	9,238 (70.16)‡	39 (24.53)	260 (65.16)‡
Obvious (visible to healthcare workers on sight)						
**Chills/sweat**						
No	2,119 (98.33)	7,791 (98.48)	4,471 (98.44)	12,978 (98.56)	158 (99.37)	393 (98.50)
Yes	36 (1.67)	120 (1.52)NS	71 (1.56)	189 (1.44)NS	1 (0.63)	6 (1.50)NS
**Cough**						
No	1,162 (53.92)	4,725 (59.73)	2,023 (44.54)	7,179 (54.52)	53 (33.33)	160 (40.10)
Yes	993 (46.08)	3,186 (40.27)‡	2,519 (55.46)	5,988 (45.48)‡	106 (66.67)	239 (59.90)†
**Breathing difficulty**						
No	1,912 (88.72)	7,031 (88.88)	3,778 (83.18)	11,181 (84.92)	102 (64.15)	283 (70.93)
Yes	243 (11.28)	880 (11.12)NS	764 (16.82)	1,986 (15.08)‡	57 (35.85)	116 (29.07)†
**Rapid breathing**						
No	2,123 (98.52)	7,802 (98.62)	4,470 (98.41)	13,008 (98.79)	155 (97.48)	392 (98.25)
Yes	32 (1.48)	109 (1.38)NS	72 (1.59)	159 (1.21)†	4 (2.52)	7 (1.75)NS
**Runny nose**						
No	1,450 (67.29)	5,817 (73.53)	3,386 (74.55)	10,218 (77.60)	130 (81.76)	330 (82.71)
Yes	705 (32.71)	2,094 (26.47)‡	1,156 (25.45)	2,949 (22.40)‡	29 (18.24)	69 (17.29)NS
Askable (can be found out by asking questions of patients/relatives)						
**Abdominal pain/diarrhoea**						
No	2,021 (93.78)	7,371 (93.17)	4,193 (92.32)	12,222 (92.82)	151 (94.97)	372 (93.23)
Yes	134 (6.22)	540 (6.83)NS	349 (7.68)	945 (7.18)NS	8 (5.03)	27 (6.77)NS
**GIT symptoms**						
No	1,942 (90.12)	7,110 (89.87)	4,048 (89.12)	11,859 (90.07)	145 (91.19)	362 (90.73)
Yes	213 (9.88)	801 (10.13)NS	494 (10.88)	1,308 (9.93)†	14 (8.81)	37 (9.27)NS
**Chest pain**						
No	2,089 (96.94)	7,711 (97.47)	4,348 (95.73)	12,759 (96.90)	153 (96.23)	387 (96.99)
Yes	66 (3.06)	200 (2.53)NS	194 (4.27)	408 (3.10)‡	6 (3.77)	12 (3.01)NS
**Fatigue**						
No	2,036 (94.48)	7,543 (95.35)	4,269 (93.99)	12,474 (94.74)	144 (90.57)	372 (93.23)
Yes	119 (5.52)	368 (4.65)†	273 (6.01)	693 (5.26)†	15 (9.43)	27 (6.77)NS
**Headache**						
No	1,829 (84.87)	6,796 (85.91)	3,880 (85.42)	11,592 (88.04)	149 (93.71)	367 (91.98)

Yes	326 (15.13)	1,115 (14.09)NS	662 (14.58)	1,575 (11.96)‡	10 (6.29)	32 (8.02)NS
**Musculoskeletal pain**						
No	2,097 (97.31)	7,710 (97.46)	4,440 (97.75)	12,878 (97.81)	152 (95.60)	386 (96.74)
Yes	58 (2.69)	201 (2.54)NS	102 (2.25)	289 (2.19)NS	7 (4.40)	13 (3.26)NS
**Sore throat**						
No	1,615 (74.94)	5,950 (75.21)	3,476 (76.53)	9,766 (74.17)	129 (81.13)	316 (79.20)
Yes	540 (25.06)	1,961 (24.79)NS	1,066 (23.47)	3,401 (25.83)‡	30 (18.87)	83 (20.80)NS
**Loss of taste**						
No	1,909 (88.58)	7,562 (95.59)	4,111 (90.51)	12,581 (95.55)	153 (96.23)	391 (97.99)
Yes	246 (11.42)	349 (4.41)‡	431 (9.49)	586 (4.45)‡	6 (3.77)	8 (2.01)†
**Loss of smell**						
No	1,851 (85.89)	7,489 (94.67)	4,067 (89.54)	12,545 (95.28)	152 (95.60)	390 (97.74)
Yes	304 (14.11)	422 (5.33)‡	475 (10.46)	622 (4.72)‡	7 (4.40)	9 (2.26)†
Measureable signs/symptoms						
**Fever**						
No	1,126 (52.25)	4,634 (58.58)	2,306 (50.77)	7,723 (58.65)	79 (49.69)	219 (54.89)
Yes	1,029 (47.75)	3,277 (41.41)‡	2,236 (49.23)	5,444 (41.35)‡	80 (50.31)	180 (45.11)NS

†=p-value<0.05; ‡=p<0.001; NS=p>0.05 or Not significant
GIT= gastrointestinal (nausea + vomiting)
Musculoskeletal pain= muscle /joint pain
§=State composition of geopolitical zones in Nigeria: South-West (Ekiti, Lagos, Ogun, Ondo, Osun and Oyo); South-South (Akwa-Ibom, Bayelsa, Cross-River, Rivers, Delta and Edo); South-East (Abia, Anambra, Ebonyi, Enugu and Imo); North-Central (Benue, Kogi, Kwara, Nasarawa, Niger, and Plateau States as well as the FCT); North-West (Jigawa, Kaduna, Kano, Katsina, Kebbi, Sokoto and Zamfara); and North-East (Adamawa, Bauchi, Borno, Gombe, Taraba and Yobe).

For peer review only - http://bmjopen.bmj.com/site/about/guidelines.xhtml

Table 5: Baseline sociodemographic and clinical characteristics of the study participants in relation to COVID-19 infection (validation dataset)

Variable	Children		Adults		Elderly	
	PCR-confirmed cases [n=2,260 (%)]	PCR positive and negative cases [N=8,077 (%)]	PCR-confirmed cases [n=4,612 (%)]	PCR positive and negative cases [N=13,274 (%)]	PCR-confirmed cases [n= 158 (%)]	PCR positive and negative cases [N=393 (%)]
**Socio-demographic features**						
**Sex**						
Female	871 (38.54)	2,986 (36.97)	1,546 (33.52)	4,808 (36.22)	61 (38.61)	151 (38.42)
Male	1,389 (61.46)	5,091 (63.03)NS	3,066 (66.48)	8,466 (63.78)‡	97 (61.39)	242 (61.58)NS
**Geopolitical zone§**						
South-west	670 (29.65)	1,778 (22.01)	1,307 (28.34)	3,134 (23.61)	39 (24.68)	88 (22.39)
South-south	723 (31.99)	2,866 (35.48)	1,611 (34.93)	5,470 (41.21)	57 (36.08)	176 (44.78)
South-east	44 (1.95)	149 (1.84)	134 (2.91)	315 (2.37)	14 (8.86)	21 (5.34)
North-central	414 (18.32)	1,499 (18.56)	777 (16.85)	2,016 (15.19)	17 (10.76)	44 (11.20)
North-west	343 (15.18)	1,620 (20.06)	649 (14.07)	2,027 (15.27)	25 (15.82)	53 (13.49)
North-east	66 (2.92)	165 (2.04)‡	134 (2.91)	312 (2.35)‡	6 (3.80)	11 (2.80)†
**Setting**						
Rural	119 (5.27)	458 (5.67)	278 (6.03)	807 (6.08)	15 (9.49)	33 (8.40)
Urban	1,033 (45.71)	3,476 (43.04)	2,269 (49.20)	6,603 (49.74)	91 (57.59)	214 (54.45)
Missing	1,108 (49.03)	4,143 (51.29)†	2,065 (44.77)	5,864 (44.18)NS	52 (32.91)	146 (37.15)NS
**Education**						
None	33 (1.46)	110 (1.36)	66 (1.43)	199 (1.50)	10 (6.33)	17 (4.33)
Nursery	14 (0.62)	73 (0.90)	1 (0.02)	3 (0.02)	0 (0.00)	0 (0.00)
Primary	63 (2.79)	270 (3.34)	61 (1.32)	171 (1.29)	6 (3.80)	13 (3.31)
Secondary	204 (9.03)	698 (8.64)	414 (8.98)	1,164 (8.77)	9 (5.70)	25 (6.36)
Tertiary	718 (31.77)	2,023 (25.05)	1,567 (33.98)	4,074 (30.69)	42 (26.58)	86 (21.88)
Other	81 (3.58)	525 (6.50)	127 (2.75)	352 (2.65)	7 (4.43)	20 (5.09)
Missing	1,147 (50.75)	4,378 (54.20)‡	2,376 (51.52)	7,311 (55.08)‡	84 (53.16)	232 (59.03)NS
**Occupation**						
Student/pupil	302 (13.36)	1,408 (17.43)	222 (4.81)	741 (5.58)	0 (0.00)	0 (0.00)
Child/housewife	66 (2.92)	248 (3.07)	97 (2.10)	272 (2.05)	6 (3.80)	19 (4.83)
Business/trading	111 (4.91)	381 (4.72)	360 (7.81)	993 (7.48)	13 (8.23)	29 (7.38)
Transporter	5 (0.22)	45 (0.56)	21 (0.46)	87 (0.66)	0 (0.00)	2 (0.51)
Healthcare worker	332 (14.69)	1,003 (12.42)	494 (10.71)	1,504 (11.33)	3 (1.90)	7 (1.78)
Laboratorian	5 (0.22)	14 (0.17)	14 (0.30)	28 (0.21)	0 (0.00)	0 (0.00)
Farmer	18 (0.80)	100 (1.24)	89 (1.93)	306 (2.31)	8 (5.06)	20 (5.09)

For peer review only - http://bmjopen.bmj.com/site/about/guidelines.xhtml

Animal-related worker	5 (0.22)	17 (0.21)	10 (0.22)	40 (0.30)	0 (0.00)	5 (1.27)
Religious/traditional leader	3 (0.13)	13 (0.16)	23 (0.50)	60 (0.45)	4 (2.53)	5 (1.27)
Other	807 (35.71)	2,517 (31.16)	2,064 (44.75)	5,775 (43.51)	79 (50.00)	188 (47.84)
Missing	606 (26.81)	2,331 (28.86)‡	1,218 (26.41)	3,468 (26.13)†	45 (28.48)	118 (30.03)NS
**Clinical signs and symptoms**						
**Clinical outcome**						
Recovered	1,451 (64.20)	2,222 (27.51)	2,850 (61.80)	3,636 (27.39)	80 (50.63)	95 (24.17)
Dead	21 (0.93)	27 (0.33)	256 (5.55)	297 (2.24)	33 (20.89)	34 (8.65)
No outcome yet	788 (34.87)	5,828 (72.16)‡	1,506 (32.65)	9,341 (70.37)‡	45 (28.48)	264 (67.18)‡
Obvious (visible to healthcare workers on sight)						
**Chills/sweat**						
No	2,236 (98.94)	7,973 (98.71)	4,538 (98.40	13,051 (98.32)	157 (99.37)	390 (99.24)
Yes	24 (1.06)	104 (1.29)NS	74 (1.60)	223 (1.68)NS	1 (0.63)	3 (0.76)NS
**Cough**						
No	1,183 (52.35)	4,668 (57.79)	2,107 (45.69)	7,212 (54.33)	61 (38.61)	161 (40.97)
Yes	1,077 (47.65)	3,409 (42.21)‡	2,505 (54.31)	6,062 (45.67)‡	97 (61.39)	232 (59.03)NS
**Breathing difficulty**						
No	2,024 (89.56)	7,252 (89.79)	3,771 (81.76)	11,244 (84.71)	98 (62.03)	258 (65.65)
Yes	236 (10.44)	825 (10.21)NS	841 (18.24)	2,030 (15.29)‡	60 (37.97)	135 (34.35)NS
**Rapid breathing**						
No	2,222 (98.32)	7,981 (98.81)	4,532 (98.27)	13,104 (98.72)	152 (96.20)	379 (96.44)
Yes	38 (1.68)	96 (1.19)†	80 (1.73)	170 (1.28)†	6 (3.80)	14 (3.56)NS
**Runny nose**						
No	1,607 (71.11)	6,018 (74.51)	3,406 (73.85)	10,278 (77.43)	129 (81.65)	328 (83.46)
Yes	653 (28.89)	2,059 (25.49)‡	1,206 (26.15)	2,996 (22.57)‡	29 (18.35)	65 (16.54)NS
Askable (can be found out by asking questions of patients/relatives)						
**Abdominal pain/diarrhoea**						
No	2,121 (93.85)	7,568 (93.70)	4,271 (92.61)	12,358 (93.10)	146 (92.41)	356 (90.59)
Yes	139 (6.15)	509 (6.30)NS	341 (7.39)	916 (6.90)NS	12 (7.59)	37 (9.41)NS
**GIT symptoms**						
No	2,030 (89.82)	7,265 (89.95)	4,052 (87.86)	11,907 (89.70)	137 (86.71)	343 (87.28)
Yes	230 (10.18)	812 (10.05)NS	560 (12.14)	1,367 (10.30)‡	21 (13.29)	50 (12.72)NS
**Chest pain**						
No	2,197 (97.21)	7,866 (97.39)	4,434 (96.14)	12,862 (96.90)	150 (94.94)	379 (96.44)
Yes	63 (2.79)	211 (2.61)NS	178 (3.86)	412 (3.10)‡	8 (5.06)	14 (3.56)NS
**Fatigue**						
No	2,107 (93.23)	7,686 (95.16)	4,314 (93.54)	12,540 (94.47)	148 (93.67)	378 (96.18)
Yes	153 (6.77)	391 (4.84)‡	298 (6.46)	734 (5.53)†	10 (6.33)	15 (3.82)†

**Headache**						
No	1,924 (85.13)	6,916 (85.63)	3,969 (86.06)	11,691 (88.07)	138 (87.34)	371 (94.40)
Yes	336 (14.87)	1,161 (14.37)NS	643 (13.94)	1,583 (11.93)‡	20 (12.66)	22 (5.60)‡
**Musculoskeletal pain**						
No	2,202 (97.43)	7,885 (97.62)	4,461 (96.73)	12,905 (97.22)	154 (97.47)	385 (97.96)
Yes	58 (2.57)	192 (2.38)NS	151 (3.27)	369 (2.78)†	4 (2.53)	8 (2.04)NS
**Sore throat**						
No	1,691 (74.82)	6,018 (74.51)	3,492 (75.72)	9,817 (73.96)	130 (82.28)	333 (84.73)
Yes	569 (25.18)	2,059 (25.49)NS	1,120 (24.28)	3,457 (26.04)‡	28 (17.72)	60 (15.27)NS
**Loss of taste**						
No	2,016 (89.20)	7,721 (95.59)	4,190 (90.85)	12,687 (95.58)	150 (94.94)	379 (96.44)
Yes	244 (10.80)	356 (4.41)‡	422 (9.15)	587 (4.42)‡	8 (5.06)	14 (3.56)NS
**Loss of smell**						
No	1,964 (86.90)	7,671 (94.97)	4,119 (89.31)	12,653 (95.32)	155 (98.10)	388 (98.73)
Yes	296 (13.10)	406 (5.03)‡	493 (10.69)	621 (4.68)‡	3 (1.90)	5 (1.27)NS
Measureable signs/symptoms						
**Fever**						
No	1,224 (54.16)	4,796 (59.38)	2,249 (48.76)	7,711 (58.09)	84 (53.16)	234 (59.54)
Yes	1,036 (45.84)	3,281 (40.62)‡	2,363 (51.24)	5,563 (41.91)‡	74 (46.84)	159 (40.46)†

†=p-value<0.05; ‡=p<0.001; NS=p>0.05 or Not significant
GIT= gastrointestinal (nausea + vomiting)
Musculoskeletal pain= muscle /joint pain
§=State composition of geopolitical zones in Nigeria: South-West (Ekiti, Lagos, Ogun, Ondo, Osun and Oyo); South-South (Akwa-Ibom, Bayelsa, Cross-River, Rivers, Delta and Edo); South-East (Abia, Anambra, Ebonyi, Enugu and Imo); North-Central (Benue, Kogi, Kwara, Nasarawa, Niger, and Plateau States as well as the FCT); North-West (Jigawa, Kaduna, Kano, Katsina, Kebbi, Sokoto and Zamfara); and North-East (Adamawa, Bauchi, Borno, Gombe, Taraba and Yobe).

only

For peer review only - http://bmjopen.bmj.com/site/about/guidelines.xhtml

Table 6: Predictive value of individual clinical symptoms for predicting COVID-19 positivity in children (n=8,077)

Variable	ROC area (95% CI)	Sensitivity (%) (95%CI)	Specificity (%) (95%CI)	PPV (%) (95% CI)	NPV (%) (95% CI)
**Cough**	0.54 (0.53-0.55)	**47.7 (45.6-49.7)**	59.9 (58.6-61.2)	31.6 (30.0-33.2)	**74.7 (73.4-75.9)**
**Runny nose**	0.52 (0.51-0.53)	28.9 (27.0-30.8)	75.8 (74.7-76.9)	31.7 (29.7-33.8)	73.3 (72.2-74.4)
**Fatigue**	0.51 (0.51-0.52)	6.8 (5.8-7.9)	95.9 (95.4-96.4)	39.1 (34.3-44.2)	72.6 (71.6-73.6)
**Loss of taste**	0.54 (0.54-0.55)	10.8 (9.5-12.1)	**98.1 (97.7-98.4)**	68.5 (63.4-73.3)	73.9 (72.9-74.9)
**Loss of smell**	**0.56 (0.55-0.56)**	13.1 (11.7-14.6)	**98.1 (97.7-98.4)**	**72.9 (68.3-77.2)**	74.4 (73.4-75.4)
**Fever**	0.54 (0.52-0.55)	45.8 (43.8-47.9)	61.4 (60.1-62.7)	31.6 (30.0-33.2)	74.5 (73.2-75.7)

The best predictive performance measures (AUROC, sensitivity, specificity, PPV and NPV values) for each outcome are highlighted in bold

Table 7: Predictive value of individual clinical symptoms for predicting COVID-19 positivity in adults (n=13,274)

Variable	ROC area (95% CI)	Sensitivity (%) (95%CI)	Specificity (%) (95%CI)	PPV (%) (95% CI)	NPV (%) (95% CI)
Cough	**0.57 (0.56-0.58)**	**54.3 (52.9-55.8)**	58.9 (57.9-60.0)	41.3 (40.1-42.6)	**70.8 (69.7-71.8)**
Runny nose	0.53 (0.52-0.54)	26.1 (24.9-27.4)	79.3 (78.5-80.2)	40.3 (38.5-42.0)	66.9 (65.9-67.8)
Chest pain	0.51 (0.50-0.51)	3.9 (3.3-4.5)	97.3 (96.9-97.6)	43.2 (38.4-48.1)	65.5 (64.7-66.3)
Fatigue	0.51 (0.50-0.51)	6.5 (5.8-7.2)	95.0 (94.5-95.4)	40.6 (37.0-44.3)	65.6 (64.8-66.4)
Headache	0.52 (0.51-0.52)	13.9 (13.0-15.0)	89.1 (88.5-89.8)	40.6 (38.2-43.1)	66.1 (65.2-66.9)
Loss of taste	0.54 (0.53-0.54)	9.2 (8.3-10.0)	98.1 (97.8-98.4)	71.9 (68.1-75.5)	67.0 (66.1-67.8)
Loss of smell	0.55 (0.54-0.55)	10.7 (9.8-11.6)	**98.5 (98.2-98.8)**	**79.4 (76.0-82.5)**	67.4 (66.6-68.3)
Fever	**0.57 (0.56-0.58)**	51.2 (49.8-52.7)	63.1 (62.0-64.1)	42.5 (41.2-43.8)	**70.8 (69.8-71.8)**

For peer review only - http://bmjopen.bmj.com/site/about/guidelines.xhtml

For peer review only

Table 8: Predictive value of individual clinical symptoms for predicting COVID-19 positivity in the elderly (n=393)

Variable	ROC area (95% CI)	Sensitivity (%) (95%CI)	Specificity (%) (95%CI)	PPV (%) (95% CI)	NPV (%) (95% CI)
Cough	0.52 (0.47-0.57)	**61.4 (53.3-69.0)**	42.6 (36.1-49.1)	41.8 (35.4-48.4)	**62.1 (54.1-69.6)**
Breathing difficulty	**0.53 (0.48-0.58)**	38.0 (30.4-46.0)	68.1 (61.7-74.0)	44.4 (35.9-53.2)	62.0 (55.8-68.0)
Loss of smell	0.51 (0.49-0.52)	1.9 (0.4-5.4)	**99.1 (97.0-99.9)**	**60.0 (14.7-94.7)**	60.1 (55.0-65.0)

Table 9: Predictive performance of un-weighted clinical score thresholds for predicting COVID-19 positivity in children, adults and elderly

Outcome	Score	ROC area (95% CI)	Sensitivity (%) (95% CI)	Specificity (%) (95% CI)	PPV (%) (95% CI)	NPV (%) (95% CI)
**Children (<17 years) n=8,077**						
**Number of symptoms**	≥1	0.55 (0.54-0.56)	**86.1 (84.6-87.5)**	23.6 (22.5-24.7)	30.4 (29.3-31.6)	**81.3 (79.4-83.2)**
	≥2	**0.58 (0.57-0.59)**	43.3 (41.2-45.3)	72.9 (71.7-74.0)	38.3 (36.4-40.2)	76.8 (75.6-77.9)
	≥3	0.55 (0.54-0.56)	16.6 (15.1-18.2)	93.4 (92.7-94.0)	49.4 (45.8-53.0)	74.2 (73.2-75.2)
	≥4	0.52 (0.52-0.53)	5.1 (4.2-6.1)	**99.5 (99.3-99.7)**	**79.3 (71.8-85.6)**	73.0 (72.0-73.9)
**Adults (17-64 years) n=13,274**						
**Number of symptoms**	≥1	0.56 (0.55-0.56)	**89.7 (88.8-90.6)**	21.7 (20.9-22.6)	37.9 (37.0-38.8)	**79.9 (78.2-81.5)**
	≥2	**0.61 (0.60-0.61)**	51.0 (49.5-52.4)	70.3 (69.3-71.2)	47.7 (46.3-49.1)	72.9 (72.0-73.9)
	≥3	0.56 (0.55-0.57)	22.3 (21.1-23.5)	90.0 (89.4-90.6)	54.3 (52.1-56.6)	68.5 (67.7-69.4)
	≥4	0.53 (0.53-0.54)	8.3 (7.5-9.1)	**98.0 (97.7-98.3)**	**68.8 (64.7-72.6)**	66.7 (65.9-67.6)
**Elderly (>65 years) n=393**						
**Number of symptoms**	≥1	0.52 (0.48-0.57)	**75.3 (67.5-81.8)**	29.4 (23.6-35.6)	41.8 (36.0-47.7)	**63.9 (54.1-72.9)**
	≥2	**0.53 (0.49-0.57)**	25.9 (19.3-33.5)	**80.4 (74.8-85.3)**	**47.1 (36.3-58.1)**	61.8 (56.1-67.2)

The best predictive performance measures (AUROC, sensitivity, specificity, PPV and NPV values) for each age group are highlighted in bold

For peer review only - http://bmjopen.bmj.com/site/about/guidelines.xhtml

Table 10: Predictive performance of statistically-derived weighted clinical score thresholds for predicting COVID-19 in children, adults and elderly						
**Outcome**	**Score**	**ROC area (95% CI)**	**Sensitivity (%) (95% CI)**	**Specificity (%) (95% CI)**	**PPV (%) (95% CI)**	**NPV (%) (95% CI)**
**Children (<17 years) n=8,077**						
**Number of symptoms**	≥1	0.55 (0.54-0.56)	**86.1 (84.6-87.5)**	23.6 (22.5-24.7)	30.4 (29.3-31.6)	**81.3 (79.4-83.2)**
	≥2	0.55 (0.54-0.56)	**86.1 (84.6-87.5)**	23.6 (22.5-24.7)	30.4 (29.3-31.6)	**81.3 (79.4-83.2)**
	≥3	0.55 (0.54-0.57)	70.7 (68.8-72.6)	40.3 (39.5-41.5)	31.5 (30.2-32.8)	78.0 (76.4-79.4)
	≥4	**0.59 (0.58-0.60)**	48.1 (46.0-50.2)	**69.4 (68.2-70.6)**	**37.9 (36.1-39.7)**	77.5 (76.3-78.6)
**Adults (17-64 years) n=13,274**						
**Number of symptoms**	≥1	0.56 (0.55-0.56)	**89.7 (88.8-90.6)**	21.7 (20.9-22.6)	37.9 (37.0-38.8)	**79.9 (78.2-81.5)**
	≥2	0.58 (0.57-0.58)	85.6 (84.6-86.6)	30.0 (29.0-30.9)	39.4 (38.5-40.4)	79.6 (78.2-81.0)
	≥3	0.58 (0.57-0.59)	82.9 (81.8-84.0)	33.4 (32.4-34.4)	39.8 (38.9-40.8)	78.6 (77.2-79.9)
	≥4	**0.59 (0.58-0.59)**	80.8 (79.6-81.9)	**36.4 (35.4-37.5)**	**40.4 (39.4-41.4)**	78.1 (76.8-79.3)
**Elderly (>65 years) n=393**						
**Number of symptoms**	≥1	0.52 (0.48-0.57)	75.3 (67.8-81.8)	29.4 (23.6-35.6)	41.8 (36.0-47.7)	63.9 (54.1-72.9)
	≥2	0.52 (0.48-0.57)	75.3 (67.8-81.8)	29.4 (23.6-35.6)	41.8 (36.0-47.7)	63.9 (54.1-72.9)

The best predictive performance measures (AUROC, sensitivity, specificity, PPV and NPV values) for each age group are highlighted in bold

only

For peer review only - http://bmjopen.bmj.com/site/about/guidelines.xhtml

Table 11: Predictive performance of clinically-derived weighted clinical score thresholds for predicting COVID-19 in children, adults and elderly

Outcome	Score	ROC area (95% CI)	Sensitivity (%) (95% CI)	Specificity (%) (95% CI)	PPV (%) (95% CI)	NPV (%) (95% CI)
**Children (<17 years) n=8,077**						
**Number of symptoms**	≥1	0.55 (0.54-0.56)	**86.1 (84.6-87.5)**	23.6 (22.5-24.7)	30.4 (29.3-31.6)	**81.3 (79.4-83.2)**
	≥2	0.54 (0.53-0.55)	81.2 (79.6-82.8)	27.1 (25.9-28.2)	30.2 (29.0-31.4)	78.8 (76.9-80.6)
	≥3	0.56 (0.55-0.57)	74.2 (72.4-76.0)	37.7 (36.5-39.0)	31.7 (30.4-32.9)	79.0 (77.5-80.5)
	≥4	**0.57 (0.56-0.58)**	40.7 (38.6-42.7)	**73.6 (72.4-74.7)**	**37.4 (35.5-39.4)**	76.1 (75.0-77.2)
**Adults (17-64 years) n=13,274**						
**Number of symptoms**	≥1	0.56 (0.55-0.56)	**89.7 (88.8-90.6)**	21.7 (20.9-22.6)	37.9 (37.0-38.8)	**79.9 (78.2-81.5)**
	≥2	0.56 (0.55-0.56)	**89.7 (88.8-90.6)**	21.7 (20.9-22.6)	37.9 (37.0-38.8)	**79.9 (78.2-81.5)**
	≥3	**0.58 (0.58-0.59)**	82.5 (81.3-83.5)	34.1 (33.1-35.1)	40.0 (39.0-41.0)	78.5 (77.1-79.8)
	≥4	**0.58 (0.58-0.59)**	79.0 (77.8-80.2)	**37.9 (36.9-39.0**	**40.4 (39.4-41.4)**	77.2 (75.9-78.5)
**Elderly (>65 years) n=393**						
**Number of symptoms**	≥1	0.52 (0.48-0.57)	75.3 (67.8-81.8)	29.4 (23.6-35.6)	41.8 (36.0-47.7)	63.9 (54.1-72.9)
	≥2	0.52 (0.48-0.57)	75.3 (67.8-81.8)	29.4 (23.6-35.6)	41.8 (36.0-47.7)	63.9 (54.1-72.9)

The best predictive performance measures (AUROC, sensitivity, specificity, PPV and NPV values) for each age group are highlighted in bold

For peer review only - http://bmjopen.bmj.com/site/about/guidelines.xhtml

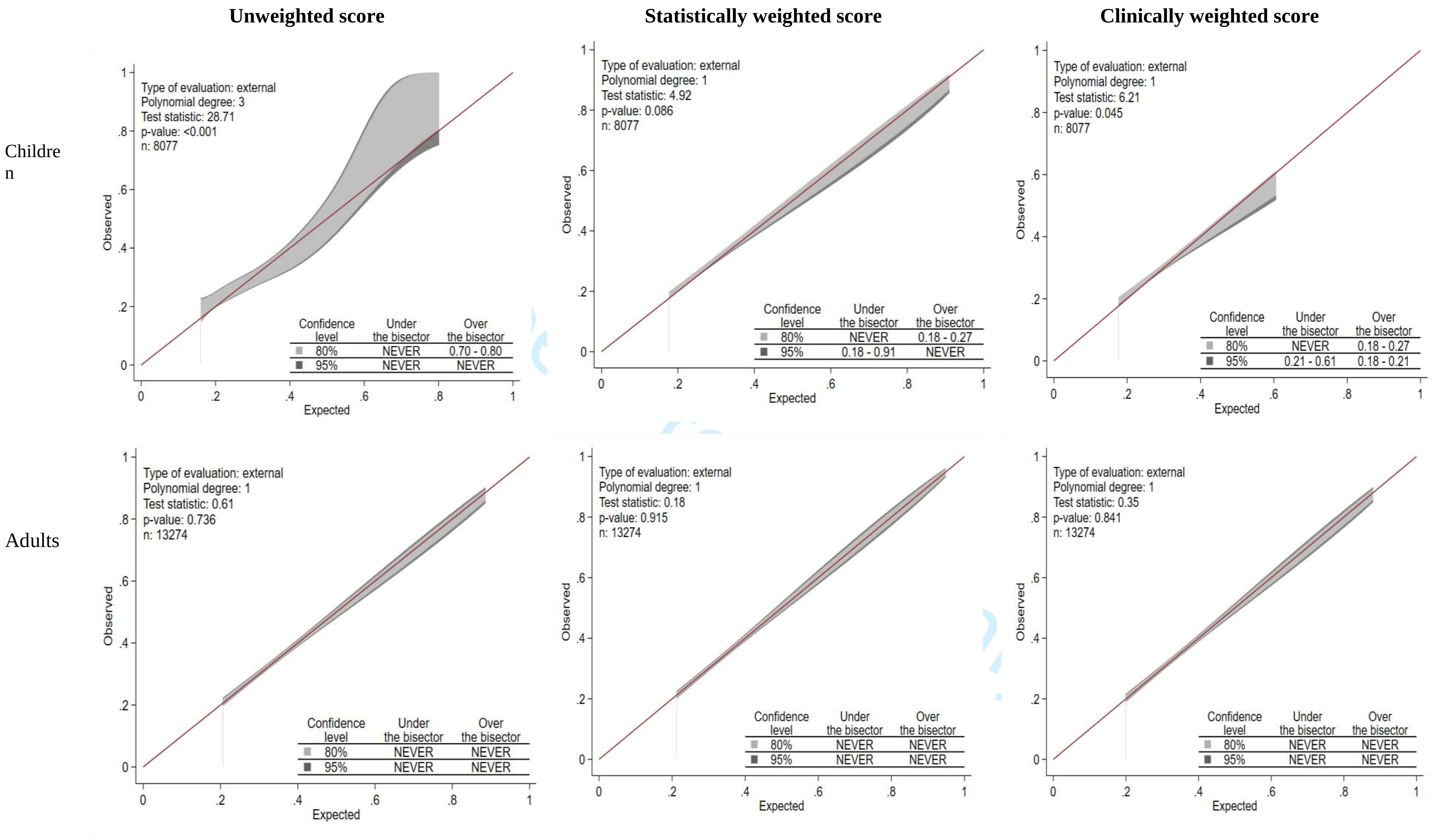
Unweighted score
Statistically weighted score
Clinically weighted score
Children
Adults
Type of evaluation: external
Polynomial degree: 3
Test statistic: 28.71
p-value: <0.001
n: 8077
Confidence level | Under the bisector | Over the bisector
80% | NEVER | 0.70 - 0.80
95% | NEVER | NEVER
Type of evaluation: external
Polynomial degree: 1
Test statistic: 4.92
p-value: 0.086
n: 8077
Confidence level | Under the bisector | Over the bisector
80% | NEVER | 0.18 - 0.27
95% | 0.18 - 0.91 | NEVER
Type of evaluation: external
Polynomial degree: 1
Test statistic: 6.21
p-value: 0.045
n: 8077
Confidence level | Under the bisector | Over the bisector
80% | NEVER | 0.18 - 0.27
95% | 0.21 - 0.61 | 0.18 - 0.21
Type of evaluation: external
Polynomial degree: 1
Test statistic: 0.61
p-value: 0.736
n: 13274
Confidence level | Under the bisector | Over the bisector
80% | NEVER | NEVER
95% | NEVER | NEVER
Type of evaluation: external
Polynomial degree: 1
Test statistic: 0.18
p-value: 0.915
n: 13274
Confidence level | Under the bisector | Over the bisector
80% | NEVER | NEVER
95% | NEVER | NEVER
Type of evaluation: external
Polynomial degree: 1
Test statistic: 0.35
p-value: 0.841
n: 13274
Confidence level | Under the bisector | Over the bisector
80% | NEVER | NEVER
95% | NEVER | NEVER
Observed
Expected

Elderly

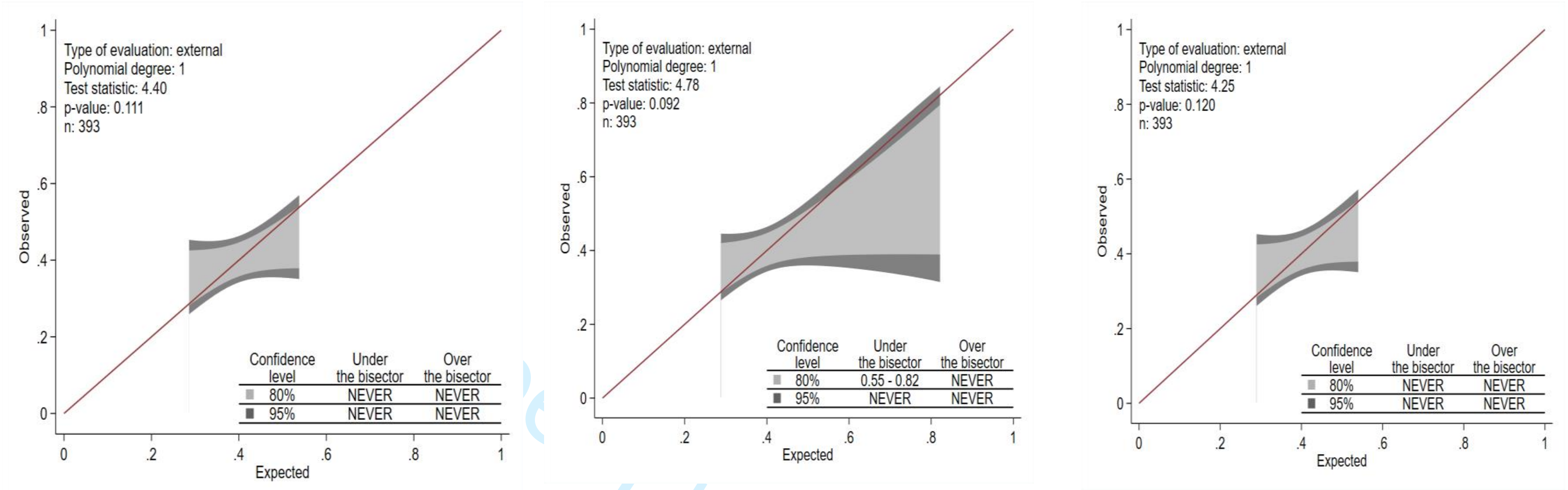

Supplementary Figure: Calibration belts and tests for children (upper panel), adults (middle panel), and elderly (lower panel)

TRIPOD Checklist: Prediction Model Development and Validation

Section/Topic	Item		Checklist Item	Page
**Title and abstract**				
Title	1	D;V	Identify the study as developing and/or validating a multivariable prediction model, the target population, and the outcome to be predicted.	Page 1
Abstract	2	D;V	Provide a summary of objectives, study design, setting, participants, sample size, predictors, outcome, statistical analysis, results, and conclusions.	Page 2; lines 43-69
**Introduction**				
Background and objectives	3a	D;V	Explain the medical context (including whether diagnostic or prognostic) and rationale for developing or validating the multivariable prediction model, including references to existing models.	Page 4; lines 105-130
	3b	D;V	Specify the objectives, including whether the study describes the development or validation of the model or both.	Page 5; lines 138-141
**Methods**				
Source of data	4a	D;V	Describe the study design or source of data (e.g., randomized trial, cohort, or registry data), separately for the development and validation data sets, if applicable.	Page 5; lines 143-149
	4b	D;V	Specify the key study dates, including start of accrual; end of accrual; and, if applicable, end of follow-up.	Page 5; lines 145-146
Participants	5a	D;V	Specify key elements of the study setting (e.g., primary care, secondary care, general population) including number and location of centres.	Page 5; lines 146-147
	5b	D;V	Describe eligibility criteria for participants.	Page 5; lines 152-154
	5c	D;V	Give details of treatments received, if relevant.	Not relevant
Outcome	6a	D;V	Clearly define the outcome that is predicted by the prediction model, including how and when assessed.	Page 6; lines 188-192
	6b	D;V	Report any actions to blind assessment of the outcome to be predicted.	Not relevant, the study being an analysis of a secondary data
Predictors	7a	D;V	Clearly define all predictors used in developing or validating the multivariable prediction model, including how and when they were measured.	Page 6; lines 192-200
	7b	D;V	Report any actions to blind assessment of predictors for the outcome and other predictors.	Not relevant, the study being an analysis of a secondary data
Sample size	8	D;V	Explain how the study size was arrived at.	Page 6; lines 169-172
Missing data	9	D;V	Describe how missing data were handled (e.g., complete-case analysis, single imputation, multiple imputation) with details of any imputation method.	Not relevant given the assumptions made prior to analyses—see page 6, lines 174-187
Statistical analysis methods	10a	D	Describe how predictors were handled in the analyses.	Page 6; lines 175-187
	10b	D	Specify type of model, all model-building procedures (including any predictor selection), and method for internal validation.	Pages 7-8; lines 206-256
	10c	V	For validation, describe how the predictions were calculated.	Page 8; lines 245-250
	10d	D;V	Specify all measures used to assess model performance and, if relevant, to compare multiple models.	Page 8; lines 245-250
	10e	V	Describe any model updating (e.g., recalibration) arising from the validation, if done.	Not done
Risk groups	11	D;V	Provide details on how risk groups were created, if done.	Not done
Development vs. validation	12	V	For validation, identify any differences from the development data in setting, eligibility criteria, outcome, and predictors.	Not relevant
**Results**				
Participants	13a	D;V	Describe the flow of participants through the study, including the number of participants with and without the outcome and, if applicable, a summary of the follow-up time. A diagram may be helpful.	Page 6; line 189
	13b	D;V	Describe the characteristics of the participants (basic demographics, clinical features, available predictors), including the number of participants with missing data for predictors and outcome.	Page 9; lines 267-276
	13c	V	For validation, show a comparison with the development data of the distribution of important variables (demographics, predictors and outcome).	Page 9; lines 275-276
Model development	14a	D	Specify the number of participants and outcome events in each analysis.	Page 9
	14b	D	If done, report the unadjusted association between each candidate predictor and outcome.	Page 13; lines 277-283
Model specification	15a	D	Present the full prediction model to allow predictions for individuals (i.e., all regression coefficients, and model intercept or baseline survival at a given time point).	Not done
	15b	D	Explain how to the use the prediction model.	Page 8; lines 246-250

For peer review only

For peer review only - http://bmjopen.bmj.com/site/about/guidelines.xhtml

TRIPOD

TRIPOD Checklist: Prediction Model Development and Validation

Model performance	16	D;V	Report performance measures (with CIs) for the prediction model.	Pages 13-18; lines 284-288; lines 316-321; lines 345-350
Model-updating	17	V	If done, report the results from any model updating (i.e., model specification, model performance).	Not relevant
**Discussion**				
Limitations	18	D;V	Discuss any limitations of the study (such as nonrepresentative sample, few events per predictor, missing data).	Page 21; lines 413-426
Interpretation	19a	V	For validation, discuss the results with reference to performance in the development data, and any other validation data.	Page 21-23; lines 443-461 & 485-493
	19b	D;V	Give an overall interpretation of the results, considering objectives, limitations, results from similar studies, and other relevant evidence.	Page 21-23; lines 427-493
Implications	20	D;V	Discuss the potential clinical use of the model and implications for future research.	Page 21-23; lines 427-493
**Other information**				
Supplementary information	21	D;V	Provide information about the availability of supplementary resources, such as study protocol, Web calculator, and data sets.	Page 27; lines 634-636
Funding	22	D;V	Give the source of funding and the role of the funders for the present study.	Page 27; lines 631-633

*Items relevant only to the development of a prediction model are denoted by D, items relating solely to a validation of a prediction model are denoted by V, and items relating to both are denoted D;V. We recommend using the TRIPOD Checklist in conjunction with the TRIPOD Explanation and Elaboration document.

For peer review only

BMJ Open

Assessing the capacity of symptom scores to predict COVID-19 positivity in Nigeria: a national derivation and validation cohort study

Journal:	*BMJ Open*
Manuscript ID	bmjopen-2021-049699.R2
Article Type:	Original research
Date Submitted by the Author:	03-Aug-2021
Complete List of Authors:	Elimian, Kelly; Nigeria Centre for Disease Control; Karolinska Institutet, Department of Global Public Health<br>Aderinola, Olaolu; Nigeria Centre for Disease Control<br>Gibson, Jack; University of Nottingham, Department of Epidemiology and Public Health<br>Myles, Puja; University of Nottingham Faculty of Medicine and Health Sciences, Epidemiology & Public Health<br>Ochu, Chinwe; Nigeria Centre for Disease Control<br>King, Carina; Karolinska Institute, Department of Global Public Health<br>Okwor, Tochi; Nigeria Centre for Disease Control, Prevention Programmes and Knowledge Management<br>Gaudenzi, Giulia; Karolinska Institutet, Department of Global Public Health<br>Olayinka, Adebola; World Health Organization<br>Zaiyad, Habib; University of Abuja Teaching Hospital<br>Ohonsi, Cornelius; Nigeria Centre for Disease Control<br>Ebhodaghe, Blessing; Nigeria Centre for Disease Control<br>Dan-Nwafor, Chioma; Nigeria Centre for Disease Control<br>Nwachukwu , William ; Nigeria Centre for Disease Control<br>Abdus-salam, Ismail; Lagos State Ministry of Health<br>Akande, Oluwatosin; University of Ilorin Teaching Hospital, Department of Epidemiology and Community Health<br>Falodun, Olanrewaju; National Hospital, Department of Internal Medicine<br>Arinze, Chinedu; Nigeria Centre for Disease Control<br>Ezeokafor, Chidiebere; National Agency for the Control of AIDS<br>Jafiya, Abubakar; Nigeria Centre for Disease Control<br>Ojimba, Anastacia; Federal Medical Centre Asaba<br>Aremu, John; Federal Teaching Hospital Gombe<br>Joseph, Emmanuel; Kaduna State Infectious Disease Control Center Community Medicine<br>Bowale, Abimbola; Mainland Hospital Yaba<br>Mutiu, Bamidele; Ministry of Health, Lagos State, Nigeria<br>Saka, Babatunde; Lagos State Government Ministry of Health<br>Jinadu, Arisekola; Nigeria Centre for Disease Control<br>Hamza, Khadeejah; Ahmadu Bello University, Department of Community Medicine<br>Ibeh, Christian; Nnamdi Azikiwe University Teaching Hospital, Department of Community Medicine<br>Bello, Shaibu; Usmanu Danfodiyo University, Department of Medical

	Education<br>Asuzu, Michael; University College Hospital, Department of Community Medicine<br>Mba, Nwando; Nigeria Centre for Disease Control<br>Oladejo, John; Nigeria Centre for Disease Control<br>Ilori, Elsie; Nigeria Centre for Disease Control<br>Alfvén, Tobias ; Karolinska Institutet, Department of Global Public Health<br>Igumbor, Ehimario; Nigeria Centre for Disease Control; University of the Western Cape, School of Public Health<br>Ihekweazu, Chikwe; Nigeria Centre for Disease Control
**Primary Subject Heading**:	Epidemiology
Secondary Subject Heading:	Global health, Infectious diseases, Public health
Keywords:	COVID-19, Public health < INFECTIOUS DISEASES, Epidemiology < TROPICAL MEDICINE

SCHOLARONE™
Manuscripts

I, the Submitting Author has the right to grant and does grant on behalf of all authors of the Work (as defined in the below author licence), an exclusive licence and/or a non-exclusive licence for contributions from authors who are: i) UK Crown employees; ii) where BMJ has agreed a CC-BY licence shall apply, and/or iii) in accordance with the terms applicable for US Federal Government officers or employees acting as part of their official duties; on a worldwide, perpetual, irrevocable, royalty-free basis to BMJ Publishing Group Ltd ("BMJ") its licensees and where the relevant Journal is co-owned by BMJ to the co-owners of the Journal, to publish the Work in this journal and any other BMJ products and to exploit all rights, as set out in our licence.

The Submitting Author accepts and understands that any supply made under these terms is made by BMJ to the Submitting Author unless you are acting as an employee on behalf of your employer or a postgraduate student of an affiliated institution which is paying any applicable article publishing charge ("APC") for Open Access articles. Where the Submitting Author wishes to make the Work available on an Open Access basis (and intends to pay the relevant APC), the terms of reuse of such Open Access shall be governed by a Creative Commons licence – details of these licences and which Creative Commons licence will apply to this Work are set out in our licence referred to above.

Other than as permitted in any relevant BMJ Author's Self Archiving Policies, I confirm this Work has not been accepted for publication elsewhere, is not being considered for publication elsewhere and does not duplicate material already published. I confirm all authors consent to publication of this Work and authorise the granting of this licence.

For peer review only

Assessing the capacity of symptom scores to predict COVID-19 positivity in Nigeria: a national derivation and validation cohort study

Kelly Elimian[1,2,3,4], Olaolu Aderinola[1], Jack Gibson[5], Puja Myles[5], Chinwe Ochu[1‡], Carina King[2], Tochi Okwor[1], Giulia Gaudenzi[2], Adebola Olayinka[6], Habib Zaiyad[7], Cornelius Ohonsi[1], Blessing Ebhodaghe[1], Chioma Dan-Nwafor[1], William Nwachukwu[1], Ismail Abdus-Salam[8], Oluwatosin Akande[9], Olanrewaju Falodun[10], Chinedu Arinze[1], Chidiebere Ezeokafor[11], Jafiya Abubakar[1], Anastacia Ojimba[12], John Aremu[13], Emmanuel Joseph[14], Abimbola Bowale[15], Bamidele Mutiu[8], Babatunde Akeem Saka[8], Arisekola Jinadu[1], Khadeejah Hamza[16], Christian Ibeh[17], Shaibu Oricha Bello[18], Michael Asuzu[19], Nwando Mba[1], John Oladejo[1], Elsie Ilori[1], Tobias Alfven[2], Ehimario Igumbor[1], Chikwe Ihekweazu[1]

[1]Nigeria Centre for Disease Control, Abuja, Nigeria
[2]Department of Global Public Health, Karolinska Institutet, Stockholm, Sweden
[3]Department of Microbiology, University of Benin, Edo State, Nigeria
[4]Nigeria COVID-19 Research Consortium, Abuja, Nigeria
[5]Department of Epidemiology and Public Health, University of Nottingham, UK
[6]World Health Organization, Abuja, Nigeria
[7]University of Abuja Teaching Hospital, Nigeria
[8]Ministry of Health, Lagos State, Nigeria
[9]University of Ilorin Teaching Hospital, Kwara State, Nigeria
[10]Department of Internal Medicine, National Hospital, Abuja, Nigeria
[11]National Agency for the Control of AIDS, Abuja, Nigeria
[12]Federal Medical Centre, Asaba, Delta State, Nigeria
[13]Federal Teaching Hospital Gombe, Gombe State, Nigeria
[14]Kaduna State Infectious Disease Control Center, Community Medicine, Kaduna State, Nigeria
[15]Mainland Hospital Yaba, Lagos State, Nigeria
[16]Department of Community Medicine, Ahmadu Bello University, Kaduna State, Nigeria
[17]Department of Community Medicine, Nnamdi Azikiwe University Teaching Hospital, Nigeria
[18]College of Health Sciences, Usmanu Danfodiyo University, Sokoto State, Nigeria
[19]Department of Community Medicine, University College Hospital, Oyo State, Nigeria.

‡Corresponding author and email address
Dr Kelly Elimian
kellyelimian@gmail.com

For peer review only - http://bmjopen.bmj.com/site/about/guidelines.xhtml

Abstract

Objectives: This study aimed to develop and validate a symptom prediction tool for COVID-19 test positivity in Nigeria.

Design: Predictive modelling study.

Setting: All Nigeria States and the Federal Capital Territory.

Participants: A cohort of 43,221 individuals within the national COVID-19 surveillance dataset from February 27 to August 27, 2020. Complete dataset was randomly split into two equal halves: derivation and validation datasets. Using the derivation dataset (n=21,477), backward multivariable logistic regression approach was used to identify symptoms positively associated with COVID-19 positivity (by RT-PCR) in children (≤17 years), adults (18-64 years) and elderly (≥65 years) patients separately.

Outcome measures: Weighted statistical and clinical scores based on beta regression coefficients and clinicians' judgements, respectively. Using the validation dataset (n=21,744), Area Under the Receiver Operating Characteristic curve (AUROC) values were used to assess the predictive capacity of individual symptoms, un-weighted score and the two weighted scores.

Results: Overall, 27.6% of children (4,415/15,988), 34.6% of adults (9,154/26,441), and 40.0% of elderly (317/792) that had been tested were positive for COVID-19. Best individual symptom predictor of COVID-19 positivity was loss of smell in children (AUROC 0.56, 95% CI: 0.55-0.56), either fever or cough in adults (AUROC 0.57, 95% CI: 0.56-0.58), and difficulty in breathing in the elderly (AUROC 0.53, 0.48-0.58) patients. In children, adults, and the elderly patients all scoring approaches showed similar predictive performance.

Conclusions: The predictive capacity of various symptom scores for COVID-19 positivity was poor overall. However, the findings could serve as an advocacy tool for more investments in resources for capacity strengthening of molecular testing for COVID-19 in Nigeria.

Keywords

Diagnosis; Symptoms; COVID-19; Predictive; Nigeria

Strengths and limitations of this study

- This study provides the early evidence on the predictive capacity of symptom scores for COVID-19 positivity in a sub-Saharan African country.

For peer review only - http://bmjopen.bmj.com/site/about/guidelines.xhtml

- The study utilised the train-test split-sample method to randomly split the analysed dataset into two (i.e. training and testing datasets), thereby allowing for validation of the generated clinical scores.
- The study adopted a participatory approach in the derivation of clinically weighted score by engaging clinicians managing COVID-19 patients, thereby enhancing the clinical relevance of the findings.
- Splitting the dataset for both derivation and validation of symptom scores might have underestimated the predictive performance of the models due to loss of power.
- The study lacked sufficient clinical laboratory data that have been shown to be significant in designing COVID-19 diagnostic models.

For peer review only

Introduction

The index case of COVID-19 in Nigeria was recorded on February 27, 2020, in Ogun State; one of the contacts of the index case was later diagnosed with the disease in the same state. As of epidemiological week 44 (October 26–November 1), Nigeria had recorded 62,964 confirmed cases, of whom 1,146 died and 58,790 recovered, resulting in a case fatality rate of 1.8%.[1] The rapid increase in COVID-19 cases in Nigeria caused a strain on the healthcare systems, particularly in the area of molecular testing using real-time polymerase chain reaction (RT-

For peer review only - http://bmjopen.bmj.com/site/about/guidelines.xhtml

PCR). Deficiencies in molecular laboratory capacity can hinder the rapid identification of persons with COVID-19 infection and initiation of appropriate treatment and public health measures.[2] For example, as of April 9, 2020, only 5,000 tests for Severe Acute Respiratory Syndrome Coronavirus 2 (SARS-CoV-2) were performed across Nigeria by eight laboratories across six States.[3] Concerted efforts have since been made to expand molecular testing capacity across the country, with 673,183 tests for SARS-CoV-2 conducted and the Nigeria Centre for Disease Control (NCDC) COVID-19 laboratory networks expanded to 69 functioning laboratories as of November 6, 2020.[4] However, with a population of over 200 million and community transmission of COVID-19, the testing rate for Nigeria to date is still well below the Africa CDC-set target of 1% for population. Generally, laboratory capacity to test for SARS-CoV-2 remains a challenge in many sub-Saharan African countries, especially in the early phase of COVID-19 pandemic.[5]

Although PCR testing is considered the gold-standard for COVID-19 diagnosis, limited access and centralised laboratory systems often contribute to delay.[6] This has contributed to making diagnostic results unavailable for urgent decision-making by frontline healthcare workers, with consequent effect on healthcare delivery and increased risk of nosocomial infection.[7] Between February 27 and June 6, 2020, for example, the median turn-around time for laboratory diagnosis of COVID-19 in Nigeria was 2 (IQR: 1-4) days; 9% of the 12,289 confirmed COVID-19 cases during this period was recorded among healthcare workers.[8] Thus, to mitigate the impact of COVID-19 on the healthcare system, while also improving community surveillance and care for patients, rapid and accurate diagnosis are crucial.[9] NCDC and its technical partners are currently conducting a study to validate rapid diagnostic test kits to determine their accuracy and clinical utility in Nigeria.

One approach to supplement laboratory testing is however the use of diagnostic prediction models that utilise clinical variables (including standard blood counts when available) to estimate the likelihood of individuals being COVID-19 positive or experiencing poor clinical outcome.[9] A systematic review of developed and/or validated prediction models found the most frequently reported predictors of COVID-19 diagnosis to be flu-like symptoms (e.g. chills, fatigue) and neutrophil count, with c-statistic estimates ≥0.90 (an excellent discriminative performance).[9] However, to date, these prediction models have been judged to be at a high risk of bias, largely because they were fitted on data that were not representative of the target population, and have suffered from poor reporting on intended use of the models or their calibration performance. While the reported performances of models to date are encouraging,

For peer review only - http://bmjopen.bmj.com/site/about/guidelines.xhtml

their adoption in clinical practice have not been recommended.[9] In addition, there has been a death of evidence from sub-Saharan Africa as the majority of available models use data from China and European countries, thus limiting their generalisability to settings such as Nigeria with different COVID-19 epidemiology, health care systems, socioeconomic conditions, and health-seeking behaviours.

The majority of confirmed COVID-19 cases in Nigeria are asymptomatic at the point of diagnosis[8]; for the confirmed cases who present with symptoms the most common symptoms tend to be non-specific (e.g. fever, cough and difficulty in breathing).[8,10,11] Using data from February 27 to June 6, 2020, presentation with cough, fever, loss of smell and loss of taste was found to be positively and independently associated with COVID-19 positivity in Nigeria[12]. But the dearth of evidence on the predictive capacity of clinical symptoms to predict COVID-19 in Nigeria (and indeed in sub-Saharan Africa) is the rationale for this study. Thus, we aimed to develop and validate the predictive capacity of clinical signs and symptoms with regards to testing positive for COVID-19 and to investigate whether there are any gains in the predictive capacity of statistically- and clinically-derived weighted combined symptom scores, as compared to an un-weighted combined score in Nigeria.

Methods

Study design and data source

This is a predictive modelling study using a retrospective cohort of persons enrolled in the Surveillance, Outbreak Response Management and Analysis System (SORMAS) database from February 27 to August 27, 2020. A detailed description of SORMAS in the COVID-19 context in Nigeria is available elsewhere.[8] Briefly, SORMAS is an open-source real-time electronic health surveillance and laboratory database, which has been in use in Nigeria since 2017. SORMAS is hosted and coordinated at the NCDC's headquarter in Abuja.

Study participants

The study population comprised children (≤17 years), adults (18-64 years) and the elderly (≥65 years) who were tested for SARS-CoV-2 infection. Eligibility for RT-PCR test during the study period was based on the NCDC COVID-19 standard case definition for suspected cases (Supplementary Table 1).[13] However, provisions were made to test persons concerned about COVID-19 infection upon presentation to designated testing centres regardless of whether they met the NCDC case definitions or not.

For peer review only - http://bmjopen.bmj.com/site/about/guidelines.xhtml

Data collection and management

Sociodemographic (e.g. age, sex, occupation, education and geopolitical zone of residence) and clinical presentation (signs and symptoms in the 14 days prior to testing) were collected from all persons tested. The collection and transportation of respiratory samples (oropharyngeal and nasopharyngeal swabs) for laboratory analysis were facilitated by trained healthcare workers according to NCDC guidelines.[14] Testing for SARS-CoV-2 by RT-PCR was performed according with the WHO guidelines.[15] All the collected data across the country, including laboratory diagnostic outcomes, were submitted by trained healthcare workers to NCDC via the SORMAS application installed on tablets or laptops. Patients who tested positive for COVID-19 were managed either in a government health facility or at home, depending on the illness severity or availability of bed space at health facilities, while observing the NCDC case management guidelines.[14]

Since this was an analysis of a secondary dataset, there was no formal sample size calculation for this study; however, the study met the standard sample size requirement of 10 outcome events per degree of freedom in prediction models (e.g., 10 binary variables in the model require 100 COVID-19 positive cases). We used the train-test split-sample method to randomly split the complete dataset into two (i.e. training and testing datasets), assigning half of the records to either the training (derivation) or testing (validation) datasets based on a random number generated within the statistical software. However, we made four major assumptions to define the study eligibility criteria. First, we assumed that a missing variable was indicative of absence (i.e., record not present), supported by findings ($p<0.001$) from a chi-square test of association between two *ad hoc* variables 'any-missing' and 'any-absent'. Second, we decided that eligible study participants needed to have complete records for both age and sex since these are crucial demographic variables. Third, given the high proportion of asymptomatic COVID-19 cases in Nigeria,[8] the study participants needed to have had at least one symptom positively recorded (i.e. symptomatic). Lastly, analyses were performed separately for children, adults and the elderly, given the evidence supporting the age-dependence of COVID-19 symptom presentation, with children more likely to be asymptomatic than adults.[16,17] Figure 1 shows a flowchart showing the processes for selecting the study population as stated in the eligibility criteria (definitions of suspected and probable cases are available in the NCDC case definition [13]). All symptoms were coded as '1' if present and '0' if not. Sex was classified as a binary variable, with '1' and '0' indicative of male and female gender, respectively.

For peer review only - http://bmjopen.bmj.com/site/about/guidelines.xhtml

Figure 1 about here

Outcome and predictor variables

The outcome was COVID-19 positivity, defined as either presence or absence of SARS-CoV-2 by RT-PCR confirmation (yes/no). Clinical prediction variables were informed by evidence from our previous study using the same dataset, though it covered a shorter period (February 27 to June 5, 2020).[12] The clinical variables were collected using a combination of self-reports by COVID-19 suspected cases and/or their caretakers as well as by objective assessment by a healthcare worker. The three categories of clinical signs and symptoms (each coded yes/no) with their respective examples include: 1. "obvious or as observed by a healthcare worker" (chills/sweat, cough, breathing difficulty, rapid breathing and runny nose); 2. "elicitable or information volunteered or given upon inquiry by patients/relatives" (abdominal pain/diarrhoea, gastrointestinal tract symptoms, chest pain, fatigue/weakness, headache, musculoskeletal pain, sore throat, loss of taste, and loss of smell); 3. and "measurable as assessed by a healthcare worker" (fever, defined as an axillary temperature ≥37.5°C).

Statistical analyses

The data were set up and analysed using Stata 13.0 (Stata Corporation, College Station, TX, USA).

Model development

Using the derivation dataset, associations between individual symptoms and COVID-19 positivity was investigated, in turn, using univariable logistic regression. Analyses were stratified by children, adults, and the elderly. The results are presented as unadjusted odds ratios (ORs) and 95% confidence intervals (95% CI). To determine which of the symptoms were independently associated with COVID-19 positivity, we first conducted a test for multicollinearity by the various age groups (children, adults, and the elderly) using the variance inflation factor (VIF) and tolerance values of each statistically significant symptom identified from the univariable logistic regression model (see Supplementary Table 2a, 2b and 2c for results). This was to ensure that symptoms were independent of each other or not collinear and therefore suitable for combining into a single score. Both VIF and tolerance values measure how much the regression coefficient for a symptom is determined by the other symptoms in the model; low VIF and high tolerance values suggest the absence of multicollinearity and vice-versa.[18] Thereafter we used the backward multivariable logistic regression approach to select

For peer review only - http://bmjopen.bmj.com/site/about/guidelines.xhtml

all the statistically significant symptoms for the model. As before, analyses were conducted for children, adults, and the elderly separately.

We then created three different symptom scores. First, an un-weighted combined score was calculated for each person (separately for children, adults, and the elderly) by allocating ‘1’ point for each symptom before summation. The un-weighted combined scores were included in the model as a continuous variable. The combined scores were further recoded as binary categorical variables to represent clinical scores≥1 (yes/no), ≥2 (yes/no), ≥3 (yes/no), and ≥4 (yes/no). Additionally, weighted combined scores were developed using two approaches. First, we used the beta regression coefficients obtained from the multivariable logistic regression model that was run previously. Each symptom was given the value of ‘1’ if present and ‘0’ if otherwise. Each symptom was then multiplied by its weight, obtained by multiplying the respective beta regression coefficients (or log of the ORs) by 10. Essentially, the beta regression coefficients measure the relative prognostic strength of each symptom when they are included simultaneously in a multivariable regression model so that the bigger the value of a symptom, the more its weight. Second, we engaged six clinicians (including infectious disease consultants) who have been managing COVID-19 patients in treatment centres and at home in the FCT, Gombe, Delta, and Kaduna. Here, the clinicians were provided with a list of symptoms identified to be statistically significant from the univariable logistic models, with the freedom to add any missing sign or symptom in the model as needed. Each clinician was then asked to independently assign a weight of 1-5 for each symptom based on experiences from managing COVID-19 patients (see Supplementary Table 3 for detail of weighted scores assigned by each clinician for the identified symptoms). Similar to the statistical weighting approach, each symptom was multiplied by the average of the combined weights assigned by all the clinicians. Predictive capacity of the various score thresholds was also compared to see which combination of symptoms was more predictive of COVID-19 positivity.

Model validation

The different models were then applied to the validation dataset. Separately for children, adults and the elderly, predictive capacity was assessed in terms of sensitivity, specificity, Area under the Receiver Operating Characteristic (ROC) curve (AUROC) value, positive predictive value (PPV) and negative predictive value (NPV);[19] and ROC curves were plotted for the comparison of clinical prediction scores.

For peer review only - http://bmjopen.bmj.com/site/about/guidelines.xhtml

Where applicable, this study is reported according to the TRIPOD (Transparent Reporting of a multivariable prediction model for Individual Prognosis Or Diagnosis) statement.[20]

External calibration belt and test for model goodness-of-fit

Using the validation dataset, external calibration test and belt were used to evaluate the goodness-of-fit of our models, an approach that examines the relationship between estimated probabilities and observed outcome rates[21]. Tests and belts often return concordant outputs: non-significant tests are often associated with the belt encompassing the 45-degree lines (good fit) and significant tests with the belt deviating from the bisector (poor fit).

Ethics and ethical considerations

The Nigeria National Health Research Ethics Committee approved the use of the SORMAS database for this study (reference number: NHREC/01/01/2007-22/06/2020).

Patient and public involvement

Being an analysis of de-identified secondary dataset, it was not possible to involve patients or the public in the design, or conduct, or reporting, or dissemination plans of this study. However, clinicians who have been managing COVID-19 patients both in health facilities and at home in Nigeria were actively engaged both in planning the study and in developing the clinical scores.

Results

Description of study population

The baseline sociodemographic and clinical characteristics of the study participants in the combined datasets are presented in Table 1. Overall, 15,988 children met the study eligibility criteria, 4,415 (27.6%) of whom tested positive for COVID-19 and 62.2% were male. A total of 26,441 adults met the study eligibility criteria, 9,154 (34.6%) of whom tested positive for COVID-19, 64.0% (16,819/26,441) were male, and 11.3% (2,977/26,441) were healthcare workers. Seven hundred and ninety-two elderly patients met the study eligibility criteria, 317

For peer review only

(40.0%) of whom tested positive for COVID-19 and 62.3% were males. Similar characteristics were observed in the derivation (Supplementary Table 4) and validation (Supplementary Table 5) cohorts for all age-groups.

For peer review only

For peer review only - http://bmjopen.bmj.com/site/about/guidelines.xhtml

Table 1: Baseline sociodemographic and clinical characteristics of all the study participants in relation to COVID-19 infection (combined dataset)

Variable	Children (≤17 years)		Adults (18-64 years)		Elderly (≥65 years)	
	PCR-confirmed cases [n=4,415 (%)]	PCR positive and negative cases [N=15,988 (%)]	PCR-confirmed cases [n=9,154 (%)]	PCR positive and negative cases [N=26,441 (%)]	PCR-confirmed cases [n=317 (%)]	PCR positive and negative cases [N=792 (%)]
**Socio-demographic features**						
**Sex**						
Female	1,773 (40.16)	6,051 (37.85)	3,080 (33.65)	9,622 (36.39)	110 (34.70)	299 (37.75)
Male	2,642 (59.84)	9,937 (62.15)‡	6,074 (66.35)	16,819 (63.61)‡	207 (65.30)	493 (62.25)NS
**Geopolitical zone§**						
South-west	1,302 (29.49)	3,539 (22.14)	2,592 (28.32)	6,245 (23.62)	86 (27.13)	185 (23.36)
South-south	1,435 (32.50)	5,689 (35.58)	3,228 (35.26)	10,911 (41.27)	108 (34.07)	340 (42.93)
South-east	90 (2.04)	278 (1.74)	273 (2.98)	609 (2.30)	25 (7.89)	37 (4.67)
North-central	816 (18.48)	3,056 (19.11)	1,526 (16.67)	4,061 (15.36)	30 (9.46)	76 (9.60)
North-west	658 (14.90)	3,118 (19.50)	1,258 (13.72)	4,005 (15.15)	45 (14.20)	116 (14.65)
North-east	114 (2.58)	308 (1.93)‡	277 (3.03)	610 (2.31)‡	23 (7.26)	38 (4.80)‡
**Setting**						
Rural	230 (5.21)	887 (5.55)	511 (5.58)	1,562 (5.91)	35 (11.04)	74 (9.34)
Urban	2,048 (46.39)	6,943 (43.43)	4,487 (49.02)	13,088 (49.50)	183 (57.73)	423 (53.41)
Missing	2,137 (48.40)	8,158 (51.03)‡	4,156 (45.40)	11,791 (44.59)NS	99 (31.23)	295 (37.25)†
**Education**						
None	58 (1.31)	217 (1.36)	128 (1.40)	368 (1.39)	25 (7.89)	48 (6.06)
Nursery	30 (0.68)	143 (0.89)	3 (0.03)	7 (0.03)	0 (0.00)	0 (0.00)
Primary	130 (2.94)	520 (3.25)	102 (1.11)	322 (1.22)	8 (2.52)	18 (2.27)
Secondary	385 (8.72)	1,379 (8.63)	792 (8.65)	2,262 (8.55)	23 (7.26)	58 (7.32)
Tertiary	1,410 (31.94)	4,066 (25.43)	3,141 (34.31)	8,145 (30.80)	87 (27.44)	168 (21.21)
Other	179 (4.05)	1,065 (6.66)	261 (2.85)	688 (2.60)	20 (6.31)	45 (5.68)
Missing	2,223 (50.35)	8,598 (53.78)‡	4,727 (51.64)	14,649 (55.40)‡	154 (48.58)	455 (57.45)†
**Occupation**						
Student/pupil	599 (13.57)	2,809 (17.57)	434 (4.74)	1,516 (5.73)	1 (0.32)	2 (0.25)
Child/housewife	133 (3.01)	489 (3.06)	178 (1.94)	500 (1.89)	13 (4.10)	42 (5.30)
Business/trading	217 (4.92)	765 (4.78)	757 (8.27)	2,043 (7.73)	19 (5.99)	47 (5.93)
Transporter	11 (0.25)	80 (0.50)	45 (0.49)	174 (0.66)	0 (0.00)	3 (0.38)
Healthcare worker	601 (13.61)	1,953 (12.22)	973 (10.63)	2,977 (11.26)	7 (2.21)	20 (2.53)
Laboratorian	15 (0.34)	40 (0.25)	26 (0.28)	63 (0.24)	0 (0.00)	0 (0.00)
Farmer	36 (0.82)	192 (1.20)	173 (1.89)	589 (2.23)	24 (7.57)	47 (5.93)
Animal-related worker	8 (0.18)	28 (0.18)	25 (0.27)	80 (0.30)	1 (0.32)	9 (1.14)
Religious/traditional leader	5 (0.11)	24 (0.15)	53 (0.58)	129 (0.49)	6 (1.89)	9 (1.14)

For peer review only - http://bmjopen.bmj.com/site/about/guidelines.xhtml

Other	1,633 (36.99)	5,126 (32.06)	4,072 (44.48)	11,408 (43.15)	164 (51.74)	375 (47.35)
Missing	1,157 (26.21)	4,482 (28.03)‡	2,418 (26.41)	6,962 (26.33)‡	82 (25.87)	238 (30.05)NS
**Clinical signs and symptoms**						
**Clinical outcome**						
Recovered	2,847 (64.48)	4,443 (27.79)	5,694 (62.20)	7,313 (27.66)	150 (47.32)	184 (23.23)
Dead	36 (0.82)	51 (0.32)	485 (5.30)	549 (2.08)	83 (26.18)	84 (10.61)
No outcome yet	1,532 (34.70)	11,494 (71.89)‡	2,975 (32.50)	18,579 (70.27)‡	84 (26.50)	524 (66.16)‡
Obvious (visible to healthcare workers on sight)						
**Chills/sweat**						
No	4,355 (98.64)	15,764 (98.60)	9,009 (98.42)	26,029 (98.44)	315 (99.37)	783 (98.86)
Yes	60 (1.36)	224 (1.40)NS	145 (1.58)	412 (1.56)NS	2 (0.63)	9 (1.14)NS
**Cough**						
No	2,345 (53.11)	9,393 (58.75)	4,130 (45.12)	14,391 (54.43)	114 (35.96)	321 (40.53)
Yes	2,070 (46.89)	6,595 (41.25)‡	5,024 (54.88)	12,050 (45.57)‡	203 (64.04)	471 (59.47)†
**Breathing difficulty**						
No	3,936 (89.15)	14,283 (89.34)	7,549 (82.47)	22,425 (84.81)	200 (63.09)	541 (68.31)
Yes	479 (10.85)	1,705 (10.66)NS	1,605 (17.53)	4,016 (15.19)‡	117 (36.91)	251 (31.69)†
**Rapid breathing**						
No	4,345 (98.41)	15,783 (98.72)	9,002 (98.34)	26,112 (98.76)	307 (96.85)	771 (97.35)
Yes	70 (1.59)	205 (1.28)†	152 (1.66)	329 (1.24)‡	10 (3.15)	21 (2.65)NS
**Runny nose**						
No	3,057 (69.24)	11,835 (74.02)	6,792 (74.20)	20,496 (77.52)	259 (81.70)	658 (83.08)
Yes	1,358 (30.76)	4,153 (25.98)‡	2,362 (25.80)	5,945 (22.48)‡	58 (18.30)	134 (16.92)NS
Elicitable (can be found out by asking questions of patients/relatives)						
**Abdominal pain/diarrhoea**						
No	4,142 (93.82)	14,939 (93.44)	8,464 (92.46)	24,580 (92.96)	297 (93.69)	728 (91.92)
Yes	273 (6.18)	1,049 (6.56)NS	690 (7.54)	1,861 (7.04)NS	20 (6.31)	64 (8.08)NS
**GIT symptoms**						
No	3,972 (89.97)	14,375 (89.91)	8,100 (88.49)	23,766 (89.88)	282 (88.96)	705 (89.02)
Yes	443 (10.03)	1,613 (10.09)NS	1,054 (11.51)	2,675 (10.12)‡	35 (11.04)	87 (10.98)NS
**Chest pain**						
No	4,286 (97.08)	15,577 (97.43)	8,782 (95.94)	25,621 (96.90)	303 (95.58)	766 (96.72)
Yes	129 (2.92)	411 (2.57)NS	372 (4.06)	820 (3.10)‡	14 (4.42)	26 (3.28)NS
**Fatigue**						
No	4,143 (93.84)	15,229 (95.25)	8,583 (93.76)	25,014 (94.60)	292 (92.11)	750 (94.70)
Yes	272 (6.16)	759 (4.75)‡	571 (6.24)	1,427 (5.40)‡	25 (7.89)	42 (5.30)†
**Headache**						
No	3,753 (85.01)	13,712 (85.76)	7,849 (85.74)	23,283 (88.06)	287 (90.54)	738 (93.18)

Yes	662 (14.99)	2,276 (14.24)NS	1,305 (14.26)	3,158 (11.94)‡	30 (9.46)	54 (6.82)ŧ
**Musculoskeletal pain**						
No	4,299 (97.37)	15,595 (97.54)	8,901 (97.24)	25,783 (97.51)	306 (96.53)	771 (97.35)
Yes	116 (2.63)	393 (2.46)NS	253 (2.76)	658 (2.49)ŧ	11 (3.47)	21 (2.65)NS
**Sore throat**						
No	3,306 (74.88)	11,968 (74.86)	6,968 (76.12)	19,583 (74.06)	259 (81.70)	649 (81.94)
Yes	1,109 (25.12)	4,020 (25.14)NS	2,186 (23.88)	6,858 (25.94)‡	58 (18.30)	143 (18.06)NS
**Loss of taste**						
No	3,925 (88.90)	15,283 (95.59)	8,301 (90.68)	25,268 (95.56)	303 (95.58)	770 (97.22)
Yes	490 (11.10)	705 (4.41)‡	853 (9.32)	1,173 (4.44)‡	14 (4.42)	22 (2.78)ŧ
**Loss of smell**						
No	3,815 (86.41)	15,160 (94.82)	8,186 (89.43)	25,198 (95.30)	307 (96.85)	778 (98.23)
Yes	600 (13.59)	828 (5.18)‡	968 (10.57)	1,243 (4.70)‡	10 (3.15)	14 (1.77)ŧ
Measureable						
**Fever**						
No	2,350 (53.23)	9,430 (58.98)	4,555 (49.76)	15,434 (58.37)	163 (51.42)	453 (57.20)
Yes	2,065 (46.77)	6,558 (41.02)‡	4,599 (50.24)	11,007 (41.63)‡	154 (48.58)	339 (42.80)ŧ

ŧ=p-value<0.05; ‡=p<0.001; NS=p>0.05 or Not significant

GIT= gastrointestinal (nausea + vomiting)

Musculoskeletal pain= muscle /joint pain

§=State composition of geopolitical zones in Nigeria: South-West (Ekiti, Lagos, Ogun, Ondo, Osun and Oyo); South-South (Akwa-Ibom, Bayelsa, Cross-River, Rivers, Delta and Edo); South-East (Abia, Anambra, Ebonyi, Enugu and Imo); North-Central (Benue, Kogi, Kwara, Nasarawa, Niger, and Plateau States as well as the FCT); North-West (Jigawa, Kaduna, Kano, Katsina, Kebbi, Sokoto and Zamfara); and North-East (Adamawa, Bauchi, Borno, Gombe, Taraba and Yobe).

For peer review only - http://bmjopen.bmj.com/site/about/guidelines.xhtml

Performance of individual symptoms for predicting COVID-19 positivity

In the unadjusted model, presentation with cough, runny nose, fatigue, loss of taste, loss of smell, and fever was significantly associated with higher odds of COVID-19 positivity in children (left panel of Table 2). In the adjusted model, however, presentation with the following symptoms remained significantly ($p<0.001$) associated with higher odds of COVID-19 positivity: cough (adjusted OR 1.32, 95% CI: 1.19-1.47), runny nose (aOR 1.48, 95% CI: 1.32-1.66), fatigue (aOR 1.53, 95% CI: 1.21-1.93), loss of taste (aOR 2.26, 95% CI: 1.63-3.12), loss of smell (aOR 4.87, 95% CI: 3.66-6.49), or fever (aOR 1.47, 95% CI: 1.33-1.63). Regarding predictive performance, loss of smell recorded the highest AUROC value (0.56, 95% CI: 0.55-0.56). Specifically, cough recorded the highest sensitivity (47.7%, 95% CI: 45.6-49.7%) and NPV (74.7%, 95% CI: 73.4-75.9%), while loss of smell recorded the highest specificity (98.1%, 95% CI: 97.7-98.4%) and PPV (72.9%, 95% CI: 68.3-77.2%) (see details in Supplementary Table 6).

The presentation of symptoms significantly associated with increased COVID-19 positivity was higher in adults than in children (middle panel of Table 2). In the adjusted model, however, it was only the presentation with cough (aOR 1.77, 95% CI: 1.64-1.91), runny nose (aOR 1.18, 95% CI: 1.08-1.30), chest pain (aOR 1.50, 95% CI: 1.21-1.85), fatigue (aOR 1.39, 95% CI: 1.17-1.64), headache (aOR 1.24, 95% CI: 1.11-1.39), loss of taste (aOR 2.33, 95% CI: 1.83-2.96), loss of smell (aOR 4.18, 95% CI: 3.30-5.28), or fever (aOR 1.58, 95% CI: 1.47-1.71) that remained independently associated with COVID-19 positivity. Regarding predictive performance, presentation with either fever or cough appeared to be the best predictor of COVID-19 positivity (AUROC 0.57, 95% CI: 0.56-0.58). Additionally, presenting with cough or fever recorded the highest sensitivity at 54.3% (52.9-55.8%) and NPV at 70.8% (69.7-71.8%); however, presenting with loss of smell recorded the highest specificity (98.5%; 95% CI: 98.2-98.8%) and PPV (79.4%, 95% CI: 76.0-82.5%) (see details in Supplementary Table 7).

For peer review only - http://bmjopen.bmj.com/site/about/guidelines.xhtml

Table 2: Associations and predictive performance of individual clinical characteristics of the study participants in relation to COVID-19 positivity (based on derivation datasets)

Variable	Children [n=7,911]			Adults [n=13,167]			Elderly [n=399]		
	**Unadjusted OR (95% CI)**	**Adjusted OR (95% CI)***	**AUROC (95% CI)§**	**Unadjusted OR (95% CI)**	**Adjusted OR (95% CI)‡**	**AUROC (95% CI)§**	**Unadjusted OR (95% CI)**	**Adjusted OR (95% CI)‡**	**AUROC (95% CI)§**
Obvious (visible to healthcare workers on sight)									
**Chills/sweat**									
No	1.00			1.00			1.00		
Yes	1.15 (0.77-1.70)NS			1.14 (0.85-1.54)NS			0.30 (0.03-2.57)NS		
**Cough**						**0.57**			
No	1.00	1.00	0.54	1.00	1.00	**(0.56-0.58)**	1.00	1.00	0.52
Yes	1.39 (1.26-1.53)‡	**1.32 (1.19-1.47)**‡	(0.53-0.55)	1.85 (1.72-1.99)‡	**1.77 (1.64-1.91)**‡		1.61 (1.06-2.44)†	1.59 (1.04-2.43)†	(0.47-0.57)
**Breathing difficulty**									
No	1.00			1.00	1.00		1.00	1.00	**0.53**
Yes	1.02 (0.87-1.19)NS			1.23 (1.11-1.35)‡	1.10 (0.99-1.22)NS		1.71 (1.11-2.66)†	1.74 (1.12-2.72)†	**(0.48-0.58)**
**Rapid breathing**									
No	1.00			1.00	1.00		1.00		
Yes	1.11 (0.73-1.68)NS			1.58 (1.15-2.16)†	1.24 (0.89-1.73)NS		2.04 (0.45-9.23)NS		
**Runny nose**									
No	1.00	1.00	0.52	1.00	1.00	0.53	1.00		
Yes	1.53 (1.37-1.70)‡	**1.48 (1.32-1.66)**‡	(0.51-0.53)	1.30 (1.20-1.42)‡	**1.18 (1.08-1.30)**‡	(0.52-0.54)	1.12 (0.66-1.88)NS		
Elicitable (can be found out from patients' responses or by asking questions of relatives)									
**Abdominal**									
**pain/diarrhoea**				1.00					
No	1.00			1.12 (0.98-1.29)NS			1.00		
Yes	0.87 (0.71-1.07)NS						0.62 (0.26-1.44)NS		
**GIT symptoms**									
No	1.00			1.00	1.00		1.00		
Yes	0.96 (0.82-1.14)NS			1.17 (1.04-1.32)†	1.11 (0.98-1.26)NS		0.91 (0.45-1.83)NS		
**Chest pain**									
No	1.00			1.00	1.00	0.51	1.00		
Yes	1.33 (0.98-1.79)NS			1.75 (1.44-2.14)‡	**1.50 (1.21-1.85)**‡	(0.50-0.51)	1.53 (0.48-4.83)NS		
**Fatigue/weakness**									
No	1.00	1.00	0.51	1.00	1.00	0.51	1.00		
Yes	1.29 (1.03-1.62)†	**1.53 (1.21-1.93)**‡	(0.51-0.52)	1.25 (1.07-1.46)†	**1.39 (1.17-1.64)**‡	(0.50-0.51)	2.00 (0.90-4.35)NS		
**Headache**									
No	1.00			1.00	1.00	0.52	1.00		
Yes	1.12 (0.98-1.29)NS			1.44 (1.29-1.60)‡	**1.24 (1.11-1.39)**‡	(0.51-0.52)	0.67 (0.31-1.44)NS		
**Musculoskeletal pain**									
No	1.00			1.00			1.00		
Yes	1.09 (0.80-1.48)NS			1.04 (0.81-1.32)NS			1.80 (0.59-5.45)NS		

**Sore throat**									
No	1.00			1.00			1.00		
Yes	1.02 (0.91-1.14)NS			0.83 (0.76-0.90)‡			0.82 (0.50-1.35)NS		
**Loss of taste**									
No	1.00	1.00	0.54	1.00	1.00	0.54	1.00		
Yes	7.07 (5.59-8.95)	**2.26 (1.63-3.12)**‡	(0.54-0.55)	5.73 (4.75-6.91)‡	**2.33 (1.83-2.96)**‡	(0.53-0.54)	4.67 (0.93-23.42)NS		
**Loss of smell**									
No	1.00	1.00	**0.56**	1.00	1.00	0.55	1.00	1.00	0.51
Yes	7.85 (6.30-9.77)‡	**4.87 (3.66-6.49)**‡	**(0.55-0.56)**	6.74 (5.58-8.14)‡	**4.18 (3.30-5.28)**‡	(0.54-0.55)	5.48 (1.12-26.72)†	7.15 (1.44-35.44)†	(0.49-0.52)
Measureable									
**Fever**									
No	1.00	1.00	0.54	1.00	1.00	**0.57**	1.00		
Yes	1.43 (1.29-1.58)‡	**1.47 (1.33-1.63)**‡	(0.52-0.55)	1.64 (1.52-1.76)‡	**1.58 (1.47-1.71)**‡	**(0.56-0.58)**	1.42 (0.95-2.12)NS		

OR: Odds ratio; CI: Confidence Interval; significant results in the adjusted model are in bold fonts.
*: The fully-adjusted model includes all statistically significant variables from the unadjusted model.
§: Area under receiver operating characteristic curve; figures are for variable that were statistically significant in the adjusted model only; the best predictive AUROC value is highlighted in bold.
Wald's p-values: ‡<0.001; †<0.05; NS=Not significant (p>0.05)

For peer review only - http://bmjopen.bmj.com/site/about/guidelines.xhtml

Only three symptoms in the elderly patients were significantly associated with COVID-19 positivity both in the unadjusted and adjusted models, with cough (aOR 1.59, 95% CI: 1.04-2.43; p=0.033), difficulty in breathing (aOR 1.74, 95% CI: 1.12-2.72; p=0.015) or loss of smell (aOR 7.15, 95% CI: 1.44-35.44; p=0.016) being independently associated with COVID-19 positivity in the latter model (right panel of Table 2). Regarding predictive performance, presenting with difficulty in breathing appeared to be the best predictor of COVID-19 positivity (AUROC 0.53, 0.48-0.58). However, presenting with cough recorded the highest sensitivity (61.4%, 95% CI: 53.3-69.0%) and NPV (62.1%, 95% CI: 54.1-69.6%), while loss of smell recorded the highest specificity (99.1%, 95% CI: 97.0-99.9%) and PPV (60.0%, 95% CI: 14.7-94.7%) (see details in Supplementary Table 8).

Performance of un-weighted, statistically- and clinically-weighted scores for predicting COVID-19 positivity

In adults, the statistically-derived weighted combined score appeared to be slightly better in predicting COVID-19 positivity (AUROC: 0.65), when compared with both the clinically-derived weighted combined and un-weighted scores with AUROC 0.63 each (Table 3). A similar pattern was recorded in children and elderly populations. For individual symptom thresholds, presenting with ≥2 symptoms on the un-weighted score appeared to be a better predictor of COVID-19 positivity (AUROC 0.61, 95% CI: 0.60-0.61) than the other symptom thresholds in adults. For the statistically weighted score, presenting with ≥4 symptoms appeared to be a better predictor of COVID-19 positivity (AUROC 0.59, 95% CI: 0.58-0.59) than the other symptom thresholds in adults. For the clinically-derived weighted score, presenting with either ≥3 or ≥4 symptoms was a better predictor of COVID-19 positivity (AUROC 0.58, 95% CI: 0.58-0.59) in adults. The detailed results including sensitivity, specificity, PPV and NPV values for each age-group are available in Supplementary Tables 9, 10 and 11. Figure 2 shows the ROC curves comparing the predictive performance of unweighted, statistically- and clinically-derived weighted combined scores in children (2a), adult (2b) and the elderly (2c) populations.

For peer review only - http://bmjopen.bmj.com/site/about/guidelines.xhtml

Table 3: Predictive performance of un-weighted, statistically- and clinically-weighted score thresholds for predicting COVID-19 positivity in children, adults and elderly

Outcome	Score	Un-weighted score	Statically-weighted score	Clinically-weighted score
		AUROC value (95% CI)		
**Children (<17 years) n=8,077**				
**Combined score**		0.6064	0.6177	0.5915
**Symptom threshold**	≥1	0.55 (0.54-0.56)	0.55 (0.54-0.56)	0.55 (0.54-0.56)
	≥2	**0.58 (0.57-0.59)**	0.55 (0.54-0.56)	0.54 (0.53-0.55)
	≥3	0.55 (0.54-0.56)	0.55 (0.54-0.57)	0.56 (0.55-0.57)
	≥4	0.52 (0.52-0.53)	**0.59 (0.58-0.60)**	**0.57 (0.56-0.58)**
**Adults (17-64 years) n=13,274**				
**Combined score**		0.6333	0.6475	0.6389
**Symptom threshold**	≥1	0.56 (0.55-0.56)	0.56 (0.55-0.56)	0.56 (0.55-0.56)
	≥2	**0.61 (0.60-0.61)**	0.58 (0.57-0.58)	0.56 (0.55-0.56)
	≥3	0.56 (0.55-0.57)	0.58 (0.57-0.59)	**0.58 (0.58-0.59)**
	≥4	0.53 (0.53-0.54)	**0.59 (0.58-0.59)**	**0.58 (0.58-0.59)**
**Elderly (≥65 years) n=393**				
**Combined score**		0.5413	0.5453	0.5426
**Symptom threshold**	≥1	0.52 (0.48-0.57)	0.52 (0.48-0.57)	0.52 (0.48-0.57)
	≥2	**0.53 (0.49-0.57)**	0.52 (0.48-0.57)	0.52 (0.48-0.57)

The best predictive AUROC values for each age group are highlighted in bold

Figure 2 about here

External calibration belt and test for model goodness-of-fit

The calibration belt in the produced plots and tests for children (p=0.086), adults (p=0.915) and elderly (p=0.091) is presented in Figure 3 and suggest that the hypothesis of good calibration is not rejected. The calibration belt and tests for the unweighted, statistically- and clinically-weighted scores in children, adults, and elderly are presented in a Supplementary Figure. With the exception of unweighted and clinically weighted scores in children, all other scores showed good calibration.

Figure 3 about here

For peer review only

For peer review only - http://bmjopen.bmj.com/site/about/guidelines.xhtml

Discussion

Principal findings

In this study we developed and validated symptom's prediction scores for COVID-19 positivity, independently in children, adults, and the elderly patients in the Nigerian context. The best individual symptom predictors of COVID-19 positivity in children, adult, and the elderly patients were loss of smell (AUROC 0.56, 95% CI: 0.55-0.56), either fever or cough (AUROC 0.57, 95% CI: 0.56-0.58), and difficulty in breathing (AUROC 0.53, 0.48-0.58), respectively. In adults, all the symptom scores showed similar performance, with the statistically weighted score (AUROC: 0.65) slightly showing better performance than the un-weighted (AUROC: 0.63) and clinically-derived weighted (AUROC: 0.64) scores. Similar results were found in children and the elderly patients. Overall, none of the symptom scores had good enough discrimination to use in practice.

Strengths and limitations of this study

To the best of our knowledge, this is the first study to have developed and validated symptom prediction scores with a view to aiding prompt recognition of COVID-19 by frontline healthcare workers in the Nigerian context and possibly in sub-Sahara Africa at large. Despite the limited accuracy of the developed prediction tool, the findings are very important for a country with limited capacity for molecular diagnosis of COVID-19, as it provides the evidential basis for advocacy for more investments in molecular diagnostics by policymakers in Nigeria. We also adopted a transparent methodology and adhered to the TRIPOD reporting statement hence minimising the vagueness often associated with the reporting of studies on the predictive performance of diagnostic models for COVID-19.[9] The methodology taken to the derivation of weighted scores is also a strength of this study. In accordance with the preferred approach for building prediction models,[9] the participatory approach taken to deriving the clinically weighted score can enhance the study relevance in the medical community.[22] The use of beta regression coefficients as opposed to odds ratios in deriving the statistical weighted scores has the advantage of being less prone to bias by small to moderate sample size.[23] A common limitation of many COVID-19 diagnostic models is bias due to over-fitting of the models on data that are not representative of the target population.[9] By using SORMAS database (hosts data from all over the country) for both derivation and validation in the present study, our findings are considerably generalisable to the COVID-19 situation in Nigeria and less prone to overestimation of COVID-19 risk among individuals tested.

For peer review only - http://bmjopen.bmj.com/site/about/guidelines.xhtml

This study however has some limitations that warrant discussion. First, being an analysis of secondary data based on practical recording of routine clinical assessments, the fundamental assumption is that the data recorded on clinical symptoms are reasonably complete; for instance, we assumed that where a symptom was not recorded as being absent rather than missing. Without any objective means of verifying this assumption, any bias caused by misclassification of the individual symptoms could potentially minimise differences in comparisons, in which case observed differences are likely to be in the direction of the null hypothesis. Second, the approach of splitting the dataset for both derivation and validation of symptom scores may have lowered the precision of estimated effect (wider 95% CIs) [24] and potentially underestimated prediction performance due to loss of power;[25] Moreover, evidence supporting the 10 event per outcome rule of thumb has been found by Smeden *et al.*[26] to be weak. Third, the study lacked detailed clinical laboratory data, such as record for albumin or albumin/globin, direct bilirubin values, and red blood cell distribution width, which have been found to be significant variables in COVID-19 diagnostic models.[9] Practically, however, the time and technical requirements for testing these laboratory data could limit their clinical utility.

Interpretation & implications of findings

Based on the systematic and critical review of diagnostic scores by Wynant *et al.*, the performance of a diagnostic model is influenced by its composition, with higher number of clinical and laboratory parameters in a model indicating better predictive performance.[9] For instance, studies in China,[27,28] Brazil,[29] Italy,[30] the Netherlands[31] and France[32] with several clinical and laboratory parameters recorded excellent discriminatory performance, albeit with substantial evidence of bias[9] and limited clinical utility. Conversely, a prediction model (containing fewer number of symptoms, heart rate, systolic and diastolic blood pressure) developed by Sun *et al.*[33] in Singapore had a poor discriminatory capacity (C statistic: 0.65; 0.57-0.73). There is evidence to further suggest that the discriminatory accuracy of a prediction model, particularly its sensitivity, can be enhanced by including certain variables including a combination of loss of smell or taste and fever.[34] In the absence of comparison study from a sub-Saharan African country, it is difficult to fully explain the variation in the findings from the present study and elsewhere. Thus a follow-up study using both clinical and laboratory parameters in a Nigerian setting or in sub-Saharan Africa (with similar healthcare system and demographic structure) is recommended.

For peer review only - http://bmjopen.bmj.com/site/about/guidelines.xhtml

Prediction performance of the un-weighted score with regards to COVID-19 positivity was better in adults than in children and the elderly patients in our study, though the predictive capacity of all the scores was poor overall. This finding has an important implication on NCDC's current definition of COVID-19 suspected cases, which emphasises acute respiratory symptoms and either travel history within 14 days prior to symptom onset or self-reported contact with a confirmed case.[13] Given our findings are indicative of age-dependency of symptom, it may be useful to review the current case definitions of COVID-19 in Nigeria. For example, we found loss of smell and either fever or cough to be better in predicting COVID-19 positivity in children and adults, respectively, while breathing difficulty was more predictive of the disease in the elderly patients. Furthermore, this finding potentially has implications on the clinical utility of existing suspected case definition in Nigeria[13] with a high proportion of asymptomatic COVID-19 cases[8] and testing system that allows persons who are concerned about their COVID-19 risk to be tested. Thus, to minimise missed diagnoses and overburdening of the healthcare system, with attendant psychological effects on health personnel,[35] there is a need for more economic investments on molecular testing across Nigeria.

Loss of smell recorded the highest specificity with regards to COVID-19 positivity for the three age groups: 98.1% in children, 98.5% in adults and 99.1% in the elderly. However, unlike the present study which explored the predictive capacity of loss of smell and taste separately, a combination of both symptoms has been shown to be more predictive.[36] Thus, the potential use of both loss of smell and taste to differentiate COVID-19 from endemic febrile and respiratory illnesses in Nigeria, such as malaria and pneumonia, with overlapping symptoms warrant further study. Additionally, possibility of using both loss of smell and taste as early indicators of emerging COVID-19 wave or a surge in Nigeria would be useful in improving COVID-19 response, such as allocation of already limited testing resources, risk communication, and aid decision making concerning lockdowns and quarantines.[37] The poor predictive capacity of cough or fever alone in the present study is congruent with that in a meta-analysis.[38]

Clinical validity (characterised by sensitivity, specificity and AUROC values) is an important criterion for assessing a clinical prediction tool[39] as it is —the ability of the prediction tool to distinguish between who has an outcome (in this case SARS-CoV-2 infection) and who does not.[40] The clinical validity of all our prediction scores was generally poor but appeared to be dependent on the number of symptoms. For instance, in our study, the un-weighted and weighted (both statistical and clinical) predictive scores presenting with fewer number of symptoms were more sensitive compared with many symptoms in children and adults; it was

For peer review only - http://bmjopen.bmj.com/site/about/guidelines.xhtml

however the opposite relative to specificity given ≥4 symptoms recorded higher specificity values than lower symptom thresholds. The poor sensitivity of many symptoms could potentially be attributable to a high proportion of false negatives, suggesting that some symptoms have limited validity for COVID-19 in children and adults. However, similarity in the predictive performance of various symptom thresholds on the two weighted scores in elderly suggests that weighting has less predictive value for this group of population. The high specificity of more symptoms could be indicative of low proportion of false positives, underlining the need to accurately assess symptoms. In practice, there is a trade-off between sensitivity and specificity such that when the consequences of having a false positive test is very serious, specificity is prioritised over sensitivity and vice-versa.[41] This is the case for the various symptom thresholds on the un-weighted scale where specificity is higher than sensitivity. A higher specificity over sensitivity is of practical relevance when the political implication of refusing to test someone with suspected COVID-19 is considered, though higher sensitivity over specificity might be given preference in the early phases of a pandemic before surge capacity is reached.

Given the rapid increase in community transmission of COVID-19 cases and deleterious impacts of instituting another lockdown (partial or complete), large-scale surveillance for capturing the epidemiological trend of COVID-19 in Nigeria is crucial. However, Nigeria has limited SARS-CoV-2 testing capacity with an average turnaround of two days, making syndromic surveillance (symptomatic monitoring) a viable complementary surveillance system. As such, our findings would be relevant in informing the design of such a surveillance system, which has been demonstrated, in Japan[42,43] and in the US,[44] to be useful in improving the understanding of COVID-19 epidemiology (often in real-time), assessing the effectiveness of public health interventions, and enhancing preparedness for the emergence of COVID-19 wave or a surge. For instance, an evaluation of a syndromic surveillance system in the US found new taste/smell loss to be highly correlated with a range of COVID-19 outcomes, highlighting their usefulness in supporting the surveillance system as an early warning system for COVID-19 prevention and control. However, the feasibility (e.g. considering selection bias and recall bias) and acceptability of a syndromic surveillance system first need to be ascertained given the large proportion of asymptomatic COVID-19 cases at diagnosis in Nigeria.[8]Positive predictive values across the various prediction thresholds, especially for the weighted scales, were generally low despite increasing proportionately with the thresholds. This could be attributable, in part, to the general mildness of the pandemic with resultant low

For peer review only - http://bmjopen.bmj.com/site/about/guidelines.xhtml

incidence of mortality in Nigeria. For instance, 66% of the 12,289 confirmed COVID-19 cases in Nigeria between February 27 and June 6, 2020 were asymptomatic at diagnosis, with an overall cumulative incidence and case fatality rate of 5·6 per 100,000 population and 2·8%, respectively[8]—these figures were substantially lower than those from European countries during the same period.[45] As such our predictive tools could perform differently during a more severe COVID-19 outbreak in Nigeria.

Conclusion

This study has investigated the possibility of using symptoms to predict COVID-19 positivity in Nigeria, and found the predictive capacity of various symptom scores to be poor overall. However, the findings have the potential to serve as an advocacy tool for more investments in resources for capacity strengthening of molecular testing for COVID-19 in Nigeria, which is crucial for improving both clinical case management and surveillance.

Acknowledgments

We acknowledge the contribution of all the COVID-19 treatment centres and laboratories within the NCDC Laboratory Networks of both epidemiological and laboratory data to SORMAS on a regular basis. We express our profound gratitude to the NCDC SORMAS team for collating and maintaining a quality dataset utilised for this study. Last but not least, we thank Professor Jo Leonard-Bee of the University of Nottingham for her input on statistical analyses.

References

1. Nigeria Centre for Disease Control. *COVID-19 Situation Report: Weekly Epidemiological Report 3 (Epi Week 44)*. Abuja, https://ncdc.gov.ng/diseases/sitreps/?cat=14&name=An update of COVID-19 outbreak in Nigeria (2020).

2. Onyeaghala AA, and Olajide I. Managing COVID-19 outbreak in Nigeria: Matters arising. *Clinical Chemistry and Laboratory Medicine* 2020; 58: 1645–1650.

3. Oladimeji O, Atiba BP, and Adeyinka DA. Leveraging polymerase chain reaction technique (Genexpert) to upscaling testing capacity for SARS-CoV-2 (COVID-19) in Nigeria: A game changer. *Pan African Medical Journal* 2020; 35: 1–2.

4. Nigeria Centre for Disease Control. NCDC Coronavirus COVID-19 Microsite: Government Laboratories, https://covid19.ncdc.gov.ng/laboratory/ (2020, accessed 6 November 2020).

5. Nkengasong J. Let Africa into the market for COVID-19 diagnostics. *Nature* 2020; 580: 565.

6. Stites EC, and Wilen CB. The Interpretation of SARS-CoV-2 Diagnostic Tests. *Med*. Epub ahead of print 21 August 2020. DOI: 10.1016/j.medj.2020.08.001.

7. Smith DS, Richey EA, and Brunetto WL. A Symptom-Based Rule for Diagnosis of COVID-19. *SN Compr Clin Med* 2020; 1–8.

8. Elimian KO, Ochu CL, Ilori E, et al. Descriptive epidemiology of coronavirus disease 2019 in Nigeria, 27 February-6 June, 2020. *Epidemiol Infect*; 148. Epub ahead of print 2020. DOI: 10.1017/S095026882000206X.

9. Wynants L, Van Calster B, Collins GS, et al. Prediction models for diagnosis and prognosis of covid-19: Systematic review and critical appraisal. *BMJ* 2020; 369: 18.

10. Abayomi A, Odukoya O, Osibogun A, et al. Presenting Symptoms and Predictors of Poor Outcomes Among 2,184 Patients with COVID-19 in Lagos State, Nigeria. *Int J Infect Dis*; 226–232.

11. Ibrahim OR, Suleiman BM, Abdullahi SB, et al. Epidemiology of COVID-19 and Predictors of Outcome in Nigeria: A Single-Center Study. *Am J Trop Med Hyg*. Epub ahead of print 26 October 2020. DOI: 10.4269/ajtmh.20-0759.

12. Elimian KO, Ochu CL, Ebhodaghe B, et al. Patient characteristics associated with COVID-19 positivity and fatality in Nigeria: retrospective cohort study. *BMJ Open* 2020; 10: 44079.

13. Nigeria Centre for Disease Control. *Case Definitions for Coronavirus Disease* . Abuja, https://covid19.ncdc.gov.ng/media/files/Case_Definition.pdf (2020, accessed 22 July 2020).

14. Nigeria Centre for Disease Control. *National Interim Guidelines for Clinical Management of COVID-19, Version 3*. Abuja, 14 March 2020.

15. World Health Organization. Laboratory testing for coronavirus disease (COVID-19) in suspected human cases: Interim guidance, 19 March 2020. *WHO*, https://www.who.int/publications-detail/laboratory-testing-for-2019-novel-

For peer review only - http://bmjopen.bmj.com/site/about/guidelines.xhtml

coronavirus-in-suspected-human-cases-20200117 (2020, accessed 1 June 2020).

16. Kronbichler A, Kresse D, Yoon S, et al. Asymptomatic patients as a source of COVID-19 infections: A systematic review and meta-analysis. *Int J Infect Dis*. Epub ahead of print 17 June 2020. DOI: 10.1016/j.ijid.2020.06.052.

17. Miri SM, Noorbakhsh F, Mohebbi SR, and Ghaemi A. Higher prevalence of asymptomatic or mild COVID-19 in children, claims and clues. *J Med Virol* 2020; jmv.26069.

18. Glantz SA, Slinker BK, and Neilands TB. *Primer of Applied Regression and Analysis of Variance* . 3rd ed. McGraw-Hill Medical, 2016.

19. Zweig M, and Campbell G. Receiver-operating characteristic (ROC) plots: A fundamental evaluation tool in clinical medicine. *Clinical Chemistry* 1993; 39: 561–577.

20. Moons KGM, Altman DG, Reitsma JB, et al. Transparent reporting of a multivariable prediction model for individual prognosis or diagnosis (TRIPOD): Explanation and elaboration. *Ann Intern Med* 2015; 162: W1–W73.

21. Nattino G, Lemeshow S, Phillips G, Finazzi S, and Bertolini G. Assessing the calibration of dichotomous outcome models with the calibration belt. *Stata J* 2017; 17: 1003–1014.

22. Salloum RG, Theis RP, Pbert L, et al. Stakeholder Engagement in Developing an Electronic Clinical Support Tool for Tobacco Prevention in Adolescent Primary Care. *Children* 2018; 5: 170.

23. Nemes S, Jonasson JM, Genell A, and Steineck G. Bias in odds ratios by logistic regression modelling and sample size. *BMC Med Res Methodol* 2009; 9: 56.

24. Katz MH. *Multivariable Analysis: A Practical Guide for Clinicians and Public Health Researchers*. 3rd Edition. Cambridge University Press, 2011.

25. Steyerberg EW, Harrell FE, Borsboom GJJM, et al. Internal validation of predictive models: Efficiency of some procedures for logistic regression analysis. *J Clin Epidemiol* 2001; 54: 774–781.

26. Van Smeden M, De Groot JAH, Moons KGM, et al. No rationale for 1 variable per 10 events criterion for binary logistic regression analysis. *BMC Med Res Methodol* 2016; 16: 1–12.

27. Wang Z, Weng J, Li Z, et al. Development and Validation of a Diagnostic Nomogram to Predict COVID-19 Pneumonia. *medRxiv* 2020; 2020.04.03.20052068.

28. Chen X, Tang Y, Mo Y, et al. A diagnostic model for coronavirus disease 2019 (COVID-19) based on radiological semantic and clinical features: a multi-center study. *Eur Radiol* 2020; 30: 4893–4902.

29. Batista AF de M, Miraglia JL, Donato THR, and Chiavegatto Filho ADP. COVID-19 diagnosis prediction in emergency care patients: a machine learning approach. *medRxiv* 2020; 2020.04.04.20052092.

30. Brinati D, Campagner A, Ferrari D, et al. Detection of COVID-19 Infection from Routine Blood Exams with Machine Learning: a Feasibility Study. *medRxiv* 2020;

For peer review only

For peer review only - http://bmjopen.bmj.com/site/about/guidelines.xhtml

2020.04.22.20075143.

31. Kurstjens S, Van Der Horst A, Herpers R, et al. Rapid identification of SARS-CoV-2-infected patients at the emergency department using routine testing. *Clin Chem Lab Med* 2020; 58: 1587–1593.

32. Tordjman M, Mekki A, Mali R, et al. Pre-test probability for SARS-Cov-2-related Infection Score: the PARIS score. *medRxiv* 2020; 2020.04.28.20081687.

33. Sun Y, Koh V, Marimuthu K, et al. Epidemiological and Clinical Predictors of COVID-19. *Clin Infect Dis* 2020; 71: 786–792.

34. Roland LT, Gurrola JG, Loftus PA, Cheung SW, and Chang JL. Smell and taste symptom-based predictive model for COVID-19 diagnosis. *Int Forum Allergy Rhinol*. Epub ahead of print 2020. DOI: 10.1002/alr.22602.

35. Zhang J, Fang Y, Lu Z, et al. Lacking Communication Would Increase General Symptom Index Scores of Medical Team Members During COVID-19 Pandemic in China: A Retrospective Cohort Study: *Inq J Heal Care Organ Provision, Financ*; 58. Epub ahead of print 22 February 2021. DOI: 10.1177/0046958021997344.

36. Menni C, Valdes AM, Freidin MB, et al. Real-time tracking of self-reported symptoms to predict potential COVID-19. *Nat Med* 2020; 26: 1037–1040.

37. Desjardins MR. Syndromic surveillance of COVID-19 using crowdsourced data. Epub ahead of print 2020. DOI: 10.1016/j.lanwpc.2020.10.

38. Borsetto D, Hopkins C, Philips V, et al. Self-reported alteration of sense of smell or taste in patients with COVID-19: a systematic review and meta-analysis on 3563 patients. *Rhinology*. Epub ahead of print 6 July 2020. DOI: 10.4193/Rhin20.185.

39. Myles PR, Nguyen-Van-Tam JS, Lim WS, et al. Comparison of CATs, CURB-65 and PMEWS as Triage Tools in Pandemic Influenza Admissions to UK Hospitals: Case Control Analysis Using Retrospective Data. *PLoS One* 2012; 7: e34428.

40. Simon R. Clinical trial designs for evaluating the medical utility of prognostic and predictive biomarkers in oncology. *Personalized Medicine* 2010; 7: 33–47.

41. Akobeng AK. Understanding diagnostic tests 3: Receiver operating characteristic curves. *Acta Paediatrica, International Journal of Paediatrics* 2007; 96: 644–647.

42. Yoneoka D, Tanoue Y, Kawashima T, et al. Large-scale epidemiological monitoring of the COVID-19 epidemic in Tokyo. *Lancet Reg Heal - West Pacific*; 3. Epub ahead of print 1 October 2020. DOI: 10.1016/j.lanwpc.2020.100016.

43. Nomura S, Yoneoka D, Shi S, et al. An assessment of self-reported COVID-19 related symptoms of 227,898 users of a social networking service in Japan: Has the regional risk changed after the declaration of the state of emergency? *Lancet Reg Heal - West Pacific* 2020; 1: 100011.

44. Güemes A, Ray S, Aboumerhi K, et al. A syndromic surveillance tool to detect anomalous clusters of COVID-19 symptoms in the United States. *Sci Rep*; 11. Epub ahead of print 2021. DOI: 10.1038/S41598-021-84145-5.

45. Kinross P, Suetens C, Gomes Dias J, et al. Rapidly increasing cumulative incidence of coronavirus disease (COVID-19) in the European Union/European Economic Area and

For peer review only - http://bmjopen.bmj.com/site/about/guidelines.xhtml

the United Kingdom, 1 January to 15 March 2020. *Eurosurveillance* 2020; 25: 2000285.

Competing interests

None declared.

Funding

This study received no specific grant from any funding agency in the public, commercial or not-for-profit sectors.

Data sharing statement

The dataset utilised for this study can be made available upon reasonable request to the Head of NCDC Research at chinwe.ochu@ncdc.gov.ng

Authors' contribution

KE contributed to the conceptualization, literature search, data management, data analysis, writing (original draft, review and editing) and formatting of document. OA contributed to the conceptualization, writing (review and editing), formatting of manuscript and funding acquisition. JG contributed to the conceptualization, data management, data analysis, writing (original draft, review and editing) and formatting of document. PM contributed to the writing (original draft, review and editing), literature search and formatting of manuscript. CO contributed to the writing (review and editing) and formatting of manuscript. CK contributed to the writing (review and editing) and formatting of manuscript. TO contributed to the writing (review and editing) and formatting of manuscript. GG contributed to the writing (review and editing) and formatting of manuscript. AO contributed to the writing (review and editing) and formatting of manuscript. HZ contributed to the writing (review and editing) and formatting of manuscript. CO contributed to the writing (review and editing) and formatting of manuscript. BE contributed to the writing (review and editing) and formatting of manuscript. CDN contributed to the writing (review and editing) and formatting of manuscript. WN contributed to the writing (review and editing) and formatting of manuscript. IAS contributed to data acquisition and curation, and writing (review) of manuscript. OA contributed to the writing (review and editing) and formatting of manuscript. OF contributed to the writing (review and editing) and formatting of manuscript. CA contributed to data acquisition and curation, and writing (review) of manuscript. CE contributed to the writing (review and editing) and

For peer review only - http://bmjopen.bmj.com/site/about/guidelines.xhtml

formatting of manuscript. JA contributed to the writing (review and editing) and formatting of manuscript. AO contributed to the writing (review and editing) and formatting of manuscript. JA contributed to the writing (review and editing) and formatting of manuscript. EJ contributed to the writing (review and editing) and formatting of manuscript. AB contributed to the writing (review and editing) and formatting of manuscript. BM contributed to the writing (review and editing) and formatting of manuscript. BAS contributed to the writing (review and editing) and formatting of manuscript. AJ contributed to the writing (review and editing) and formatting of manuscript. KH contributed to the writing (review and editing) and formatting of manuscript. CI contributed to the writing (review and editing) and formatting of manuscript. SOB contributed to the writing (review and editing) and formatting of manuscript. MA contributed to the writing (review and editing) and formatting of manuscript. NM contributed to supervision, writing (review), funding acquisition, project administration, and resources. JO contributed to supervision, writing (review), funding acquisition, project administration, and resources. EI contributed to supervision, writing (review), funding acquisition, project administration, and resources. TA contributed to the writing (review and editing) and formatting of manuscript. EI contributed to the writing (original draft, review and editing), literature search and formatting of manuscript. CI contributed to conceptualization, supervision, writing (review and editing), funding acquisition, project administration, and resources. All authors invest significant contributions and approved the final draft.

Legends

Figure 1 presents a flow chart showing the processes for selecting records utilised for this study.

Figure 2 presents a comparison of unweighted, statistically weighted and clinically weighted combined scores for predicting COVID-19 positivity. Specifically, Figure 2a focuses on children, 2b on adults, and 2c on the elderly populations.

Figure 3 presents the calibration belt and tests for children, adults and elderly.

For peer review only

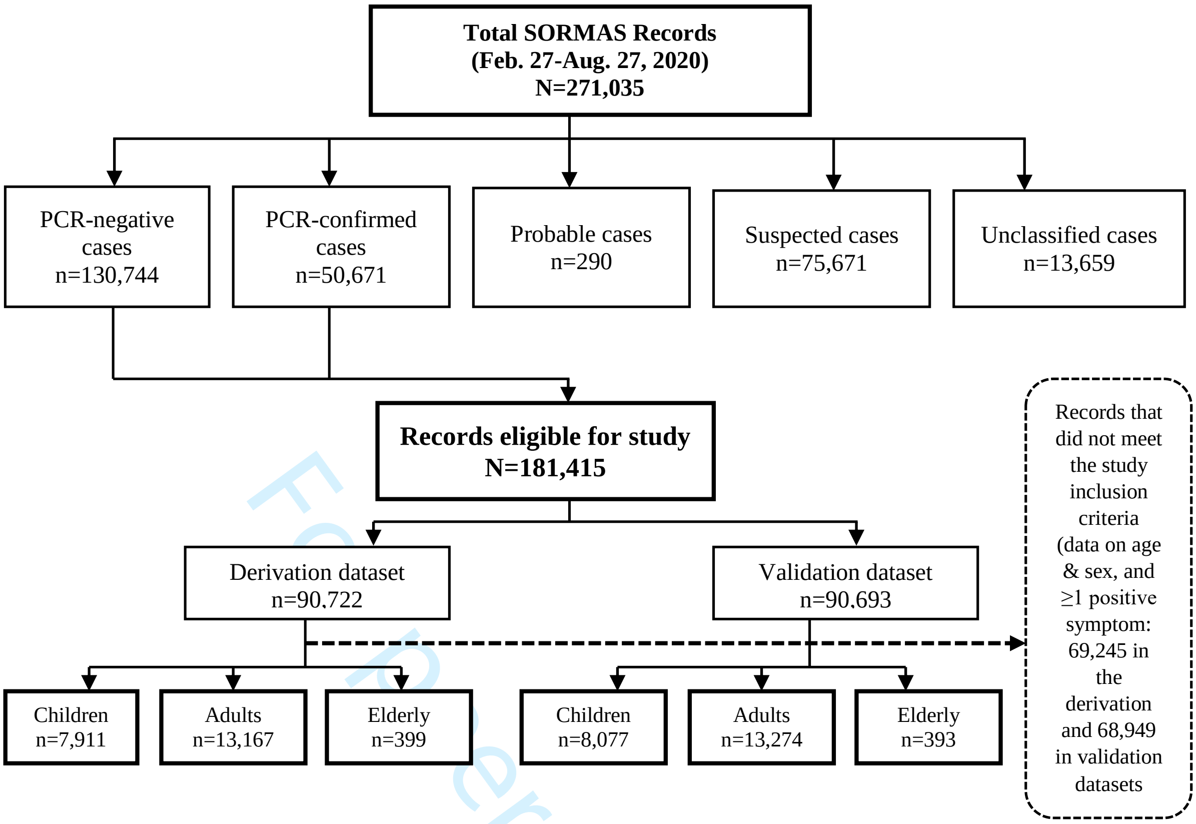

Figure 1: Flow charts showing the processes for selecting records for this study

For peer review only

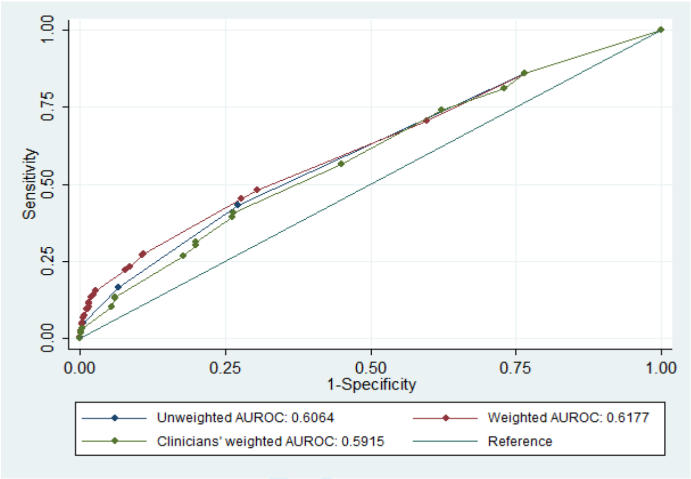

a)

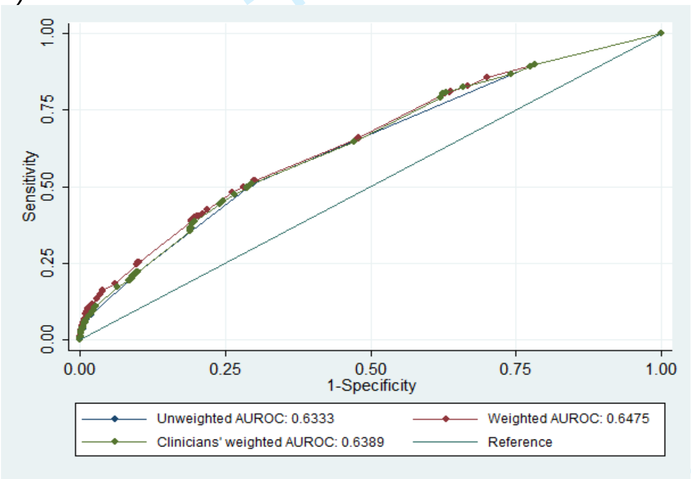

b)

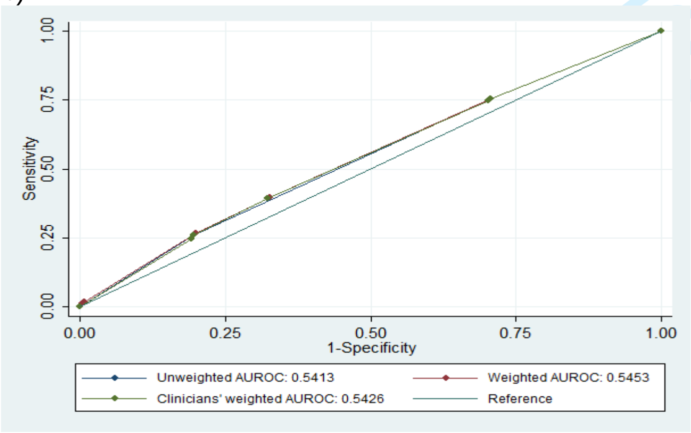

c)

Figure 2: Comparison of unweighted, statistically weighted and clinically weighted combined scores for predicting COVID-19 positivity in children (a), adults (b) and the elderly (c)

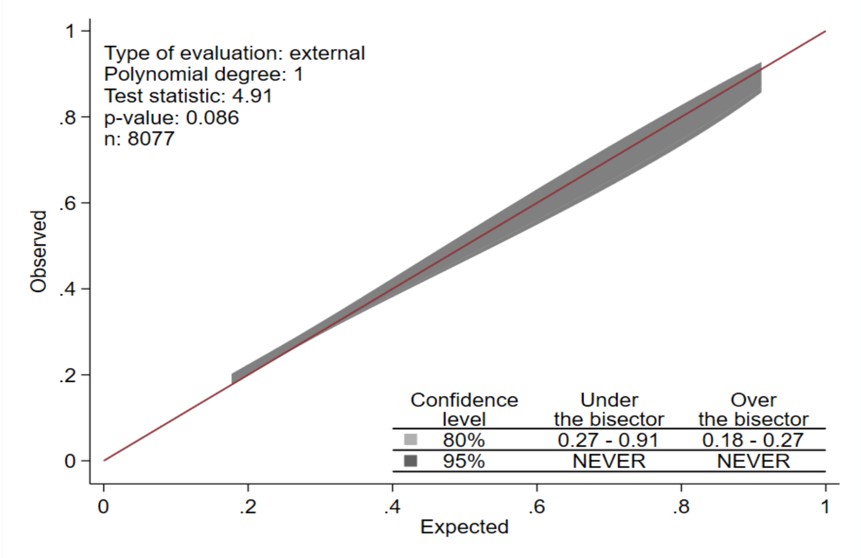

a) Children

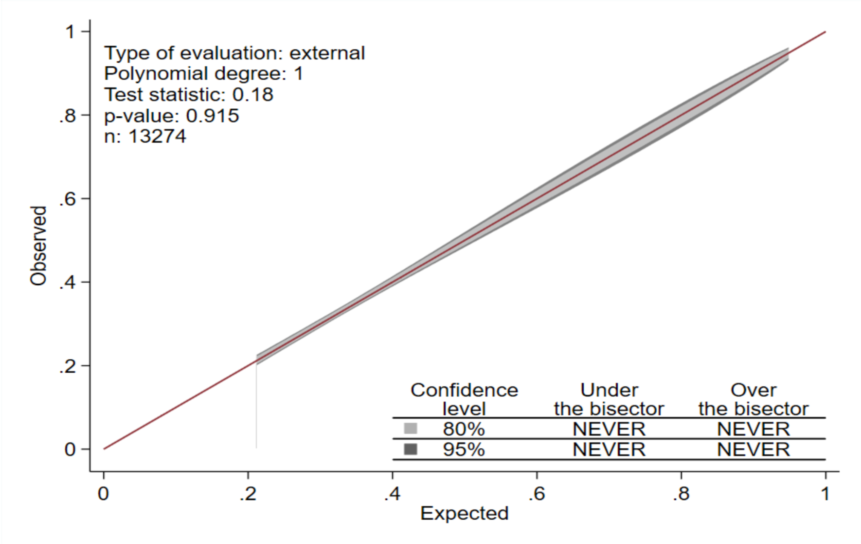

b) Adults

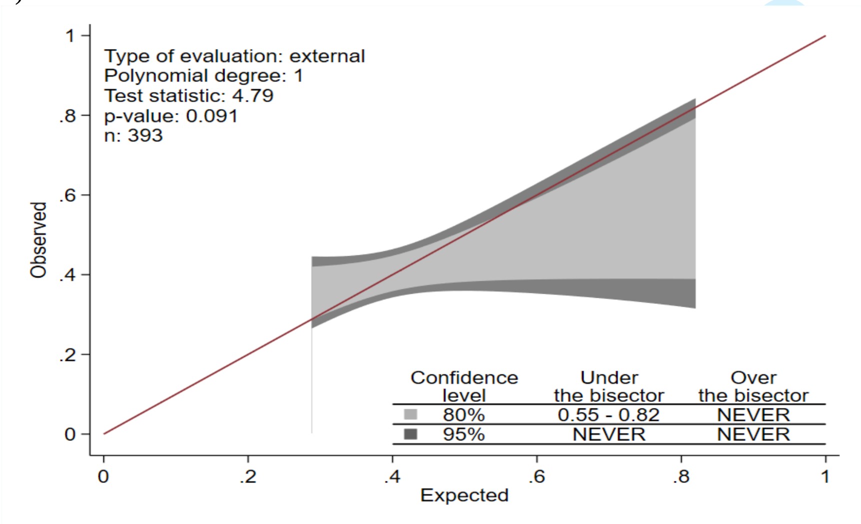

c) Elderly

Figure 3: Calibration belts and tests for children (upper panel), adults (middle panel), and elderly (lower panel)

Supplementary Materials

Supplementary Table 1: NCDC COVID-19 case definitions, February 27-August 27, 2020	
**Case category**	**Definition/criteria**
**Suspected COVID-19 case**	**1. Symptoms with international travel**<br>Anyone with acute respiratory symptoms (fever and either cough, difficulty breathing or shortness of breath) OR new respiratory symptoms (cough, difficulty breathing or shortness of breath) without fever and no other explanation, **AND** a history of travel to or residence in a country reporting cases within 14 days before symptom onset; Or new respiratory symptoms with contact to a confirmed case in the last 14 days before symptom onset;<br>OR<br>**2. Symptoms with contact to confirmed case**<br>Anyone with new respiratory symptoms (cough, difficulty breathing or shortness of breath, with or without fever), **AND** contact with a confirmed or probable COVID-19 case in the last 14 days prior to symptom onset;<br>OR<br>**3. Acute respiratory illness in an area of moderate or high COVID-19 prevalence with no other explanation**<br>Anyone with acute respiratory illness within the last 10 days (fever and either cough, difficulty breathing or shortness of breath), **AND** absence of an alternative diagnosis that explains the clinical presentation **AND** residing or working in the last 14 days in an area identified by NCDC as a moderate or high prevalence region.
**Probable case**	Any suspected case:<br>• For whom testing for COVID-19 is indeterminate test results;<br>• For whom testing was positive on a pan-coronavirus assay; OR<br>• Where samples were not collected prior to the demise of a suspect case
**Confirmed case**	Anyone with laboratory confirmation of SARS-CoV-2 infection with or without signs and symptoms.
**Contact**	Defined as anyone who experienced any one of the following exposures during the 2 days before and/or within 14 days after the onset of symptoms of a probable or confirmed case:<br>• Face-to-face contact with a probable or confirmed case within 1 metre and for more than 15 minutes;<br>• Direct physical contact with a probable or confirmed case;<br>• Direct care for a patient with probable or confirmed COVID-19 disease without using proper personal protective equipment; OR<br>• Other situations as indicated by local risk assessments.<br><br>For confirmed asymptomatic cases, the period of contact is measured as the 2 days before, through the 14 days after the date on which the sample was taken which led to confirmation.

For peer review only

Assessment of multicollinearity between clinical symptoms and COVID-19 positivity

The VIF and tolerance values (Supplementary Tables 3a, 3b and 3c for results) from the assessment of multicollinearity between the statistically significant symptoms in the unadjusted models and COVID-19 positivity in children, adults and elderly were less than 10 and greater than 0.1, implying it was appropriate to combine the independent symptoms into an index or clinical score.

Supplementary Table 2a: Findings from the assessment of multicollinearity between clinical signs and symptoms and COVID-19 diagnosis in children

Clinical sign/symptom	Derivation dataset n=7,911	
	Variance of Inflation Factor (VIF)	Tolerance
Cough	1.02	0.9800
Runny nose	1.03	0.9724
Fatigue	1.01	0.9864
Loss of taste	1.95	0.5132
Loss of smell	1.95	0.5139
Fever	1.01	0.9853
Mean VIF=1.33		

Supplementary Table 2b: Findings from the assessment of multicollinearity between clinical signs and symptoms and COVID-19 diagnosis in adults

Clinical sign/symptom	Derivation dataset n=13,167	
	Variance of Inflation Factor (VIF)	Tolerance
Cough	1.05	0.9569
Difficulty breathing	1.04	0.9613
Rapid breathing	1.01	0.9901
Runny nose	1.03	0.9734
Gastrointestinal tract symptom	1.02	0.9847
Chest pain	1.02	0.9770
Fatigue	1.02	0.9805
Headache	1.04	0.9609
Loss of taste	1.79	0.5574
Loss of smell	1.79	0.5581
Fever	1.03	0.9678
Mean VIF=1.17		

Supplementary Table 2c: Findings from the assessment of multicollinearity between clinical signs and symptoms and COVID-19 diagnosis in elderly

Clinical sign/symptom	Derivation dataset n=3,369	
	Variance of Inflation Factor (VIF)	Tolerance
Cough	1.01	0.9871
Difficulty breathing	1.02	0.9800
Loss of smell	1.01	0.9891
Mean VIF=1.01		

For peer review only - http://bmjopen.bmj.com/site/about/guidelines.xhtml

Supplementary Table 3: Assignment of clinical weights based on clinicians' judgement or experiences from managing COVID-19 cases in Nigeria

Symptom	Weighting based on clinicians' judgement						
	**Delta State**	**FCT**	**Kaduna State**	**Gombe State**	**Infectious disease consultant§**	**Infectious disease consultant¶**	**Average weight‡**
**Children**							
Cough	3	5	3	1	3	3	**3.0**
Runny nose	2	2	4	2	2	2	**2.3**
Fatigue	2	2	2	1	1	1	**1.5**
Loss of taste	1	3	2	1	1	1	**1.5**
Loss of smell	1	3	2	1	1	1	**1.5**
Fever	2	5	3	5	3	5	**3.8**
**Adults**							
Cough	5	5	4	5	3	3	**4.2**
Runny nose	3	1	2	3	2	3	**2.3**
Chest pain	3	1	2	1	3	2	**2.0**
Fatigue	5	1	3	4	2	4	**3.2**
Headache	3	2	4	1	2	1	**2.2**
Loss of taste	4	3	3	4	5	3	**3.7**
Loss of smell	5	3	3	4	5	3	**3.8**
Fever	3	5	4	4	3	5	**4.0**
**Elderly**							
Cough	4	5	4	5	3	4	**4.2**
Breathing difficulty	5	5	4	5	5	5	**4.8**
Loss of smell	5	3	3	5	5	3	**4.0**

FCT: Federal Capital Territory
‡: Sum of weights from various sources divided by 6 (i.e. number of weight sources)
§: Provides care to COVID-19 patients in a health-facility in the FCT
¶: Provides home-care to COVID-19 patients in the FCT

For peer review only - http://bmjopen.bmj.com/site/about/guidelines.xhtml

Table 4: Baseline sociodemographic and clinical characteristics of the study participants in relation to COVID-19 infection (derivation dataset)

Variable	Children		Adults		Elderly	
	PCR-confirmed cases [n=2,155 (%)]	PCR positive and negative cases [N=7,911 (%)]	PCR-confirmed cases [n=4,542 (%)]	PCR positive and negative cases [N=13,167 (%)]	PCR-confirmed cases [n=159 (%)]	PCR positive and negative cases [N=399 (%)]
**Socio-demographic features**						
**Sex**						
Female	902 (41.86)	3,065 (38.74)	1,534 (33.77)	4,814 (36.56)	49 (30.82)	148 (37.09)
Male	1,253 (58.14)	4,846 (61.26)†	3,008 (66.23)	8,353 (63.44)‡	110 (69.18)	251 (62.91)†
**Geopolitical zone§**						
South-west	632 (29.33)	1,761 (22.26)	1,285 (28.29)	3,111 (23.63)	47 (29.56)	97 (24.31)
South-south	712 (33.04)	2,823 (35.68)	1,617 (35.60)	5,441 (41.32)	51 (32.08)	164 (41.10)
South-east	46 (2.13)	129 (1.63)	139 (3.06)	294 (2.23)	11 (6.92)	16 (4.01)
North-central	402 (18.65)	1,557 (19.68)	749 (16.49)	2,045 (15.53)	13 (8.18)	32 (8.02)
North-west	315 (14.62)	1,498 (18.94)	609 (13.41)	1,978 (15.02)	20 (12.58)	63 (15.79)
North-east	48 (2.23)	143 (1.81)‡	143 (3.15)	298 (2.26)‡	17 (10.69)	27 (6.77)†
**Setting**						
Rural	111 (5.15)	429 (5.42)	233 (5.13)	755 (5.73)	20 (12.58)	41 (10.28)
Urban	1,015 (47.10)	3,467 (43.83)	2,218 (48.83)	6,485 (49.25)	92 (57.86)	209 (52.38)
Missing	1,029 (47.75)	4,015 (50.75)†	2,091 (46.04)	5,927 (45.01)†	47 (29.56)	149 (37.34)†
**Education**						
None	25 (1.16)	107 (1.35)	62 (1.37)	169 (1.28)	15 (9.43)	31 (7.77)
Nursery	16 (0.74)	70 (0.88)	2 (0.04)	4 (0.03)	0 (0.00)	0 (0.00)
Primary	67 (3.11)	250 (3.16)	41 (0.90)	151 (1.15)	2 (1.26)	5 (1.25)
Secondary	181 (8.40)	681 (8.61)	378 (8.32)	1,098 (8.34)	14 (8.81)	33 (8.27)
Tertiary	692 (32.11)	2,043 (25.82)	1,574 (34.65)	4,071 (30.92)	45 (28.30)	82 (20.55)
Other	98 (4.55)	540 (6.83)	134 (2.95)	336 (2.55)	13 (8.18)	25 (6.27)
Missing	1,076 (49.93)	4,220 (53.34)‡	2,351 (51.76)	7,338 (55.73)‡	70 (44.03)	223 (55.89)†
**Occupation**						
Student/pupil	297 (13.78)	1,401 (17.71)	212 (4.67)	775 (5.89)	1 (0.63)	2 (0.50)
Child/housewife	67 (3.11)	241 (3.05)	81 (1.78)	228 (1.73)	7 (4.40)	23 (5.76)
Business/trading	106 (4.92)	384 (4.85)	397 (8.74)	1,050 (7.97)	6 (3.77)	18 (4.51)
Transporter	6 (0.28)	35 (0.44)	24 (0.53)	87 (0.66)	0 (0.00)	1 (0.25)
Healthcare worker	269 (12.48)	950 (12.01)	479 (10.55)	1,473 (11.19)	4 (2.52)	13 (3.26)
Laboratorian	10 (0.46)	26 (0.33)	12 (0.26)	35 (0.27)	0 (0.00)	0 (0.00)
Farmer	18 (0.84)	92 (1.16)	84 (1.85)	283 (2.15)	16 (10.06)	27 (6.77)
Animal-related worker	3 (0.14)	11 (0.14)	15 (0.33)	40 (0.30)	1 (0.63)	4 (1.00)
Religious/traditional leader	2 (0.09)	11 (0.14)	30 (0.66)	69 (0.52)	2 (1.26)	4 (1.00)

Other	826 (38.33)	2,609 (32.98)	2,008 (44.21)	5,633 (42.78)	85 (53.46)	187 (46.87)
Missing	551 (25.57)	2,151 (27.19)‡	1,200 (26.42)	3,494 (26.54)‡	37 (23.27)	120 (30.08)NS
**Clinical signs and symptoms**						
**Clinical outcome**						
Recovered	1,396 (64.78)	2,221 (28.07)	2,844 (62.62)	3,677 (27.93)	70 (44.03)	89 (22.31)
Dead	15 (0.70)	24 (0.30)	229 (5.04)	252 (1.91)	50 (31.45)	50 (12.53)
No outcome yet	744 (34.52)	5,666 (71.62)‡	1,469 (32.34)	9,238 (70.16)‡	39 (24.53)	260 (65.16)‡
Obvious (visible to healthcare workers on sight)						
**Chills/sweat**						
No	2,119 (98.33)	7,791 (98.48)	4,471 (98.44)	12,978 (98.56)	158 (99.37)	393 (98.50)
Yes	36 (1.67)	120 (1.52)NS	71 (1.56)	189 (1.44)NS	1 (0.63)	6 (1.50)NS
**Cough**						
No	1,162 (53.92)	4,725 (59.73)	2,023 (44.54)	7,179 (54.52)	53 (33.33)	160 (40.10)
Yes	993 (46.08)	3,186 (40.27)‡	2,519 (55.46)	5,988 (45.48)‡	106 (66.67)	239 (59.90)†
**Breathing difficulty**						
No	1,912 (88.72)	7,031 (88.88)	3,778 (83.18)	11,181 (84.92)	102 (64.15)	283 (70.93)
Yes	243 (11.28)	880 (11.12)NS	764 (16.82)	1,986 (15.08)‡	57 (35.85)	116 (29.07)†
**Rapid breathing**						
No	2,123 (98.52)	7,802 (98.62)	4,470 (98.41)	13,008 (98.79)	155 (97.48)	392 (98.25)
Yes	32 (1.48)	109 (1.38)NS	72 (1.59)	159 (1.21)†	4 (2.52)	7 (1.75)NS
**Runny nose**						
No	1,450 (67.29)	5,817 (73.53)	3,386 (74.55)	10,218 (77.60)	130 (81.76)	330 (82.71)
Yes	705 (32.71)	2,094 (26.47)‡	1,156 (25.45)	2,949 (22.40)‡	29 (18.24)	69 (17.29)NS
Askable (can be found out by asking questions of patients/relatives)						
**Abdominal pain/diarrhoea**						
No	2,021 (93.78)	7,371 (93.17)	4,193 (92.32)	12,222 (92.82)	151 (94.97)	372 (93.23)
Yes	134 (6.22)	540 (6.83)NS	349 (7.68)	945 (7.18)NS	8 (5.03)	27 (6.77)NS
**GIT symptoms**						
No	1,942 (90.12)	7,110 (89.87)	4,048 (89.12)	11,859 (90.07)	145 (91.19)	362 (90.73)
Yes	213 (9.88)	801 (10.13)NS	494 (10.88)	1,308 (9.93)†	14 (8.81)	37 (9.27)NS
**Chest pain**						
No	2,089 (96.94)	7,711 (97.47)	4,348 (95.73)	12,759 (96.90)	153 (96.23)	387 (96.99)
Yes	66 (3.06)	200 (2.53)NS	194 (4.27)	408 (3.10)‡	6 (3.77)	12 (3.01)NS
**Fatigue**						
No	2,036 (94.48)	7,543 (95.35)	4,269 (93.99)	12,474 (94.74)	144 (90.57)	372 (93.23)
Yes	119 (5.52)	368 (4.65)†	273 (6.01)	693 (5.26)†	15 (9.43)	27 (6.77)NS
**Headache**						
No	1,829 (84.87)	6,796 (85.91)	3,880 (85.42)	11,592 (88.04)	149 (93.71)	367 (91.98)

Yes	326 (15.13)	1,115 (14.09)NS	662 (14.58)	1,575 (11.96)‡	10 (6.29)	32 (8.02)NS
**Musculoskeletal pain**						
No	2,097 (97.31)	7,710 (97.46)	4,440 (97.75)	12,878 (97.81)	152 (95.60)	386 (96.74)
Yes	58 (2.69)	201 (2.54)NS	102 (2.25)	289 (2.19)NS	7 (4.40)	13 (3.26)NS
**Sore throat**						
No	1,615 (74.94)	5,950 (75.21)	3,476 (76.53)	9,766 (74.17)	129 (81.13)	316 (79.20)
Yes	540 (25.06)	1,961 (24.79)NS	1,066 (23.47)	3,401 (25.83)‡	30 (18.87)	83 (20.80)NS
**Loss of taste**						
No	1,909 (88.58)	7,562 (95.59)	4,111 (90.51)	12,581 (95.55)	153 (96.23)	391 (97.99)
Yes	246 (11.42)	349 (4.41)‡	431 (9.49)	586 (4.45)‡	6 (3.77)	8 (2.01)†
**Loss of smell**						
No	1,851 (85.89)	7,489 (94.67)	4,067 (89.54)	12,545 (95.28)	152 (95.60)	390 (97.74)
Yes	304 (14.11)	422 (5.33)‡	475 (10.46)	622 (4.72)‡	7 (4.40)	9 (2.26)†
Measureable signs/symptoms						
**Fever**						
No	1,126 (52.25)	4,634 (58.58)	2,306 (50.77)	7,723 (58.65)	79 (49.69)	219 (54.89)
Yes	1,029 (47.75)	3,277 (41.41)‡	2,236 (49.23)	5,444 (41.35)‡	80 (50.31)	180 (45.11)NS

†=p-value<0.05; ‡=p<0.001; NS=p>0.05 or Not significant

GIT= gastrointestinal (nausea + vomiting)

Musculoskeletal pain= muscle /joint pain

§=State composition of geopolitical zones in Nigeria: South-West (Ekiti, Lagos, Ogun, Ondo, Osun and Oyo); South-South (Akwa-Ibom, Bayelsa, Cross-River, Rivers, Delta and Edo); South-East (Abia, Anambra, Ebonyi, Enugu and Imo); North-Central (Benue, Kogi, Kwara, Nasarawa, Niger, and Plateau States as well as the FCT); North-West (Jigawa, Kaduna, Kano, Katsina, Kebbi, Sokoto and Zamfara); and North-East (Adamawa, Bauchi, Borno, Gombe, Taraba and Yobe).

For peer review only - http://bmjopen.bmj.com/site/about/guidelines.xhtml

Table 5: Baseline sociodemographic and clinical characteristics of the study participants in relation to COVID-19 infection (validation dataset)

Variable	Children		Adults		Elderly	
	PCR-confirmed cases [n=2,260 (%)]	PCR positive and negative cases [N=8,077 (%)]	PCR-confirmed cases [n=4,612 (%)]	PCR positive and negative cases [N=13,274 (%)]	PCR-confirmed cases [n= 158 (%)]	PCR positive and negative cases [N=393 (%)]
**Socio-demographic features**						
**Sex**						
Female	871 (38.54)	2,986 (36.97)	1,546 (33.52)	4,808 (36.22)	61 (38.61)	151 (38.42)
Male	1,389 (61.46)	5,091 (63.03)NS	3,066 (66.48)	8,466 (63.78)‡	97 (61.39)	242 (61.58)NS
**Geopolitical zone§**						
South-west	670 (29.65)	1,778 (22.01)	1,307 (28.34)	3,134 (23.61)	39 (24.68)	88 (22.39)
South-south	723 (31.99)	2,866 (35.48)	1,611 (34.93)	5,470 (41.21)	57 (36.08)	176 (44.78)
South-east	44 (1.95)	149 (1.84)	134 (2.91)	315 (2.37)	14 (8.86)	21 (5.34)
North-central	414 (18.32)	1,499 (18.56)	777 (16.85)	2,016 (15.19)	17 (10.76)	44 (11.20)
North-west	343 (15.18)	1,620 (20.06)	649 (14.07)	2,027 (15.27)	25 (15.82)	53 (13.49)
North-east	66 (2.92)	165 (2.04)‡	134 (2.91)	312 (2.35)‡	6 (3.80)	11 (2.80)†
**Setting**						
Rural	119 (5.27)	458 (5.67)	278 (6.03)	807 (6.08)	15 (9.49)	33 (8.40)
Urban	1,033 (45.71)	3,476 (43.04)	2,269 (49.20)	6,603 (49.74)	91 (57.59)	214 (54.45)
Missing	1,108 (49.03)	4,143 (51.29)†	2,065 (44.77)	5,864 (44.18)NS	52 (32.91)	146 (37.15)NS
**Education**						
None	33 (1.46)	110 (1.36)	66 (1.43)	199 (1.50)	10 (6.33)	17 (4.33)
Nursery	14 (0.62)	73 (0.90)	1 (0.02)	3 (0.02)	0 (0.00)	0 (0.00)
Primary	63 (2.79)	270 (3.34)	61 (1.32)	171 (1.29)	6 (3.80)	13 (3.31)
Secondary	204 (9.03)	698 (8.64)	414 (8.98)	1,164 (8.77)	9 (5.70)	25 (6.36)
Tertiary	718 (31.77)	2,023 (25.05)	1,567 (33.98)	4,074 (30.69)	42 (26.58)	86 (21.88)
Other	81 (3.58)	525 (6.50)	127 (2.75)	352 (2.65)	7 (4.43)	20 (5.09)
Missing	1,147 (50.75)	4,378 (54.20)‡	2,376 (51.52)	7,311 (55.08)‡	84 (53.16)	232 (59.03)NS
**Occupation**						
Student/pupil	302 (13.36)	1,408 (17.43)	222 (4.81)	741 (5.58)	0 (0.00)	0 (0.00)
Child/housewife	66 (2.92)	248 (3.07)	97 (2.10)	272 (2.05)	6 (3.80)	19 (4.83)
Business/trading	111 (4.91)	381 (4.72)	360 (7.81)	993 (7.48)	13 (8.23)	29 (7.38)
Transporter	5 (0.22)	45 (0.56)	21 (0.46)	87 (0.66)	0 (0.00)	2 (0.51)
Healthcare worker	332 (14.69)	1,003 (12.42)	494 (10.71)	1,504 (11.33)	3 (1.90)	7 (1.78)
Laboratorian	5 (0.22)	14 (0.17)	14 (0.30)	28 (0.21)	0 (0.00)	0 (0.00)
Farmer	18 (0.80)	100 (1.24)	89 (1.93)	306 (2.31)	8 (5.06)	20 (5.09)

Animal-related worker	5 (0.22)	17 (0.21)	10 (0.22)	40 (0.30)	0 (0.00)	5 (1.27)
Religious/traditional leader	3 (0.13)	13 (0.16)	23 (0.50)	60 (0.45)	4 (2.53)	5 (1.27)
Other	807 (35.71)	2,517 (31.16)	2,064 (44.75)	5,775 (43.51)	79 (50.00)	188 (47.84)
Missing	606 (26.81)	2,331 (28.86)‡	1,218 (26.41)	3,468 (26.13)†	45 (28.48)	118 (30.03)NS
**Clinical signs and symptoms**						
**Clinical outcome**						
Recovered	1,451 (64.20)	2,222 (27.51)	2,850 (61.80)	3,636 (27.39)	80 (50.63)	95 (24.17)
Dead	21 (0.93)	27 (0.33)	256 (5.55)	297 (2.24)	33 (20.89)	34 (8.65)
No outcome yet	788 (34.87)	5,828 (72.16)‡	1,506 (32.65)	9,341 (70.37)‡	45 (28.48)	264 (67.18)‡
Obvious (visible to healthcare workers on sight)						
**Chills/sweat**						
No	2,236 (98.94)	7,973 (98.71)	4,538 (98.40	13,051 (98.32)	157 (99.37)	390 (99.24)
Yes	24 (1.06)	104 (1.29)NS	74 (1.60)	223 (1.68)NS	1 (0.63)	3 (0.76)NS
**Cough**						
No	1,183 (52.35)	4,668 (57.79)	2,107 (45.69)	7,212 (54.33)	61 (38.61)	161 (40.97)
Yes	1,077 (47.65)	3,409 (42.21)‡	2,505 (54.31)	6,062 (45.67)‡	97 (61.39)	232 (59.03)NS
**Breathing difficulty**						
No	2,024 (89.56)	7,252 (89.79)	3,771 (81.76)	11,244 (84.71)	98 (62.03)	258 (65.65)
Yes	236 (10.44)	825 (10.21)NS	841 (18.24)	2,030 (15.29)‡	60 (37.97)	135 (34.35)NS
**Rapid breathing**						
No	2,222 (98.32)	7,981 (98.81)	4,532 (98.27)	13,104 (98.72)	152 (96.20)	379 (96.44)
Yes	38 (1.68)	96 (1.19)†	80 (1.73)	170 (1.28)†	6 (3.80)	14 (3.56)NS
**Runny nose**						
No	1,607 (71.11)	6,018 (74.51)	3,406 (73.85)	10,278 (77.43)	129 (81.65)	328 (83.46)
Yes	653 (28.89)	2,059 (25.49)‡	1,206 (26.15)	2,996 (22.57)‡	29 (18.35)	65 (16.54)NS
Askable (can be found out by asking questions of patients/relatives)						
**Abdominal pain/diarrhoea**						
No	2,121 (93.85)	7,568 (93.70)	4,271 (92.61)	12,358 (93.10)	146 (92.41)	356 (90.59)
Yes	139 (6.15)	509 (6.30)NS	341 (7.39)	916 (6.90)NS	12 (7.59)	37 (9.41)NS
**GIT symptoms**						
No	2,030 (89.82)	7,265 (89.95)	4,052 (87.86)	11,907 (89.70)	137 (86.71)	343 (87.28)
Yes	230 (10.18)	812 (10.05)NS	560 (12.14)	1,367 (10.30)‡	21 (13.29)	50 (12.72)NS
**Chest pain**						
No	2,197 (97.21)	7,866 (97.39)	4,434 (96.14)	12,862 (96.90)	150 (94.94)	379 (96.44)
Yes	63 (2.79)	211 (2.61)NS	178 (3.86)	412 (3.10)‡	8 (5.06)	14 (3.56)NS
**Fatigue**						
No	2,107 (93.23)	7,686 (95.16)	4,314 (93.54)	12,540 (94.47)	148 (93.67)	378 (96.18)
Yes	153 (6.77)	391 (4.84)‡	298 (6.46)	734 (5.53)†	10 (6.33)	15 (3.82)†

For peer review only - http://bmjopen.bmj.com/site/about/guidelines.xhtml

**Headache**						
No	1,924 (85.13)	6,916 (85.63)	3,969 (86.06)	11,691 (88.07)	138 (87.34)	371 (94.40)
Yes	336 (14.87)	1,161 (14.37)NS	643 (13.94)	1,583 (11.93)‡	20 (12.66)	22 (5.60)‡
**Musculoskeletal pain**						
No	2,202 (97.43)	7,885 (97.62)	4,461 (96.73)	12,905 (97.22)	154 (97.47)	385 (97.96)
Yes	58 (2.57)	192 (2.38)NS	151 (3.27)	369 (2.78)†	4 (2.53)	8 (2.04)NS
**Sore throat**						
No	1,691 (74.82)	6,018 (74.51)	3,492 (75.72)	9,817 (73.96)	130 (82.28)	333 (84.73)
Yes	569 (25.18)	2,059 (25.49)NS	1,120 (24.28)	3,457 (26.04)‡	28 (17.72)	60 (15.27)NS
**Loss of taste**						
No	2,016 (89.20)	7,721 (95.59)	4,190 (90.85)	12,687 (95.58)	150 (94.94)	379 (96.44)
Yes	244 (10.80)	356 (4.41)‡	422 (9.15)	587 (4.42)‡	8 (5.06)	14 (3.56)NS
**Loss of smell**						
No	1,964 (86.90)	7,671 (94.97)	4,119 (89.31)	12,653 (95.32)	155 (98.10)	388 (98.73)
Yes	296 (13.10)	406 (5.03)‡	493 (10.69)	621 (4.68)‡	3 (1.90)	5 (1.27)NS
Measureable signs/symptoms						
**Fever**						
No	1,224 (54.16)	4,796 (59.38)	2,249 (48.76)	7,711 (58.09)	84 (53.16)	234 (59.54)
Yes	1,036 (45.84)	3,281 (40.62)‡	2,363 (51.24)	5,563 (41.91)‡	74 (46.84)	159 (40.46)†

†=p-value<0.05; ‡=p<0.001; NS=p>0.05 or Not significant
GIT= gastrointestinal (nausea + vomiting)
Musculoskeletal pain= muscle /joint pain
§=State composition of geopolitical zones in Nigeria: South-West (Ekiti, Lagos, Ogun, Ondo, Osun and Oyo); South-South (Akwa-Ibom, Bayelsa, Cross-River, Rivers, Delta and Edo); South-East (Abia, Anambra, Ebonyi, Enugu and Imo); North-Central (Benue, Kogi, Kwara, Nasarawa, Niger, and Plateau States as well as the FCT); North-West (Jigawa, Kaduna, Kano, Katsina, Kebbi, Sokoto and Zamfara); and North-East (Adamawa, Bauchi, Borno, Gombe, Taraba and Yobe).

For peer review only - http://bmjopen.bmj.com/site/about/guidelines.xhtml

Table 6: Predictive value of individual clinical symptoms for predicting COVID-19 positivity in children (n=8,077)

Variable	ROC area (95% CI)	Sensitivity (%) (95%CI)	Specificity (%) (95%CI)	PPV (%) (95% CI)	NPV (%) (95% CI)
**Cough**	0.54 (0.53-0.55)	**47.7 (45.6-49.7)**	59.9 (58.6-61.2)	31.6 (30.0-33.2)	**74.7 (73.4-75.9)**
**Runny nose**	0.52 (0.51-0.53)	28.9 (27.0-30.8)	75.8 (74.7-76.9)	31.7 (29.7-33.8)	73.3 (72.2-74.4)
**Fatigue**	0.51 (0.51-0.52)	6.8 (5.8-7.9)	95.9 (95.4-96.4)	39.1 (34.3-44.2)	72.6 (71.6-73.6)
**Loss of taste**	0.54 (0.54-0.55)	10.8 (9.5-12.1)	**98.1 (97.7-98.4)**	68.5 (63.4-73.3)	73.9 (72.9-74.9)
**Loss of smell**	**0.56 (0.55-0.56)**	13.1 (11.7-14.6)	**98.1 (97.7-98.4)**	**72.9 (68.3-77.2)**	74.4 (73.4-75.4)
**Fever**	0.54 (0.52-0.55)	45.8 (43.8-47.9)	61.4 (60.1-62.7)	31.6 (30.0-33.2)	74.5 (73.2-75.7)

The best predictive performance measures (AUROC, sensitivity, specificity, PPV and NPV values) for each outcome are highlighted in bold

Table 7: Predictive value of individual clinical symptoms for predicting COVID-19 positivity in adults (n=13,274)

Variable	ROC area (95% CI)	Sensitivity (%) (95%CI)	Specificity (%) (95%CI)	PPV (%) (95% CI)	NPV (%) (95% CI)
Cough	**0.57 (0.56-0.58)**	**54.3 (52.9-55.8)**	58.9 (57.9-60.0)	41.3 (40.1-42.6)	**70.8 (69.7-71.8)**
Runny nose	0.53 (0.52-0.54)	26.1 (24.9-27.4)	79.3 (78.5-80.2)	40.3 (38.5-42.0)	66.9 (65.9-67.8)
Chest pain	0.51 (0.50-0.51)	3.9 (3.3-4.5)	97.3 (96.9-97.6)	43.2 (38.4-48.1)	65.5 (64.7-66.3)
Fatigue	0.51 (0.50-0.51)	6.5 (5.8-7.2)	95.0 (94.5-95.4)	40.6 (37.0-44.3)	65.6 (64.8-66.4)
Headache	0.52 (0.51-0.52)	13.9 (13.0-15.0)	89.1 (88.5-89.8)	40.6 (38.2-43.1)	66.1 (65.2-66.9)
Loss of taste	0.54 (0.53-0.54)	9.2 (8.3-10.0)	98.1 (97.8-98.4)	71.9 (68.1-75.5)	67.0 (66.1-67.8)
Loss of smell	0.55 (0.54-0.55)	10.7 (9.8-11.6)	**98.5 (98.2-98.8)**	**79.4 (76.0-82.5)**	67.4 (66.6-68.3)
Fever	**0.57 (0.56-0.58)**	51.2 (49.8-52.7)	63.1 (62.0-64.1)	42.5 (41.2-43.8)	**70.8 (69.8-71.8)**

For peer review only - http://bmjopen.bmj.com/site/about/guidelines.xhtml

Table 8: Predictive value of individual clinical symptoms for predicting COVID-19 positivity in the elderly (n=393)

Variable	ROC area (95% CI)	Sensitivity (%) (95%CI)	Specificity (%) (95%CI)	PPV (%) (95% CI)	NPV (%) (95% CI)
Cough	0.52 (0.47-0.57)	**61.4 (53.3-69.0)**	42.6 (36.1-49.1)	41.8 (35.4-48.4)	**62.1 (54.1-69.6)**
Breathing difficulty	**0.53 (0.48-0.58)**	38.0 (30.4-46.0)	68.1 (61.7-74.0)	44.4 (35.9-53.2)	62.0 (55.8-68.0)
Loss of smell	0.51 (0.49-0.52)	1.9 (0.4-5.4)	**99.1 (97.0-99.9)**	**60.0 (14.7-94.7)**	60.1 (55.0-65.0)

Table 9: Predictive performance of un-weighted clinical score thresholds for predicting COVID-19 positivity in children, adults and elderly

Outcome	Score	ROC area (95% CI)	Sensitivity (%) (95% CI)	Specificity (%) (95% CI)	PPV (%) (95% CI)	NPV (%) (95% CI)
**Children (<17 years) n=8,077**						
**Number of symptoms**	≥1	0.55 (0.54-0.56)	**86.1 (84.6-87.5)**	23.6 (22.5-24.7)	30.4 (29.3-31.6)	**81.3 (79.4-83.2)**
	≥2	**0.58 (0.57-0.59)**	43.3 (41.2-45.3)	72.9 (71.7-74.0)	38.3 (36.4-40.2)	76.8 (75.6-77.9)
	≥3	0.55 (0.54-0.56)	16.6 (15.1-18.2)	93.4 (92.7-94.0)	49.4 (45.8-53.0)	74.2 (73.2-75.2)
	≥4	0.52 (0.52-0.53)	5.1 (4.2-6.1)	**99.5 (99.3-99.7)**	**79.3 (71.8-85.6)**	73.0 (72.0-73.9)
**Adults (17-64 years) n=13,274**						
**Number of symptoms**	≥1	0.56 (0.55-0.56)	**89.7 (88.8-90.6)**	21.7 (20.9-22.6)	37.9 (37.0-38.8)	**79.9 (78.2-81.5)**
	≥2	**0.61 (0.60-0.61)**	51.0 (49.5-52.4)	70.3 (69.3-71.2)	47.7 (46.3-49.1)	72.9 (72.0-73.9)
	≥3	0.56 (0.55-0.57)	22.3 (21.1-23.5)	90.0 (89.4-90.6)	54.3 (52.1-56.6)	68.5 (67.7-69.4)
	≥4	0.53 (0.53-0.54)	8.3 (7.5-9.1)	**98.0 (97.7-98.3)**	**68.8 (64.7-72.6)**	66.7 (65.9-67.6)
**Elderly (>65 years) n=393**						
**Number of symptoms**	≥1	0.52 (0.48-0.57)	**75.3 (67.5-81.8)**	29.4 (23.6-35.6)	41.8 (36.0-47.7)	**63.9 (54.1-72.9)**
	≥2	**0.53 (0.49-0.57)**	25.9 (19.3-33.5)	**80.4 (74.8-85.3)**	**47.1 (36.3-58.1)**	61.8 (56.1-67.2)

The best predictive performance measures (AUROC, sensitivity, specificity, PPV and NPV values) for each age group are highlighted in bold

Table 10: Predictive performance of statistically-derived weighted clinical score thresholds for predicting COVID-19 in children, adults and elderly

Outcome	Score	ROC area (95% CI)	Sensitivity (%) (95% CI)	Specificity (%) (95% CI)	PPV (%) (95% CI)	NPV (%) (95% CI)
**Children (<17 years) n=8,077**						
Number of symptoms	≥1	0.55 (0.54-0.56)	**86.1 (84.6-87.5)**	23.6 (22.5-24.7)	30.4 (29.3-31.6)	**81.3 (79.4-83.2)**
	≥2	0.55 (0.54-0.56)	**86.1 (84.6-87.5)**	23.6 (22.5-24.7)	30.4 (29.3-31.6)	**81.3 (79.4-83.2)**
	≥3	0.55 (0.54-0.57)	70.7 (68.8-72.6)	40.3 (39.5-41.5)	31.5 (30.2-32.8)	78.0 (76.4-79.4)
	≥4	**0.59 (0.58-0.60)**	48.1 (46.0-50.2)	**69.4 (68.2-70.6)**	**37.9 (36.1-39.7)**	77.5 (76.3-78.6)
**Adults (17-64 years) n=13,274**						
Number of symptoms	≥1	0.56 (0.55-0.56)	**89.7 (88.8-90.6)**	21.7 (20.9-22.6)	37.9 (37.0-38.8)	**79.9 (78.2-81.5)**
	≥2	0.58 (0.57-0.58)	85.6 (84.6-86.6)	30.0 (29.0-30.9)	39.4 (38.5-40.4)	79.6 (78.2-81.0)
	≥3	0.58 (0.57-0.59)	82.9 (81.8-84.0)	33.4 (32.4-34.4)	39.8 (38.9-40.8)	78.6 (77.2-79.9)
	≥4	**0.59 (0.58-0.59)**	80.8 (79.6-81.9)	**36.4 (35.4-37.5)**	**40.4 (39.4-41.4)**	78.1 (76.8-79.3)
**Elderly (>65 years) n=393**						
Number of symptoms	≥1	0.52 (0.48-0.57)	75.3 (67.8-81.8)	29.4 (23.6-35.6)	41.8 (36.0-47.7)	63.9 (54.1-72.9)
	≥2	0.52 (0.48-0.57)	75.3 (67.8-81.8)	29.4 (23.6-35.6)	41.8 (36.0-47.7)	63.9 (54.1-72.9)

The best predictive performance measures (AUROC, sensitivity, specificity, PPV and NPV values) for each age group are highlighted in bold

only

For peer review only - http://bmjopen.bmj.com/site/about/guidelines.xhtml

Table 11: Predictive performance of clinically-derived weighted clinical score thresholds for predicting COVID-19 in children, adults and elderly

Outcome	Score	ROC area (95% CI)	Sensitivity (%) (95% CI)	Specificity (%) (95% CI)	PPV (%) (95% CI)	NPV (%) (95% CI)
**Children (<17 years) n=8,077**						
**Number of symptoms**	≥1	0.55 (0.54-0.56)	**86.1 (84.6-87.5)**	23.6 (22.5-24.7)	30.4 (29.3-31.6)	**81.3 (79.4-83.2)**
	≥2	0.54 (0.53-0.55)	81.2 (79.6-82.8)	27.1 (25.9-28.2)	30.2 (29.0-31.4)	78.8 (76.9-80.6)
	≥3	0.56 (0.55-0.57)	74.2 (72.4-76.0)	37.7 (36.5-39.0)	31.7 (30.4-32.9)	79.0 (77.5-80.5)
	≥4	**0.57 (0.56-0.58)**	40.7 (38.6-42.7)	**73.6 (72.4-74.7)**	**37.4 (35.5-39.4)**	76.1 (75.0-77.2)
**Adults (17-64 years) n=13,274**						
**Number of symptoms**	≥1	0.56 (0.55-0.56)	**89.7 (88.8-90.6)**	21.7 (20.9-22.6)	37.9 (37.0-38.8)	**79.9 (78.2-81.5)**
	≥2	0.56 (0.55-0.56)	**89.7 (88.8-90.6)**	21.7 (20.9-22.6)	37.9 (37.0-38.8)	**79.9 (78.2-81.5)**
	≥3	**0.58 (0.58-0.59)**	82.5 (81.3-83.5)	34.1 (33.1-35.1)	40.0 (39.0-41.0)	78.5 (77.1-79.8)
	≥4	**0.58 (0.58-0.59)**	79.0 (77.8-80.2)	**37.9 (36.9-39.0**	**40.4 (39.4-41.4)**	77.2 (75.9-78.5)
**Elderly (>65 years) n=393**						
**Number of symptoms**	≥1	0.52 (0.48-0.57)	75.3 (67.8-81.8)	29.4 (23.6-35.6)	41.8 (36.0-47.7)	63.9 (54.1-72.9)
	≥2	0.52 (0.48-0.57)	75.3 (67.8-81.8)	29.4 (23.6-35.6)	41.8 (36.0-47.7)	63.9 (54.1-72.9)

The best predictive performance measures (AUROC, sensitivity, specificity, PPV and NPV values) for each age group are highlighted in bold

For peer review only - http://bmjopen.bmj.com/site/about/guidelines.xhtml

	Unweighted score	Statistically weighted score	Clinically weighted score
Children			
Adults			

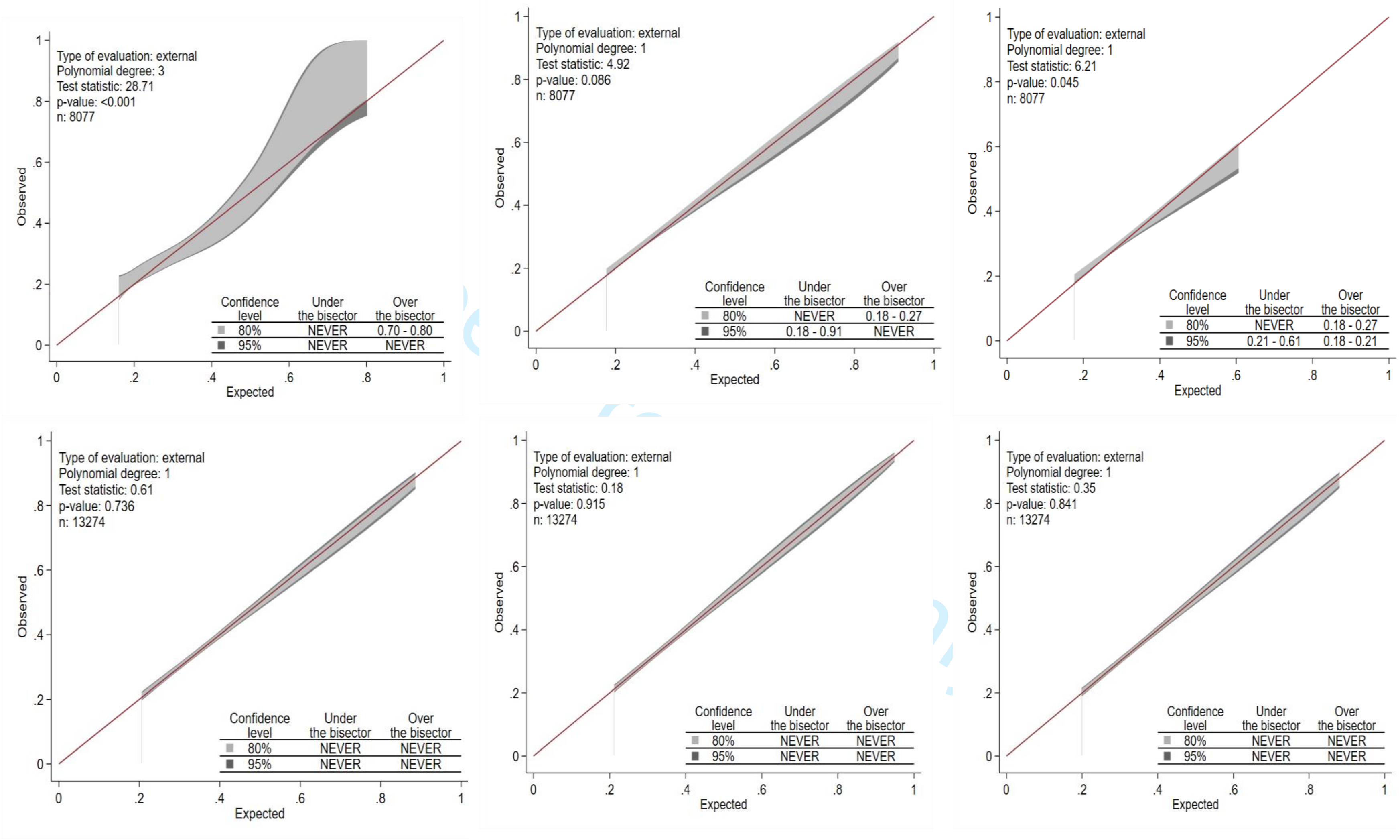

14

Elderly

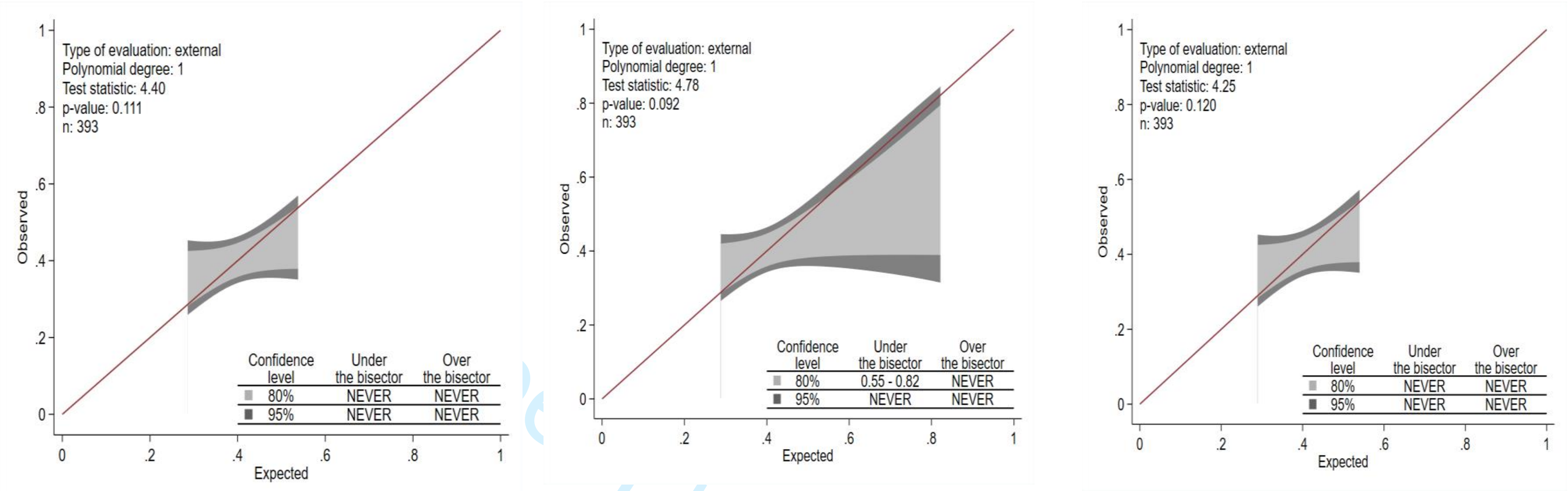

Supplementary Figure: Calibration belts and tests for children (upper panel), adults (middle panel), and elderly (lower panel)

TRIPOD Checklist: Prediction Model Development and Validation

Section/Topic	Item		Checklist Item	Page
**Title and abstract**				
Title	1	D;V	Identify the study as developing and/or validating a multivariable prediction model, the target population, and the outcome to be predicted.	Page 1
Abstract	2	D;V	Provide a summary of objectives, study design, setting, participants, sample size, predictors, outcome, statistical analysis, results, and conclusions.	Page 2; lines 43-69
**Introduction**				
Background and objectives	3a	D;V	Explain the medical context (including whether diagnostic or prognostic) and rationale for developing or validating the multivariable prediction model, including references to existing models.	Page 4; lines 105-130
	3b	D;V	Specify the objectives, including whether the study describes the development or validation of the model or both.	Page 5; lines 138-141
**Methods**				
Source of data	4a	D;V	Describe the study design or source of data (e.g., randomized trial, cohort, or registry data), separately for the development and validation data sets, if applicable.	Page 5; lines 143-149
	4b	D;V	Specify the key study dates, including start of accrual; end of accrual; and, if applicable, end of follow-up.	Page 5; lines 145-146
Participants	5a	D;V	Specify key elements of the study setting (e.g., primary care, secondary care, general population) including number and location of centres.	Page 5; lines 146-147
	5b	D;V	Describe eligibility criteria for participants.	Page 5; lines 152-154
	5c	D;V	Give details of treatments received, if relevant.	Not relevant
Outcome	6a	D;V	Clearly define the outcome that is predicted by the prediction model, including how and when assessed.	Page 6; lines 188-192
	6b	D;V	Report any actions to blind assessment of the outcome to be predicted.	Not relevant, the study being an analysis of a secondary data
Predictors	7a	D;V	Clearly define all predictors used in developing or validating the multivariable prediction model, including how and when they were measured.	Page 6; lines 192-200
	7b	D;V	Report any actions to blind assessment of predictors for the outcome and other predictors.	Not relevant, the study being an analysis of a secondary data
Sample size	8	D;V	Explain how the study size was arrived at.	Page 6; lines 169-172
Missing data	9	D;V	Describe how missing data were handled (e.g., complete-case analysis, single imputation, multiple imputation) with details of any imputation method.	Not relevant given the assumptions made prior to analyses—see page 6, lines 174-187
Statistical analysis methods	10a	D	Describe how predictors were handled in the analyses.	Page 6; lines 175-187
	10b	D	Specify type of model, all model-building procedures (including any predictor selection), and method for internal validation.	Pages 7-8; lines 206-256
	10c	V	For validation, describe how the predictions were calculated.	Page 8; lines 245-250
	10d	D;V	Specify all measures used to assess model performance and, if relevant, to compare multiple models.	Page 8; lines 245-250
	10e	V	Describe any model updating (e.g., recalibration) arising from the validation, if done.	Not done
Risk groups	11	D;V	Provide details on how risk groups were created, if done.	Not done
Development vs. validation	12	V	For validation, identify any differences from the development data in setting, eligibility criteria, outcome, and predictors.	Not relevant
**Results**				
Participants	13a	D;V	Describe the flow of participants through the study, including the number of participants with and without the outcome and, if applicable, a summary of the follow-up time. A diagram may be helpful.	Page 6; line 189
	13b	D;V	Describe the characteristics of the participants (basic demographics, clinical features, available predictors), including the number of participants with missing data for predictors and outcome.	Page 9; lines 267-276
	13c	V	For validation, show a comparison with the development data of the distribution of important variables (demographics, predictors and outcome).	Page 9; lines 275-276
Model development	14a	D	Specify the number of participants and outcome events in each analysis.	Page 9
	14b	D	If done, report the unadjusted association between each candidate predictor and outcome.	Page 13; lines 277-283
Model specification	15a	D	Present the full prediction model to allow predictions for individuals (i.e., all regression coefficients, and model intercept or baseline survival at a given time point).	Not done
	15b	D	Explain how to the use the prediction model.	Page 8; lines 246-250

For peer review only

TRIPOD Checklist: Prediction Model Development and Validation

Model performance	16	D;V	Report performance measures (with CIs) for the prediction model.	Pages 13-18; lines 284-288; lines 316-321; lines 345-350
Model-updating	17	V	If done, report the results from any model updating (i.e., model specification, model performance).	Not relevant
**Discussion**				
Limitations	18	D;V	Discuss any limitations of the study (such as nonrepresentative sample, few events per predictor, missing data).	Page 21; lines 413-426
Interpretation	19a	V	For validation, discuss the results with reference to performance in the development data, and any other validation data.	Page 21-23; lines 443-461 & 485-493
	19b	D;V	Give an overall interpretation of the results, considering objectives, limitations, results from similar studies, and other relevant evidence.	Page 21-23; lines 427-493
Implications	20	D;V	Discuss the potential clinical use of the model and implications for future research.	Page 21-23; lines 427-493
**Other information**				
Supplementary information	21	D;V	Provide information about the availability of supplementary resources, such as study protocol, Web calculator, and data sets.	Page 27; lines 634-636
Funding	22	D;V	Give the source of funding and the role of the funders for the present study.	Page 27; lines 631-633

*Items relevant only to the development of a prediction model are denoted by D, items relating solely to a validation of a prediction model are denoted by V, and items relating to both are denoted D;V. We recommend using the TRIPOD Checklist in conjunction with the TRIPOD Explanation and Elaboration document.

For peer review only
